# DAB2IP down-regulates HSP90AA1 to inhibit the malignant biological behaviors of colorectal cancer

**DOI:** 10.1186/s12885-022-09596-z

**Published:** 2022-05-19

**Authors:** Mengna Zhang, Yanan Peng, Zhenwei Yang, Hailin Zhang, Cong Xu, Lan Liu, Qiu Zhao, Jixiong Wu, Hongling Wang, Jing Liu

**Affiliations:** 1 Department of Gastroenterology, Zhongnan Hospital of Wuhan University, No. 169, Donghu Road, Wuchang District, WuhanHubei Province, 430071 China; 2 Hubei Clinical Center & Key Lab of Intestinal & Colorectal Diseases, Wuhan, 430071 China; 3 Tongji Hospital of Huazhong University of Science and Technology, Wuhan, 430030 China; 4 Department of Gastroenterology, Huanggang Central Hospital, Huangzhou District, No.11, Kaopeng Street, HuanggangHubei Province, 438000 China

**Keywords:** CRC, DAB2IP, HSP90AA1, SRP9

## Abstract

**Background:**

Studies have shown that DAB2IP inhibits cancer progression, while HSP90AA1 promotes cancer progression. However, the specific regulatory mechanism of DAB2IP and HSP90AA1 in colorectal cancer (CRC) is not clear. Our aim is to investigate the role and mechanism of DAB2IP and HSP90AA1 in the development of CRC.

**Methods:**

We used bioinformation to analyze the interaction between DAB2IP and HSP90AA1 and predict their downstream pathways. Then, a series of in vitro and in vivo experiments were conducted to reveal the role of DAB2IP and HSP90AA1 in the invasion and metastasis of colorectal cancer, and flow cytometry was used to explore their effects on apoptosis.

**Results:**

Loss of DAB2IP was associated with poor prognosis of CRC. In contrast, elevated expression of HSP90AA1 was associated with the malignant behavior of CRC. The present study demonstrated a negative correlation between DAB2IP and HSP90AA1. Using bioinformatic analysis, we scanned SRP9 which was highly expressed in CRC, as a co-related gene of DAB2IP and HSP90AA1. Mechanistically, DAB2IP promoted apoptosis through HSP90AA1/SRP9/ASK1/JNK signaling axis in CRC.

**Conclusions:**

These findings provide evidence that DAB2IP-based therapy may enhance the anticancer effect of HSP90AA1 inhibitors, and combined targeting of DAB2IP and HSP90AA1 may be a powerful treatment strategy to combat CRC.

**Supplementary Information:**

The online version contains supplementary material available at 10.1186/s12885-022-09596-z.

## Background

The incidence of colorectal cancer (CRC) is increasing globally and has an extremely high mortality rate. Activation of proto-oncogenes and inactivation of tumor suppressor genes contribute to the manipulation of CRC malignant phenotypes. Identification of essential factors and molecular mechanisms of CRC progression plays an important role in CRC treatment. In recent years, molecular targeted therapy provided new insight into the treatment of CRC [1, 2]. Drugs targeting EGFR, PD-1/PD-L1, BRAF, CTLA-4, and NTRK have been approved for the treatment of CRC patients and have achieved great benefits. Significant progress has been made in molecular targeted therapy of CRC [3]. However, the regulatory mechanisms of these critical molecules remain unclear. Therefore, it is important to elucidate the molecular mechanisms of tumorigenesis and pathogenesis of CRC.

DAB2IP is a member of the RAS-GTPase (Ras-GAP) family and interacts with the tumor suppressor DOC2/DAB2 [4]. DAB2IP is a scaffold protein that maintains cellular homeostasis by inhibiting the PI3K-Akt pathway and enhancing ASK1-JNK-mediated apoptosis to link the cascades of the survival and death signals [5]. However, DAB2IP is downregulated due to epigenetic modification in various malignancies [6–8]. Loss of DAB2IP induces malignant phenotypes of cancer cells, such as epithelial-mesenchymal transformation, tumor metastasis, and stemness through multiple signaling pathways [9, 10]. A study of Jiang Min et al. demonstrated that DAB2IP is significantly downregulated in colon cancer tissues in CRC and negatively correlated with tumor differentiation and metastasis [10]. Similarly, patients with lower DAB2IP expression had shorter overall survival times, demonstrating that DAB2IP was a potent predictive factor for the prognosis of CRC patients [10]. Nevertheless, the roles of DAB2IP-related molecules and their regulatory mechanisms in the development of CRC are still not clear.

Heat shock protein 90 (HSP90) is a highly conserved molecular chaperone that has been suggested to be a tumor promoter for the development of various cancers, including CRC [11]. Blocking HSP90 has been proven effective in suppressing multiple tumors, and a string of HSP90 inhibitors are under development [11]. Heat shock protein 90 alpha family class A member 1 (HSP90AA1), a stress-inducible member of the HSP90 family [12], regulates a range of proto-oncogene products (such as c-Myc) or important signal transduction factors during tumor pathogenesis [13, 14]. Recent studies demonstrated that HSP90AA1 facilitated tumor progression, invasion, and chemoresistance [15]. Furthermore, HSP90AA1 can act as a secreted extracellular factor involved in inflammatory responses. Tumor cells take advantage of this process to facilitate malignant phenotypes [16]. A study reported that HSP90AA1 deletion was associated with a favorable prognosis after surgery in 206 gastric cancer patients [17]. These data demonstrated that HSP90AA1 is an attractive therapeutic target. However, the specific functions and regulatory mechanisms of HSP90AA1 remain to be further researched in CRC. In the present study, we found that there was a negative correlation between DAB2IP and HSP90AA1 in CRC. Therefore, in the present study, we aimed to investigate the roles and mechanisms of the DAB2IP and HSP90AA1 nexus in the development of CRC.

## Methods

### Datasets acquisition, data analyses

In this paper, we used the gene expression profile based on the GSE8671 [18] and The Cancer Genome Atlas (https://portal.gdc.cancer.gov/). There were 32 CRC tissue and 32 normal tissues in GSE8671. We downloaded 612 TCGA samples, including 568 CRC samples and 44 normal samples. The expression levels of DAB2IP and HSP90AA1 between CRC tissue and normal tissue were analyzed. We investigated the association between DAB2IP and HSP90AA1 in three large colon cancer datasets “Tumor Colon—EXPO—315—MAS5.0—u133p2”, “Tumor Colon—Sieber—290—MAS5.0—u133p2”, “Tumor Colon—SieberSmith—355—MAS5.0—u133p2” provided by R2: Genomics Analysis and Visualization Platform (http://r2.amc.nl).

Then, we performed gene ontology (GO) analysis to disclose functional enrichment of genes related to DAB2IP or HSP90AA1 in a selected colon cancer dataset “Tumor Colon—SieberSmith—355—MAS5.0—u133p2” on the R2 platform. The intersection analysis was conducted by VENNY (https://bioinfogp.cnb.csic.es/tools/venny/). We also collected tumor tissue and corresponding paracancer tissue from 7 patients. The information of CRC patient samples was showed in Table S1. This program was admitted by the ethics committee of Zhongnan Hospital of Wuhan University (protocol #2,020,150). All subjects gave written informed consent in accordance with the Declaration of Helsinki.

### Cell culture and reagents

HT29, HCT116, SW480, DLD-1 were cultured in DMEM, and NCM460 cells were maintained in Roswell Park Memorial Institute 1640 medium (Hyclone, USA) supplemented with 10% fetal bovine serum (FBS) (GIBCO/BRL, MD, USA), 100 U/mL penicillin and 100 mg/mL streptomycin. And they were purchased from China Center for Type Culture Collection (CCTCC, Wuhan, China). The cells were cultured at 37 °C with 5% CO2. A siRNA targeting the HSP90AA1, DAB2IP, or SRP9 transcript and a nonspecific control siRNA were purchased from Guangzhou RiboBio (Guangzhou RiboBio Co, Ltd, Guangzhou, China). The target sequences were supplemented in Table S2. The specific siRNA or negative control was transfected into CRC cells with Lipofectamine 2000 transfection reagent (Invitrogen, USA) following the manufacturer's instructions. Terazosin hydrochloride, HSP90 agonist were purchased from TargetMol company.

### Plasmids transfection

pcDNA3.1( +) and pcDNA3.1( +)-DAB2IP plasmids were kindly provided by Prof. Daxing Xie from Tongji Cancer Research Institute. DAB2IP plasmids or corresponding empty vector were transfected into HCT116 and HT29 cells using Lipofectamine 2000 (Invitrogen, USA), respectively. Before transfection, cells were plated at 1.8 × 10^5^ cells/well in 6-well cell culture plates. Then 2 μg plasmid and 5 μl lipofectamine2000 were diluted in 0.2 ml Opti-MEM medium (Gibco, Life Technologies) following the manufacturer's instructions. Subsequently, cells were maintained at 37 °C for 6–8 h, and then the mixture was replaced by 2 ml of DMEM complete culture medium. Western blot and PCR were performed after 48 h to detect overexpression efficiency.

### Western blotting

Cells were lysed in lysis buffer containing protease inhibitor. Protein concentration was determined using a BCA protein assay kit (Beyotime Biotechnology Co, Ltd). Equal amounts of denatured proteins were separated by SDS-PAGE gels and then transferred onto PVDF membranes. The membranes were blocked in 5% non-fat milk at room temperature for 1.5 h and then incubated with a primary antibody, followed by a horseradish peroxidase-conjugated secondary antibody. Protein expression levels were detected using Image Lab software (Bio-Rad, USA). Immunoreactive bands were visualized with an enhanced chemiluminescent detection kit (Beyotime Biotechnology). The information on antibodies was supplemented in Table S3.

### Reverse transcription-quantitative PCR (RT-qPCR) analysis

Total RNA was harvested using Trizol reagent (Takara, Japan), and reverse transcription (RT) was performed using Transcript First-strand cDNA Synthesis SuperMix (Roche, Switzerland). The primers used in this study were designed and synthesized by Tsingke Company (Beijing, China) (shown in Supplementary Table S4). The quantitative real-time PCR (qRT-PCR) experiments were performed using SYBR-Green reagents (Takara Bio Inc., Shiga, Japan). GAPDH was used as an internal control. Fold change obtained from Ct values using 2^−ΔΔCt^ methodology was converted into logarithmic base 2 for statistical analysis. *P* values < 0.05 were considered statistically significant. Each sample was run in triplicate, and the control group was set as 1.

### Apoptosis assay

The cells were harvested in 0.25% trypsin and washed twice with PBS. The degree of apoptosis in cells was assessed using the Annexin V-PE/propidium iodide (PI) apoptosis detection kit (BD, China) by flow cytometric analysis. The experiments were performed in triplicate. The results represent the means ± SD. CytExpert software was used to analyze the ratio of apoptotic cells.

### Cell viability

Cell viability was evaluated by the Cell Counting Kit-8 (CCK-8) reagent (KeyGEN Biotech, China) according to the manufacturer’s instructions. HT29 and HCT116 cells were seeded into 96-well plates, and the total cell number was determined at the indicated time points. After adhesion, HSP90AA1 or DAB2IP siRNA was transfected into cells for 24 h, 48 h, 72 h. And then, 10 μl of CCK-8 reagent was added to each well and cultured at 37℃ for 3 h. The relative number of surviving cells was determined by measuring the optical density (O.D.) of the cell lysates at 450 nm.

### Transwell assay

Cell migration in response to increased serum concentrations was assessed using transwell chambers (8 μm pore size; Millipore, Billerica, MA, USA), according to the manufacturer's protocol. In brief, CRC cells were transfected with control-siRNA, DAB2IP-siRNA, or HSP90AA1-siRNA. Approximately 48 h after transfection, HCT116 (250 000 cells), or HT29 (200 000 cells) in 150ul DMEM were incubated on membrane inserts with 8.0-*μ*m pores in 24-well plates. Chemo-attractants (600ul DMEM containing 20% FBS) were placed in the bottom wells. After 24 h incubation at 37 °C, cells that did not migrate were removed from the top side of the inserts with a cotton swab. The filter membrane was fixed and stained with Mayer's hematoxylin (Sigma, St. Louis, MO, USA). The number of invading cells on the membrane was counted in five random fields at × 200 magnification. Experiments were repeated three times in triplicate.

### Scratch wound healing assay

Scratch wound healing assay was performed to verify that DAB2IP regulated the migration of CRC cells through HSP90AA1. Each cell line was seeded into a 6-cm dish. After 24 h, the cell sheets were stretched with a 200ul pipette tip and then treated with corresponding siRNA (100 ng/ml) in a serum-free medium for 48 h. After 0 and 48 h, three different areas of the scratched cell sheets were observed under microscopy. Wounded areas were measured using ImageJ software, and the rate of migration was calculated as a proportion of the areas at 48/0 h. All experiments were repeated three times.

### Animal studies

The 4-week-old BALB/C nude mice were purchased from Beijing Vital River Laboratory Animal Technology Company (China). Mice were randomly divided into two groups of 4 mice in each group after 1 week of environmental adaptation. The CRC cell line HCT116 was pretreated with DAB2IP plasmid and corresponding control vector for 48 h before subcutaneous injection in mice. Two hundred micrograms of vector or DAB2IP plasmids-pretreated colon cell suspension (about 5 × 10^6^ cells in PBS) were subcutaneously injected on the right flanks of male BALB/C nude mice at 5 weeks old. The body weight and the tumor size were measured every 2 days. Mice were sacrificed after 2 weeks. Tumor volume was calculated by the formula (length x width2)/2. Tumor tissues were collected and processed for western blot and immunohistochemical analysis. All animal experiments were conducted following standard procedures and approved by the Animal Ethics Committee of Wuhan University (protocol #AF217). Animal experiments are referenced from the following researches [19–22]. All the experiments were carried out by double blind method. The study was carried out in compliance with the ARRIVE guidelines.

### Statistical analysis

R (version 4.0.3) was applied for data research. The level of significance for gene expression among different types of tissues was analyzed using student t-test or Wilcox test. Gene correlation analysis was performed on the R2 platform. The Kaplan–Meier method was used for survival data processing. Statistical significance was defined as *P* < 0.05.

All the experimental results were confirmed in at least three independent experiments, and all data were presented as mean ± SD. Two-sided student’s t-tests or analysis of variance (ANOVA) tests were used to assess statistically significant differences. *P* values < 0.05 were considered statistically significant.

## Results

### DAB2IP negatively regulates the expression of HSP90AA1 in CRC

**Fig. 1:**
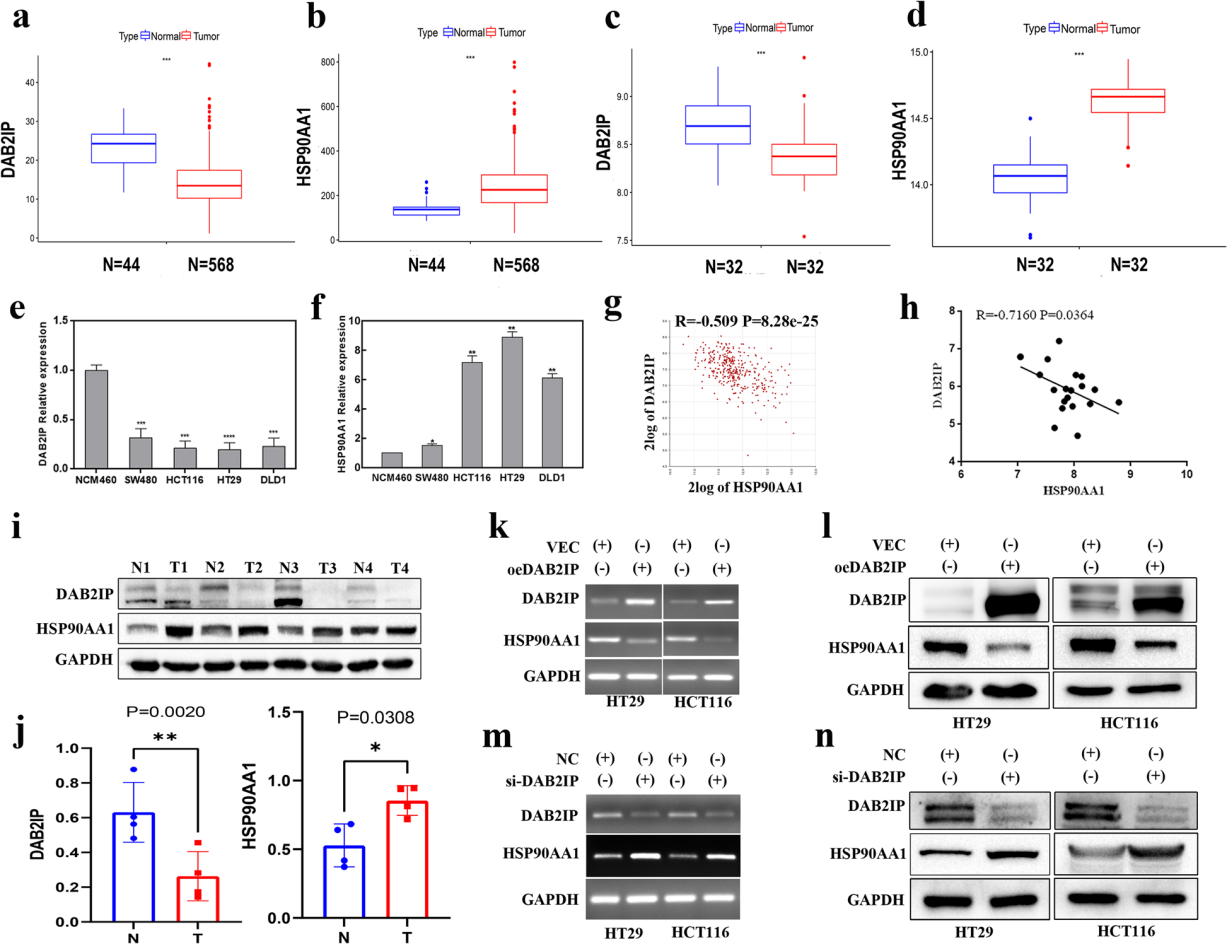
DAB2IP negatively regulates the expression of HSP90AA1 in CRC. **a** The mRNA expression level of DAB2IP in human normal tissues and colorectal cancer in the TCGA database. Wilcox test was used, *P < 0.05, ** *P* < 0.01, ***P < 0.001 and *P* < 0.05 was considered statistically significant. **b** The mRNA expression level of HSP90AA1 in human normal tissues and colorectal cancer in the TCGA database. Wilcox test was used, *P < 0.05, ** *P* < 0.01, ***P < 0.001 and *P* < 0.05 was considered statistically significant. **c** The mRNA expression level of DAB2IP in human normal colon tissue and colorectal cancer in GSE8671. Wilcox test was used, *P < 0.05, ** *P* < 0.01, ***P < 0.001 and *P* < 0.05 was considered statistically significant. **d** The mRNA expression level of HSP90AA1 in human normal colon tissue and colorectal cancer in GSE8671. Wilcox test was used, *P < 0.05, ** *P* < 0.01, ***P < 0.001 and *P* < 0.05 was considered statistically significant. **e** The mRNA expression levels of DAB2IP in normal colon epithelial cells NCM460 and CRC cell lines SW480, HT29, HCT116, and DLD1 were detected by quantitative PCR. **f** The mRNA expression levels of HSP90AA1 in normal colon epithelial cells NCM460 and CRC cell lines SW480, HT29, HCT116, and DLD1 were detected by quantitative PCR. The above RT-PCR data were expressed as mean ± SEM, *n* = 3; * *p* < 0.05, ** *p* < 0.01;*** *p* < 0.001. **g** Spearman correlation analysis of the expression of DAB2IP and HSP90AA1 in the CRC dataset "Tumor Colon—Siebersmith 355-MAS5.0-U133P2" in the R2 database. **h** Spearman correlation analysis of DAB2IP and HSP90AA1 expression in 19 human CRC samples. **i** Protein expression levels of DAB2IP and HSP90AA1 proteins in 4 pairs of CRC tissues and non-tumor colon tissues of patients. **j** Statistical analysis of protein gray value, which was quantified by Image J software. Two-sided student’s t-tests was used, * *p* < 0.05, ** *p* < 0.01;*** *p* < 0.001. **k** **l **The effect of DAB2IP overexpression on the expression of HSP90AA1 was detected by PCR and Western blotting. **m n** Changes in HSP90AA1 expression after knocking down DAB2IP were detected by PCR and Western blotting

Studies have shown that the expression of DAB2IP is down-regulated while the expression of HSP90 is upregulated in a variety of tumors [23, 24]. To determine the expression levels of DAB2IP and HSP90AA1 in CRC, we used the CRC datasets from GSE8671 and TCGA databases, and human CRC cell lines. As shown in Fig. 1a, c, the expression of DAB2IP in CRC was downregulated compared with that in normal tissue in the TCGA and GSE8671 database. The expression of HSP90AA1 was upregulated in CRC tissue (Fig. 1b, d). DAB2IP expression was downregulated in CRC cell lines compared to normal colon epithelial cells (Fig. 1e). HSP90AA1 expression was upregulated in CRC cell lines (Fig. 1f).

We divided colorectal cancer patients from TCGA into two groups according to the median expression of HSP90AA1, and analyzed the relationship between HSP90AA1 and gender, Methylation subtype, microsatellite instability (MSI) status, Pathologic stage, TNM stage, race and age, as shown in Supplementary Table 5. The results showed that HSP90AA1 expression was significantly correlated with methylation subtype, MSI status and race. HSP90AA1 is highly expressed in high CpG island methylator phenotype (CIMP-H) and Cluster 4 in Methylation Subtype (*P* < 0.001), MSI-H in MSI Status (*P* < 0.001) and white in Race (*P* < 0.001).

Spearman correlation analysis was used to analyze the correlation between DAB2IP and HSP90AA1 expression in CRC datasets. As shown in Fig. 1g and Figure S1, DAB2IP was negatively correlated with HSP90AA1 (*R* = -0.509, *P* = 8.28e-25). Furthermore, we used 19 samples of CRC patients for correlation verification (Fig. 1h), and found a significant negative correlation between DAB2IP and HSP90AA1 (*R* = -0.716, *P* = 0.0364). These results indicated that there was a negative correlation between DAB2IP and HSP90AA1 at the mRNA level. In addition, the protein expression of DAB2IP in CRC tissues was downregulated while the expression of HSP90AA1 was upregulated compared with that in normal tissues (Fig. 1i, j).

To further explore the regulatory relationship between DAB2IP and HSP90AA1, we conducted in vitro experiments in HT29 and HCT116 cells. We first detected the changes in the mRNA and protein levels of HSP90AA1 by overexpression of DAB2IP. DAB2IP overexpression significantly downregulated the expression of HSP90AA1 (Fig. 1k, l). In contrast, knocking down DAB2IP with siRNA significantly increased the expression of HSP90AA1 (Fig. 1m, n). These results indicated that DAB2IP negatively regulated the expression of HSP90AA1.

### DAB2IP counteracts the proliferation, migration, and apoptosis of CRC cell lines induced by HSP90AA1

Recent studies have shown that the absence of DAB2IP or increased expression of HSP90 promotes malignant behavior in various tumors [23, 25]. Therefore, we further explored whether DAB2IP could affect the malignant biological behaviors of CRC cells induced by HSP90AA1. DAB2IP or HSP90AA1 were silenced by siRNA in CRC cells, respectively. The down-regulation efficiency of DAB2IP or HSP90AA1 gene was confirmed by Western blotting (Figure S5a, b).

**Fig. 2:**
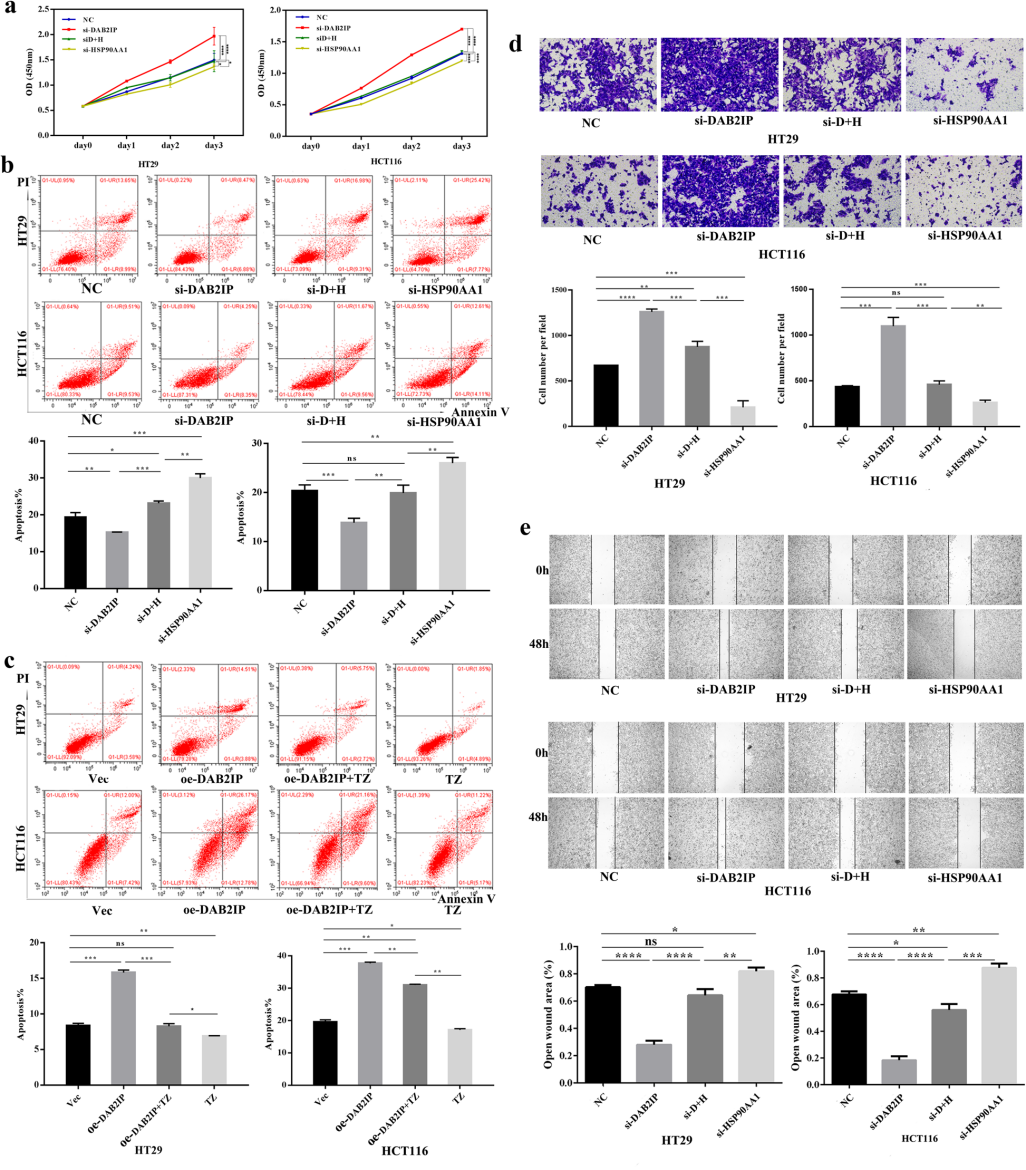
DAB2IP regulates the malignant behavior of CRC cells through HSP90AA1. **a** CCK8 assay demonstrated that DAB2IP regulated the proliferation of CRC cells by HSP90AA1. *P < 0.05, ** *P* < 0.01, ***P < 0.001, ****P < 0.0001. The data were expressed as mean ± SD, *n* = 3. **b c** Apoptosis of CRC cells was regulated by DAB2IP/ HSP90AA1 signal pathway, quantification at the bottom. Apoptosis was analyzed by measuring Annexin V-PE positive cells by flow cytometry. *P < 0.05, ** *P* < 0.01, ***P < 0.001, ****P < 0.0001. The data were expressed as mean ± SD, *n* = 3. **d e** Transwell assay and Wound scratch assay demonstrated that DAB2IP regulated the migration of CRC cells by regulating HSP90AA1 expression, quantification at the bottom. *P < 0.05, ** *P* < 0.01, ***P < 0.001, ****P < 0.0001. The data were expressed as mean ± SD, *n* = 3

The CCK-8 assay showed that silence of DAB2IP promoted the proliferation of CRC cells, and knockdown of HSP90AA1 inhibited the proliferation of CRC cells (Fig. 2a). The rescue experiment demonstrated that knockdown of HSP90AA1 could restore the proliferation of CRC cells induced by silence of DAB2IP, suggesting that DAB2IP could offset the increased proliferation of CRC cell lines induced by HSP90AA1. Besides, HSP90AA1 knockdown restored the apoptosis of CRC cells reduced by downregulation of DAB2IP expression (Fig. 2b). The rescue experiment manifested that DAB2IP also could compensate the apoptosis of CRC cell lines decreased by HSP90AA1. Also, application of terazosin (TZ), an activator of HSP90 [26, 27], reduced the apoptosis rate in the DAB2IP overexpressing cells (Fig. 2c). Furthermore, transwell assays and scratch wound healing assays showed that silence of DAB2IP prompted the migration of CRC cells, while knockdown of HSP90AA1 suppressed the migration (Fig. 2d). HSP90AA1 knockdown rescued the increased migration of CRC cells induced by DAB2IP silencing (Fig. 2e). These data indicated that DAB2IP could counteract the migration of CRC cell lines raised via HSP90AA1 activation. Taken together, these results demonstrated that DAB2IP alleviated the malignant phenotypes of CRC caused by HSP90AA1, including proliferation, apoptosis and migration.

### SRP9 is a differentially expressed co-related gene of DAB2IP and HSP90AA1 in CRC

**Fig. 3:**
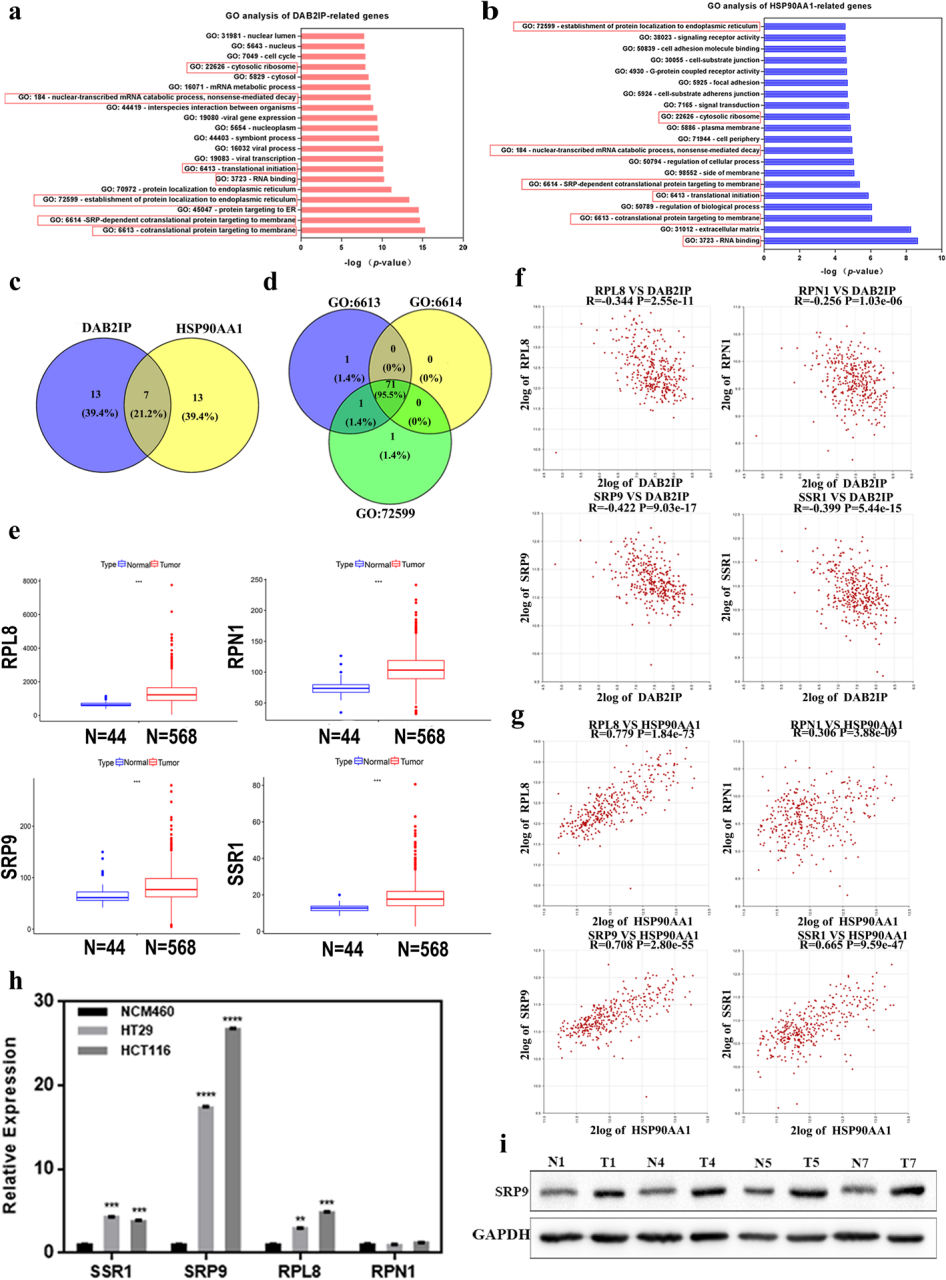
SRP9 is a co-related differentially expressed gene of DAB2IP and HSP90AA1 in CRC. **a** The results of related genes of DAB2IP in the colon cancer dataset "Tumor Colon—Siebersmith 355-MAS5.0-U133P2" by Gene Ontology (GO) analysis. **b** The results of related genes of HSP90AA1 in the colon cancer dataset "Tumor Colon—Siebersmith 355-MAS5.0-U133P2" by GO analysis. **c** Venn analysis of GO terms of DAB2IP and HSP90AA1 related genes gained 7 common GO terms. **d** Venn analysis of three common GO terms with high coincidence degree gained 71 co-related genes. **e** Four differentially expressed genes were screened from 71 co-related genes of DAB2IP and HSP90AA1, RPL8, RPN1, SRP9, SSR1 mRNA expression levels in human normal tissues and colorectal cancer in the TCGA database. Wilcox test was used, *P < 0.05, ** *P* < 0.01, ***P < 0.001 and *P* < 0.05 was considered statistically significant. **f** Verification of correlation between 4 differentially expressed genes and DAB2IP in R2 database. **g** Verification of correlation between 4 differentially expressed genes and HSP90AA1 in R2 database. **h** Real-time PCR was used to detect the expression levels of four differentially expressed genes in normal intestinal epithelial cells (NCM460 cells) and CRC cell lines (HCT116 and HT29 cells). **i** Protein expression levels of SRP9 in 4 pairs of CRC tissues and adjacent normal tissues from samples of CRC patients

To further explore molecular mechanisms in the occurrence and development of colorectal cancer, 355 CRC samples were used for Gene Ontology (GO) analysis of DAB2IP or HSP90AA1 related genes respectively (Fig. 3a, b). The related genes were obtained on the R2 platform based on Spearman analysis. GO analysis is a Gene Ontology analysis, which covers three aspects of biology: cellular components, molecular functions, and biological processes [28]. DAB2IP related genes in CRC were mainly involved in the cytosolic ribosome, nuclear-transcribed mRNA catabolic process, protein targeting to the ER, the establishment of protein localization to the endoplasmic reticulum, translational initiation, RNA binding, SRP-dependent co-translational protein targeting to the membrane, co-translational protein targeting to the membrane and other processes. The genes related to HSP90AA1 were mainly involved in the cytosolic ribosome and nuclear-transcribed mRNA catabolic process, regulation of the biological process, regulation of the cellular process, translational initiation, RNA binding, the establishment of protein localization to the endoplasmic reticulum, SRP-dependent co-translational protein targeting to the membrane, co-translational protein targeting to the membrane and other processes.

Venn analysis presented that there were 7 identical GO terms in which DAB2IP and HSP90AA1 related genes were both involved (Fig. 3c). They were cytosolic ribosome, nuclear-transcribed mRNA catabolic process, translational initiation, RNA binding, the establishment of protein localization to the endoplasmic reticulum, SRP-dependent co-translational protein targeting to the membrane, co-translational protein targeting to the membrane. These results suggested that DAB2IP and HSP90AA1 might be involved in the same physiological function and pathway in CRC. Interestingly, there was a high degree of gene coincidence in three common GO terms, namely, co-translational protein targeting to the membrane, SRP-dependent co-translational protein targeting to the membrane, and establishment of protein localization to the endoplasmic reticulum (Figure S3). The intersection of these three highly coincident GO terms (Fig. 3d) obtained 71 identical genes (the red in Figure S3), which were screened as the co-related genes of DAB2IP and HSP90AA1. Then we analyzed the expression levels of the 71 co-related genes in CRC and normal tissues in the GSE8671 and TCGA databases. Compared with normal tissues, SSR1, SRP9, RPL8, and RPN1 were highly expressed in CRC tissues (Fig. 3e, Figure S4). SSR1, SRP9, RPL8, and RPN1 were screened as the co-related differentially expressed genes of DAB2IP and HSP90AA1. To verify the correlations between these four co-related genes and DAB2IP or HSP90AA1, we performed spearman correlation analysis in the R2 database. The results suggested that SSR1, SRP9, RPL8, and RPN1 were all negatively correlated with DAB2IP (Fig. 3f) but positively correlated with HSP90AA1 (Fig. 3g). The String database (https://cn.string-db.org/) is a database that studies the interactions between proteins. We found that DAB2IP may interact with HSP90AA1, SRP9, RPN1, SSR1 and RPL8 at the protein level by seting confidence as 0.4 (Figure S6).

Furthermore, we compared the expression levels of SSR1, SRP9, RPL8, and RPN1 in CRC cell lines (HT29 and HCT116) with normal colon epithelial cells (NCM460). SSR1, SRP9, and RPL8 were all upregulated in CRC cell lines (Fig. 3h), while there was no significant difference in RPN1 expression. As SRP9 was highest expressed in CRC cell lines compared to other 3 co-related genes and had a strong correlation with both DAB2IP and HSP90AA1, it was selected as the best candidate gene for further research. We verified the expression level of SRP9 was significantly upregulated in CRC (Fig. 3i).

### DAB2IP negatively regulates the expression of SRP9 through HSP90AA1

**Fig. 4:**
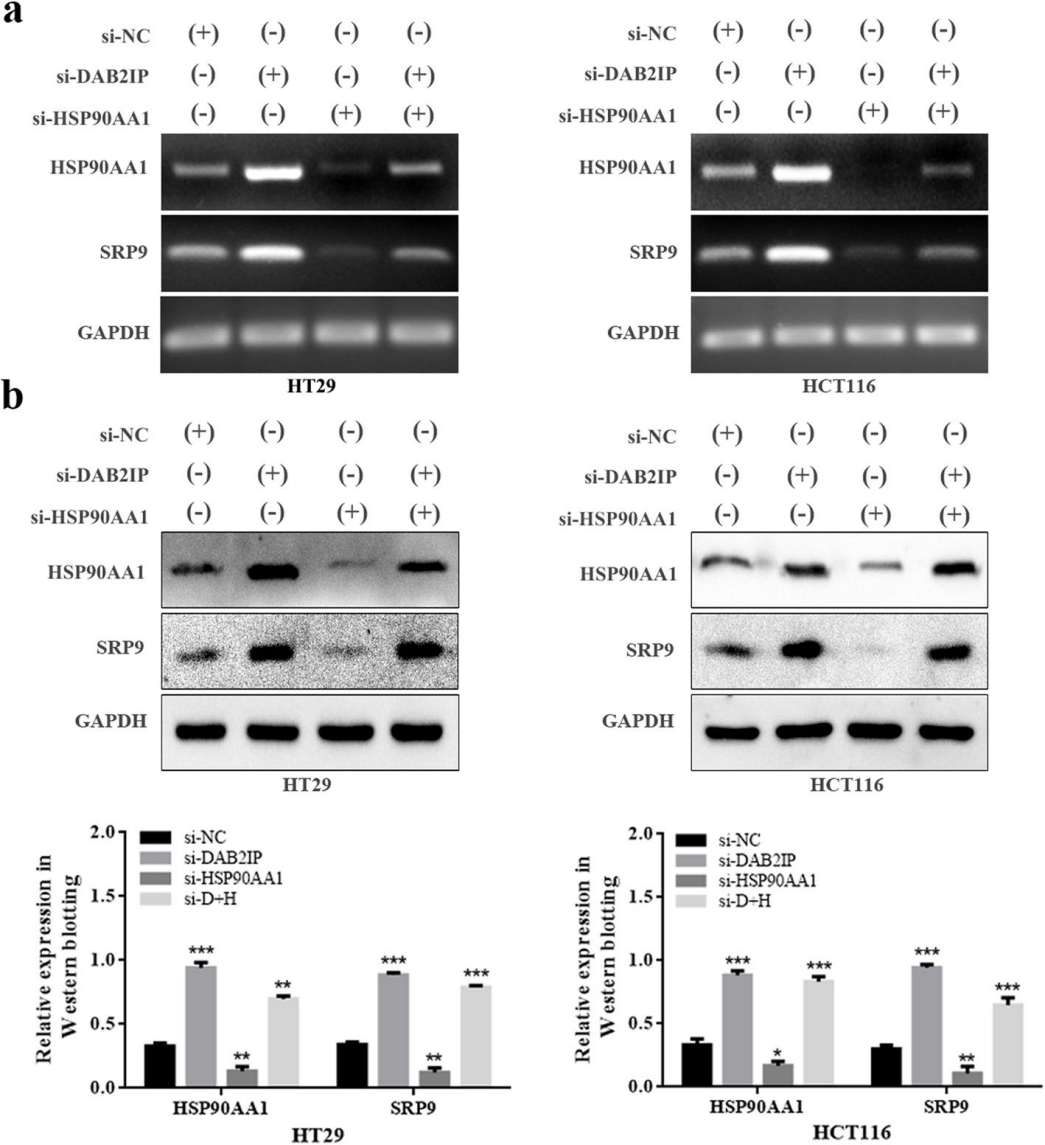
DAB2IP negatively regulates the expression of SRP9 through HSP90AA1. **a** Changes of SRP9 expression detected by PCR under different treatments, including NC, siDAB2IP, and siDAB2IP + siHSP90AA1, siHSP90AA1. **b** Changes of SRP9 expression detected by Western blotting under different treatments, including NC, siDAB2IP, and siDAB2IP + siHSP90AA1, siHSP90AA1. The gray intensity of protein expression in HT29 and HCT116 cells were quantified by Image J software. Data was presented as mean ± SD, *n* = 3; *P < 0.05, **P < 0.01, ***P < 0.001

Previous study has shown that the increased expression of SRP9 in colorectal cancer is highly likely to play a promoting cancer role [29], but its function, regulatory mechanism, and effect on tumor cells remain unclear. Correlation analysis showed that SRP9 was negatively correlated with DAB2IP but positively correlated with HSP90AA1. Next, we conducted to study the regulatory mechanism of SRP9. Silencing DAB2IP increased the expression of SRP9, while silencing of HSP90AA1 decreased SRP9 expression in CRC cell lines (Fig. 4a, b). In addition, rescue experiments had proved that the expression of SRP9 was largely reversed by silencing HSP90AA1 in DAB2IP-knockdown cells (Fig. 4a, b), confirming that DAB2IP regulated the expression of SRP9 through HSP90AA1. Additionally, consensus molecular subtype (CMS) classification is interesting in CRC [30]. We investigated the differences in the expression of DAB2IP, HSP90AA1 and SRP9 among different subtypes of CMS classification in colon cancer. As shown in Figure S7, DAB2IP and SRP9 showed no significant difference in expression among different CMS types, but HSP90AA1 expression in CMS1 and CMS4 was significantly higher than that in CMS2 and CMS3.

### DAB2IP regulates apoptosis through HSP90AA1/SRP9/ASK1/JNK axis in CRC cells

**Fig. 5:**
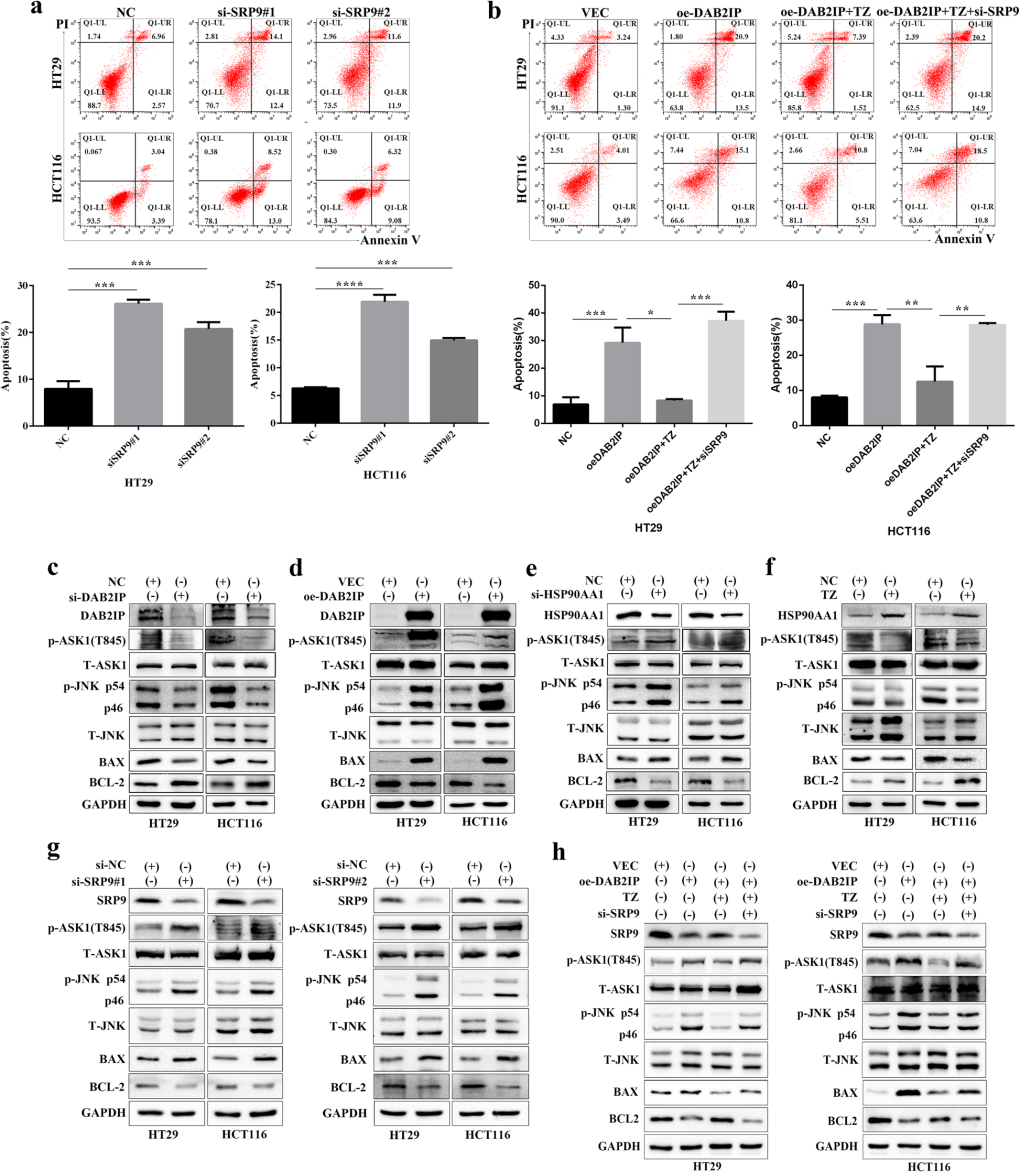
DAB2IP regulates the apoptosis of CRC cells through HSP90AA1/SRP9/ASK1/JNK axis. **a** Apoptosis detection after SRP9 silencing by flow cytometry in CRC cell lines. Apoptosis was analyzed by flow cytometry. *P < 0.05, ** *P* < 0.01, ***P < 0.001, ****P < 0.0001. The data were expressed as mean ± SD, *n* = 3. **b** Apoptosis of CRC cells was regulated by DAB2IP/HSP90AA1/SRP9 signal pathway. Apoptosis was analyzed by flow cytometry. *P < 0.05, ** *P* < 0.01, ***P < 0.001, ****P < 0.0001. The data were expressed as mean ± SD, *n* = 3. **c d** Overexpression of DAB2IP promoted apoptosis or knockdown of DAB2IP decreased apoptosis through the ASK1/JNK pathway. **e f** Knockdown of HSP90AA1 increased apoptosis or activation of HSP90AA1 by TZ inhibited apoptosis through the ASK1/JNK pathway. **g** Knockdown of SRP9 increased apoptosis through the ASK1/JNK pathway. **h** Loss of DAB2IP impeded apoptosis through HSP90AA1/SRP9/ASK1/JNK axis in CRC cells

We further investigated whether SRP9 played a role in the apoptosis modulation of CRC cell lines (HT29 and HCT116). We silenced SRP9 by siRNA and the down-regulation efficiency of SRP9 gene was showed in Figure S5c. As shown in Fig. 5a, the apoptosis of CRC cell lines was increased upon SRP9 knockdown. We further verified whether DAB2IP regulated the apoptosis of CRC cells through the HSP90AA1/SRP9 signaling axis. We found that activation of HSP90AA1 reduced the cell apoptosis based on DAB2IP overexpression. The above effect mediated by DAB2IP/HSP90AA1 was recovered via downregulation of SRP9 (Fig. 5b). These results suggested that HSP90AA1 and SRP9 inhibited tumor cell apoptosis, while DAB2IP promoted tumor cell apoptosis. Next, we explored the specific molecular mechanism in regulating apoptosis. Previous studies have demonstrated that DAB2IP and HSP90 play opposite roles in coordinating cell apoptosis through the ASK1/JNK signaling pathway [5, 31]. In CRC, few studies show the effects of DAB2IP or HSP90AA1 on the ASK1/JNK pathway. We observed that DAB2IP knockdown or activation of HSP90AA1 reduced the expression of phosphorylated ASK1, phosphorylated JNK, and the apoptotic protein BAX but increased the anti-apoptotic protein BCL-2 (Fig. 5c, f). DAB2IP overexpression or silence of HSP90AA1 had an opposite effect (Fig. 5d, e). These results suggested that DAB2IP and HSP90AA1 exerted their physiological functions through the ASK1/JNK pathway. Furthermore, western blot analysis indicated that phosphorylated ASK1, phosphorylated JNK and BAX expression were increased, whereas BCL-2 level was decreased in SRP9-knockdown cells compared with their counterparts, demonstrating that SRP9 regulated apoptosis also through the ASK1/JNK pathway in CRC cell lines (Fig. 5g). Next, we conducted rescue experiments, as showed in Fig. 5h, overexpression of DAB2IP increasing the expression of phosphorylated ASK1 and JNK and the apoptotic protein BAX and decreasing the anti-apoptotic protein BCL-2. Then we found that HSP90AA1 activation decreased the expression of phosphorylated ASK1 and JNK and the apoptotic protein BAX and increased the anti-apoptotic protein BCL-2 in DAB2IP overexpression cells. Finally, we found the expression of phosphorylated ASK1 and JNK and the apoptotic protein BAX were increased but the anti-apoptotic protein BCL-2 was decreased in oe-DAB2IP + TZ + si-SRP9 group. Combined with our previous conclusion that DAB2IP regulates SRP9 expression through HSP90AA1, we proved that DAB2IP regulated apoptosis through the HSP90AA1/SRP9/ASK1/JNK signaling axis.

### Overexpression of DAB2IP decreases HSP90AA1 expression and inhibits the progression of CRC in vivo

**Fig. 6:**
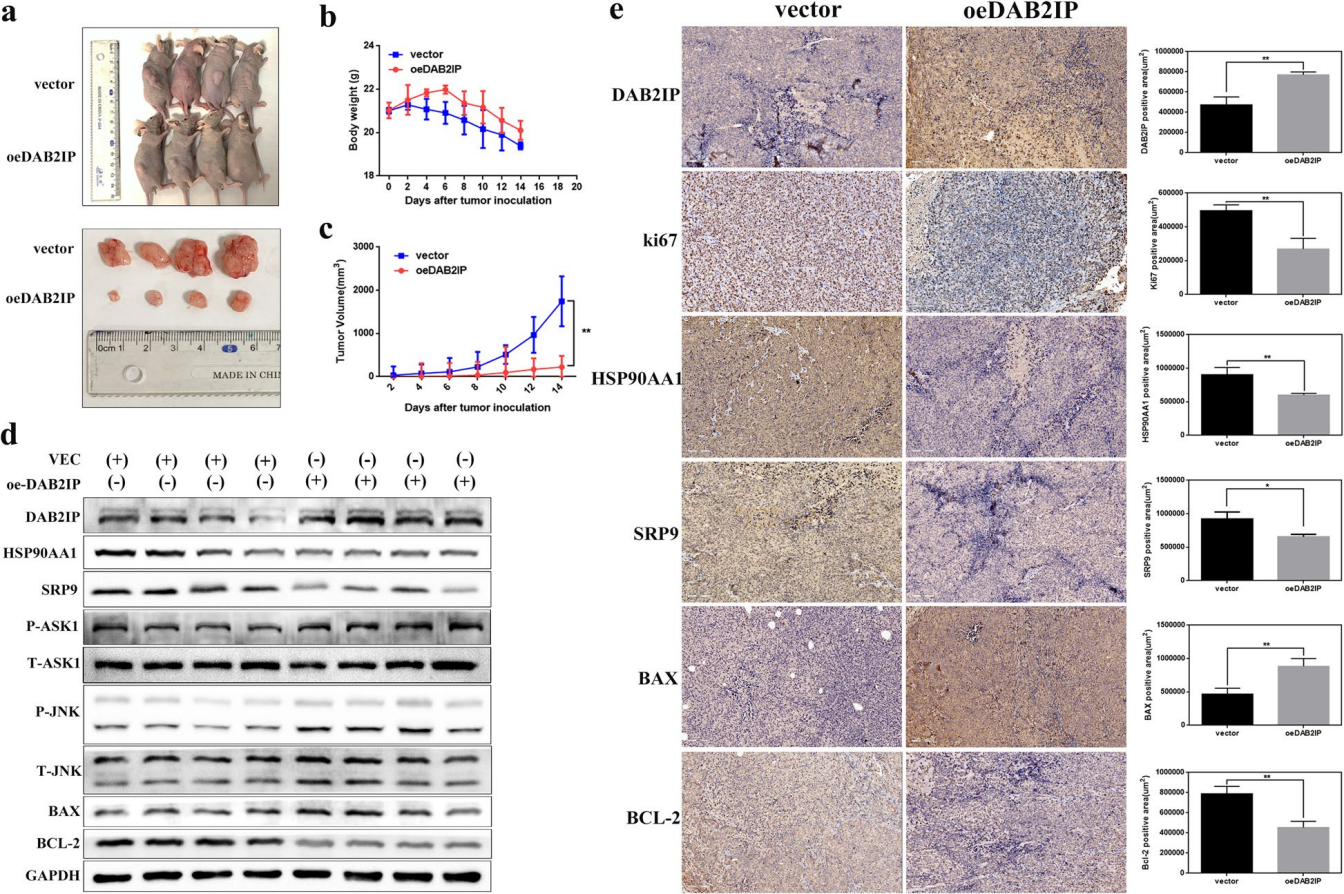
DAB2IP overexpression reduces the expression of HSP90AA1 and hinders the progression of colorectal cancer in vivo. **a** Tumor images of DAB2IP-overexpression and control nude mice (*n* = 4/group). **b** Bodyweight of the DAB2IP-overexpression and control groups. The body weight was measured every other day. **c** Tumor volumes of the DAB2IP-overexpression and control groups. The tumors were measured every 2 days. **d** HSP90AA1, SRP9, p-ASK1, p-JNK, BAX, BCL-2 expression in xenograft tumors transfected with DAB2IP plasmids or vector by Western blotting. **e** Ki-67 staining and immunohistochemical analysis of HSP90AA1 and apoptosis proteins BAX, BCL-2 (200 x) in xenograft tumors transfected with DAB2IP plasmids or vector. For each treatment, 3 independent fields from different biopsies were executed. *P < 0.05; **P < 0.01; ***P < 0.001

**Fig. 7:**
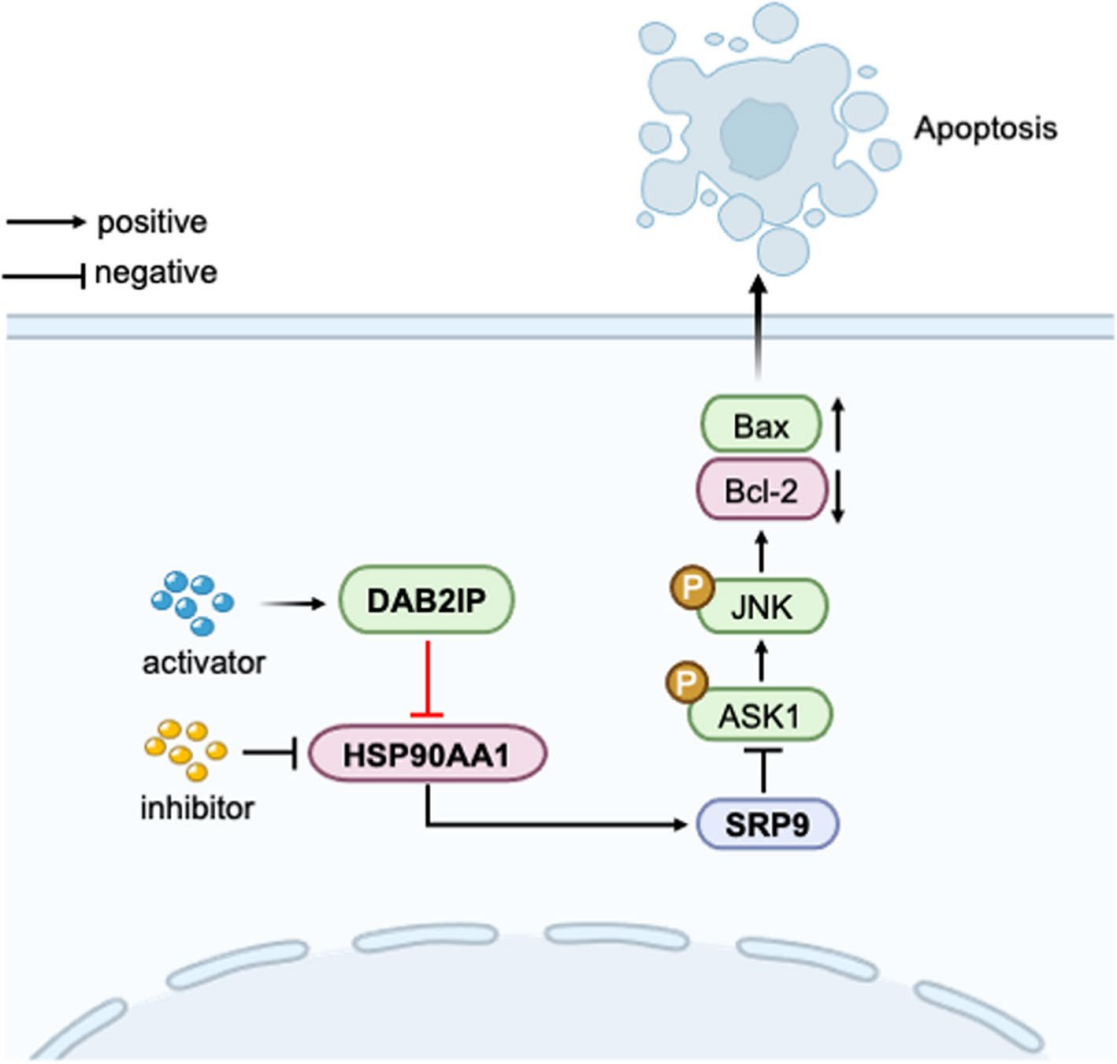
Schematic model showing the role of DAB2IP in the regulation of apoptosis

To verify the results in vivo, we constructed a CRC xenograft model in nude mice. We pretreated HCT116 cells with DAB2IP plasmids or the corresponding empty vector before subcutaneous injection into BALB/c nude mice. Tumor volumes in nude mice implanted with DAB2IP-overexpressing HCT116 cells were significantly smaller than those in the corresponding control group (Fig. 6a). Consistently, the rate of tumor growth in the DAB2IP-overexpressing group was also considerably slower than that in the vector group (Fig. 6c). However, there was no significant difference in weight loss between the two groups (Fig. 6b). Western blot and immunohistochemistry analysis validated that overexpression of DAB2IP decreased the expression levels of HSP90AA1, SRP9, and BCL-2 while increasing the levels of p-ASK1, p-JNK, and BAX in vivo (Fig. 6d, e). We also found that the expression of Ki-67, a marker of proliferation, decreased in the DAB2IP plasmid-treated group (Fig. 6e). In conclusion, our results suggested that DAB2IP could inhibit the expression of HSP90AA1 and suppress the malignant phenotypes and tumorigenicity of CRC cancer cells both in vitro and in vivo (Fig. 7).

## Discussion

Researches have demonstrated that the absence of DAB2IP in tumors is significantly related to poor patient survival [10, 32, 33]. However, overexpression of HSP90AA1 in multiple cancers was associated with tumorigenesis and development, making it a potential target for cancer therapy [12, 34–36]. In this study, we proved that DAB2IP negatively regulated the expression of HSP90AA1. In vitro studies proved that knockdown of HSP90AA1 offset the apoptosis, proliferation, and metastasis affected by DAB2IP knockdown, indicating that DAB2IP and HSP90AA1 are involved in the regulation of the same physiological process in CRC. In addition, SRP9 was identified as a co-related gene of DAB2IP and HSP90AA1, the upregulation of which negatively affected apoptosis in CRC. Furthermore, in vitro experiments demonstrated that DAB2IP regulated apoptosis through the HSP90AA1/SRP9/ASK1/JNK signaling axis.

DAB2IP acts as an adapter that negatively regulates multiple signaling pathways in cell signal transduction, contributing to maintaining cell homeostasis [23]. HSP90 is one of the most widely tested targets for cancer treatment. Studies have reported that inhibiting HSP90 can reverse the malignant phenotypes of cancer cells [37–39], and some drug-resistant tumors still show significant sensitivity to HSP90 inhibitors [40–42]. Despite numerous promising studies on HSP90 inhibitors, they have not been approved for clinical application due to unfavorable side effects. The key to solving this problem is to explore the molecular and cellular mechanisms of HSP90 family members. As a member of the HSP90 family, HSP90AA1was also shown l as a potential therapeutic target [12]. When cells were stimulated or homeostasis was disrupted, the level of HSP90AA1 increased [43, 44]. In our study, we first proved that DAB2IP suppressed the expression and function of HSP90AA1. The loss of DAB2IP resulted in constant HSP90AA1 overexpression, which promoted the malignant phenotypes of tumors. From a therapeutic perspective, activation of DAB2IP may enhance the effects of HSP90 inhibitors, or the combination of DAB2IP activation and HSP90 inhibitors may decrease the dose of HSP90 inhibitors, reducing the side effects without impairing efficacy.

In order to find the downstream regulation mechanism of DAB2IP and HSP90AA1 in CRC, we took the intersection of GO semantics of DAB2IP and HSP90AA1 related genes to obtain the three most related GO terms. The genes in these 3 GO terms were intersected again to obtain 71 identical genes. Finally, the expression levels of these 71 genes in normal colon tissues and CRC were checked, and 4 differentially expressed genes of SRP9, RPN1, SSR1, and RPL8 were obtained. Then we found that DAB2IP have interaction relationship with HSP90AA1, SRP9, RPN1, SSR1, RPL8 at the protein level in the STRING database. Then the expression levels of 4 genes were detected in normal intestinal epithelial cells and different CRC cell lines, and it was found that SRP9 was significantly differentially expressed and the results were the same in CRC patient specimens. Based on the above, we selected SRP9 as a further study object.

In this study, we identified SRP9 as a co-related gene of DAB2IP and HSP90AA1. SRP9 is a subunit of signal recognition particles (SRPs) [45–47]. Studies have shown that under stress conditions, the binding of SRP9/14 and 40S ribosomes promotes the formation of stress particles contributing to the cell survival [48]. Most studies thus far, have been limited to the SRP9 molecular structure level. Nevertheless, a few studies explored the role and mechanism of SRP9 in tumors. For instance, in breast cancer, SRP9/14 functions as an RNA binding protein to bind RN7SL1, shielding the virus-like inflammatory response [49]. In addition, SRP9 expression is upregulated in liver cancer [50]. More importantly, proteomic analysis of CRC tissues and validation of clinical specimens suggested that SRP9 acted as a potential biomarker for colorectal cancer [29]. In our study, we sufficiently proved that the expression of SRP9 was upregulated in CRC, which was consistent with a previous report detected in surgical human colorectal cancer tissues [29].

As a co-related gene, SRP9 expression was negatively regulated by DAB2IP through HSP90AA1. The results widened the range of DAB2IP as a signal modulation and revealed a regulatory relationship between HSP90AA1 and SRP9. HSP90AA1 is a stress-related protein, and its expression is upregulated under cellular stress [12]. Coincidentally, the upregulation of SRP9 is also related to the cellular stress response [48]. Thus, we inferred that and HSP90AA1 might be involved in cellular stress by upregulating the expression of SRP9.

SRP9 played a key role in the stress response to inhibit cell apoptosis and promote cell survival [48]. In our study, we found that knockdown of SRP9 promoted the apoptosis of CRC cells, suggesting that SRP9 has an inhibitory effect on apoptosis. In addition, SRP9 knockdown increased the phosphorylation of ASK1 and JNK, and the apoptotic protein Bax, and decreased the expression of Bcl-2, which confirmed that SRP9 could inhibit apoptosis through the ASK1/JNK pathway. However, the specific mechanisms need to be explored further.

According to our results, DAB2IP, HSP90AA1, and SRP9 participate in the regulation of apoptosis through the ASK1/JNK pathway. It is well known that DAB2IP interacts with the ASK1/JNK pathway to regulate apoptosis [5]. Elevated expression of HSP90AA1 and SRP9 under stress protect cells from apoptosis, but the mechanisms remain unclear. In our study, we finally proved that DAB2IP regulated apoptosis through the HSP90AA1/SRP9/ASK1/JNK signaling axis. Our results provide new insights into the mechanisms by which DAB2IP deficiency and HSP90AA1 overexpression mediate malignant behavior in CRC cells. Moreover, SRP9 might be a potential biomarker for colorectal cancer. However, the functional and molecular mechanisms of SRP9 need to be further studied.

There are still significant limitations in our study. We proved that DAB2IP negatively regulated the expression of HSP90AA1, and that HSP90AA1 positively regulated SRP9 expression, but we did not figure out the specific regulatory mechanisms. In addition, at the functional level, although we studied DAB2IP regulated the malignant phenotypes of CRC through HSP90AA1, including proliferation, migration, and apoptosis, the effect on more malignant phenotypes needs to be further explored. At the mechanistic level, we believed that the mechanism of DAB2IP, HSP90AA1 and SRP9 in regulating apoptosis was worthy of further investigation. According to the results, we proposed that the combined targeting of DAB2IP and HSP90AA1 could be an effective treatment of CRC, which should be further verified in more in-depth experimental studies and multicenter clinical trials.

In conclusion, our results identified that the regulatory relationship between DAB2IP and HSP90AA1 had critical implications on malignant manners of CRC. We provided new evidence for therapeutic approaches using DAB2IP agonists and HSP90 inhibitors to treat or prevent CRC. We also unraveled the underlying mechanisms of DAB2IP, HSP90AA1 and their co-related gene, SRP9 in regulating apoptosis of CRC cells.

## Supplementary Information

**Additional file 1.**

**Additional file 2.**

**Additional file 3. **

## Data Availability

The data of this study are available from the corresponding author upon request. The gene expression profile based on the GSE867118 and The Cancer Genome Atlas (https://portal.gdc.cancer.gov/). Three large colon cancer datasets “Tumor Colon—EXPO—315—MAS5.0—u133p2”, “Tumor Colon—Sieber—290—MAS5.0—u133p2”, “Tumor Colon—SieberSmith—355—MAS5.0—u133p2” provided by R2: Genomics Analysis and Visualization Platform (http://r2.amc.nl).
