# Supplementary material for: DAB2IP down-regulates HSP90AA1 to inhibit the malignant biological behaviors of colorectal cancer
Source: BMC Cancer. 2022 May 19;22:561. doi: 10.1186/s12885-022-09596-z (PMC9118737; doi:10.1186/s12885-022-09596-z)

**Supplementary Information**

**Figures and Figure legends**


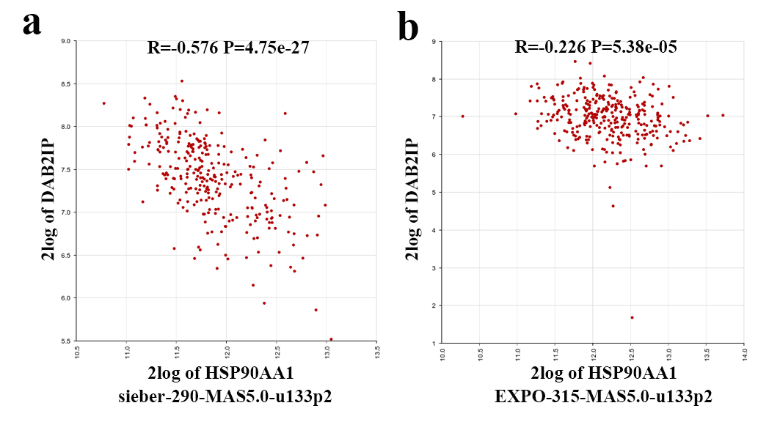


**Figure S1.** **Correlation analysis of DAB2IP and HSP90AA1 in “sieber-290-MAS5.0-u133p2” and “EXPO-315-MAS5.0-u133p2” datasets.**


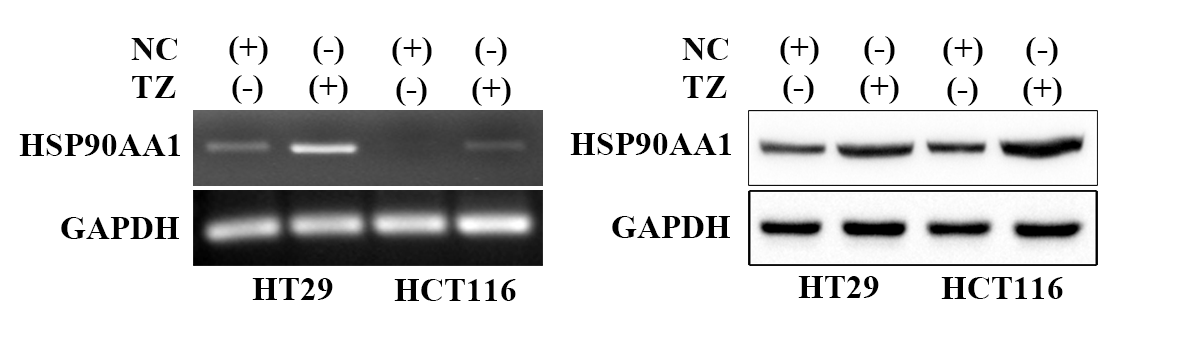


**Figure S2.** **Changes in mRNA and protein expression of HSP90AA1 after terazosin treatment.** Cells were treated with TZ at a concentration of 50uM for 24h. mRNA and protein were extracted to detect the expression level of HSP90AA1.


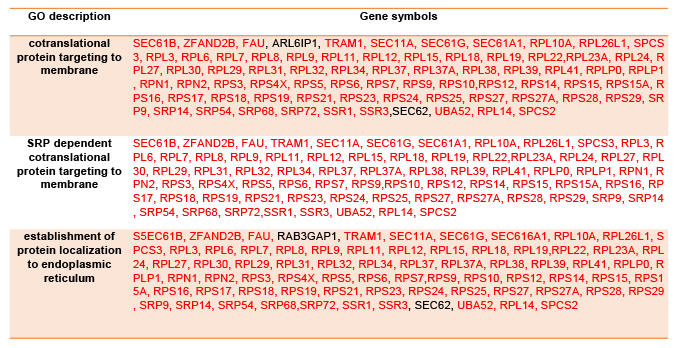


**Figure S3.** **The specific genes in three highly coincident GO terms.** Red represented the same genes in three GO terms, and black represented different genes.

**
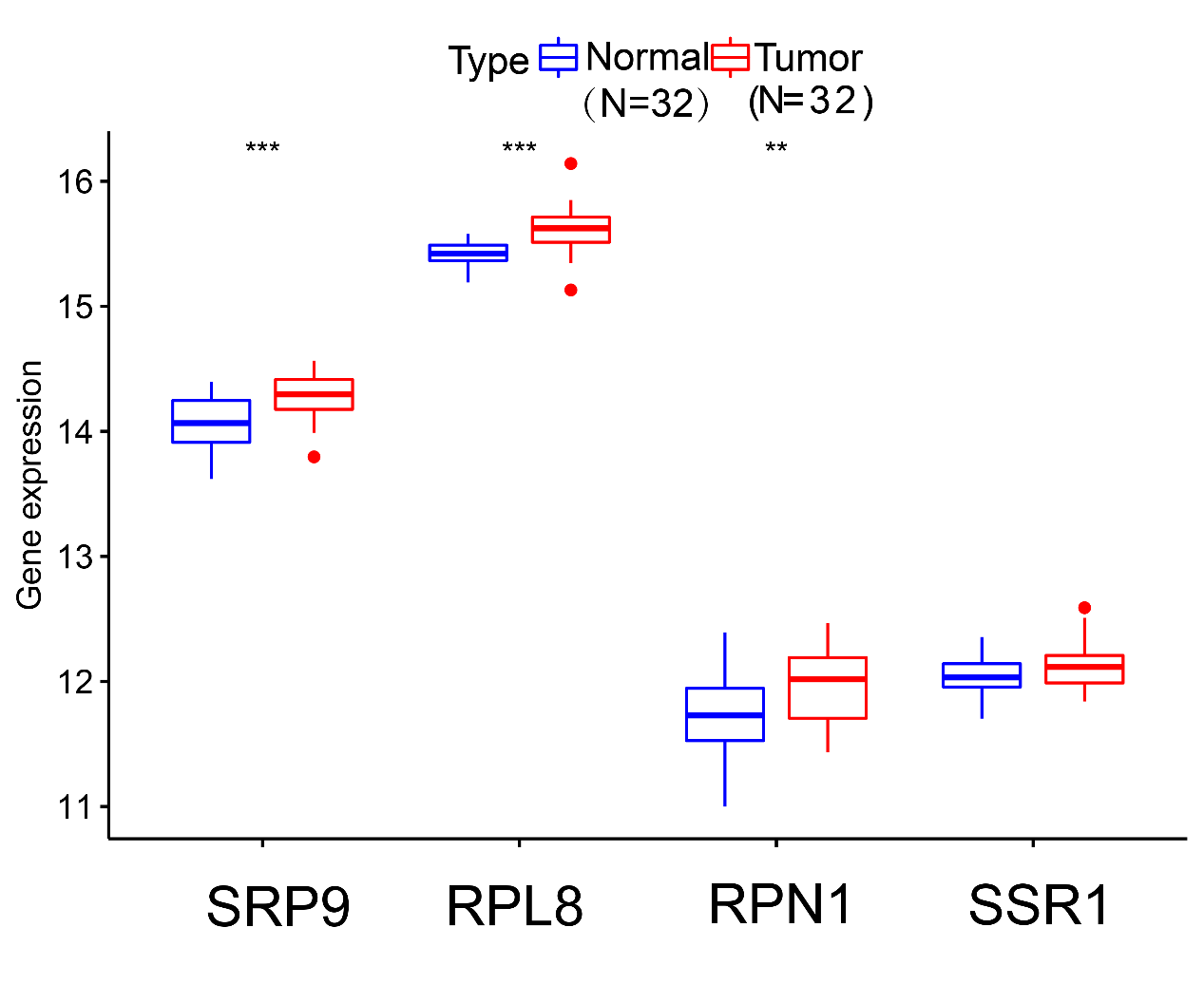
**

**Figure S4.** **Differentially expressed genes were verified in GSE8671.** The mRNA expression level of SRP9, RPL8, RPN1 and SSR1 in human normal colon tissue and colorectal cancer in GSE8671. Wilcox test was used, *P<0.05, ** P<0.01, ***P<0.001 and P<0.05 was considered statistically significant.


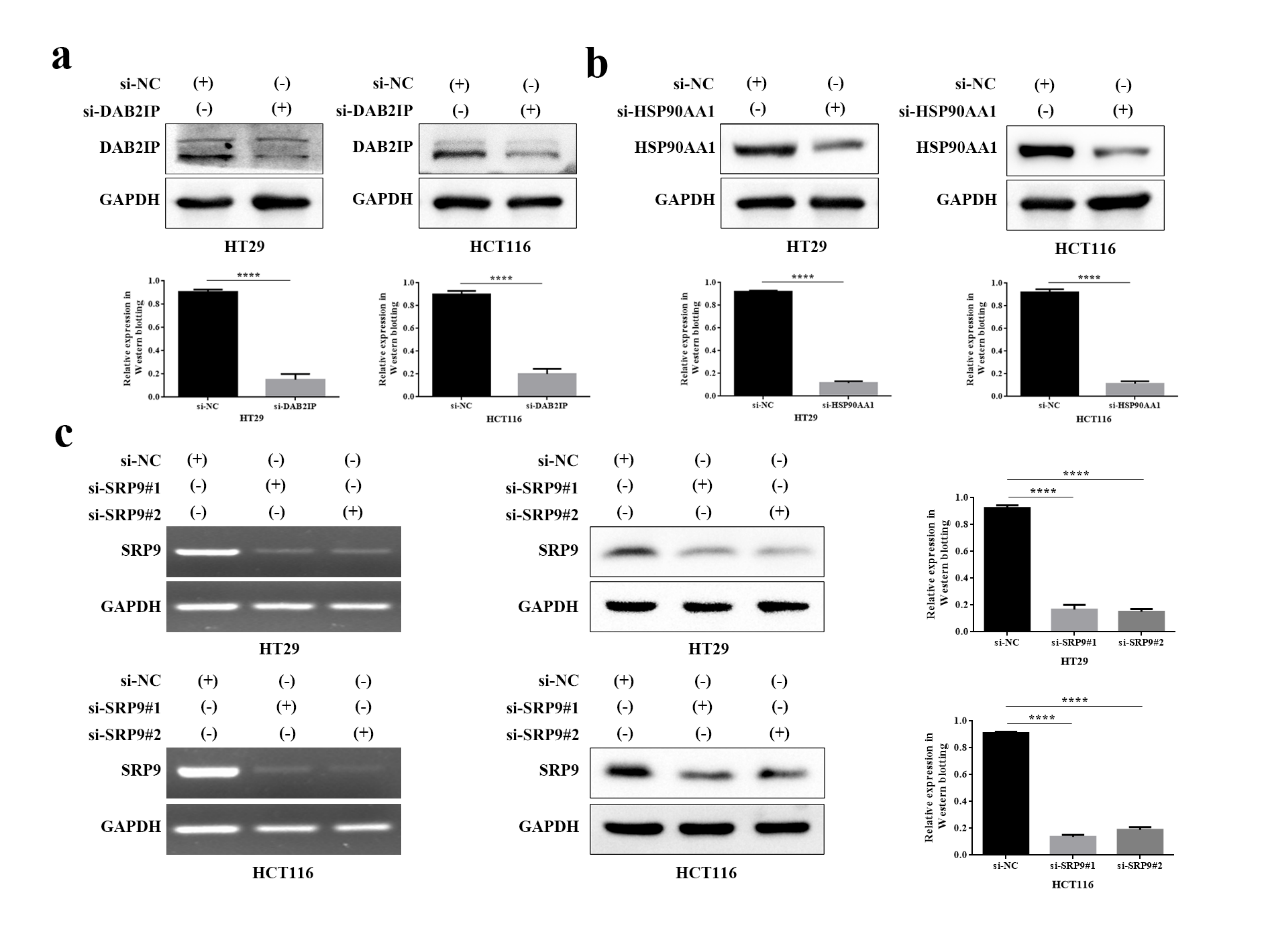


**Figure S5. Inhibition rate of DAB2IP, HSP90AA1 or SRP9 knockdown by corresponding siRNA in HT29 and HCT116 cells.** The gray intensity of protein expression in HT29 and HCT116 cells were quantified by image J software. Data was presented as mean ± SD, n = 3; **P<0.05, **P<0.01, ***P<0.001, ****P<0.0001.*


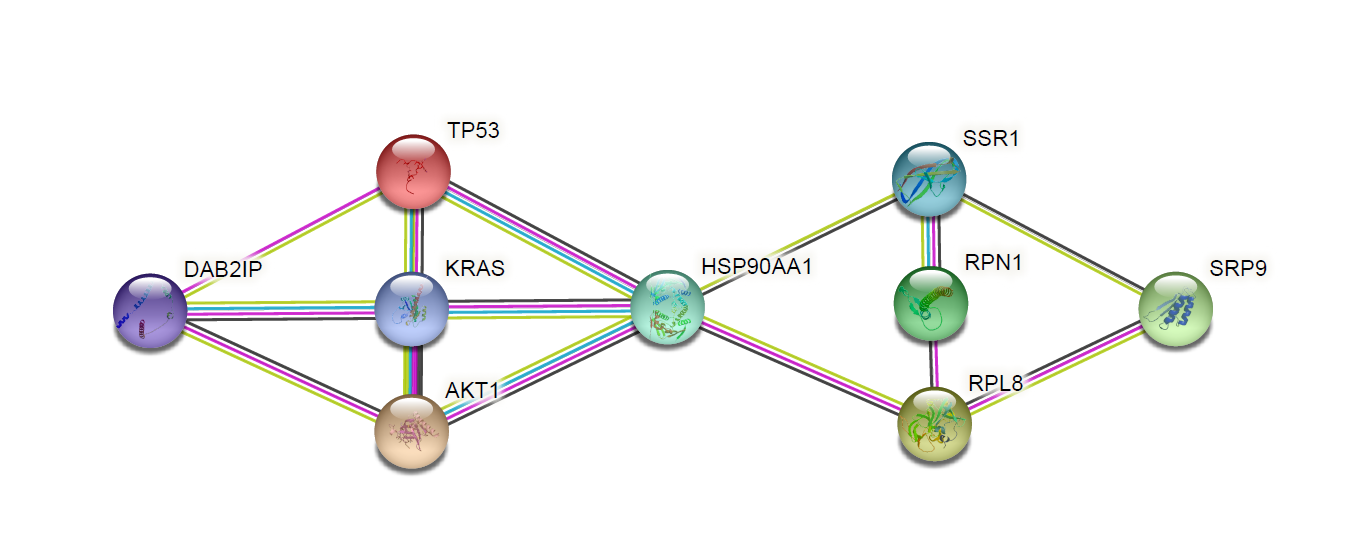


**Figure S6. The interaction between DAB2IP and HSP90AA1, SRP9, RPN1, SSR1, RPL84 in the String database.**

**
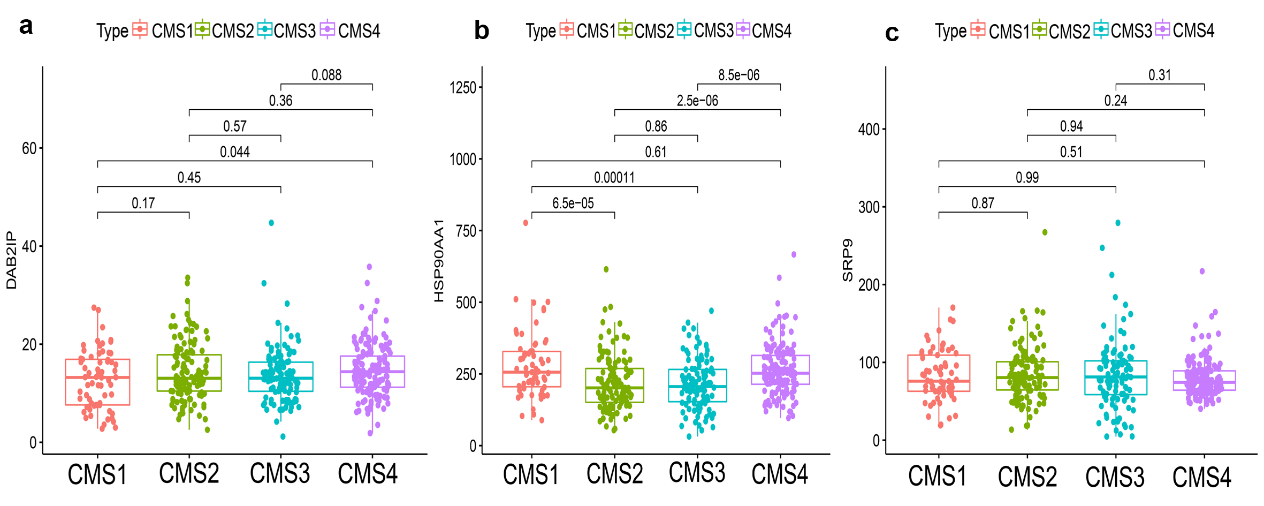
**

**Figure S7. The expression of DAB2IP, HSP90AA1 and SRP9 among different subtypes of CMS classification in colon cancer.**

**Table S1. information of CRC patient samples.**

| **Number** | **Sex** | **Age** | **Cancer type** | **Pathologic tumor stage** |
| --- | --- | --- | --- | --- |
| T1/N1 | Male | 70 | Colon adenocarcinoma | ⅡA（T3，No，cMo） |
| T2/N2 | Female | 47 | Colon adenocarcinoma | ⅢC（T4a，N1b，cMo） |
| T3/N3 | Male | 61 | Colon adenocarcinoma | ⅢB（T3，N1，cM0） |
| T4/N4 | Female | 75 | Colon adenocarcinoma | ⅡA（T3，No，cMo） |
| T5/N5 | Male | 49 | Colon adenocarcinoma | ⅡA（T3，No，cMo） |
| T7/N7 | Female | 62 | Colon adenocarcinoma | ⅡA（T3，No，cMo） |

**T****a****b****le S2. Sequences of siRNA**

| siRNA | Sequences |
| --- | --- |
| hsa-DAB2IP | GGTGAAGGACTTCCTGACA |
| hsa-HSP90AA1 | CTACAATTCCTCTGATAAT |
| hsa-SRP9 | siRNA#1: CACGTGTGGTTCTCAAATA  siRNA#2: CCGCAATGTTACCATGGAA |

**Table S3.** **Antibody information.**

| Item | number | source | concentration |
| --- | --- | --- | --- |
| DAB2IP | 23582-1-AP | Proteintech, America | 1:1000 |
| HSP90AA1 | 13171-1-AP | Proteintech, America | 1:2000 |
| SRP9 | sc-514722 | Santa Cruz Biotechnology, America | 1:500 |
| p-ASK1 | #3765 | CST, America | 1:1000 |
| t-ASK1 | #8662 | CST, America | 1:1000 |
| p-JNK | #4668 | CST, America | 1:1000 |
| t-JNK | #9258 | CST, America | 1:1000 |
| BAX | #5023 | CST, America | 1:1000 |
| BCL-2 | 12789-1-AP | Proteintech, America | 1:2000 |

**T****a****b****le S4.** **Sequences of primers for PCR.**

| Gene | Forward Primer (5’ to 3’) | Reverse Primer (5’ to 3’) |
| --- | --- | --- |
| DAB2IP | TATAAGGCAGGCGCGGGGGT | CGCTGCATGTTGGTCCACTCAT |
| HSP90AA1 | CGTCTTCGGAAACATGGCTT | CGGTTTGACACAACCACCTT |
| SSR1 | TATGTTCCTTGCTGGTCT | TATGTTCCTTGCTGGTCT |
| SRP9 | GGTTCTGAGGCCTTGCTTCT | CACACGTGCCTTCATAGGGT |
| RPL8 | TGACGCCGTGTTTCCTCTTT | TGATGTCCTTGACGATGCCC |
| RPN1 | ACCGTCACTTTGACGAGACC | ATTTCGCTCACTCTGTCGCA |
| GAPDH | GAAGGTGAAGGTCG GAGTC | GAAGATGGTGA TGGGATTTC |

| **Table S5. The correlation between HSP90AA1 and clinical features in CRC.** | | | | |
| --- | --- | --- | --- | --- |
| Characteristic | Low | High | p |  |
| n | 271 | 271 |  |  |
| Gender, n (%) |  |  | 0.605 |  |
| female | 123 (22.7%) | 130 (24%) |  |  |
| male | 148 (27.3%) | 141 (26%) |  |  |
| Methylation subtype, n (%) |  |  | < 0.001 |  |
| CIMP-H | 27 (5.4%) | 63 (12.5%) |  |  |
| CIMP-L | 57 (11.3%) | 56 (11.1%) |  |  |
| Cluster3 | 103 (20.4%) | 44 (8.7%) |  |  |
| Cluster4 | 62 (12.3%) | 92 (18.3%) |  |  |
| MSI status, n (%) |  |  | < 0.001 |  |
| Indeterminate | 14 (2.7%) | 6 (1.1%) |  |  |
| MSI-H | 22 (4.2%) | 59 (11.2%) |  |  |
| MSI-L | 40 (7.6%) | 42 (8%) |  |  |
| MSS | 187 (35.6%) | 156 (29.7%) |  |  |
| Pathologic stage, n (%) |  |  | 0.059 |  |
| stage Ⅰ | 48 (9.1%) | 45 (8.5%) |  |  |
| stage Ⅱ | 91 (17.3%) | 118 (22.4%) |  |  |
| stage Ⅲ | 85 (16.1%) | 63 (12%) |  |  |
| stage Ⅳ | 42 (8%) | 35 (6.6%) |  |  |
| M stage, n (%) |  |  | 0.613 |  |
| M0 | 201 (42.1%) | 201 (42.1%) |  |  |
| M1 | 41 (8.6%) | 35 (7.3%) |  |  |
| N stage, n (%) |  |  | 0.059 |  |
| N0 | 146 (27%) | 172 (31.9%) |  |  |
| N1 | 73 (13.5%) | 56 (10.4%) |  |  |
| N2 | 52 (9.6%) | 41 (7.6%) |  |  |
| T stage, n (%) |  |  | 0.860 |  |
| T1 | 9 (1.7%) | 6 (1.1%) |  |  |
| T2 | 46 (8.5%) | 47 (8.7%) |  |  |
| T3 | 185 (34.3%) | 184 (34.1%) |  |  |
| T4 | 30 (5.6%) | 33 (6.1%) |  |  |
| Race, n (%) |  |  | < 0.001 |  |
| american indian or alaska native | 0 (0%) | 1 (0.3%) |  |  |
| asian | 3 (0.9%) | 9 (2.8%) |  |  |
| black or african american | 39 (12.1%) | 20 (6.2%) |  |  |
| white | 89 (27.6%) | 161 (50%) |  |  |
| location, n (%) |  |  | 0.106 |  |
| colon | 215 (39.9%) | 231 (42.9%) |  |  |
| rectum | 54 (10%) | 39 (7.2%) |  |  |
| Age, n (%) |  |  | 0.598 |  |
| <65 | 104 (19.3%) | 111 (20.6%) |  |  |
| >=65 | 166 (30.7%) | 159 (29.4%) |  |  |
| HSP90AA1, meidan (IQR) | 169.82 (131.43, 199.61) | 292.57 (252.25, 344.33) | < 0.001 |  |

STR profiling of cell lines


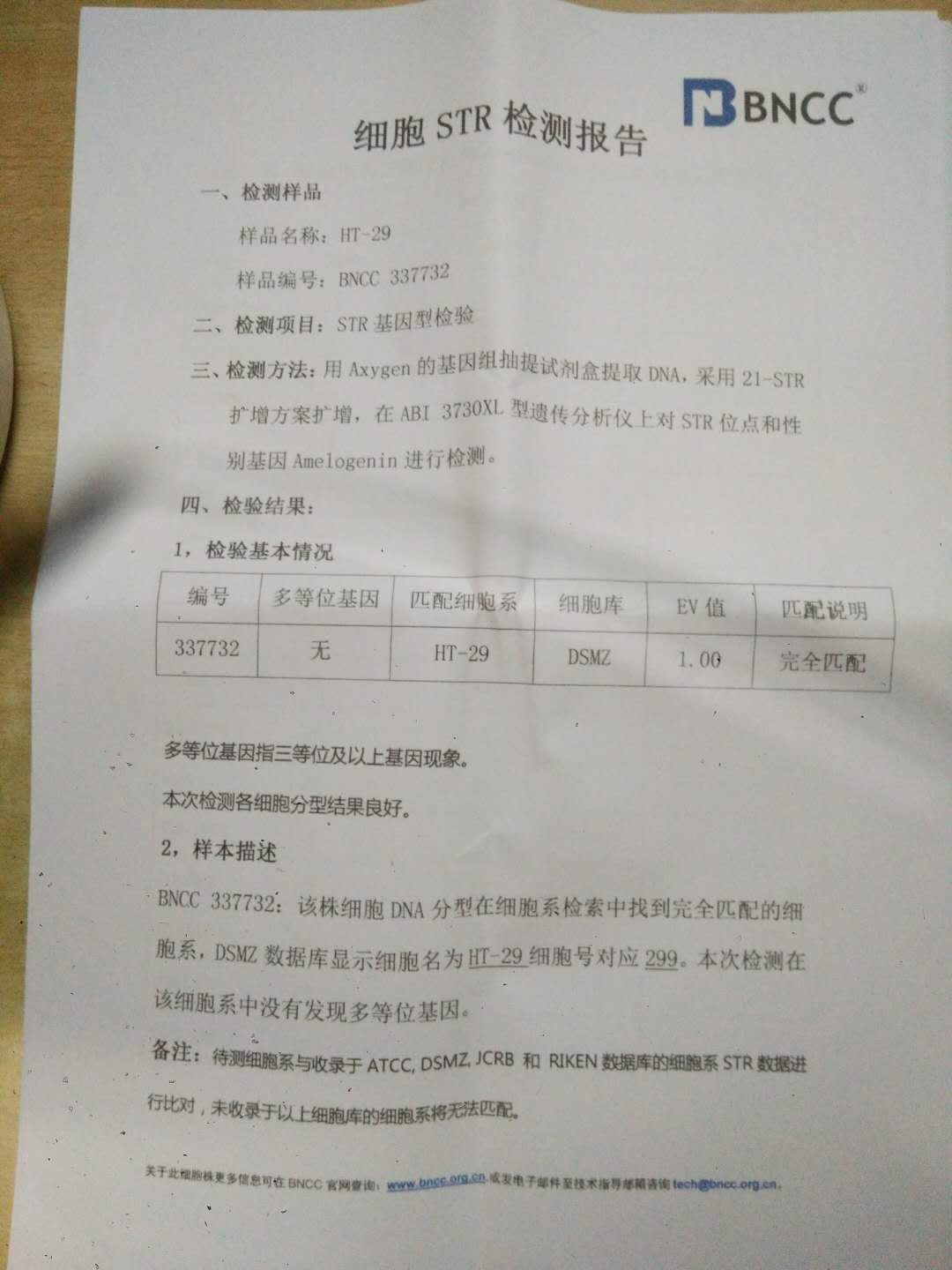


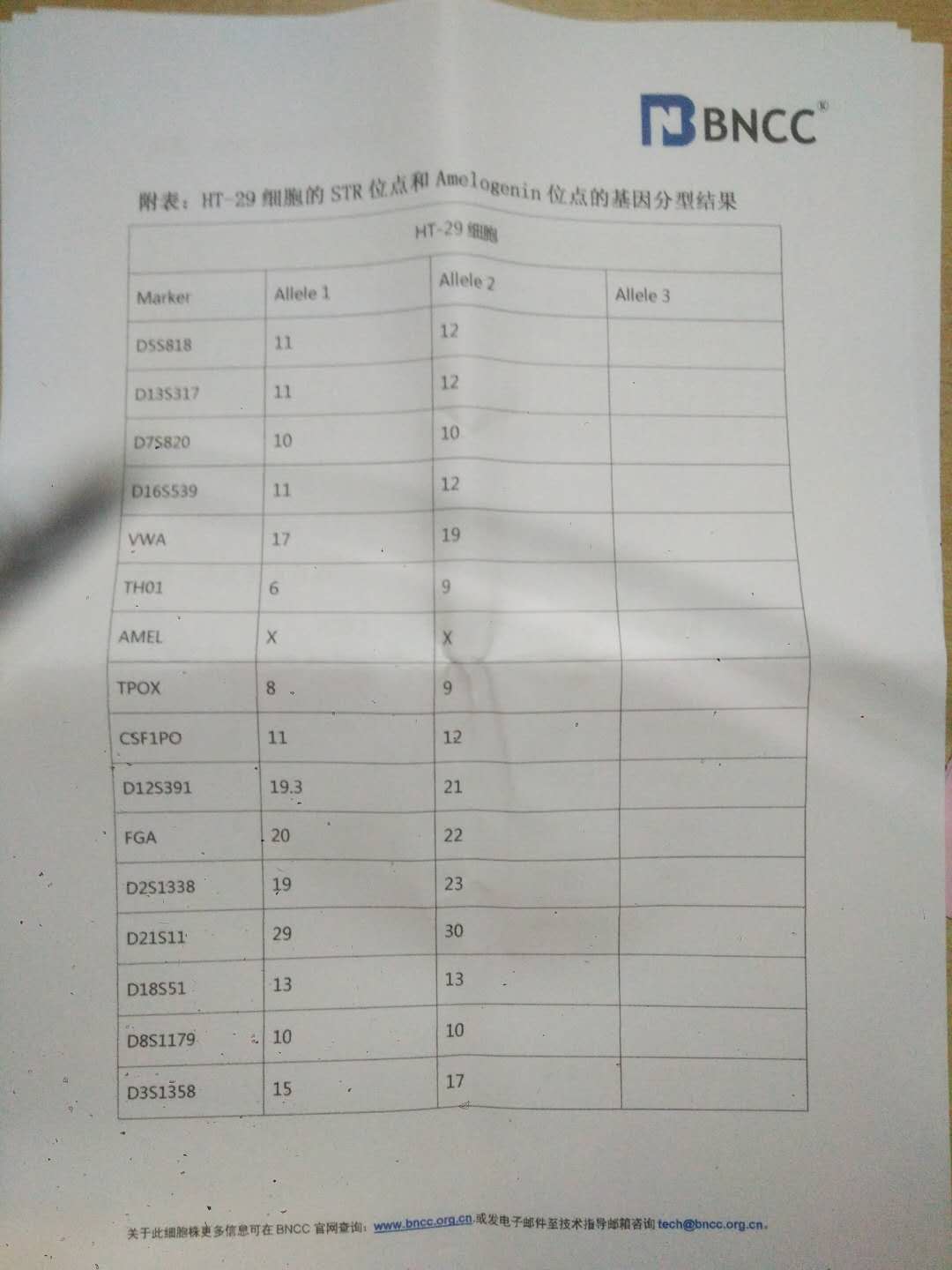


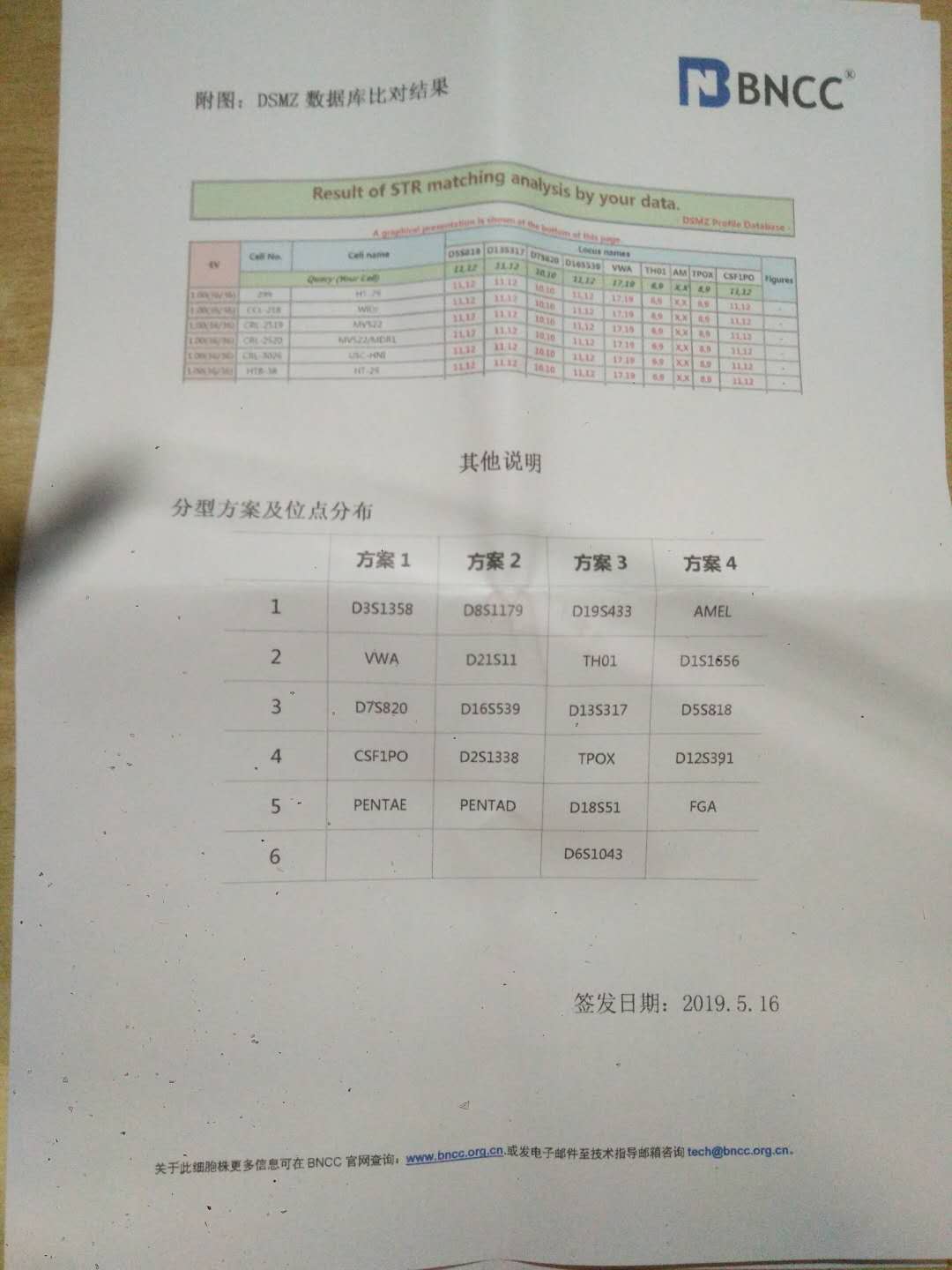


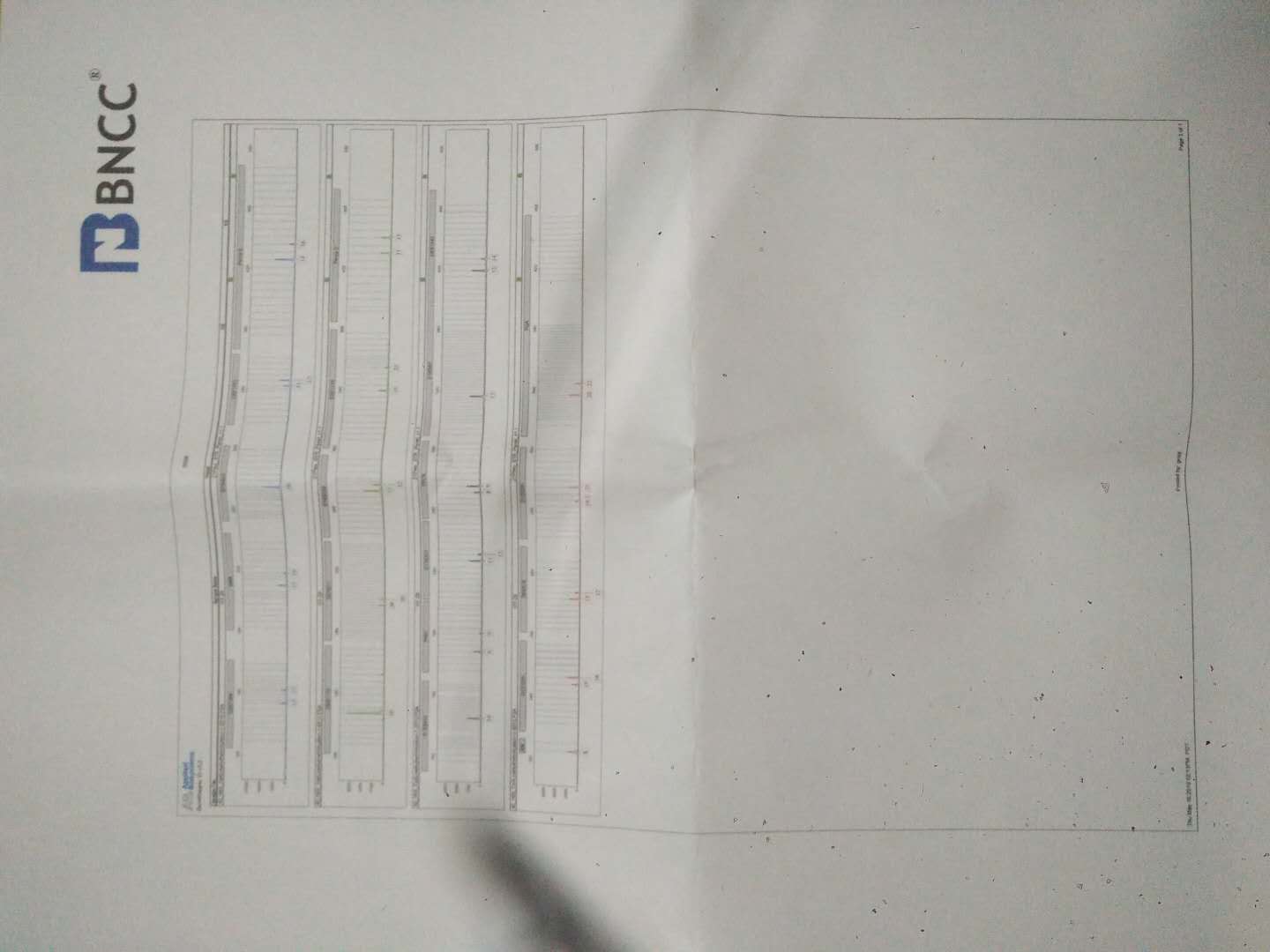

Supplement: Supplementary file 1 — Additional file 1. [file 12885_2022_9596_MOESM1_ESM.docx]
