# Supplementary material for: DAB2IP down-regulates HSP90AA1 to inhibit the malignant biological behaviors of colorectal cancer
Source: BMC Cancer. 2022 May 19;22:561. doi: 10.1186/s12885-022-09596-z (PMC9118737; doi:10.1186/s12885-022-09596-z)
Supplement: Supplementary file 2 — Additional file 2. [file 12885_2022_9596_MOESM2_ESM.pdf]

Since our original exposure was very bright, there was no way to change it, and we could not see the film by adjusting the contrast, so we provided three strips for parallel experiments.

A detailed description of full unedited blots (Supplementary page)

Page 3. Supplementary full unedited blots of other two parallel experiments for Fig.1i high contrast blots HSP90AA1 and GAPDH

Page 5&6. Supplementary full unedited blots of other two parallel experiments for Fig.1l high contrast blots HSP90AA1(HT29 and HCT116) and GAPDH (HT29 and HCT116)

Page 8. Supplementary full unedited blots of other two parallel experiments for Fig.1n high contrast blots HSP90AA1(HT29)

Page 10. Supplementary full unedited blots of other two parallel experiments for Fig.3i high contrast blots GAPDH

Page 16&17. Supplementary full unedited blots of other two parallel experiments for Fig.5c high contrast blots BAX (HT29 and HCT116), BCL-2(HT29) and GAPDH (HT29 and HCT116)

Page 22&23. Supplementary full unedited blots of other two parallel experiments for Fig.5d high contrast blots T-JNK (HT29 and HCT116) and GAPDH (HT29 and HCT116)

Page 28&29. Supplementary full unedited blots of other two parallel experiments for Fig.5e high contrast blots BAX (HT29 and HCT116) and HSP90AA1 (HT29 and HCT116)

Page 34&35&36. Supplementary full unedited blots of other two parallel experiments for Fig.5f high contrast blots p-JNK (HT29 and HCT116), BAX (HT29 and HCT116) and GAPDH (HT29 and HCT116)

Page 41&42&43. Supplementary full unedited blots of other two parallel experiments for Fig. 5g (si#1) high contrast blots SRP9(HT29 and HCT116), BAX (HT29 and HCT116) and GAPDH (HT29 and HCT116)

Page 48. Supplementary full unedited blots of other two parallel experiments for Fig. 5g (si#2) high contrast blots p-ASK1(HCT116) and GAPDH (HT29 and HCT116)

Page 53&54. Supplementary full unedited blots of other two parallel experiments for Fig.5h high contrast blots T-JNK (HT29 and HCT116) and GAPDH (HT29 and HCT116)

Page 58. Supplementary full unedited blots of other two parallel experiments for Fig. S2 high contrast blots HSP90AA1 and GAPDH

Page 62. Supplementary full unedited blots of other two parallel experiments for Fig. S2 high contrast blots HSP90AA1 (HT29 and HCT116) and SRP9 (HCT116)

A. Full unedited blots for Fig.1i

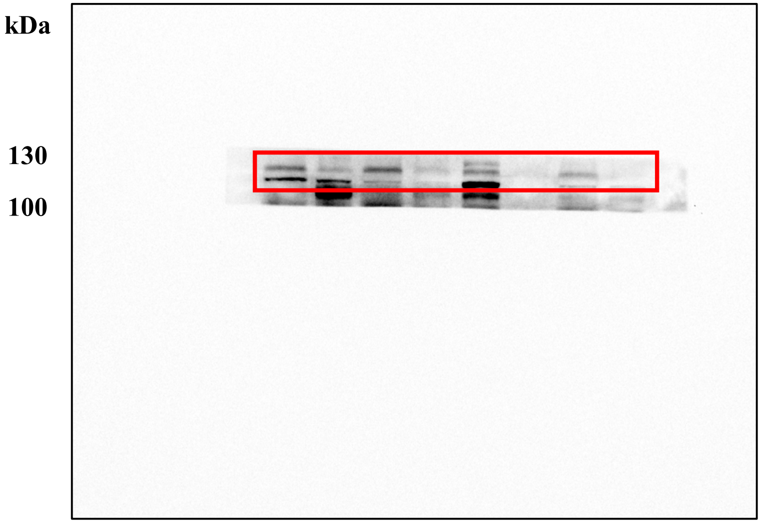

Anti-DAB2IP

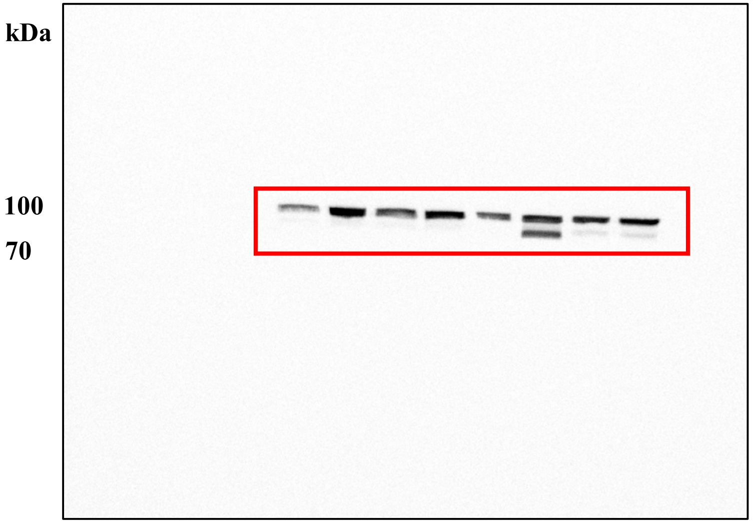

Anti-HSP90AA1

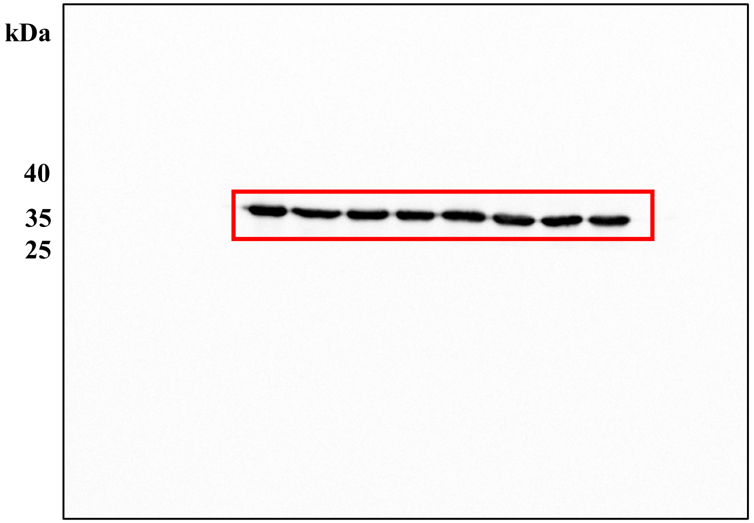

Anti-GAPDH

Sup A. Full unedited blots for Fig.1i

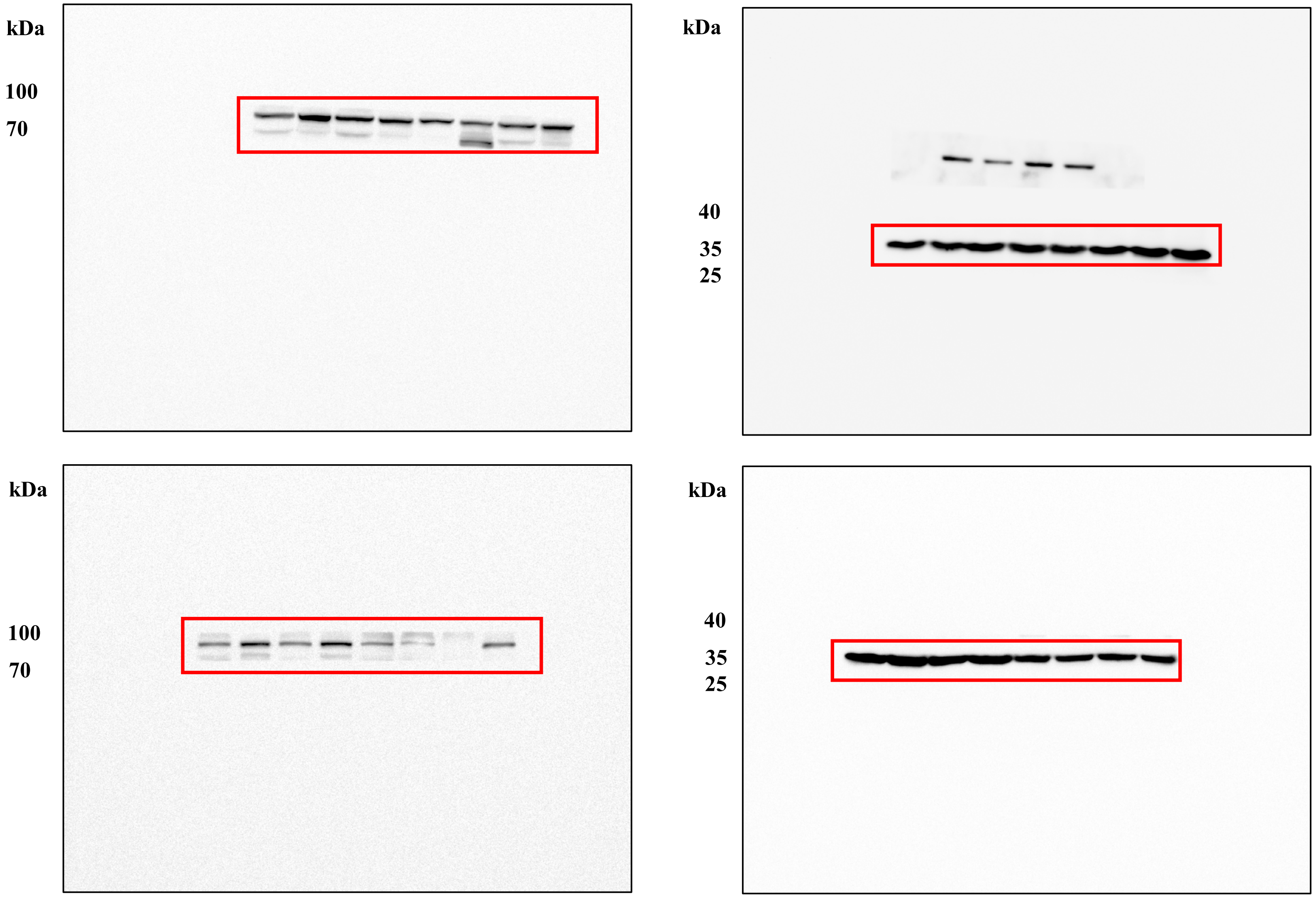

Anti-HSP90AA1

Anti-GAPDH

B. Full unedited blots for Fig.1l

HT29

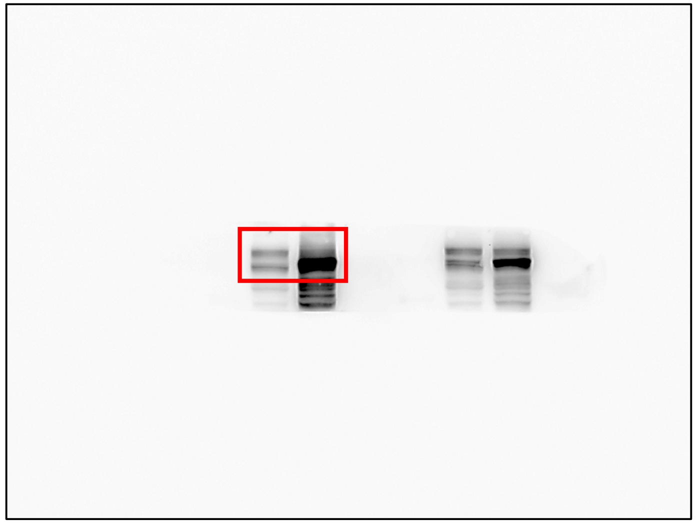

Anti-DAB2IP

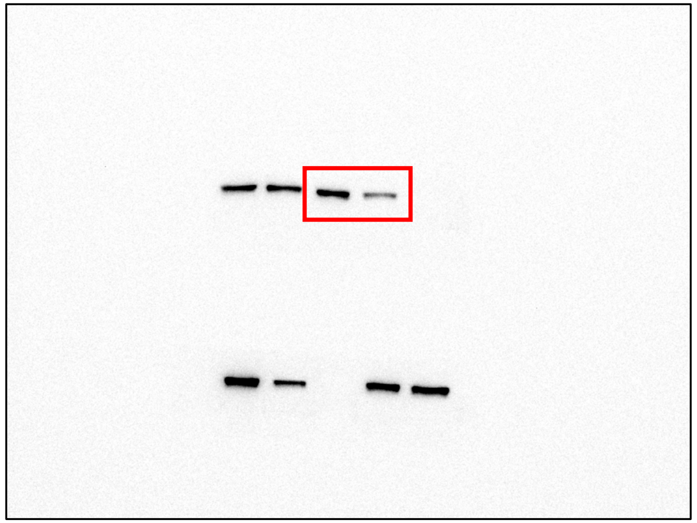

Anti-HSP90AA1

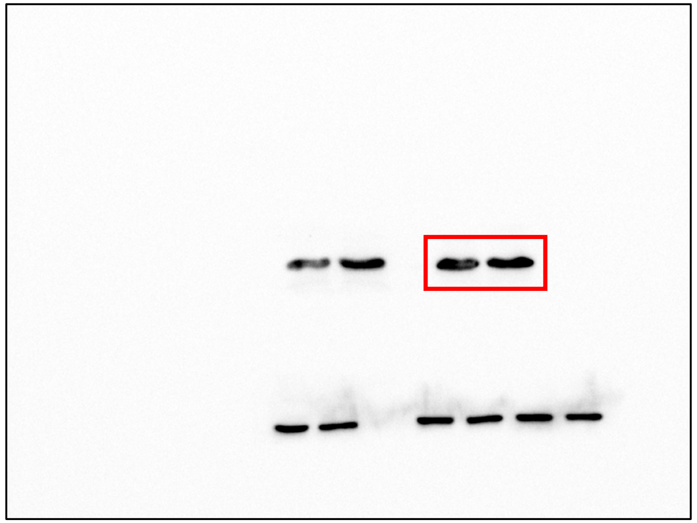

Anti-GAPDH

HCT116

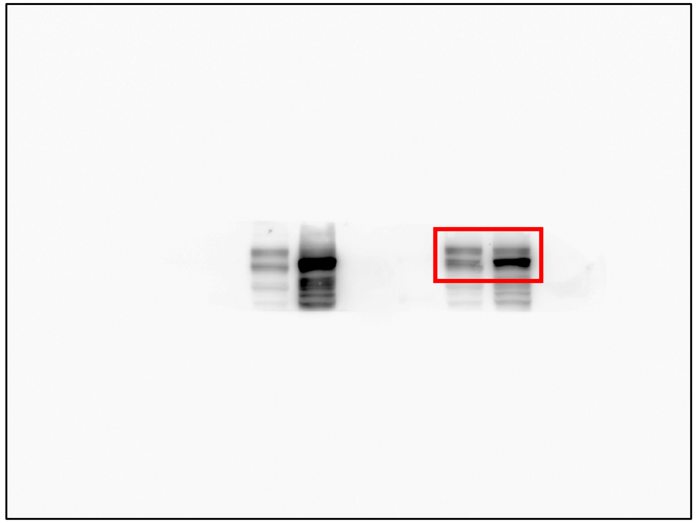

Anti-DAB2IP

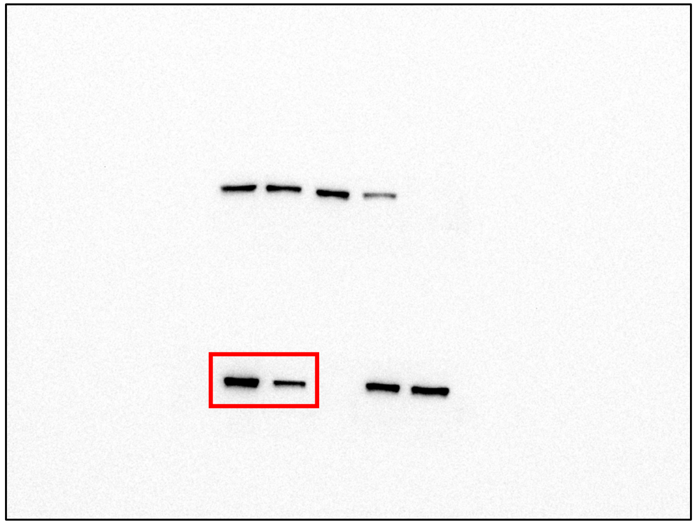

Anti-HSP90AA1

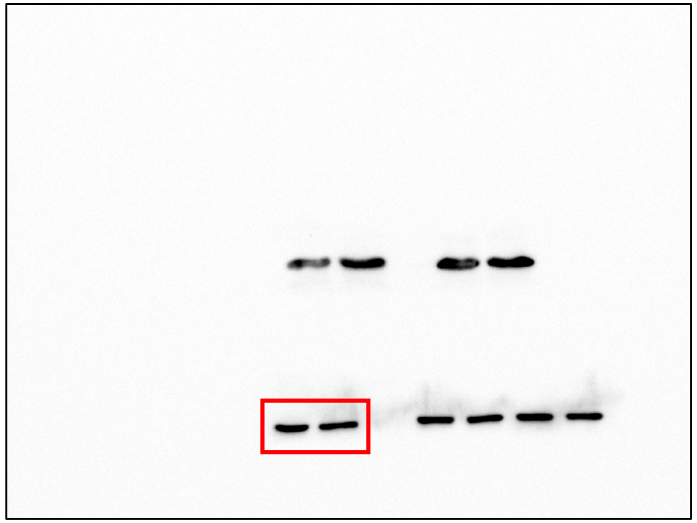

Anti-GAPDH

Sup B. Full unedited blots for Fig.1l

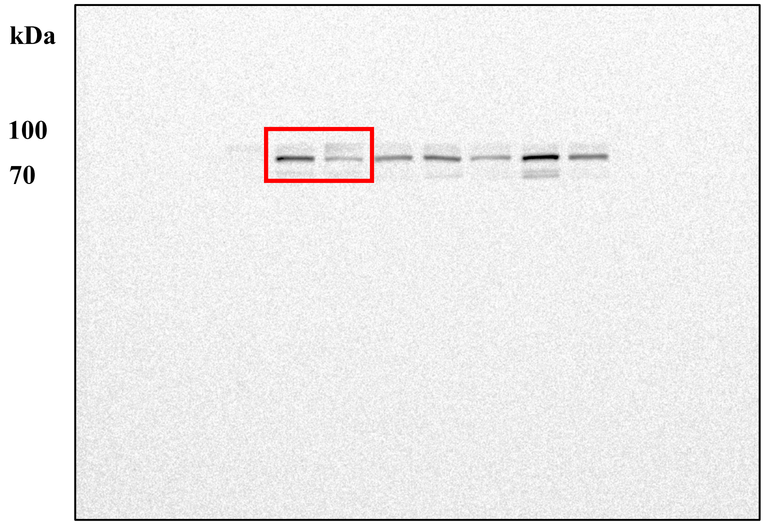

HT29 Anti-HSP90AA1

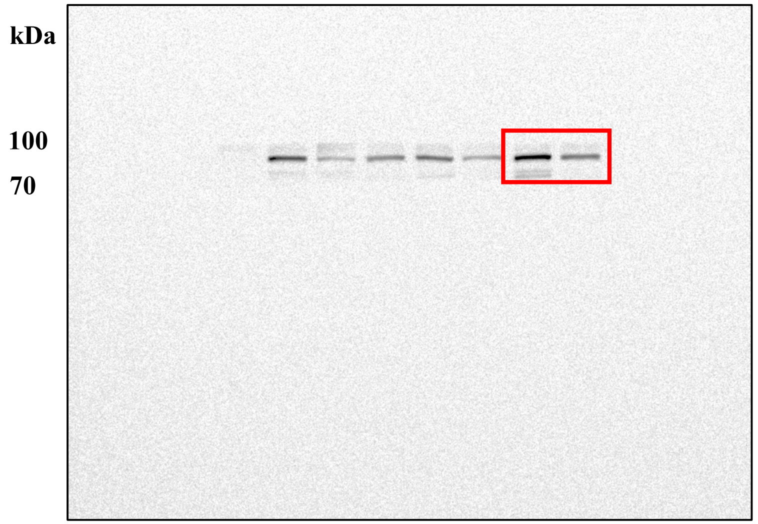

HCT116 Anti-HSP90AA1

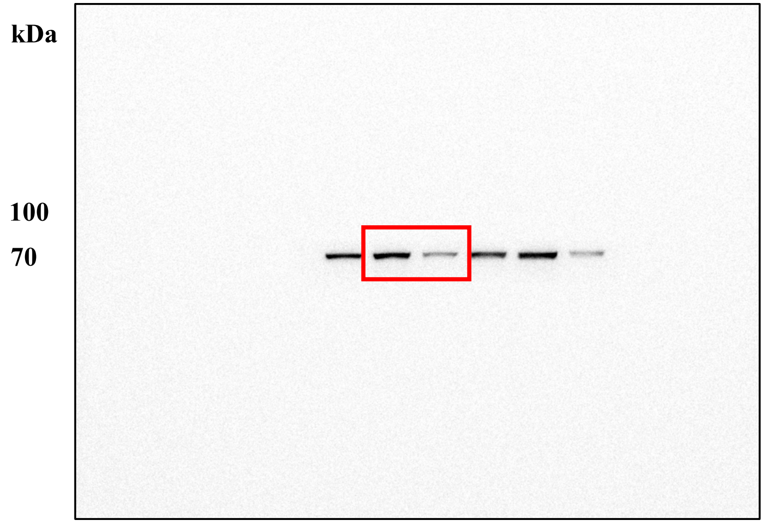

HT29 Anti-HSP90AA1

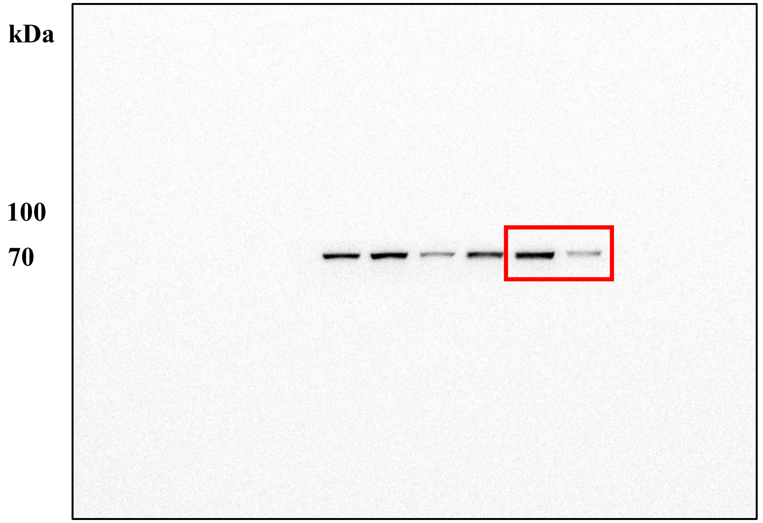

HCT116 Anti-HSP90AA1

Sup B. Full unedited blots for Fig.1l

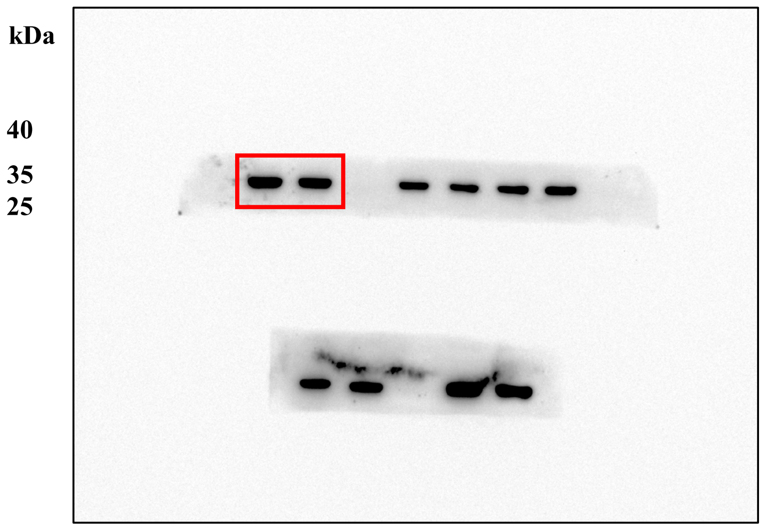

HT29 Anti-GAPDH

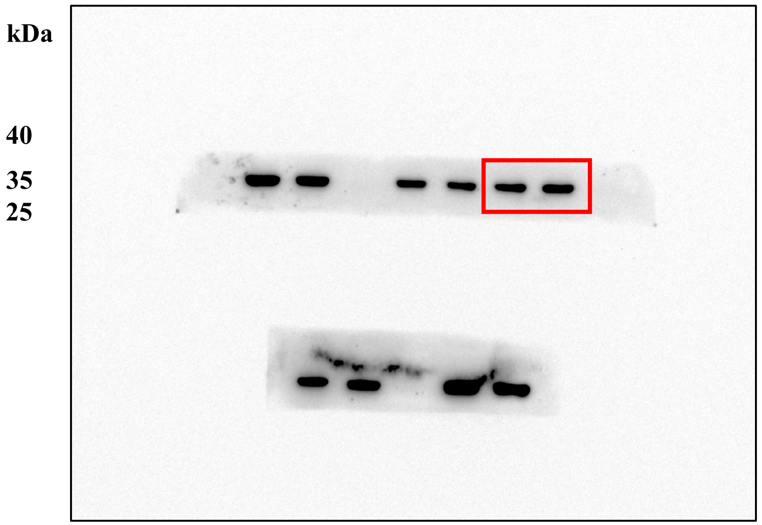

HCT116 Anti-GAPDH

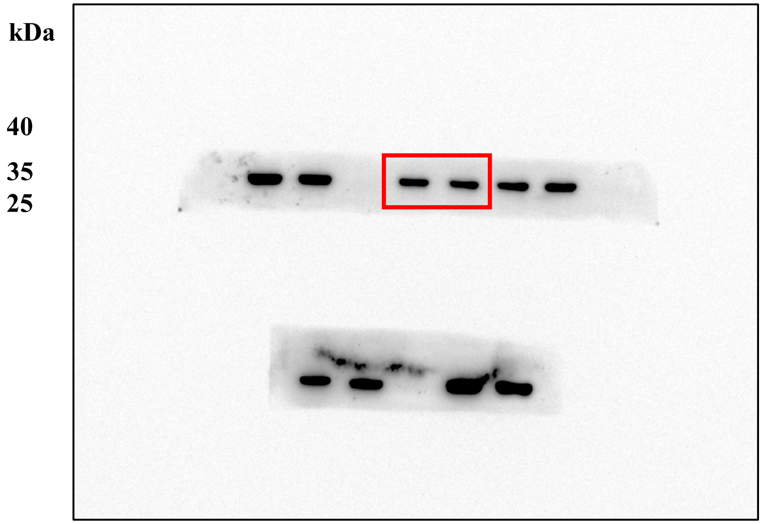

HT29 Anti-GAPDH

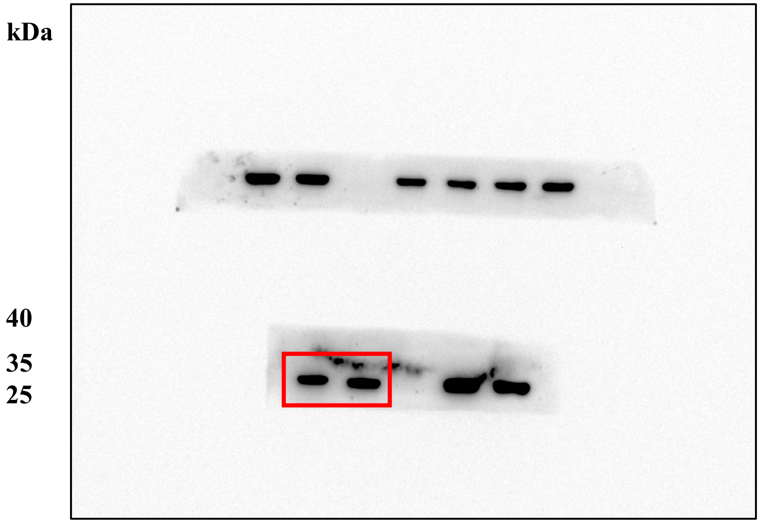

HCT116 Anti-GAPDH

C. Full unedited blots for Fig.1n

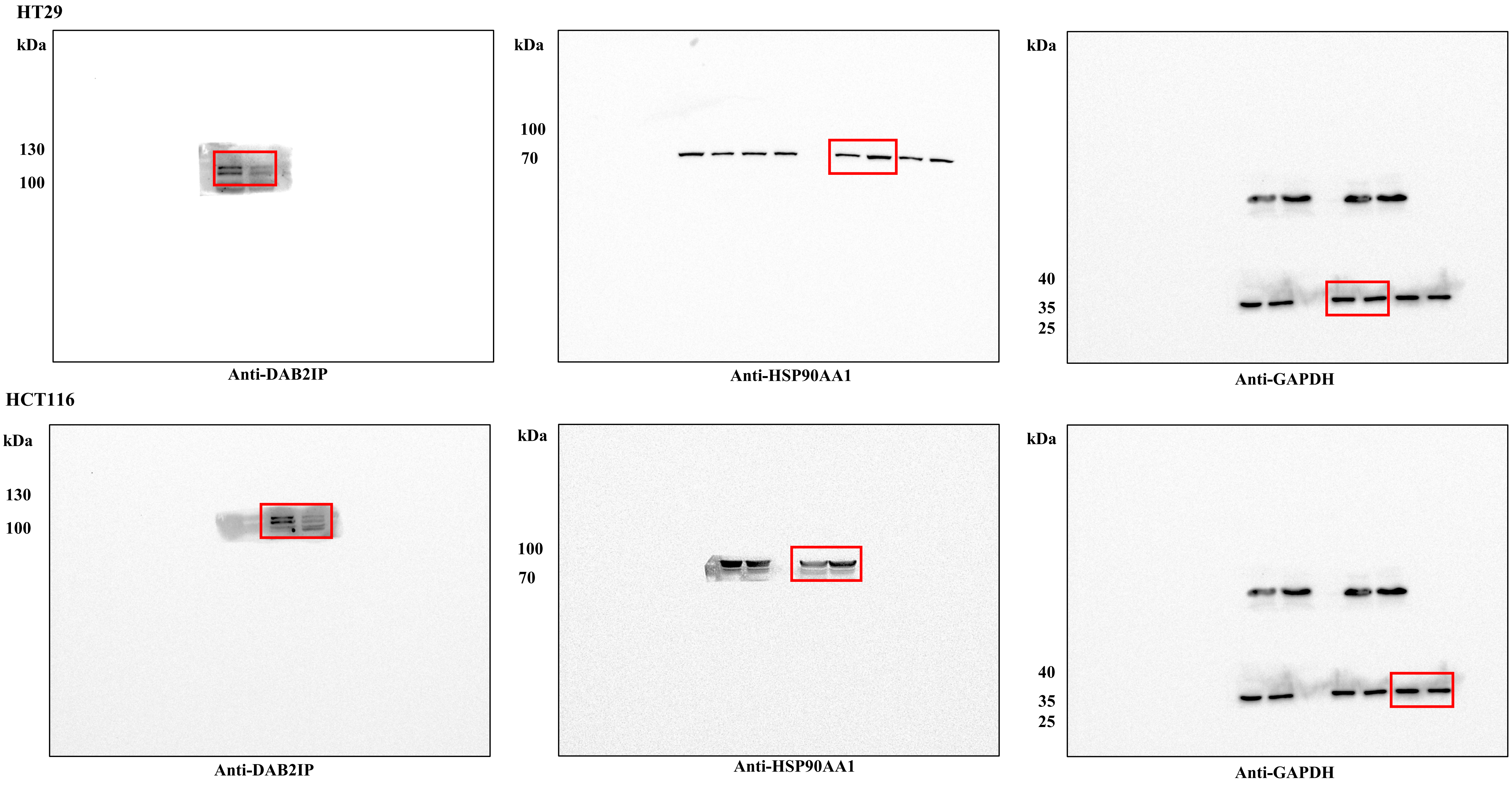

Sup C. Full unedited blots for Fig.1n

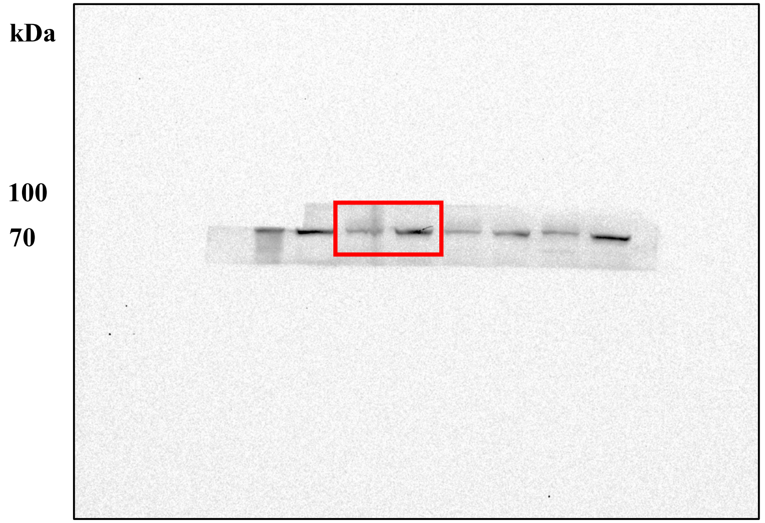

HT29 Anti-HSP90AA1

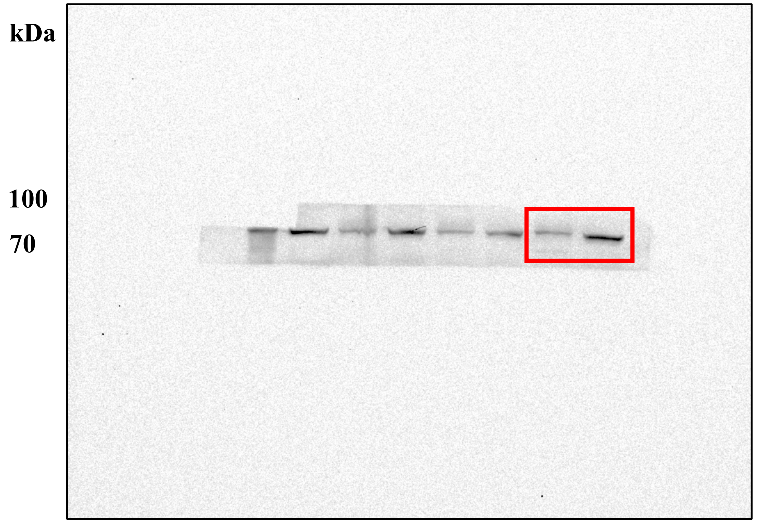

HT29 Anti-HSP90AA1

D. Full unedited blots for Fig.3i

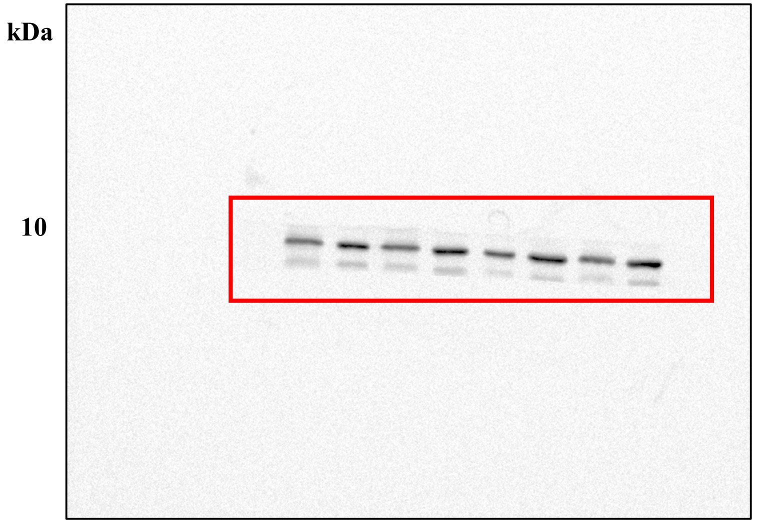

Anti-SRP9

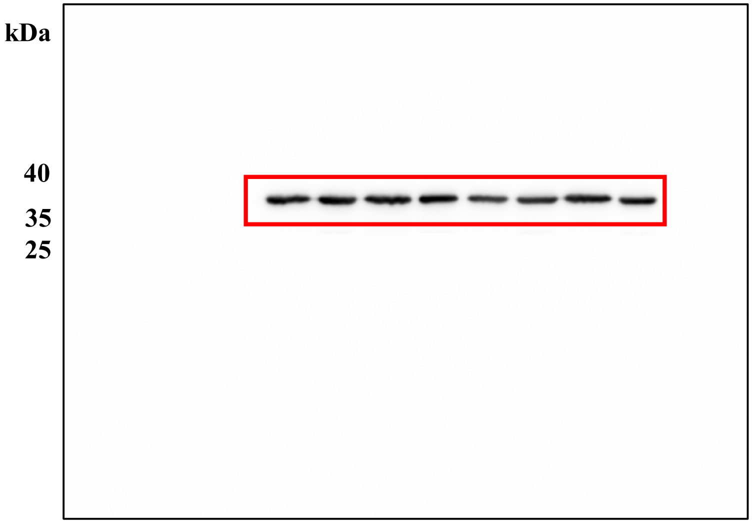

Anti-GAPDH

Sup D. Full unedited blots for Fig.3i

kDa
40
35
25

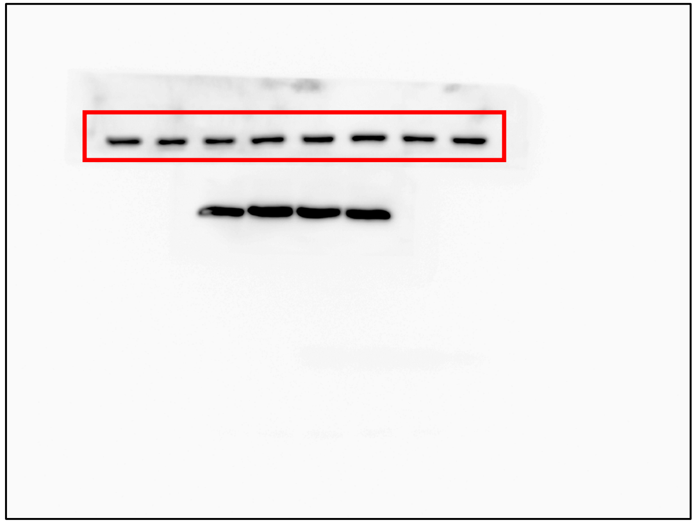

Anti-GAPDH

kDa
40
35
25

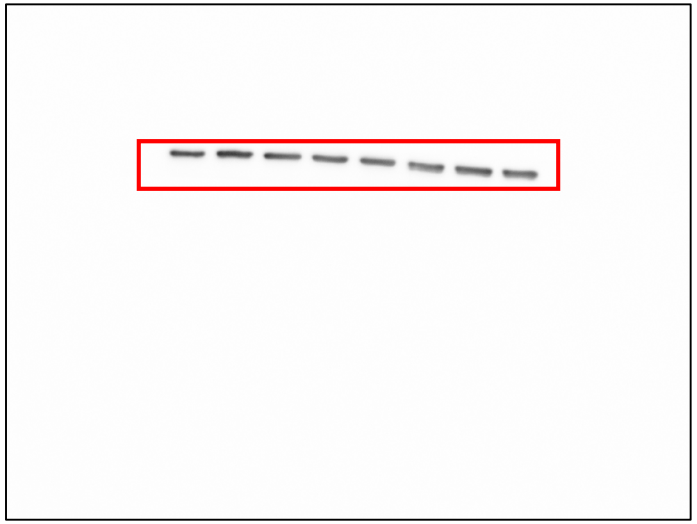

Anti-GAPDH

E. Full unedited blots for Fig.4b

HT29

kDa
100
70

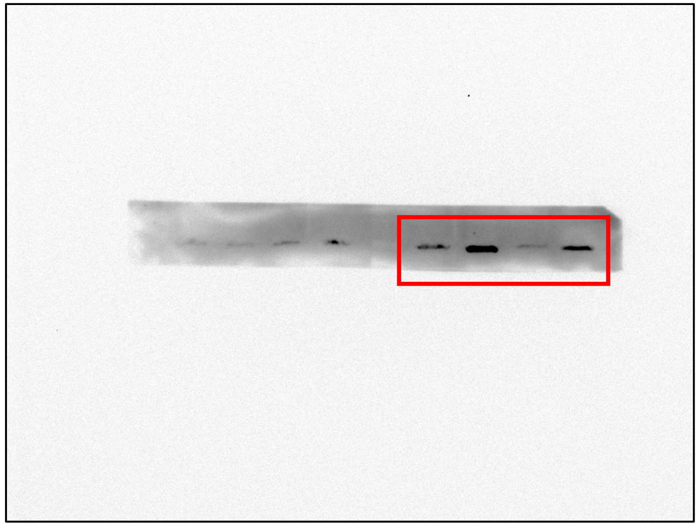

Anti-HSP90AA1

kDa
10

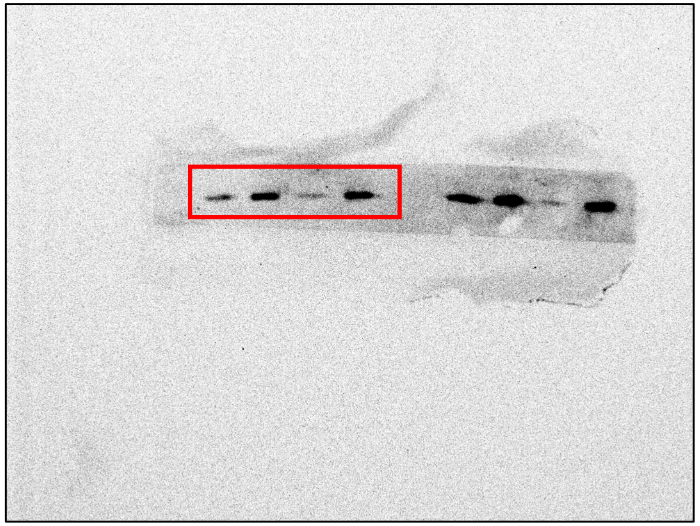

Anti-SRP9

kDa
40
35
25

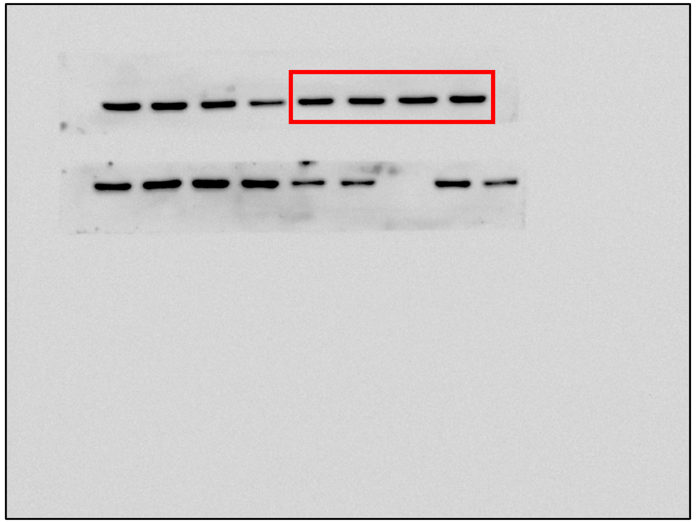

Anti-GAPDH

HCT116

kDa
100
70

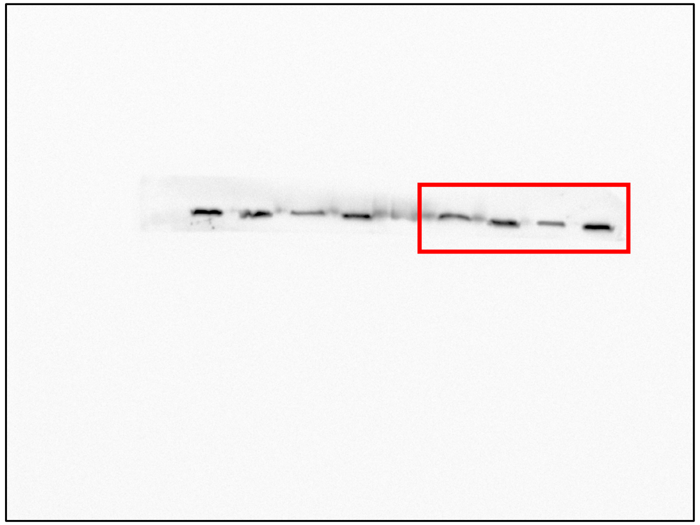

Anti-HSP90AA1

kDa
10

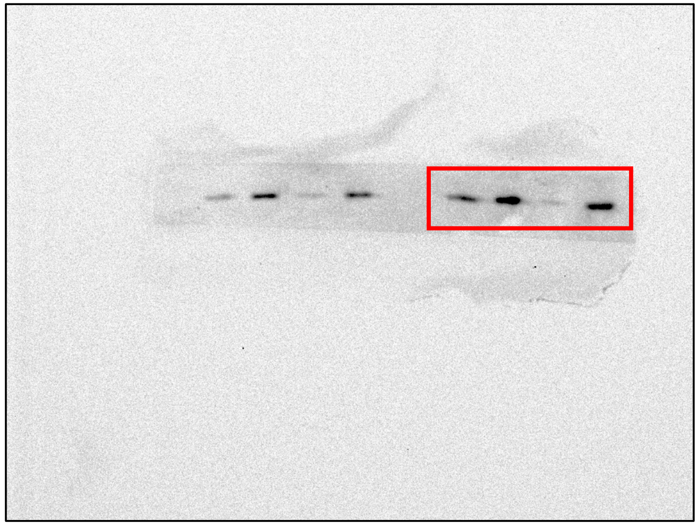

Anti-SRP9

kDa
40
35
25

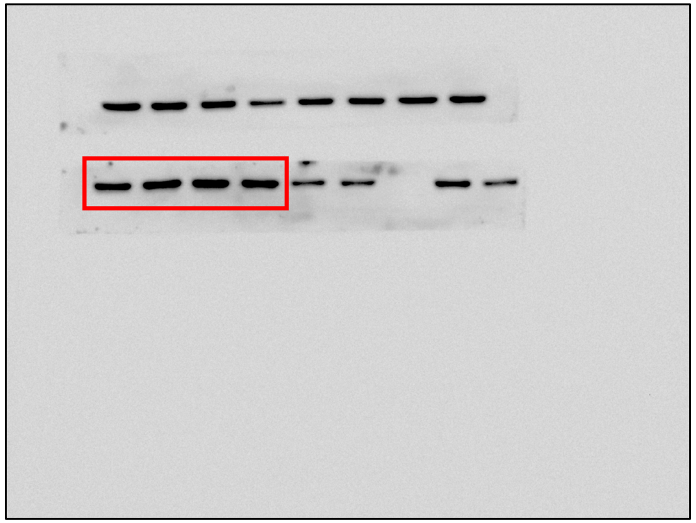

Anti-GAPDH

F. Full unedited blots for Fig. 5c HT29

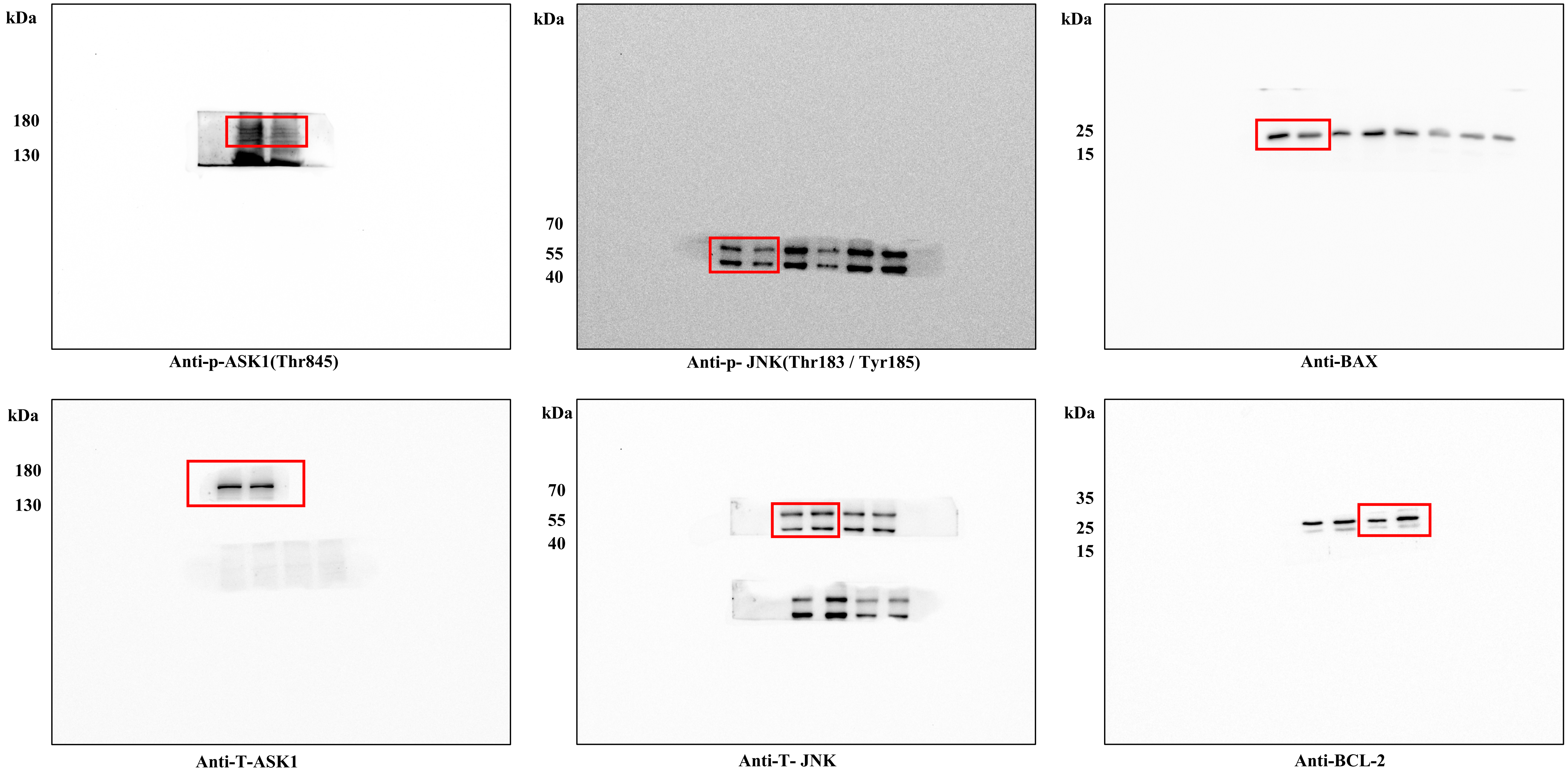

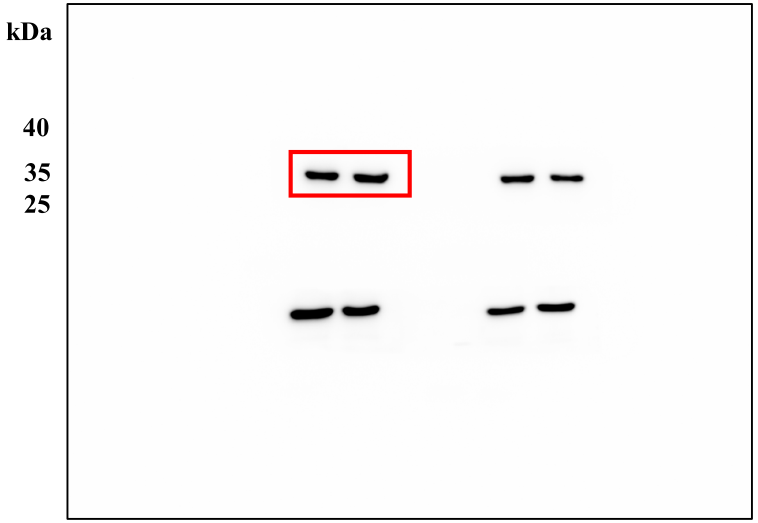

Anti-GAPDH

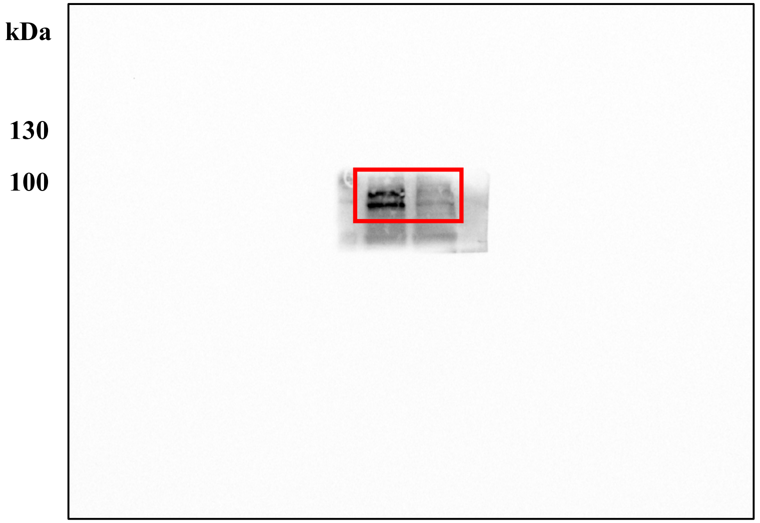

Anti-DAB2IP

F. Full unedited blots for Fig.5c HCT116

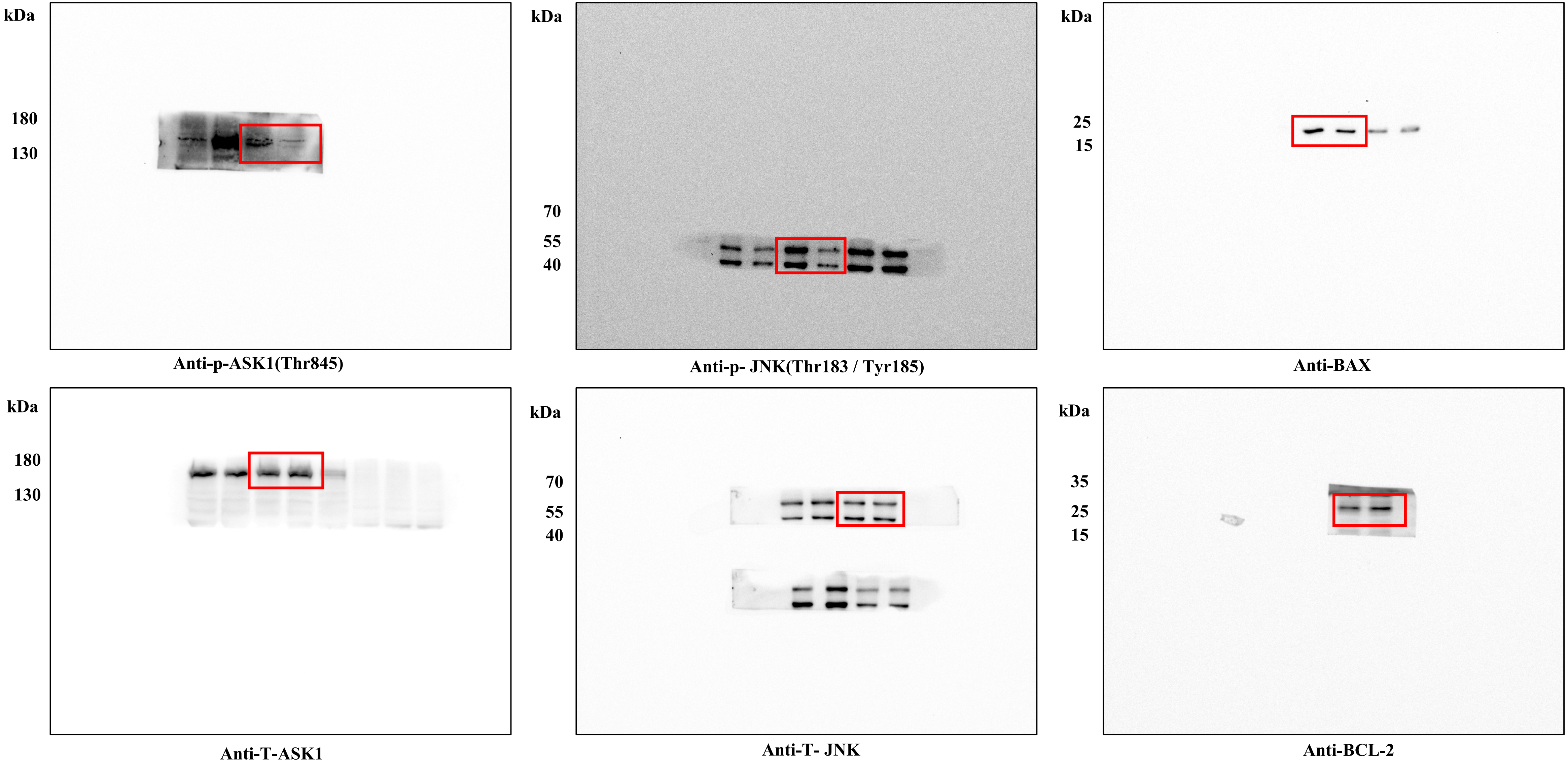

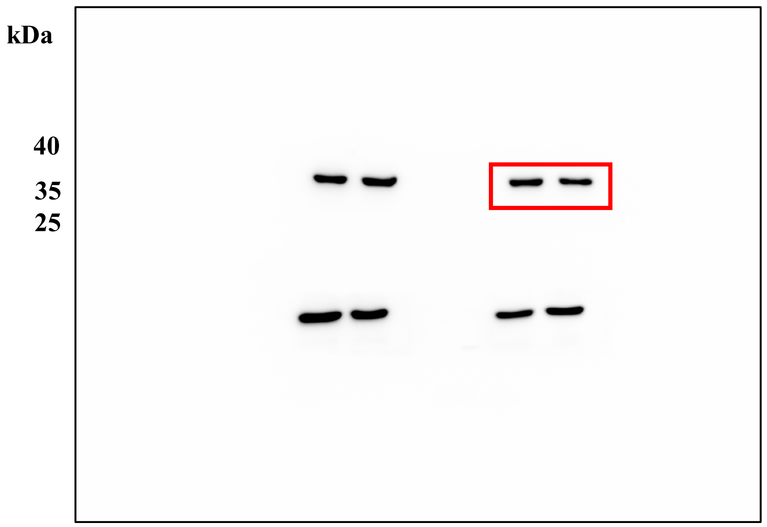

Anti-GAPDH

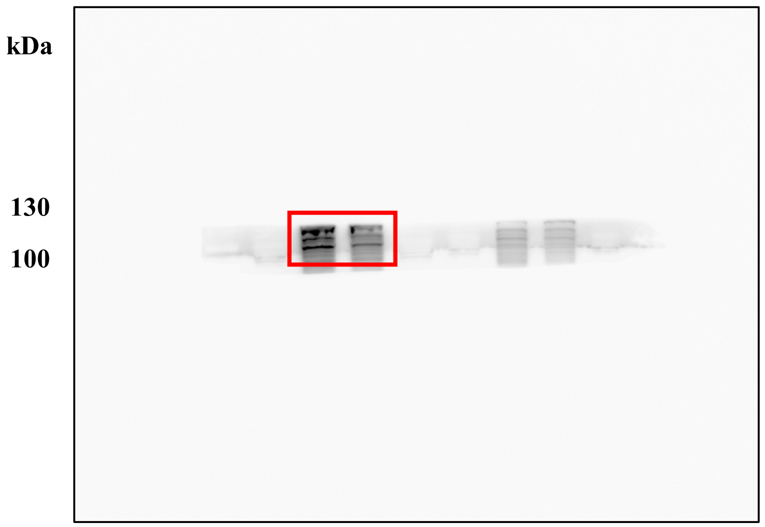

Anti-DAB2IP

Sup F. Full unedited blots for Fig.5c

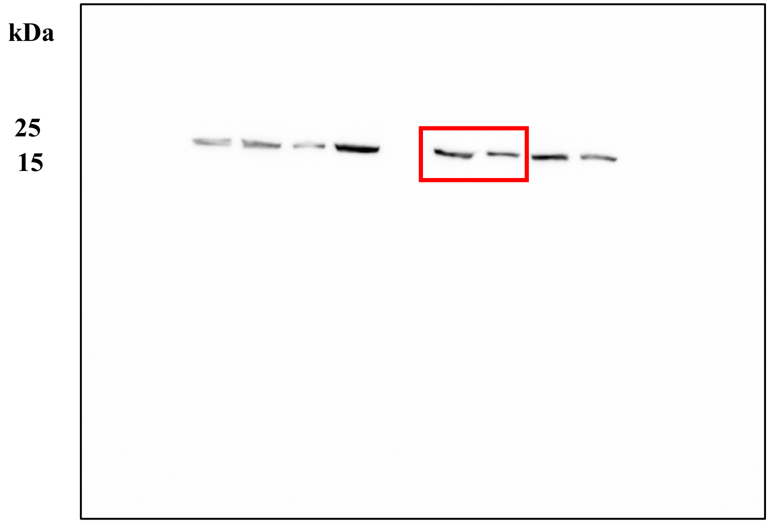

HT29 Anti-BAX

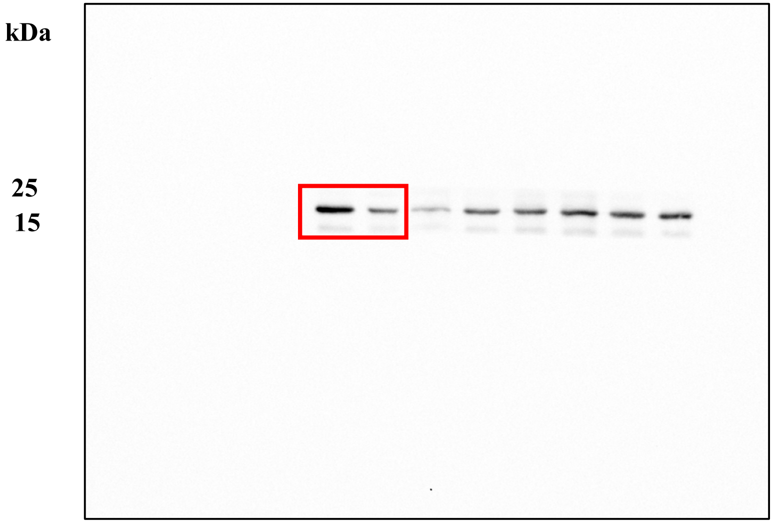

HCT116 Anti-BAX

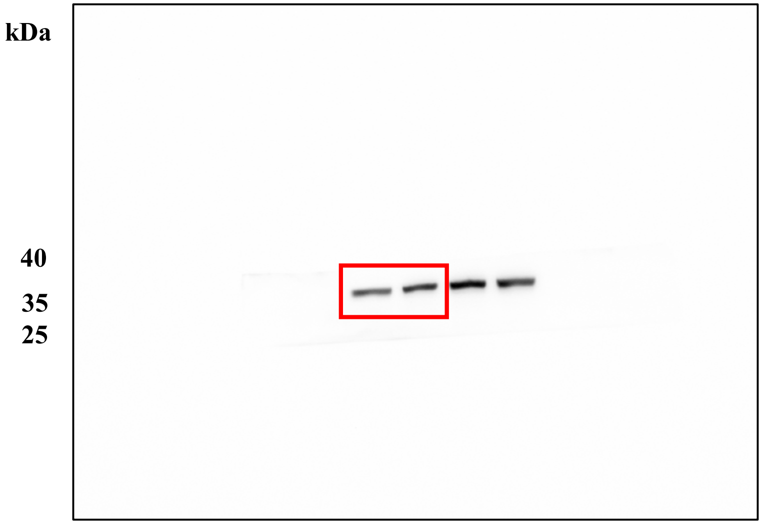

HT29 Anti-BCL-2

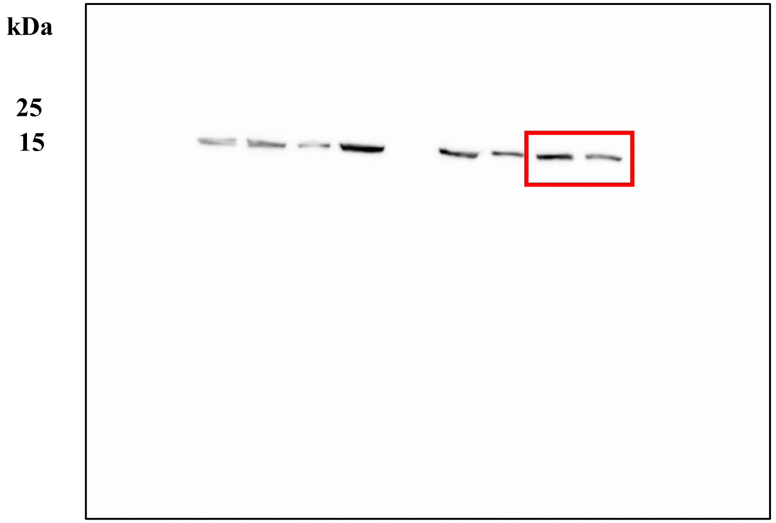

HT29 Anti-BAX

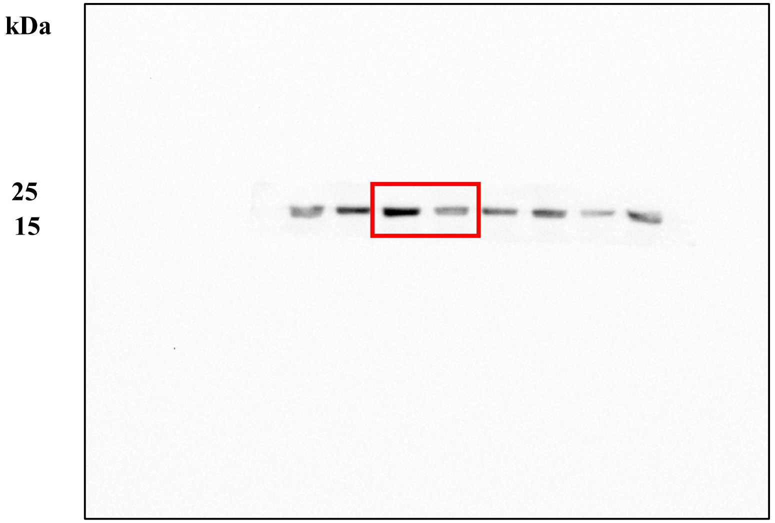

HCT116 Anti-BAX

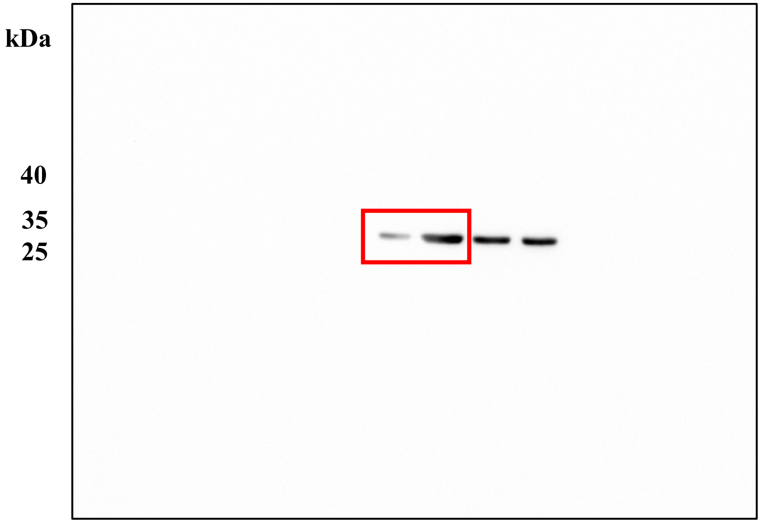

HT29 Anti-BCL-2

Sup F. Full unedited blots for Fig.5c

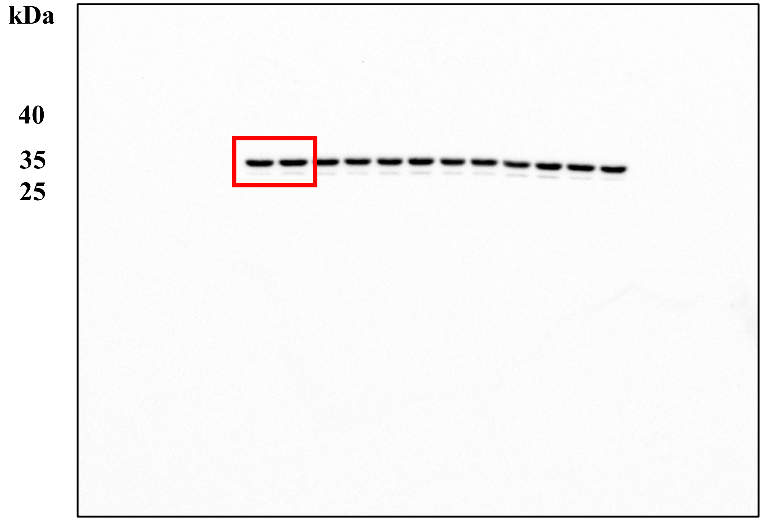

HT29 Anti-GAPDH

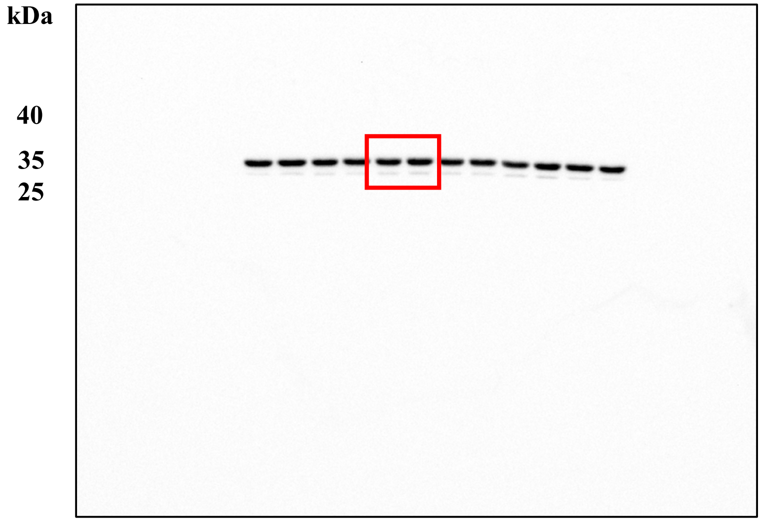

HCT116 Anti-GAPDH

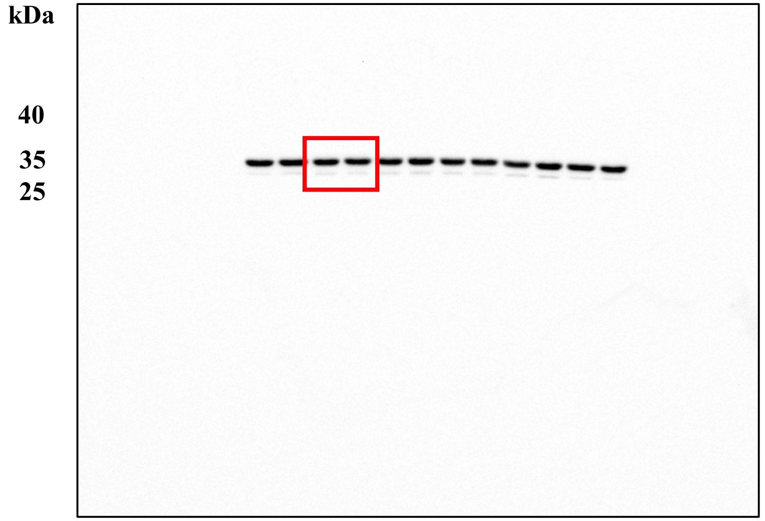

HT29 Anti-GAPDH

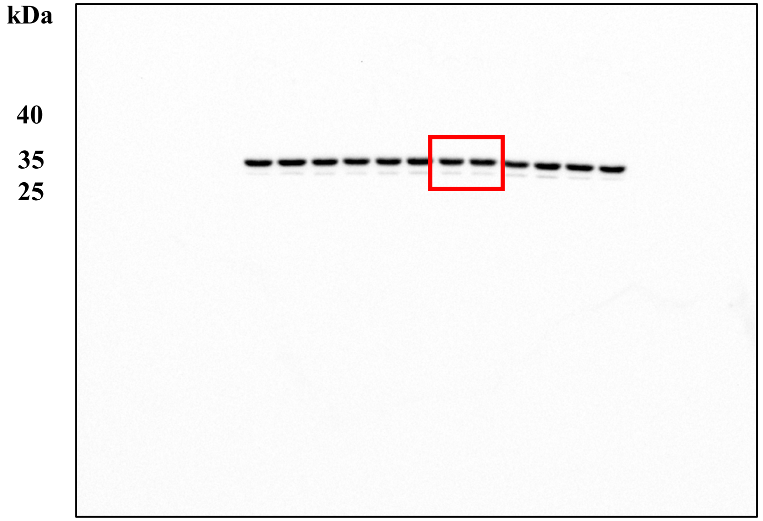

HCT116 Anti-GAPDH

G. Full unedited blots for Fig.5d HT29

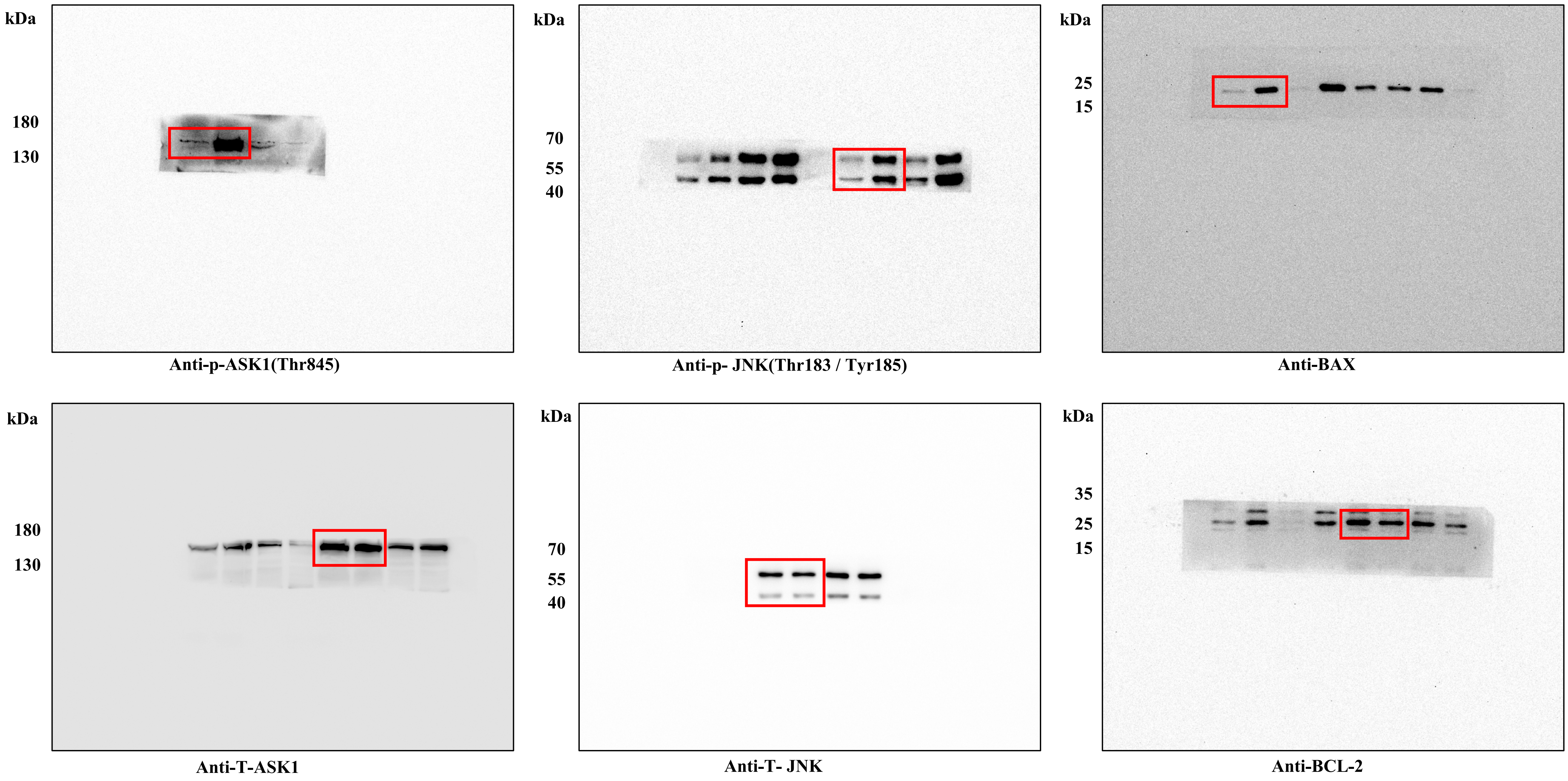

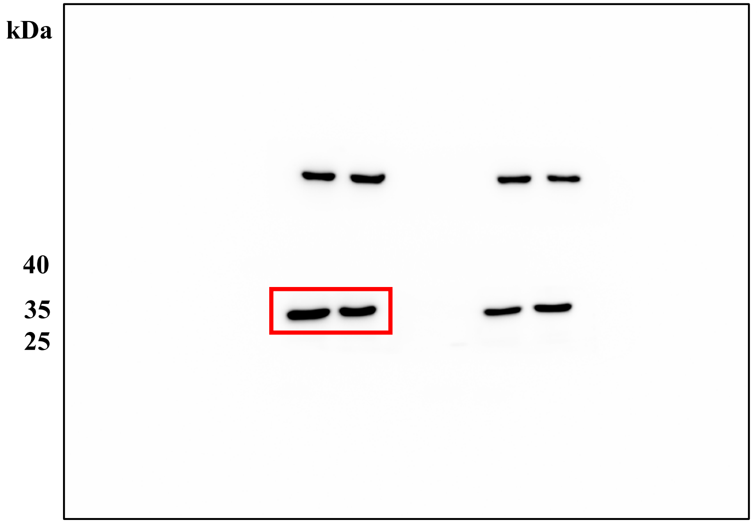

Anti-GAPDH

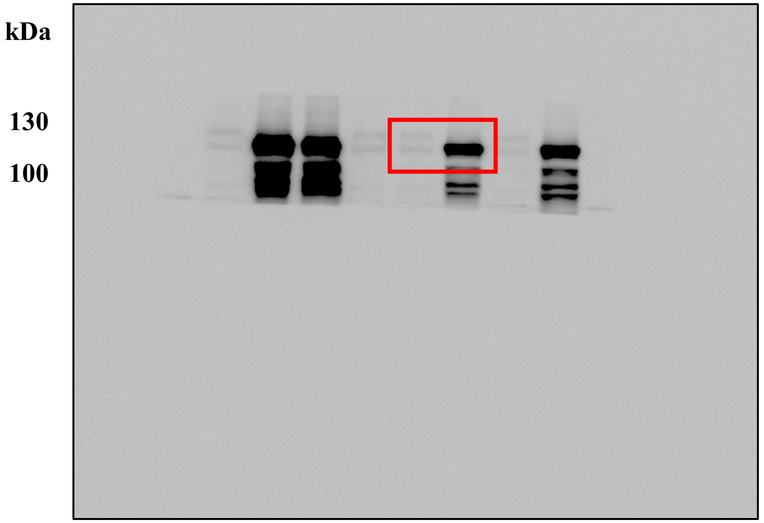

Anti-DAB2IP

G. Full unedited blots for Fig.5d HCT116

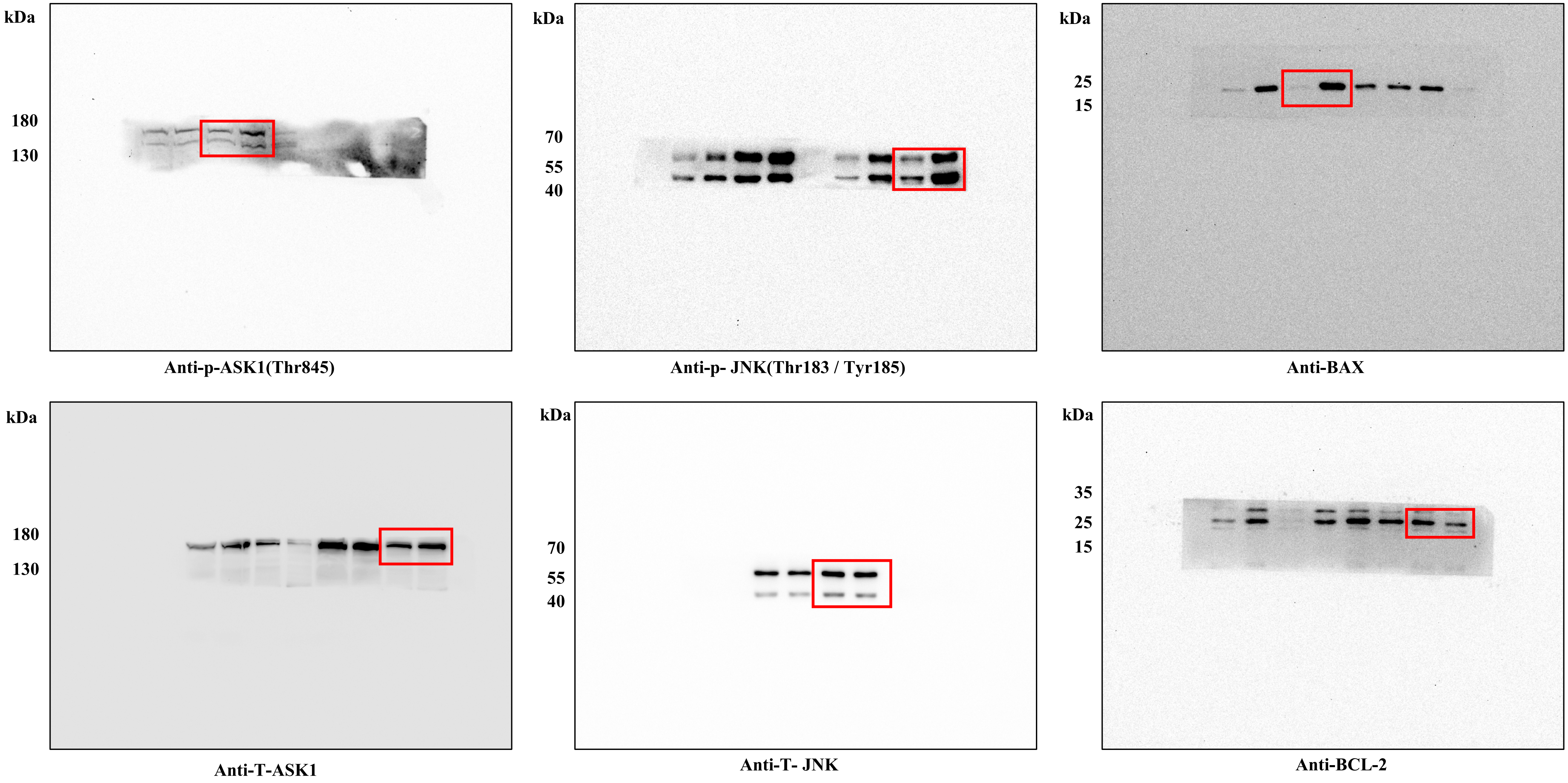

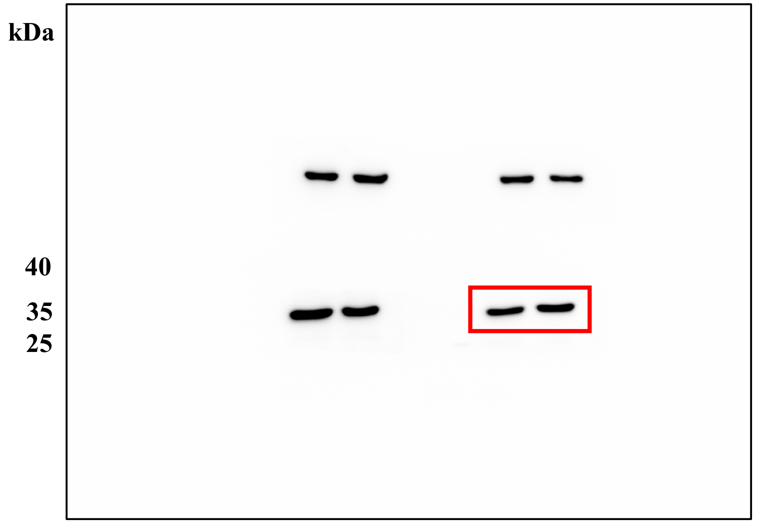

Anti-GAPDH

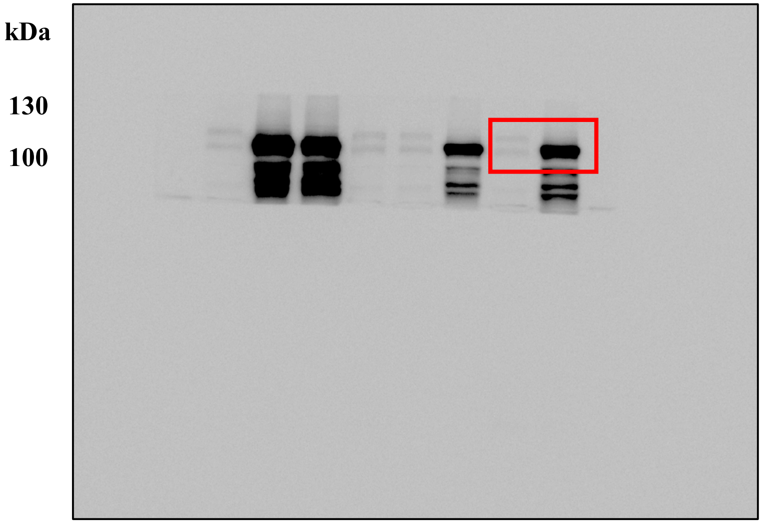

Anti-DAB2IP

Sup G. Full unedited blots for Fig.5d

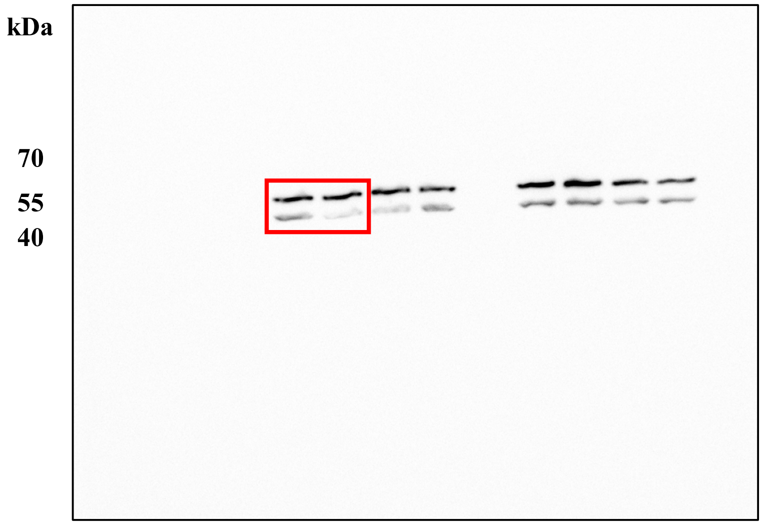

HT29 Anti-T- JNK

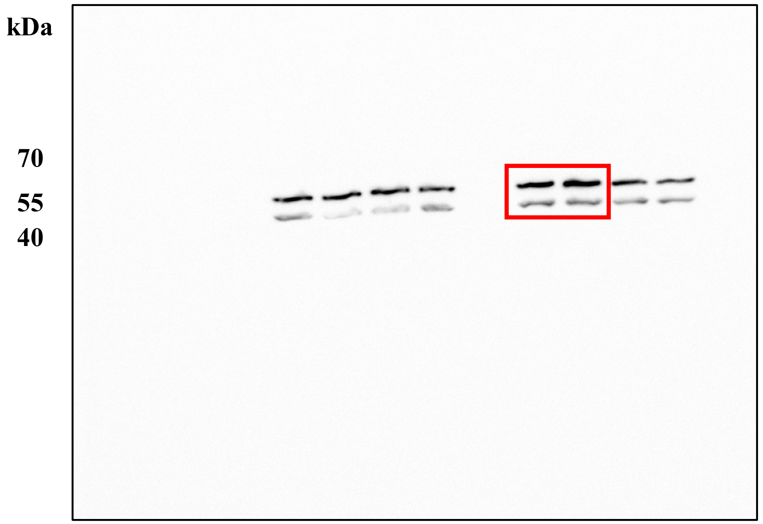

HCT16 Anti-T- JNK

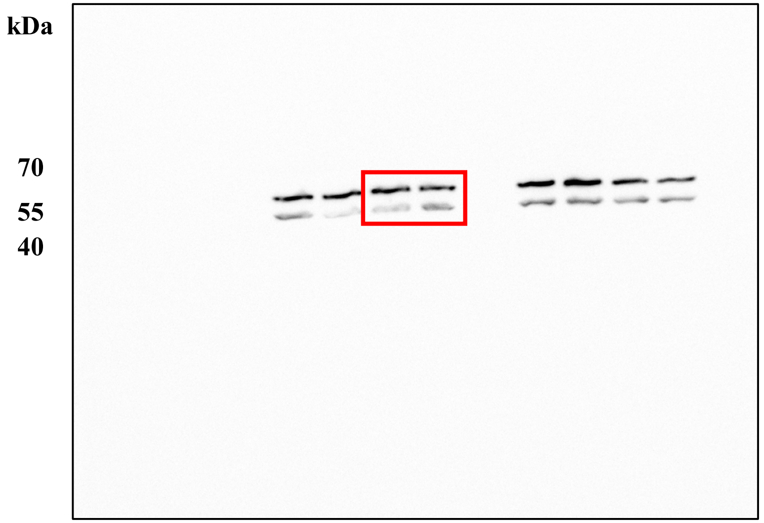

HT29 Anti-T- JNK

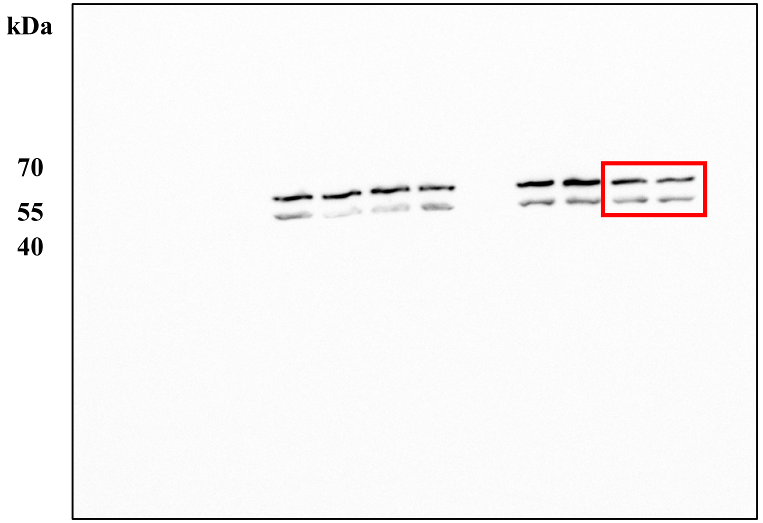

HCT16 Anti-T- JNK

Sup G. Full unedited blots for Fig.5d

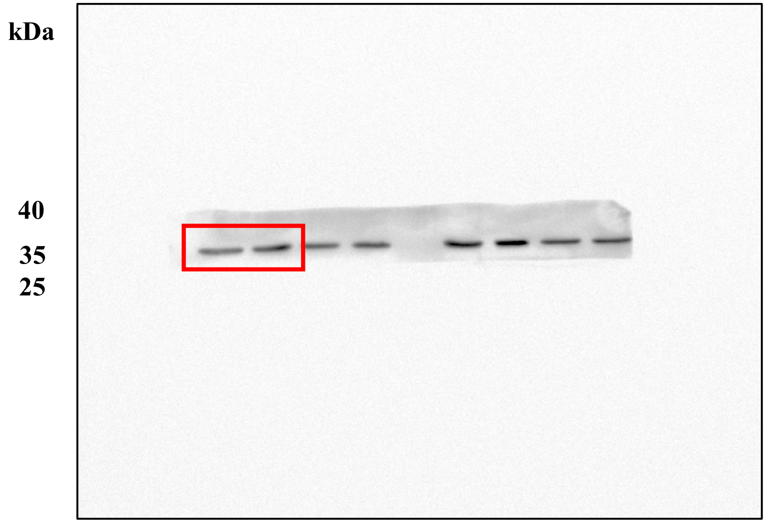

HT29 Anti-GAPDH

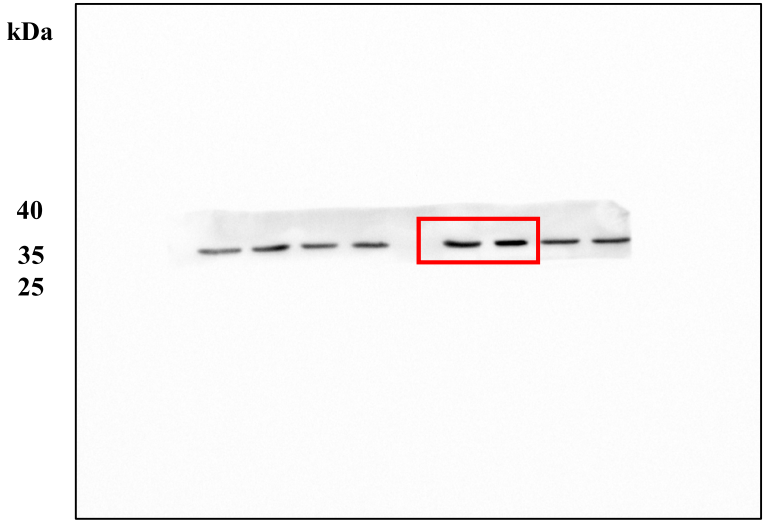

HCT116 Anti-GAPDH

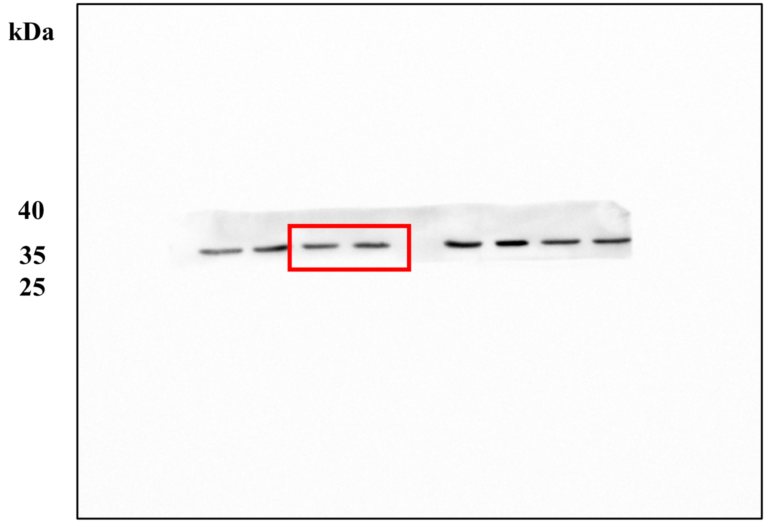

HT29 Anti-GAPDH

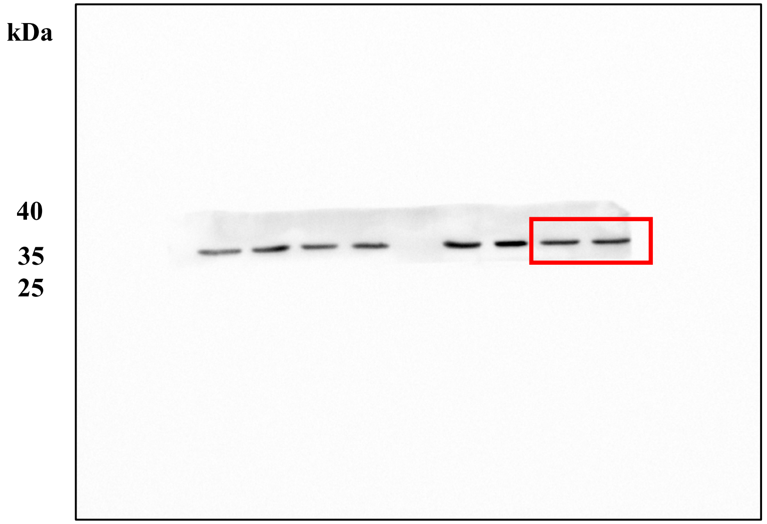

HCT116 Anti-GAPDH

H. Full unedited blots for Fig.5e HT29

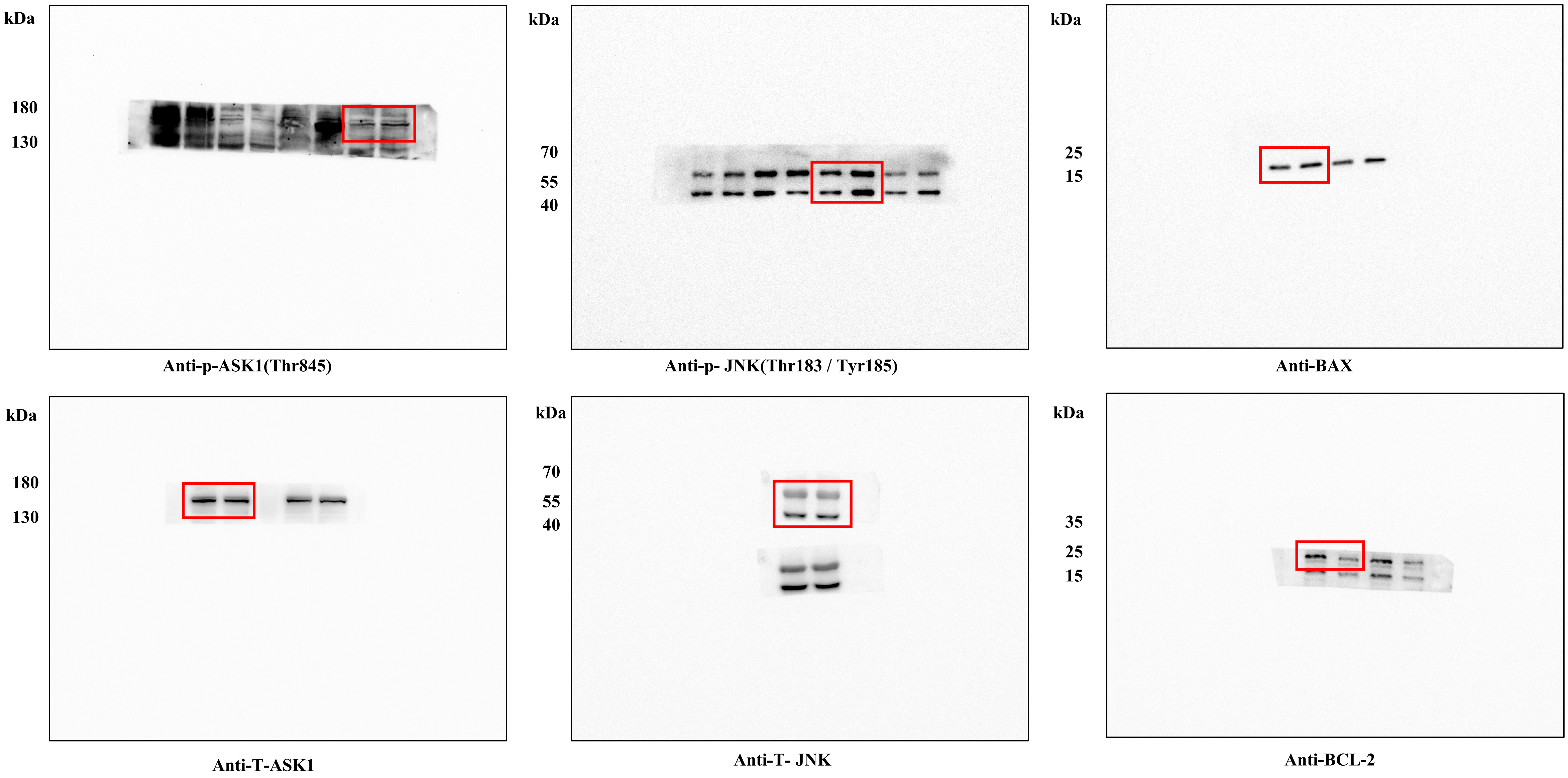

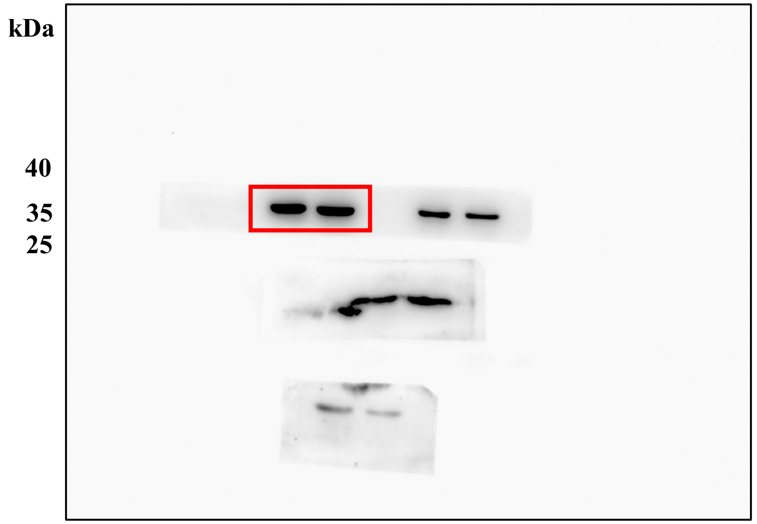

Anti-GAPDH

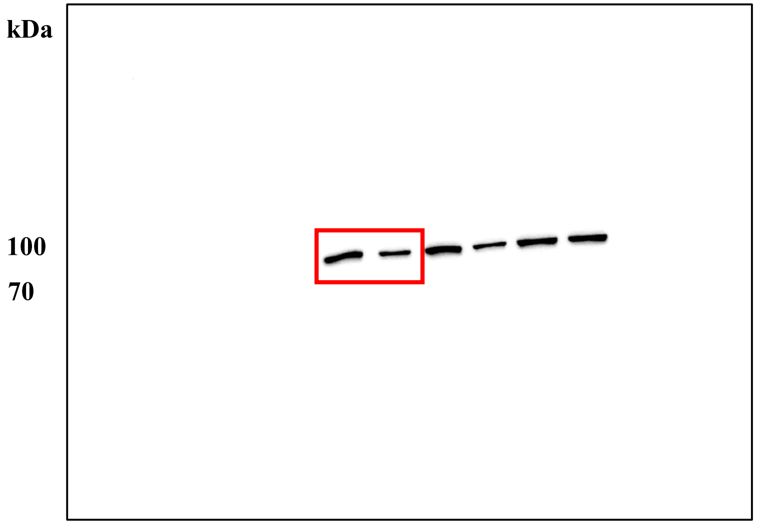

Anti-HSP90AA1

H. Full unedited blots for Fig.5e HCT116

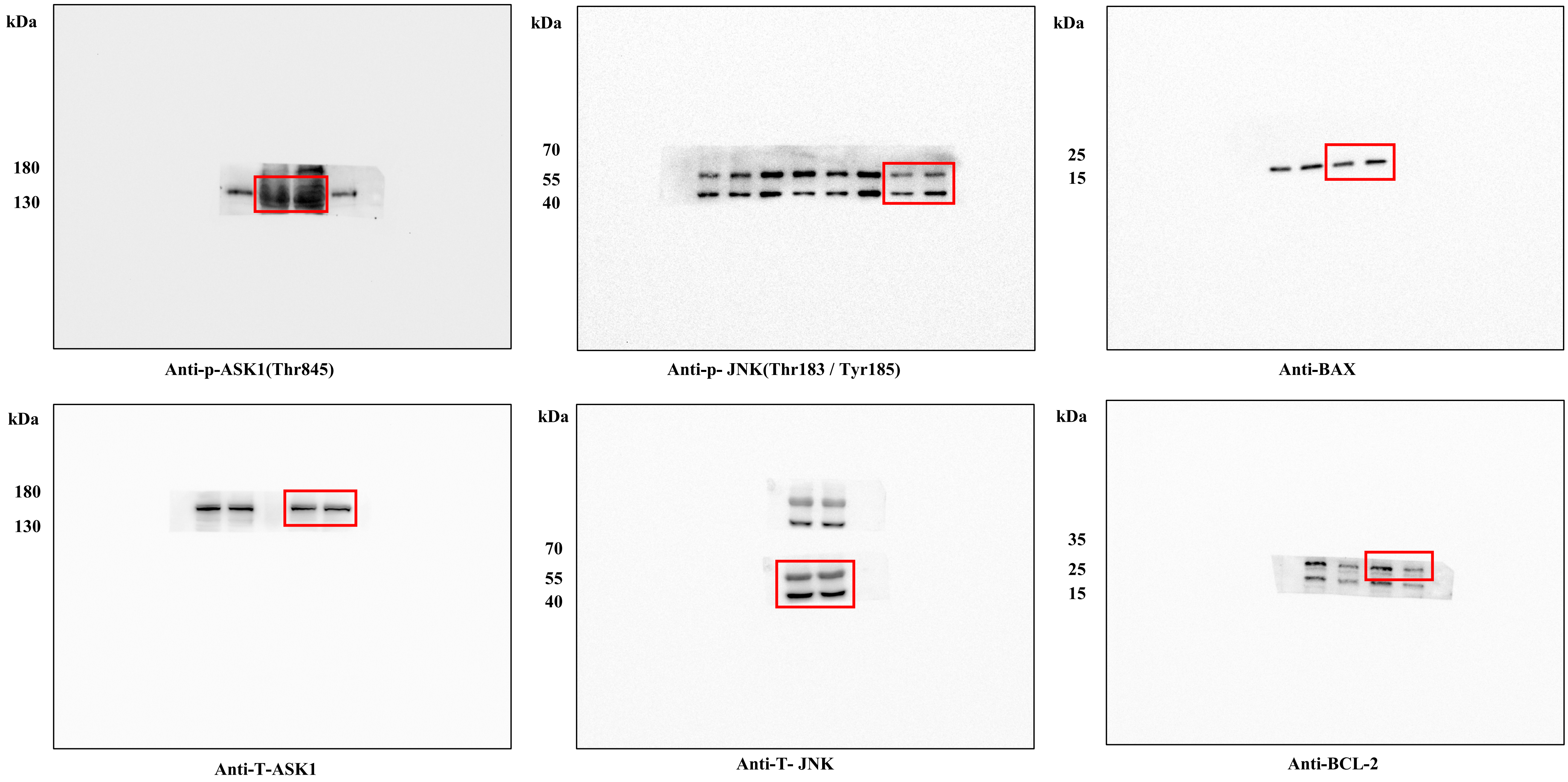

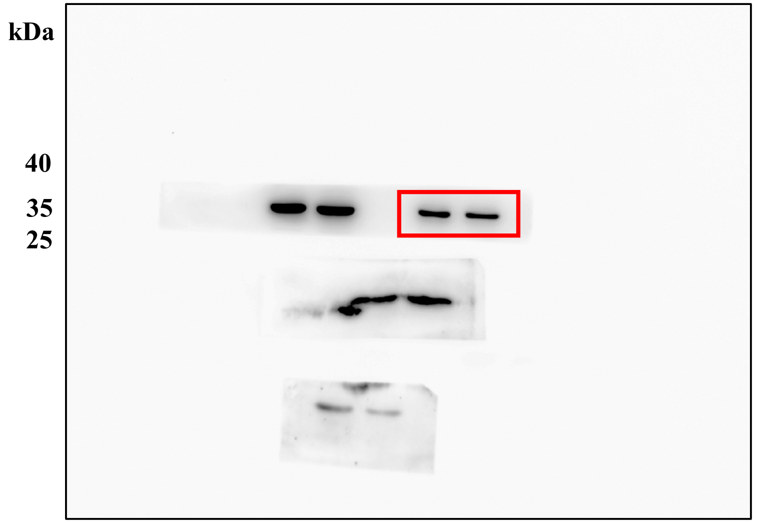

Anti-GAPDH

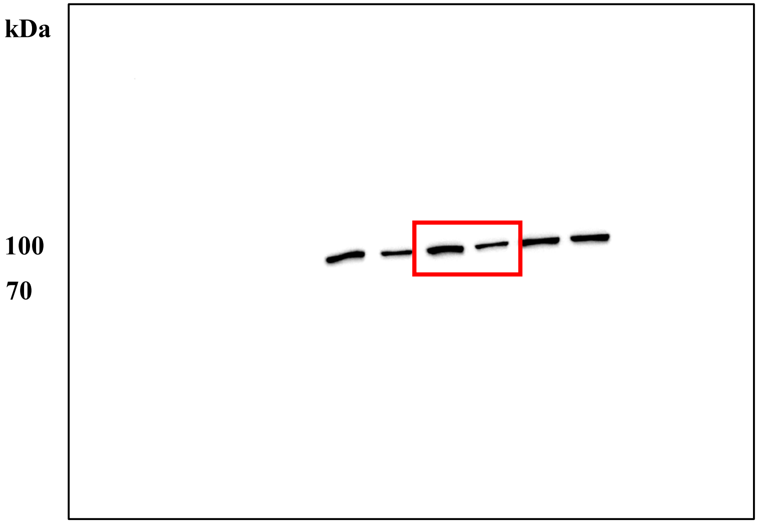

Anti-HSP90AA1

Sup H. Full unedited blots for Fig.5e

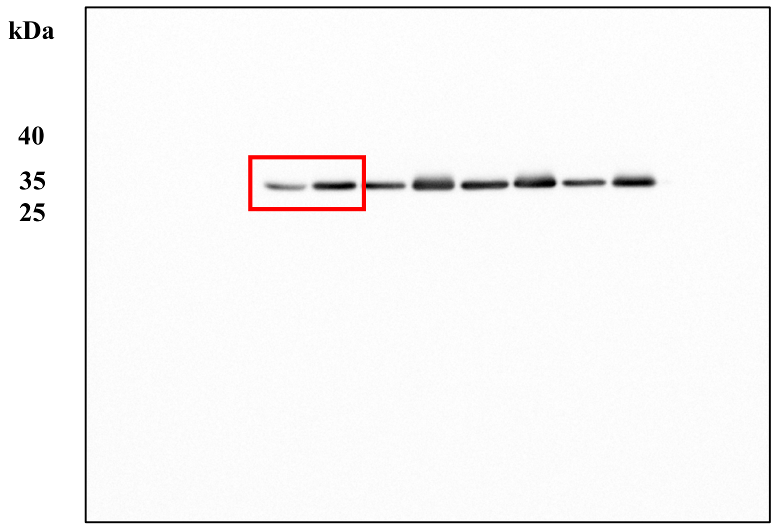

HT29 Anti-BAX

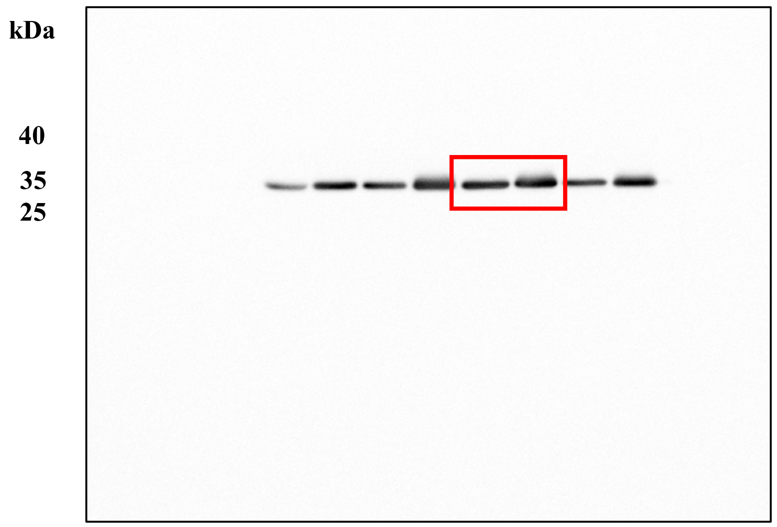

HCT116 Anti-BAX

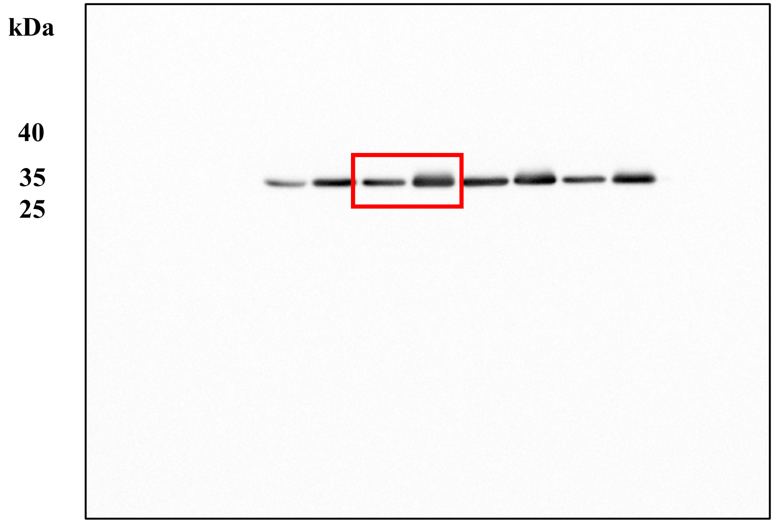

HT29 Anti-BAX

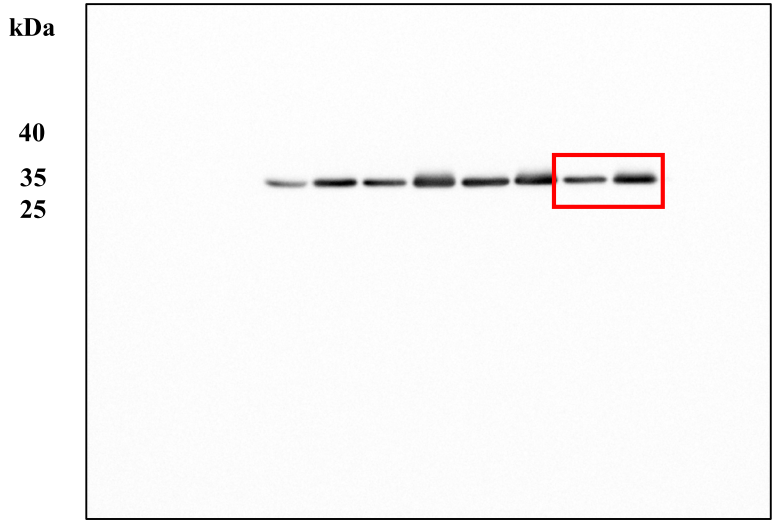

HCT116 Anti-BAX

Sup H. Full unedited blots for Fig.5e

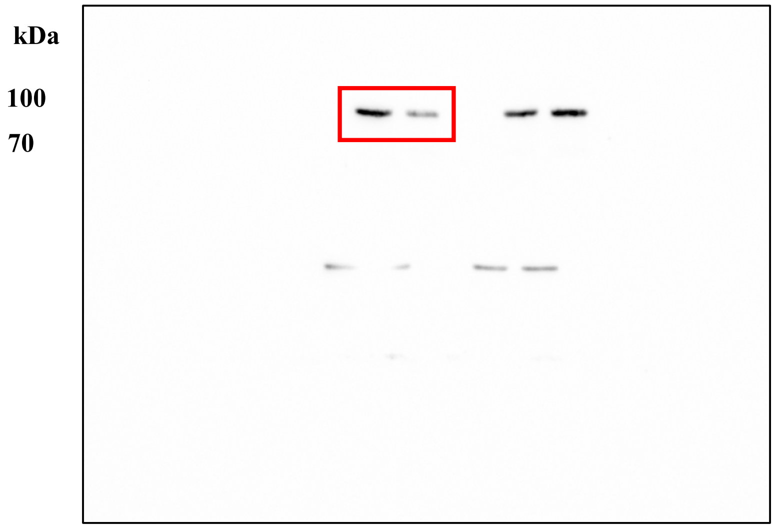

HT29 Anti-HSP90AA1

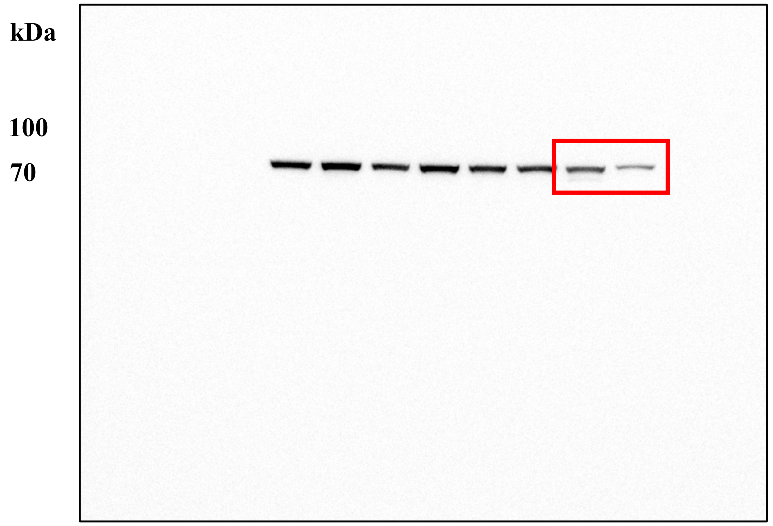

HCT116 Anti-HSP90AA1

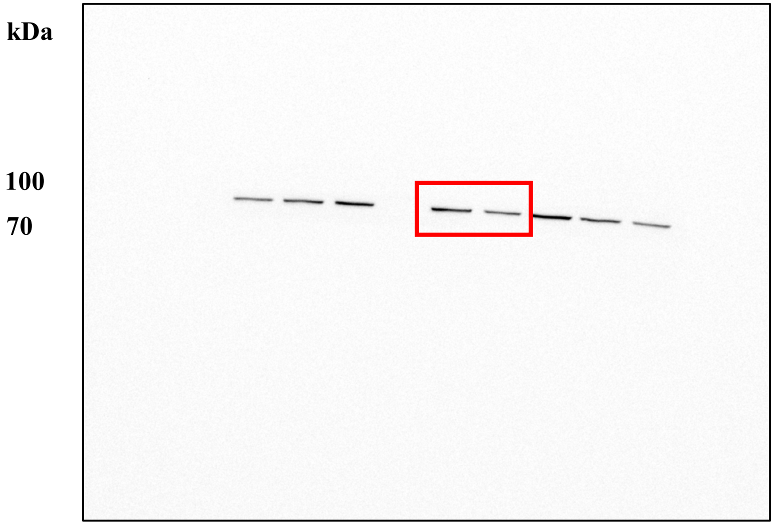

HT29 Anti-HSP90AA1

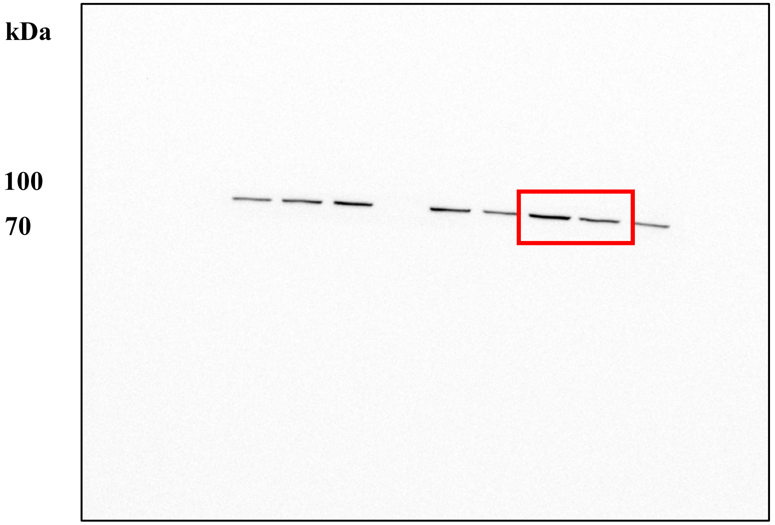

HCT116 Anti-HSP90AA1

I. Full unedited blots for Fig.5f HT29

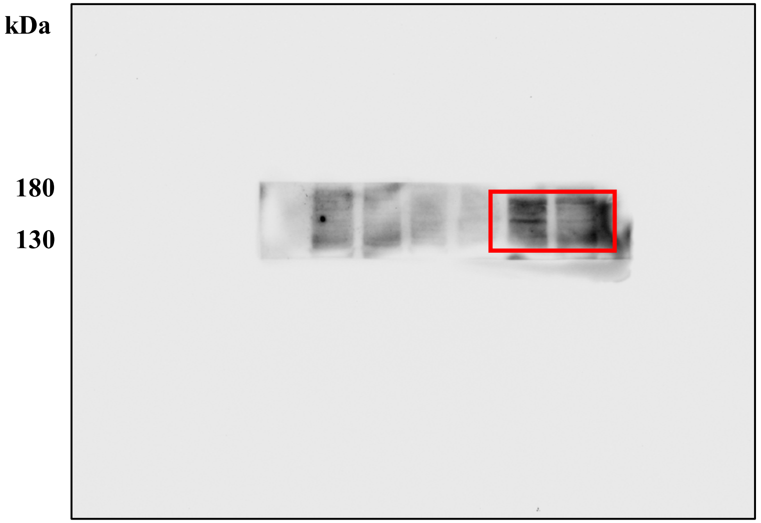

Anti-p-ASK1(Thr845)

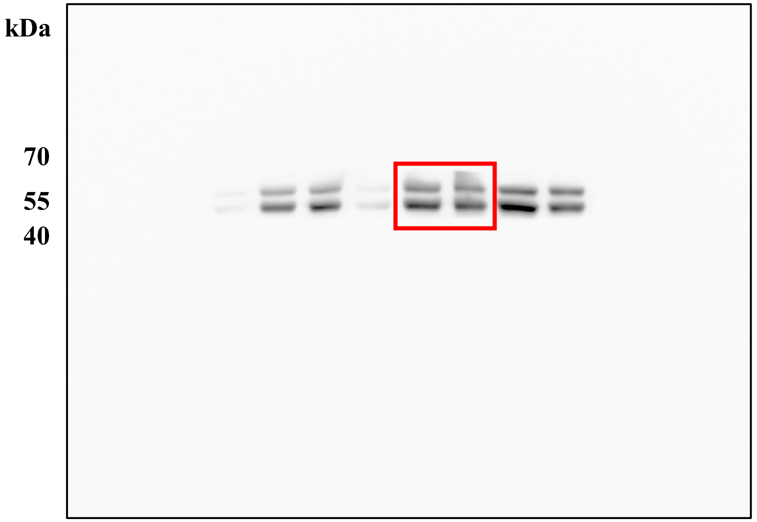

Anti-p- JNK(Thr183 / Tyr185)

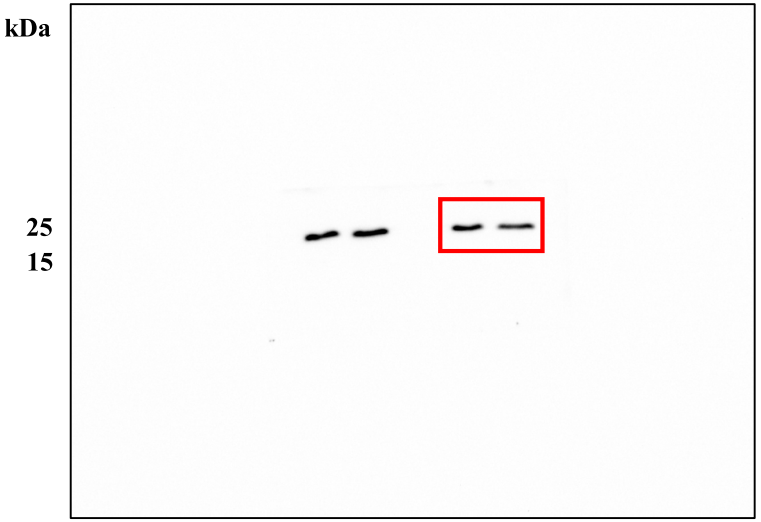

Anti-BAX

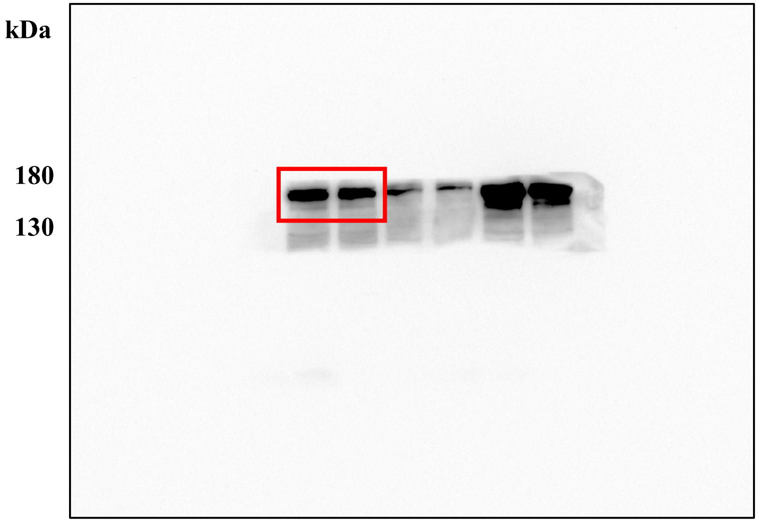

Anti-T-ASK1

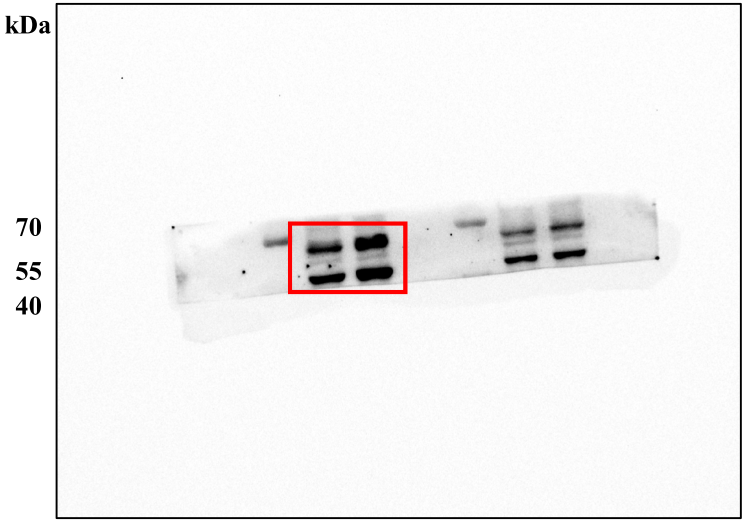

Anti-T- JNK

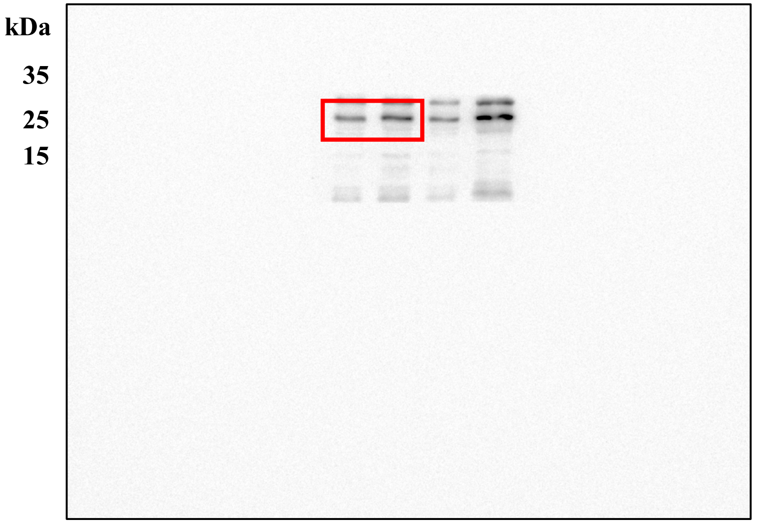

Anti-BCL-2

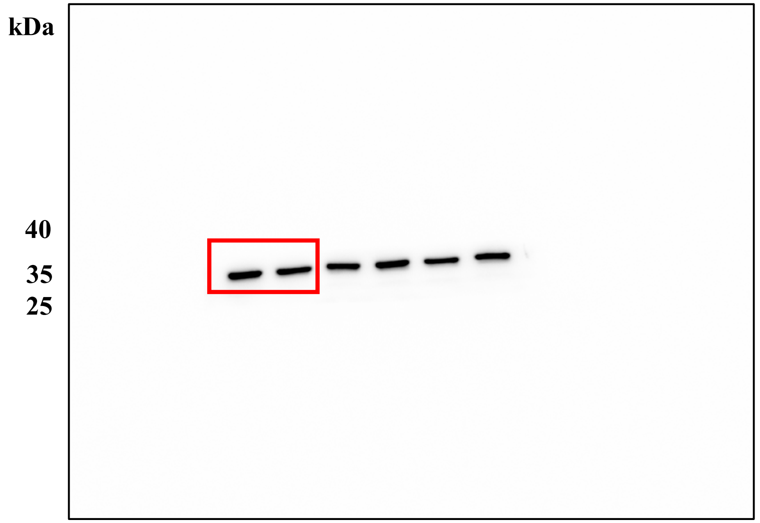

Anti-GAPDH

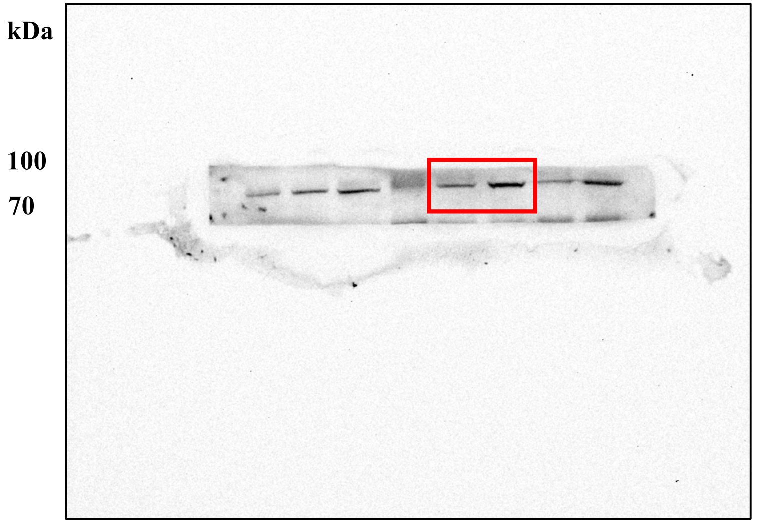

Anti-HSP90AA1

I. Full unedited blots for Fig.5f HCT116

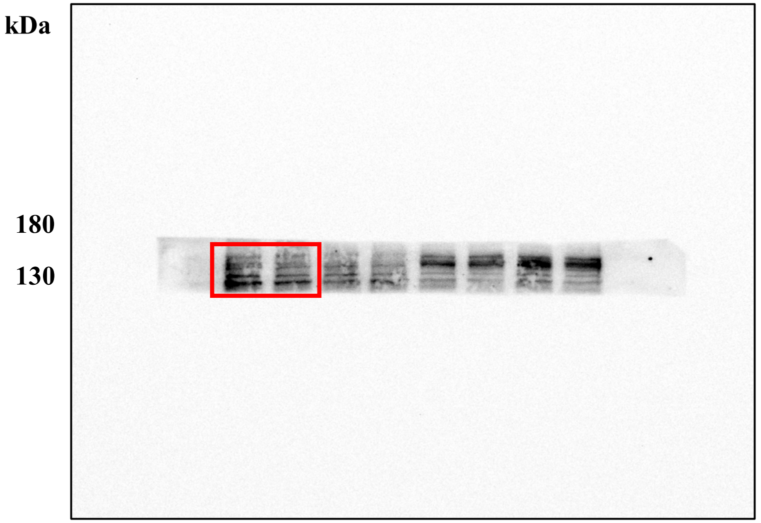

Anti-p-ASK1(Thr845)

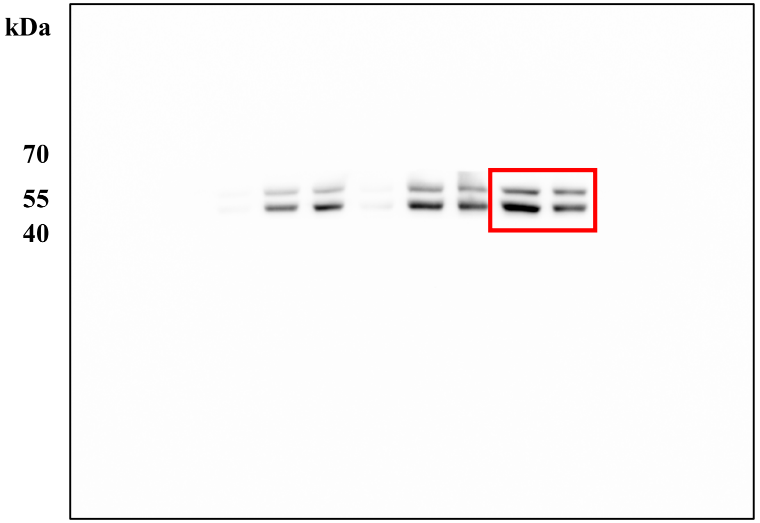

Anti-p- JNK(Thr183 / Tyr185)

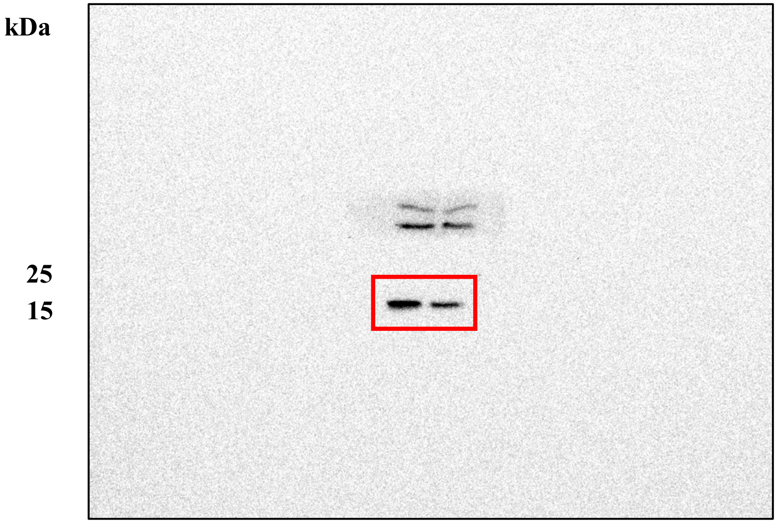

Anti-BAX

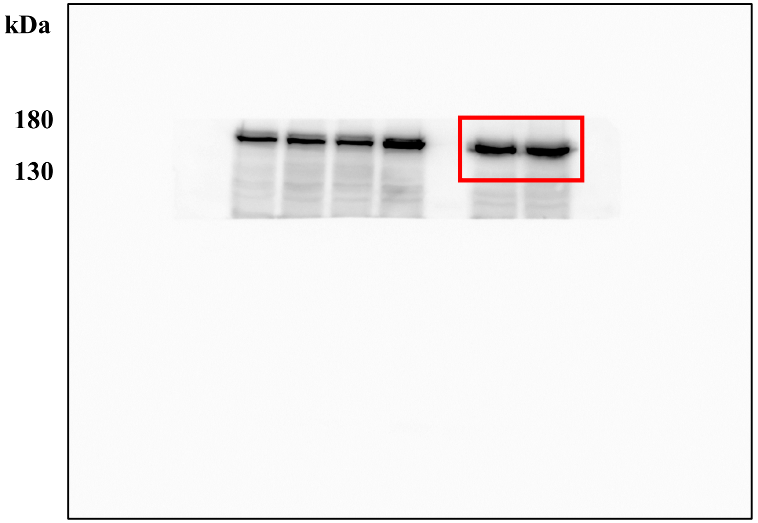

Anti-T-ASK1

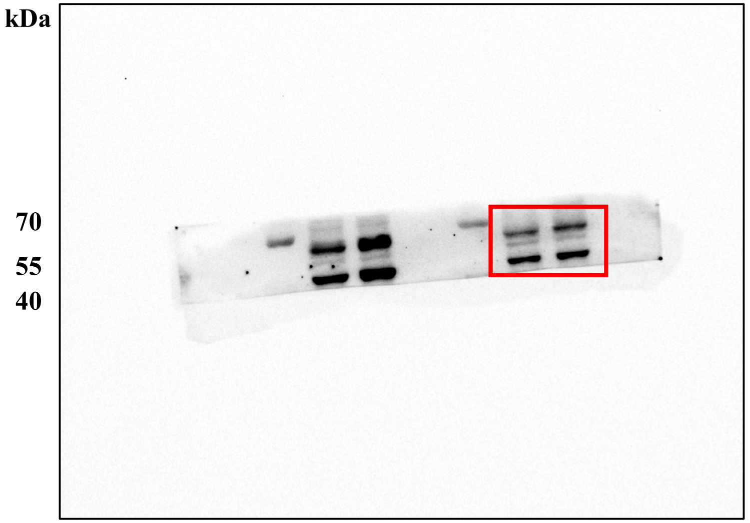

Anti-T- JNK

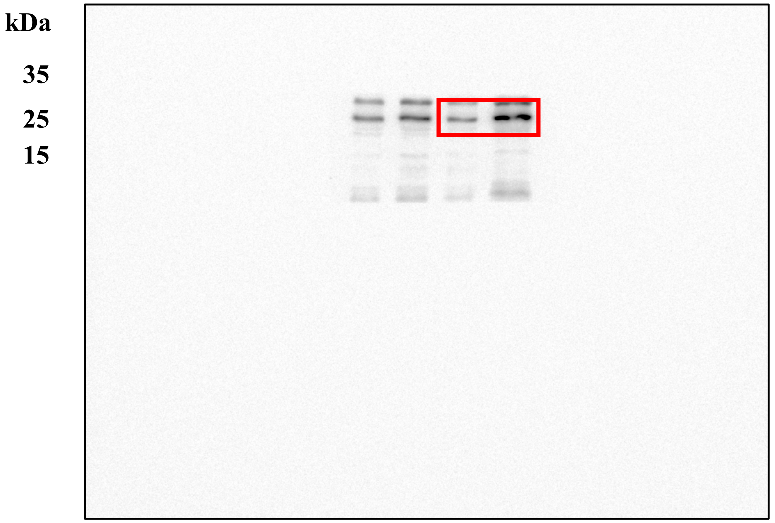

Anti-BCL-2

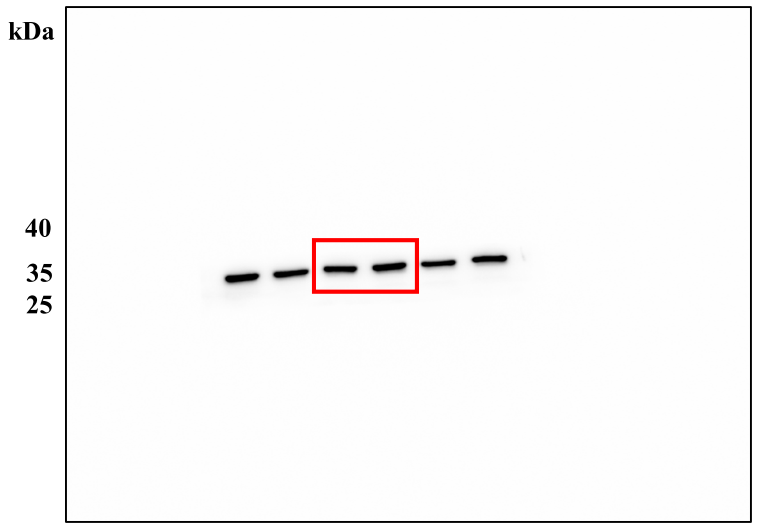

Anti-GAPDH

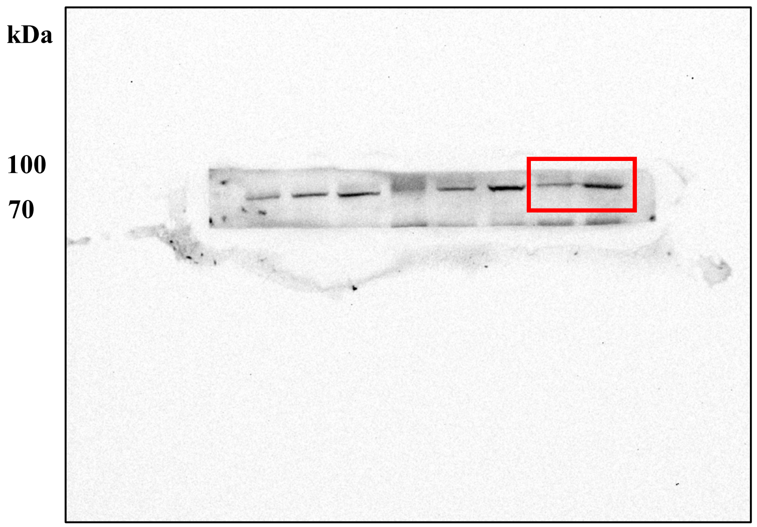

Anti-HSP90AA1

Sup I. Full unedited blots for Fig.5f

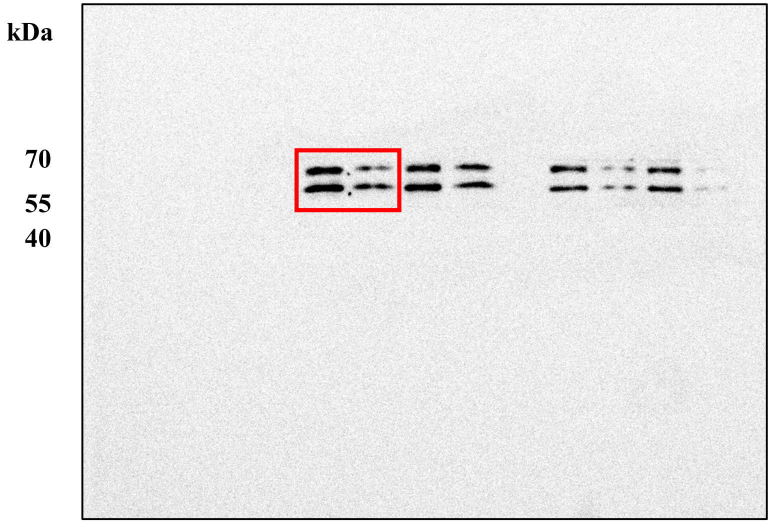

HT29 Anti-p- JNK(Thr183 / Tyr185)

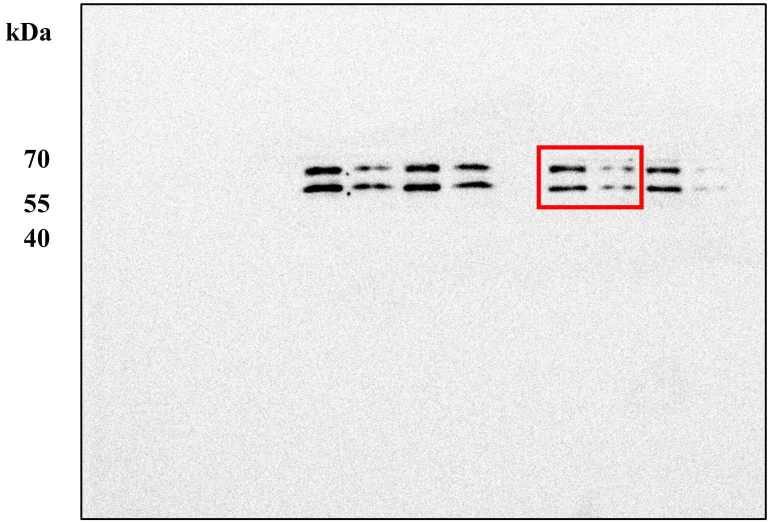

HCT116 Anti-p- JNK(Thr183 / Tyr185)

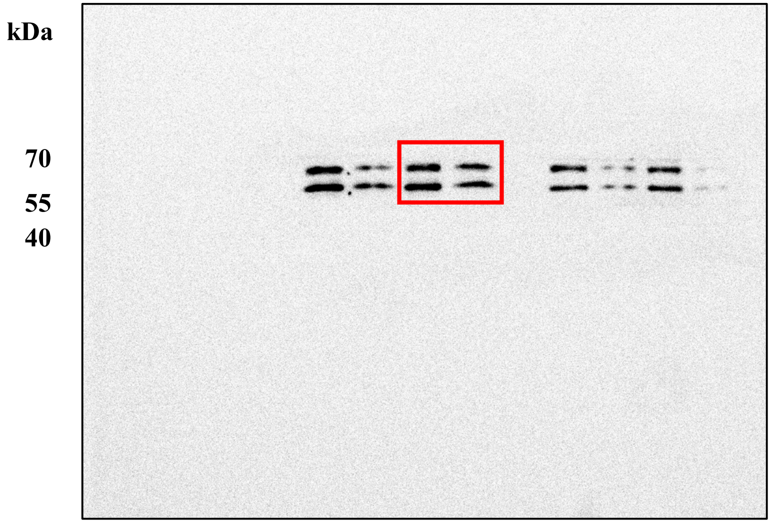

HT29 Anti-p- JNK(Thr183 / Tyr185)

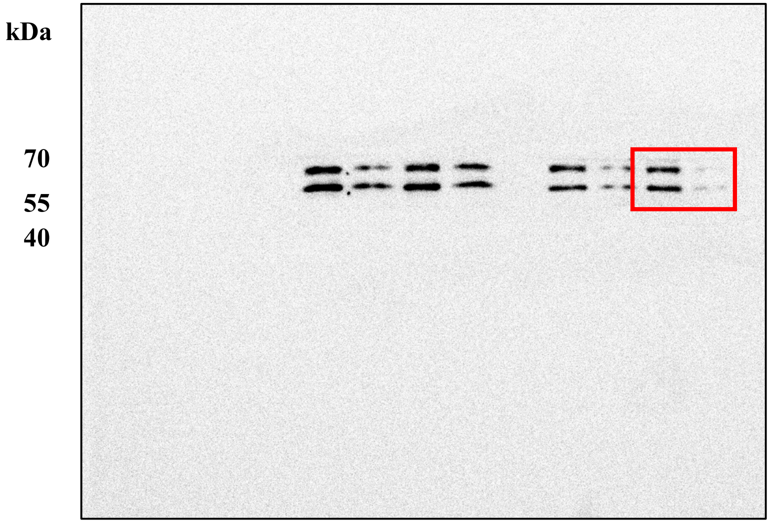

HCT116 Anti-p- JNK(Thr183 / Tyr185)

Sup I. Full unedited blots for Fig.5f

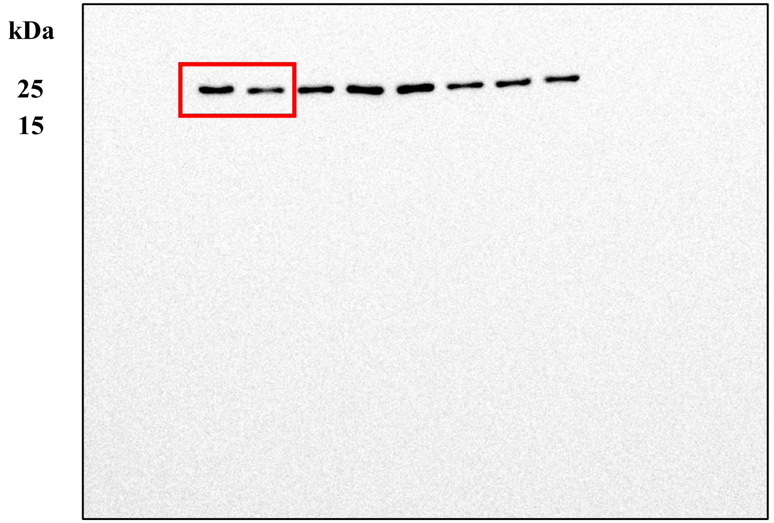

HT29 Anti-BAX

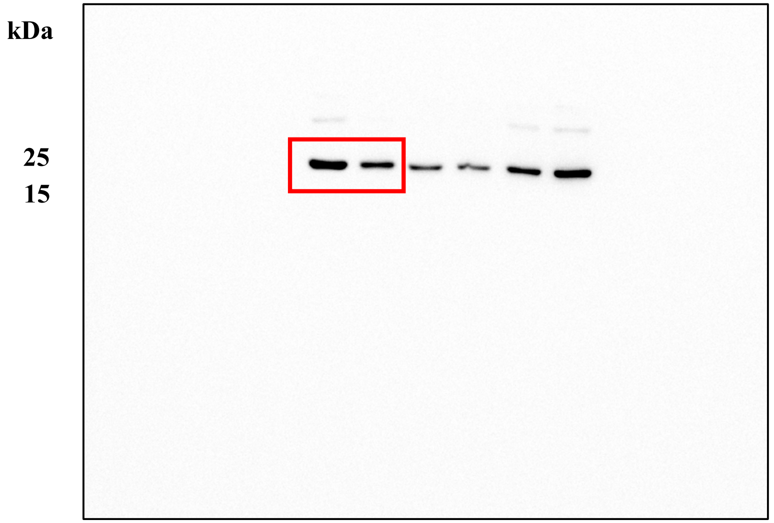

HCT116 Anti-BAX

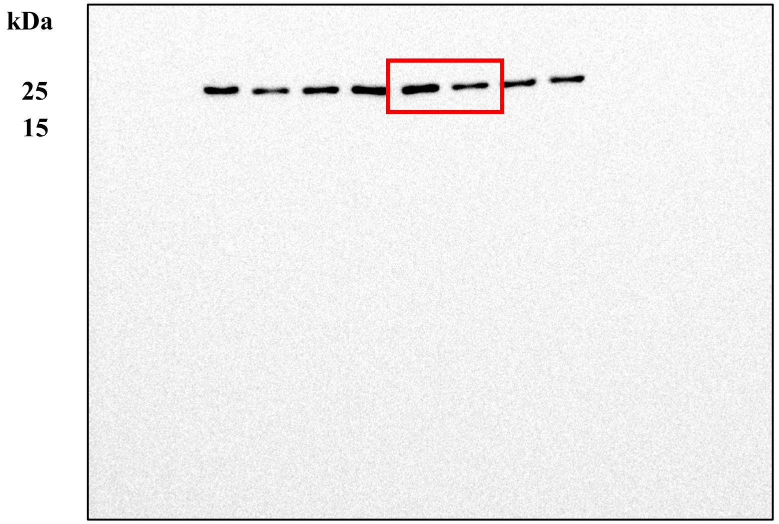

HT29 Anti-BAX

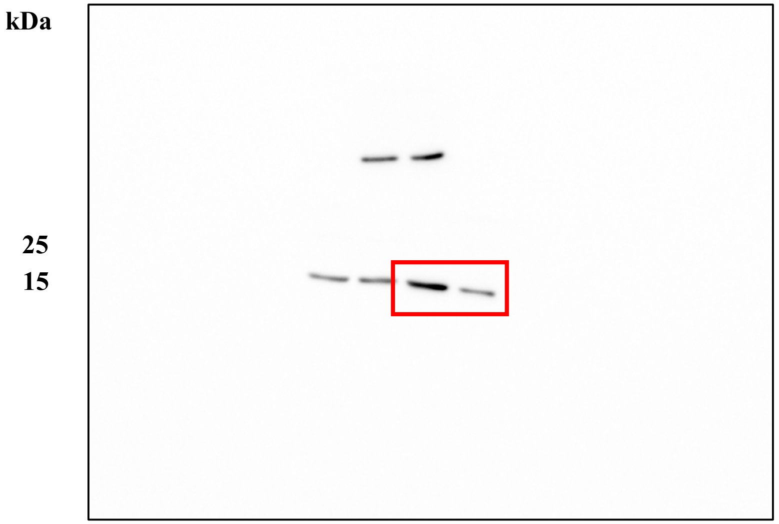

HCT116 Anti-BAX

Sup I. Full unedited blots for Fig.5f

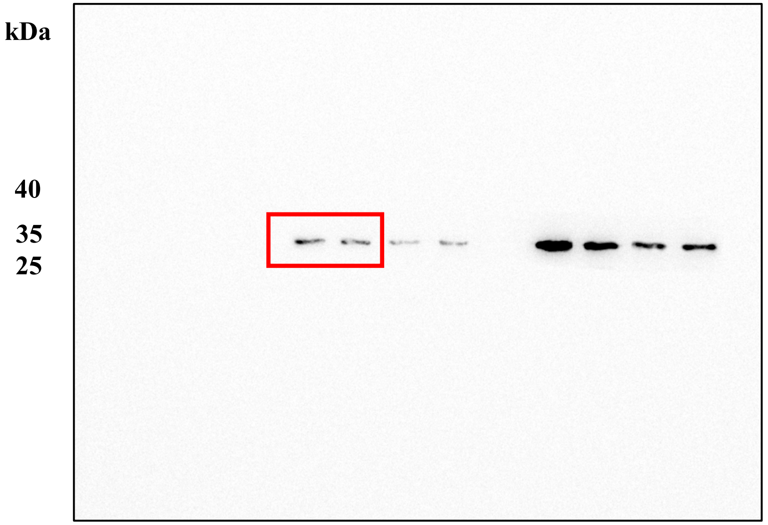

HT29 Anti-GAPDH

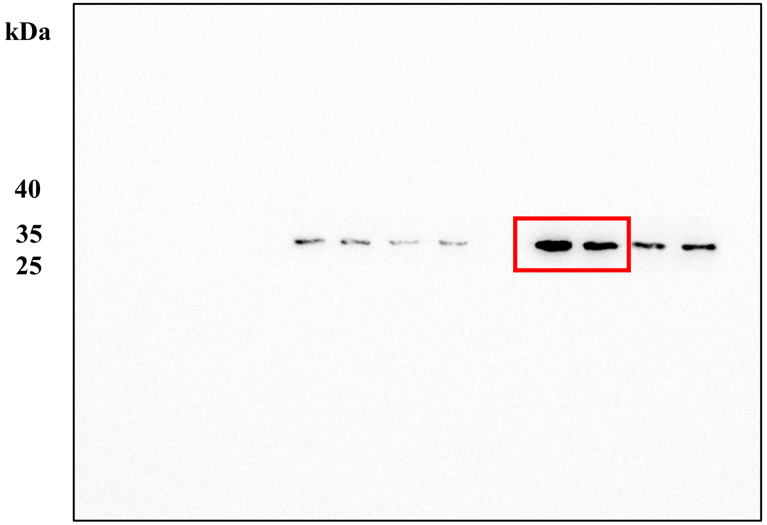

HCT116 Anti-GAPDH

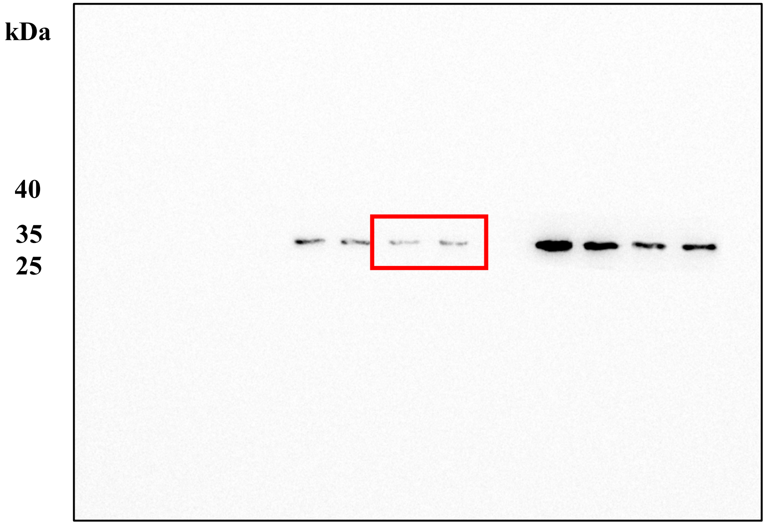

HT29 Anti-GAPDH

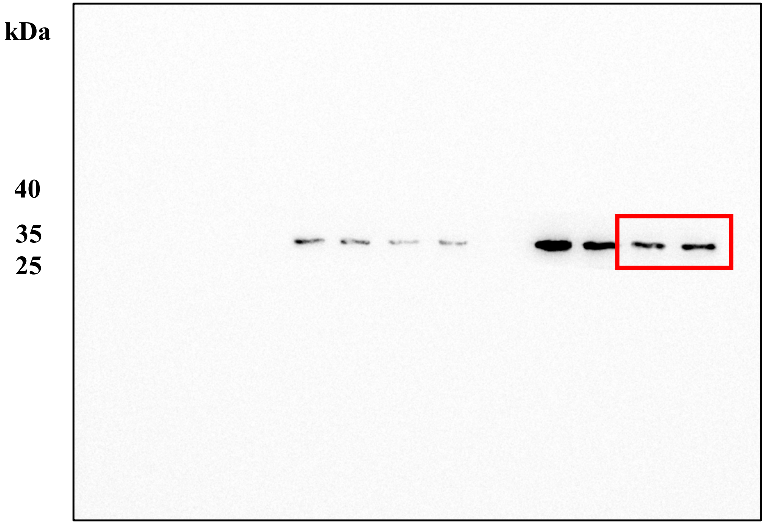

HCT116 Anti-GAPDH

J. Full unedited blots for Fig.5g HT29(si#1)

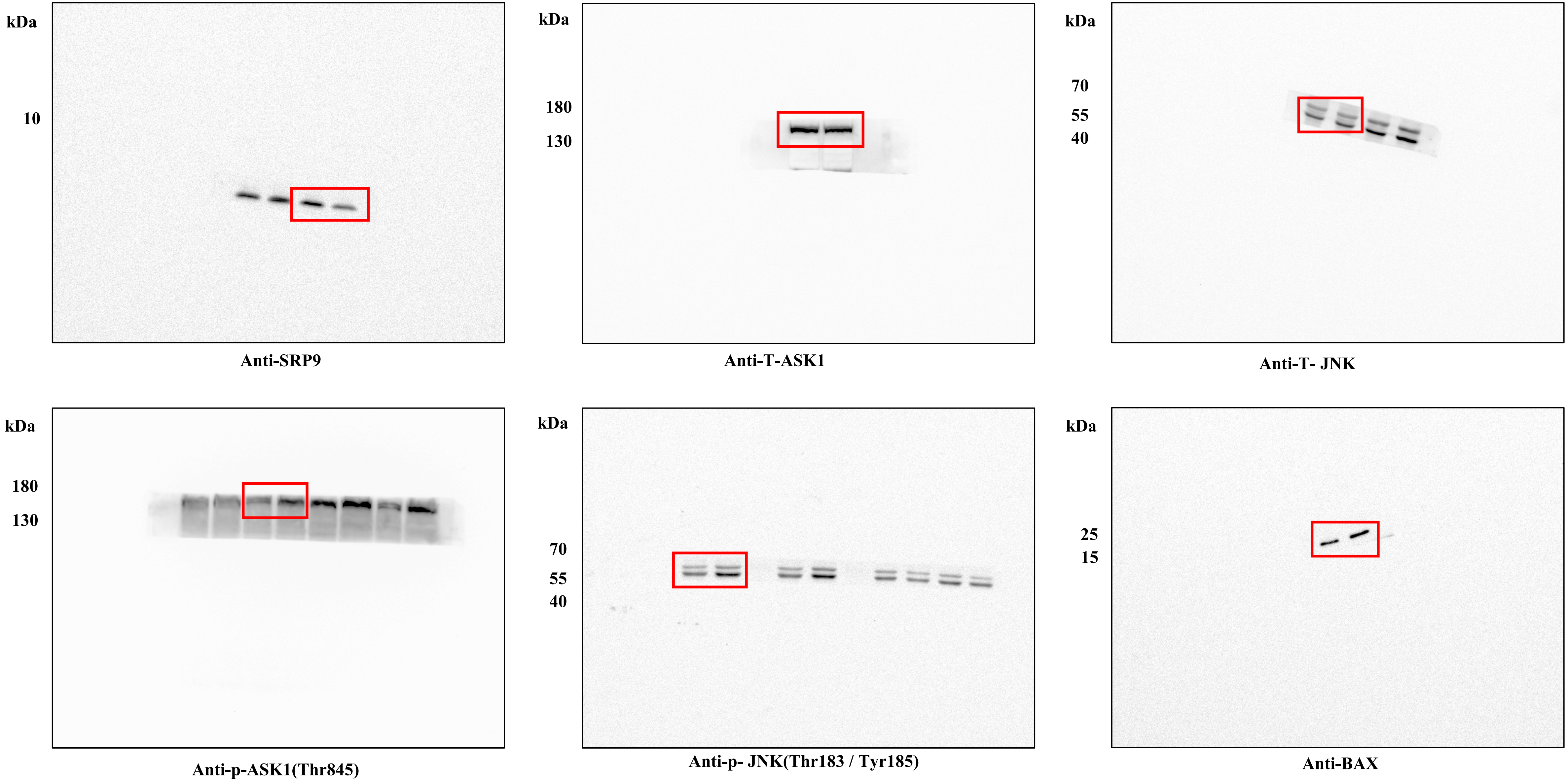

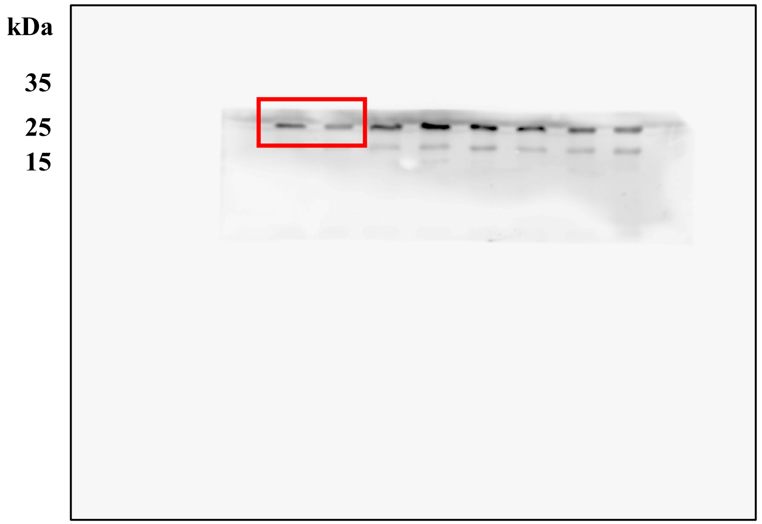

Anti-BCL-2

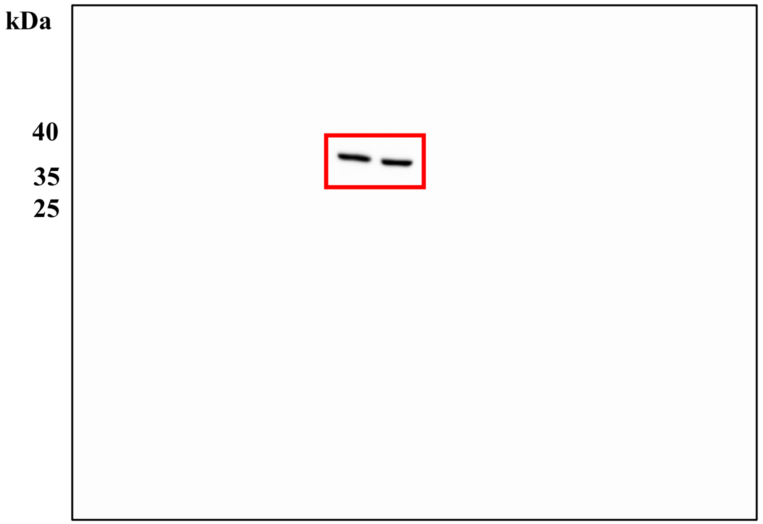

Anti-GAPDH

J. Full unedited blots for Fig.5g HCT116 (si#1)

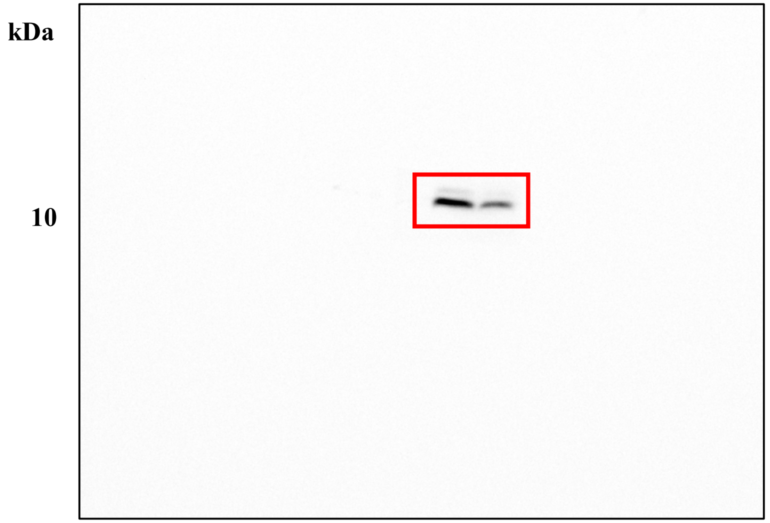

Anti-SRP9

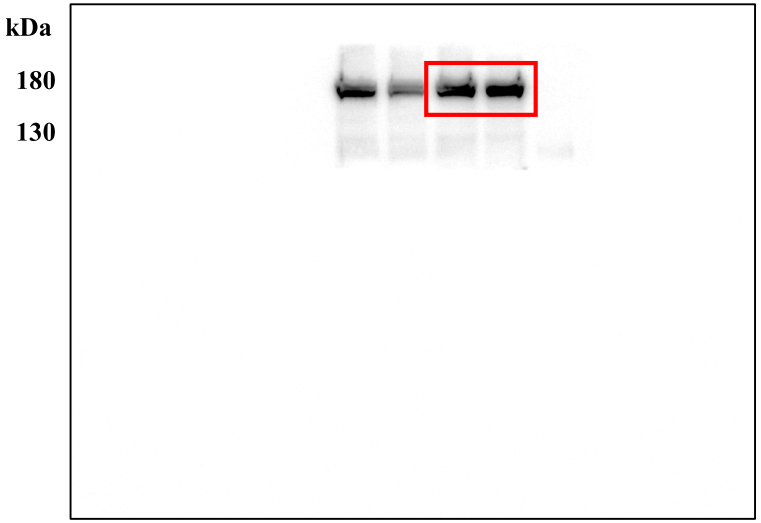

Anti-T-ASK1

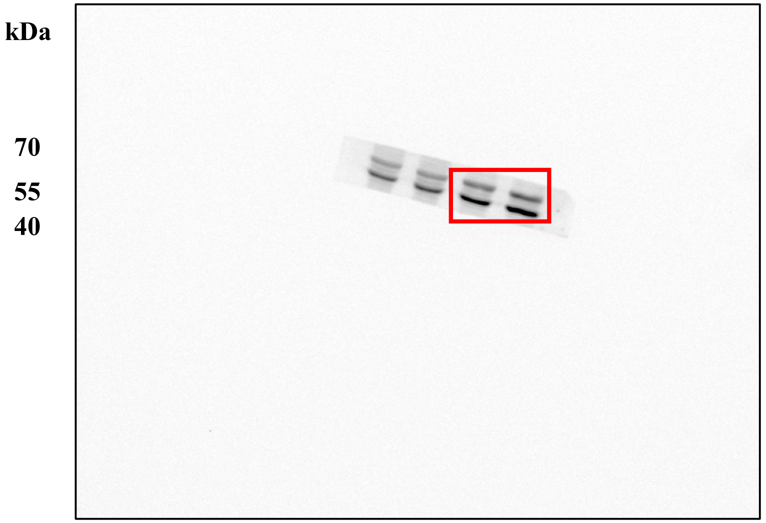

Anti-T- JNK

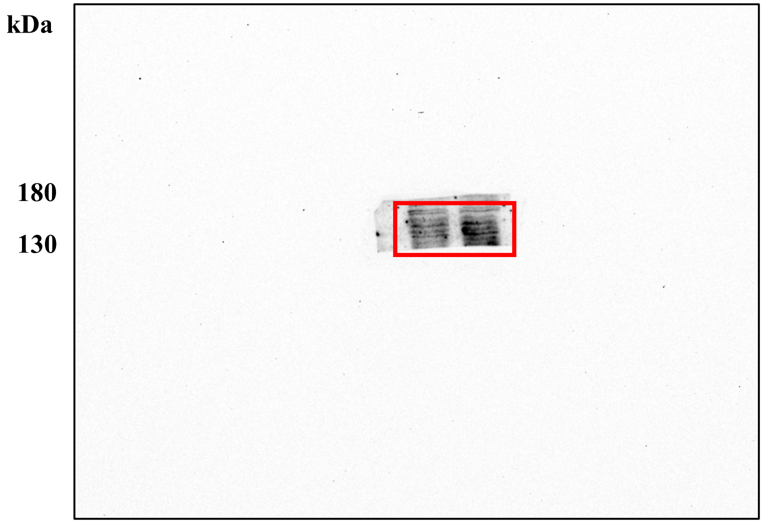

Anti-p-ASK1(Thr845)

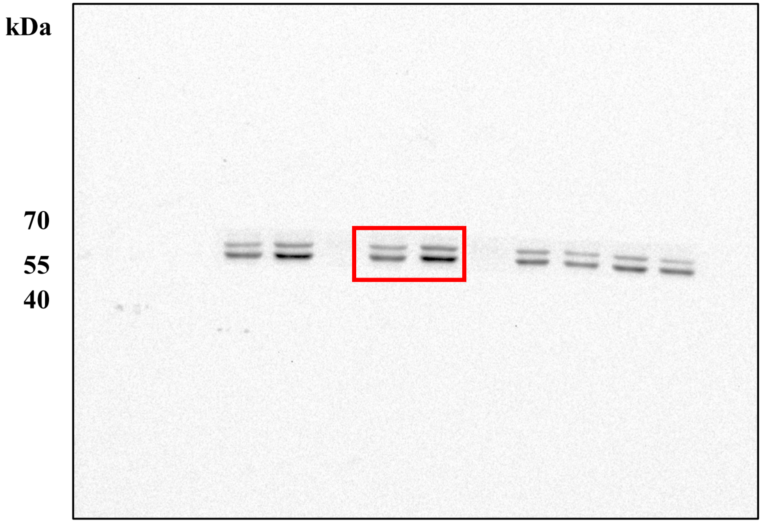

Anti-p- JNK(Thr183 / Tyr185)

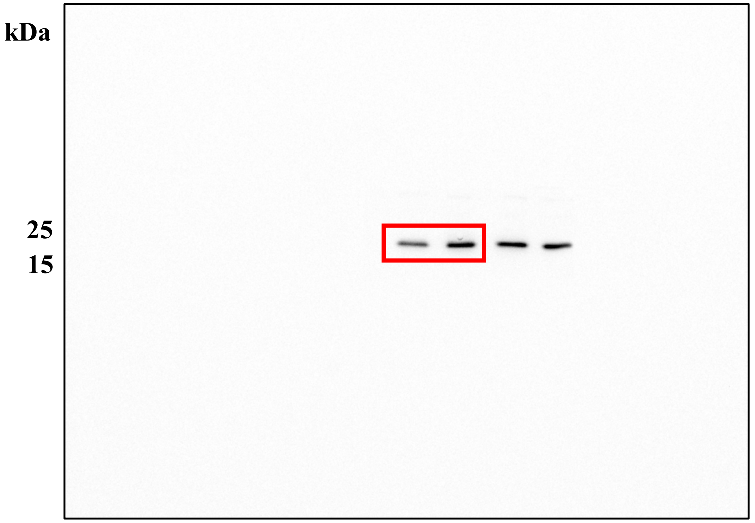

Anti-BAX

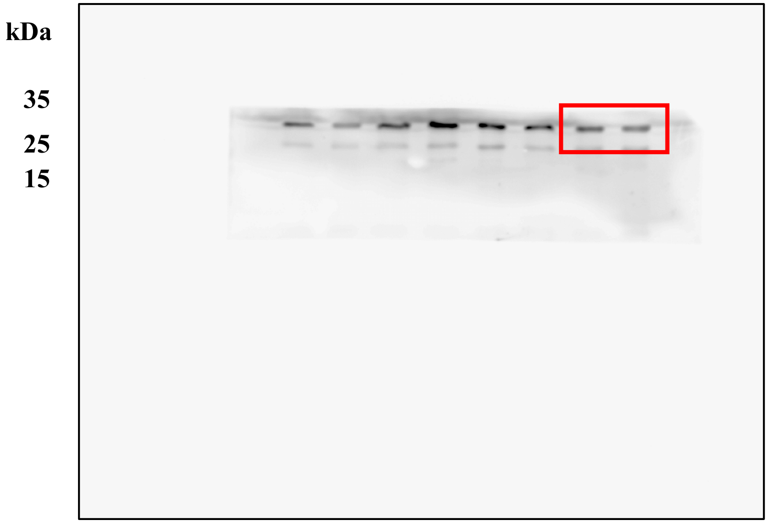

Anti-BCL-2

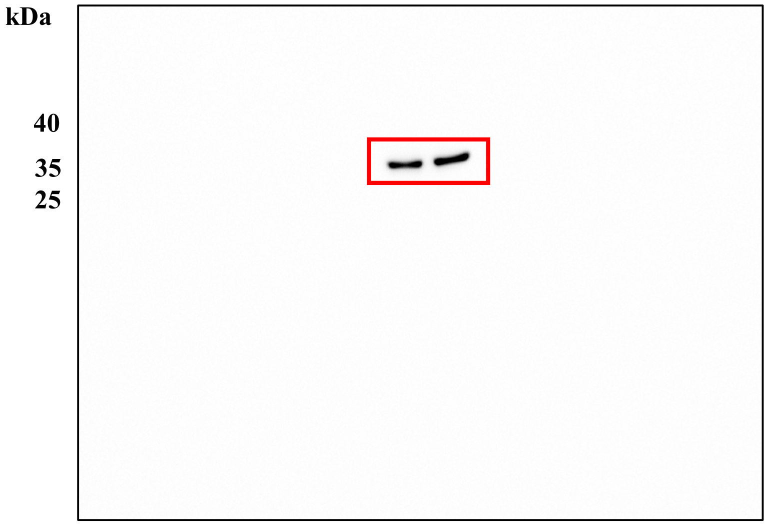

Anti-GAPDH

Sup J. Full unedited blots for Fig.5g (si#1)

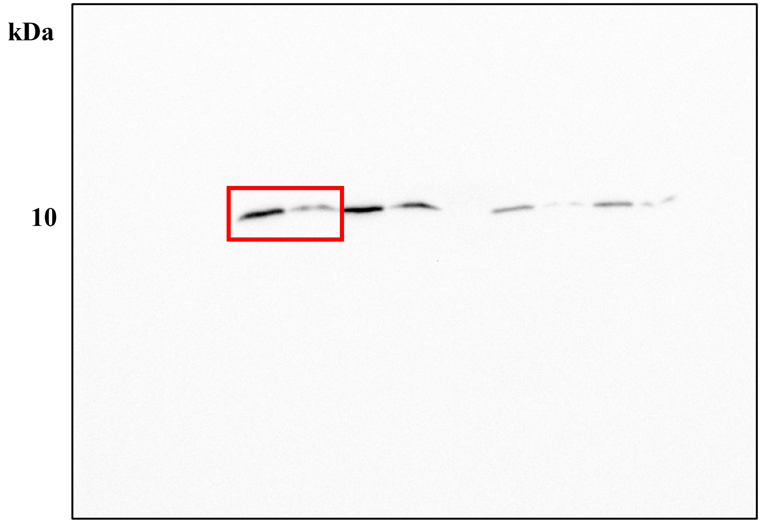

HT29 Anti-SRP9

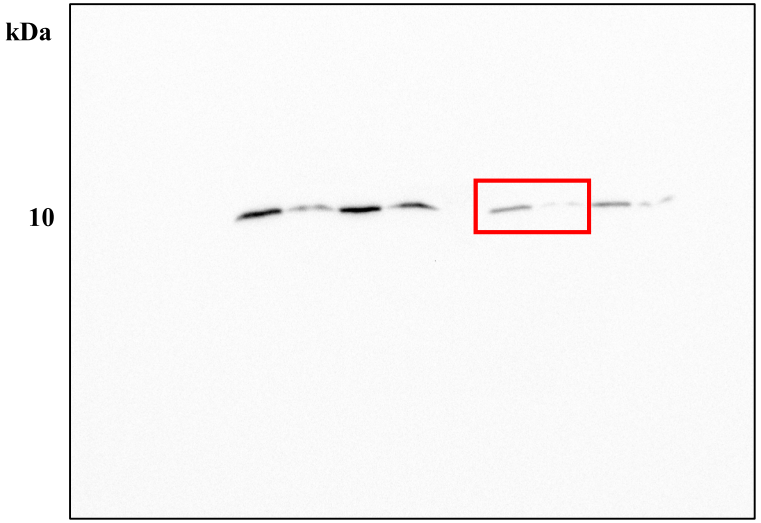

HCT116 Anti-SRP9

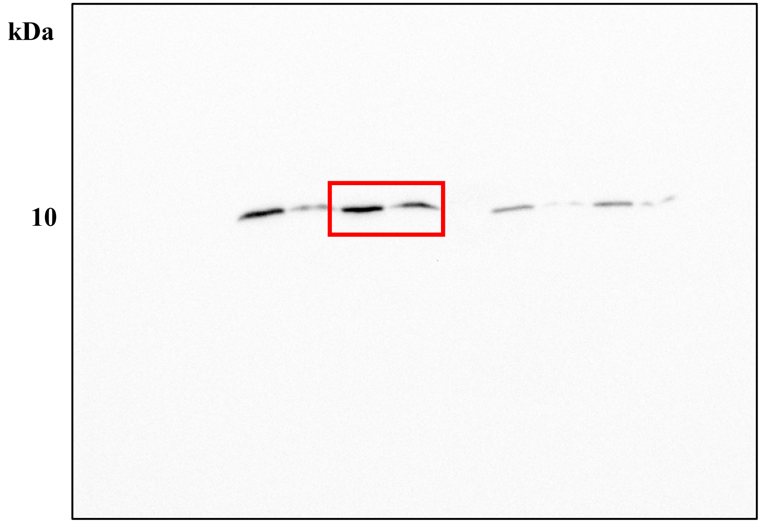

HT29 Anti-SRP9

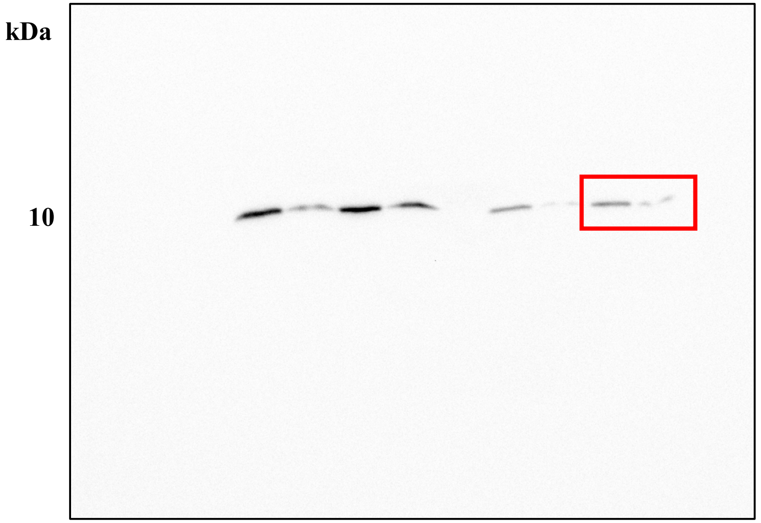

HCT116 Anti-SRP9

Sup J. Full unedited blots for Fig.5g (si#1)

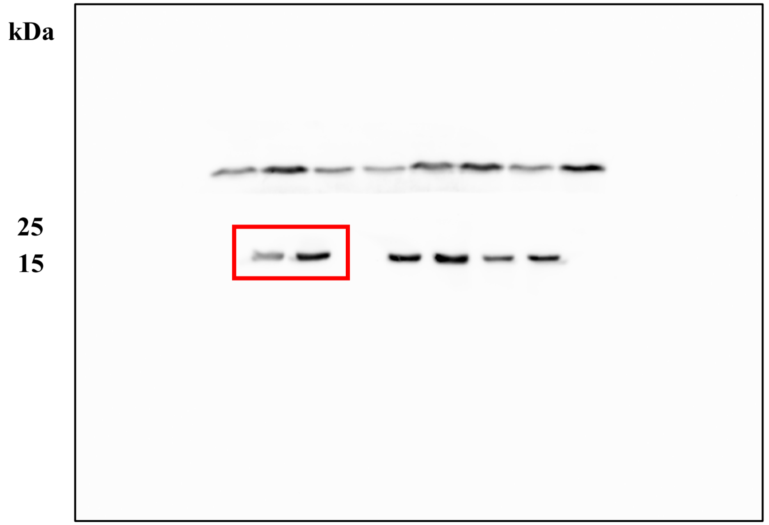

HT29 Anti-BAX

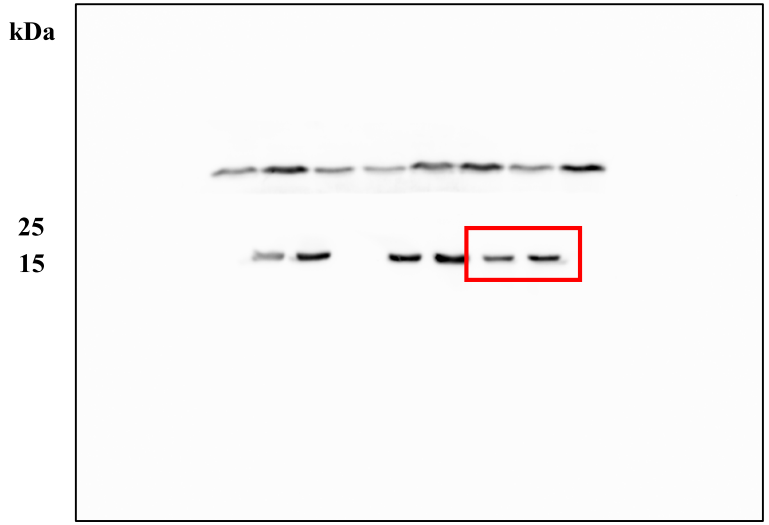

HCT116 Anti-BAX

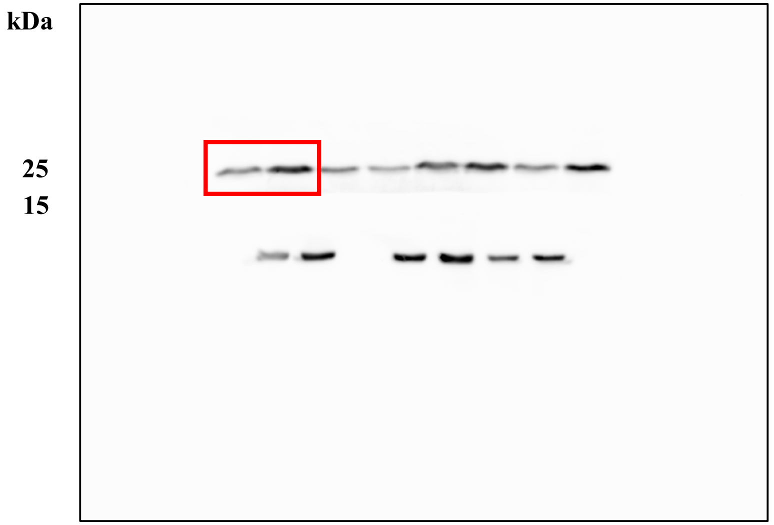

HT29 Anti-BAX

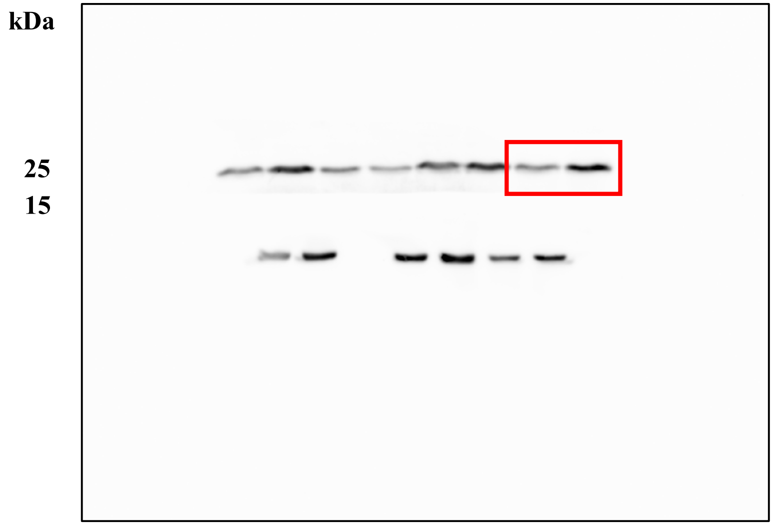

HCT116 Anti-BAX

Sup J. Full unedited blots for Fig.5g (si#1)

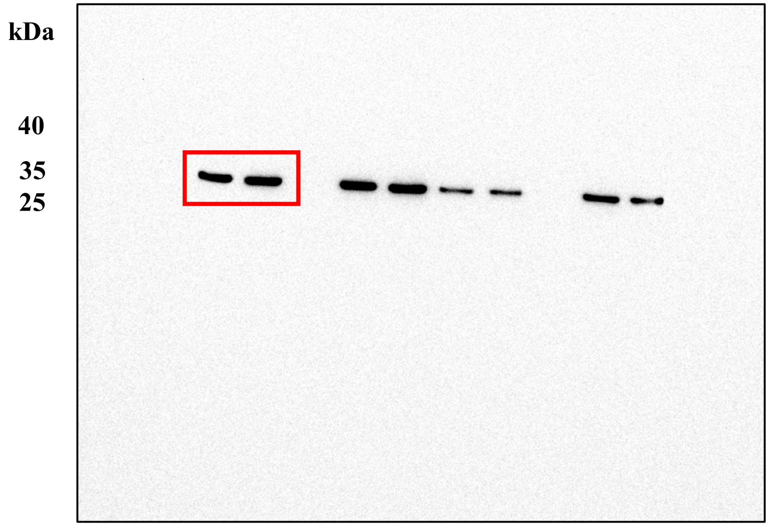

HT29 Anti-GAPDH

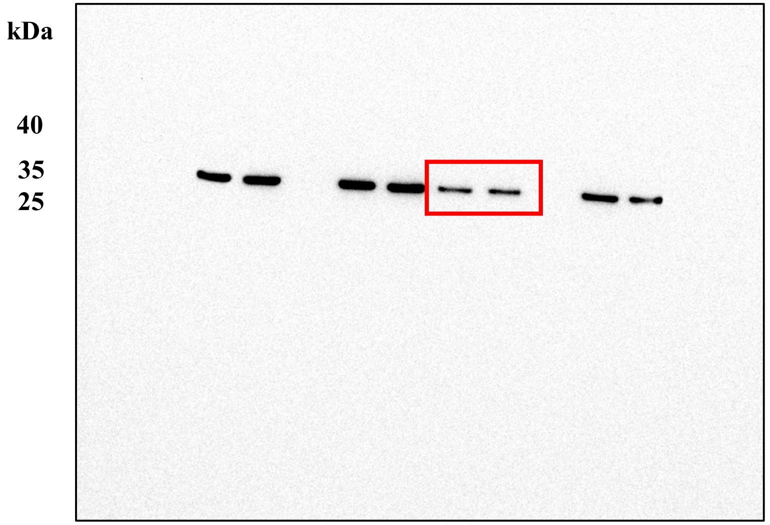

HCT116 Anti-GAPDH

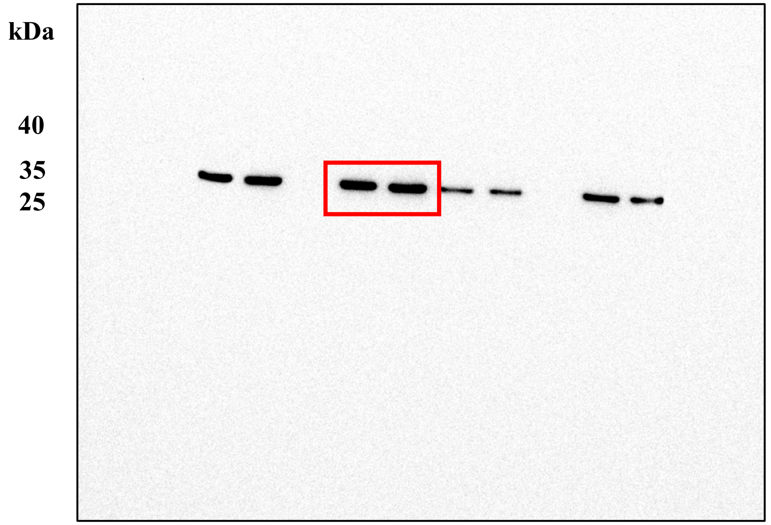

HT29 Anti-GAPDH

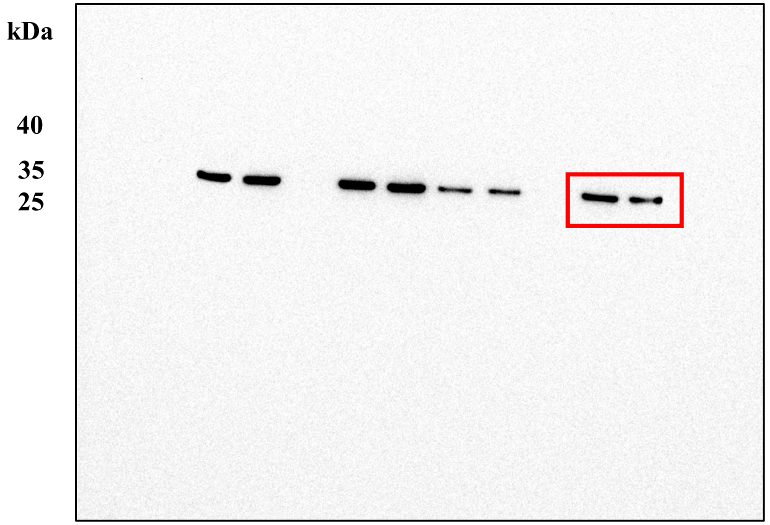

HCT116 Anti-GAPDH

J. Full unedited blots for Fig.5g HT29(si#2)

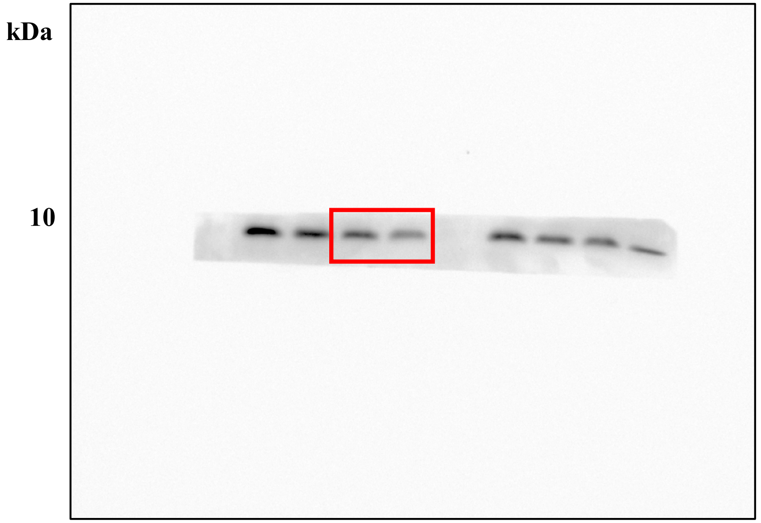

Anti-SRP9

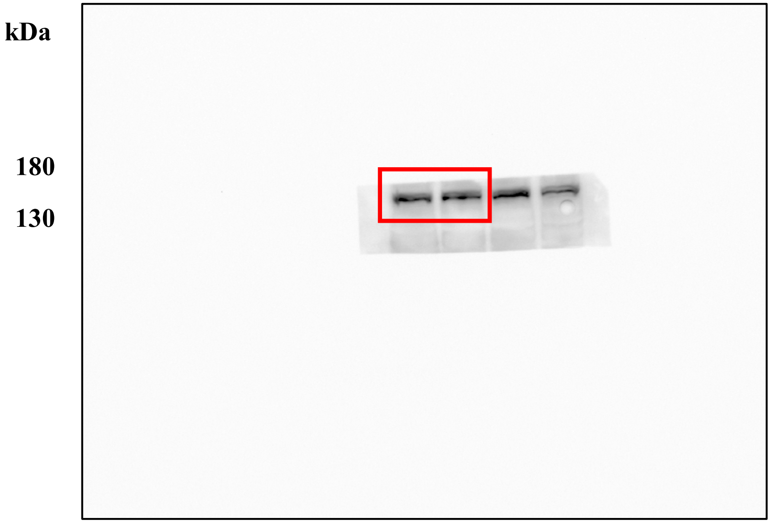

Anti-T-ASK1

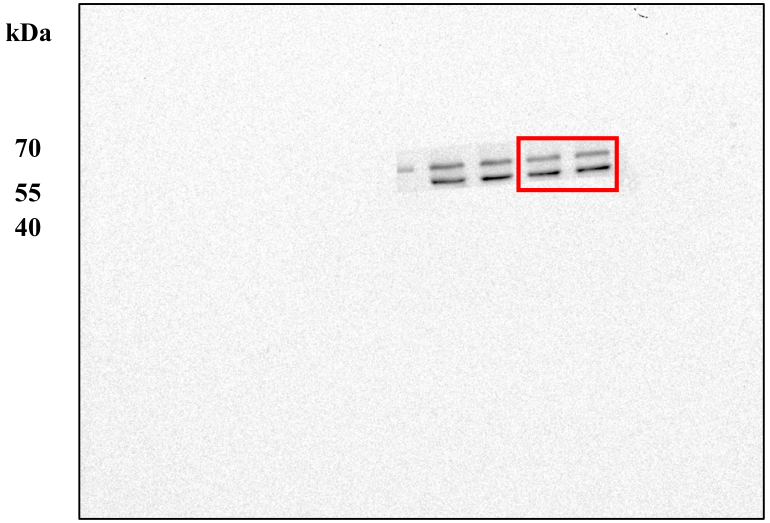

Anti-T- JNK

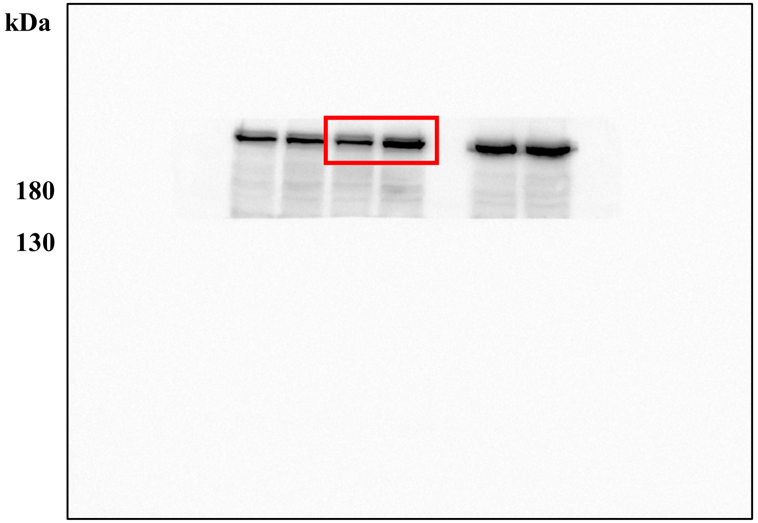

Anti-p-ASK1(Thr845)

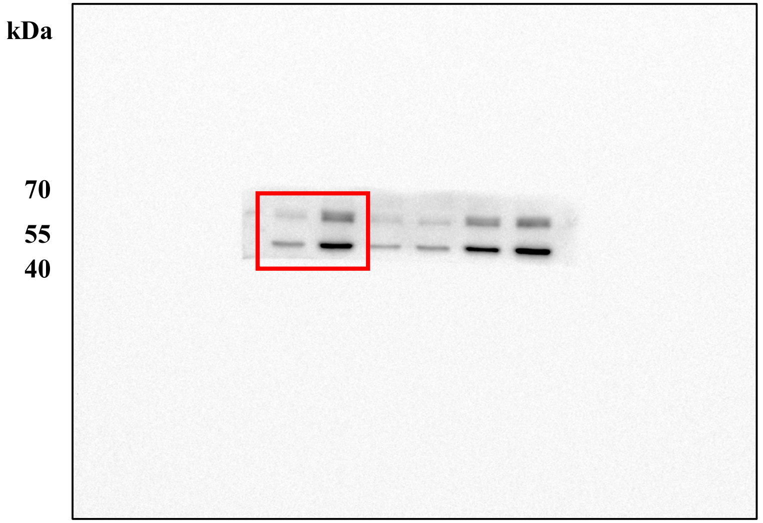

Anti-p- JNK(Thr183 / Tyr185)

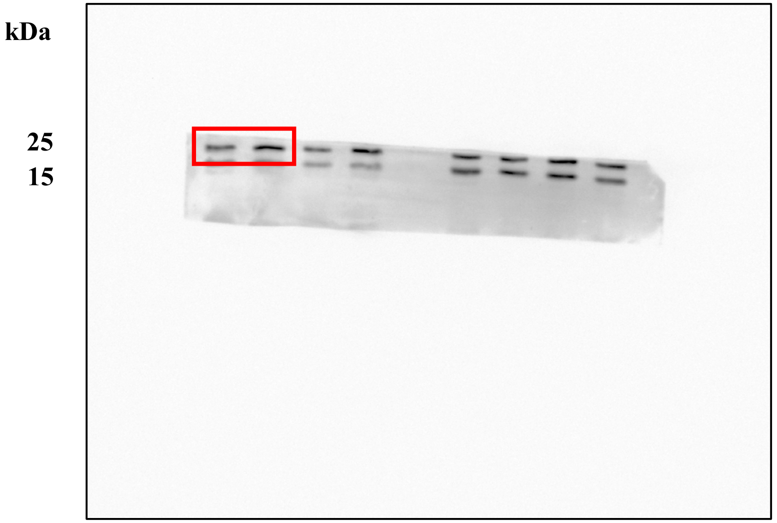

Anti-BAX

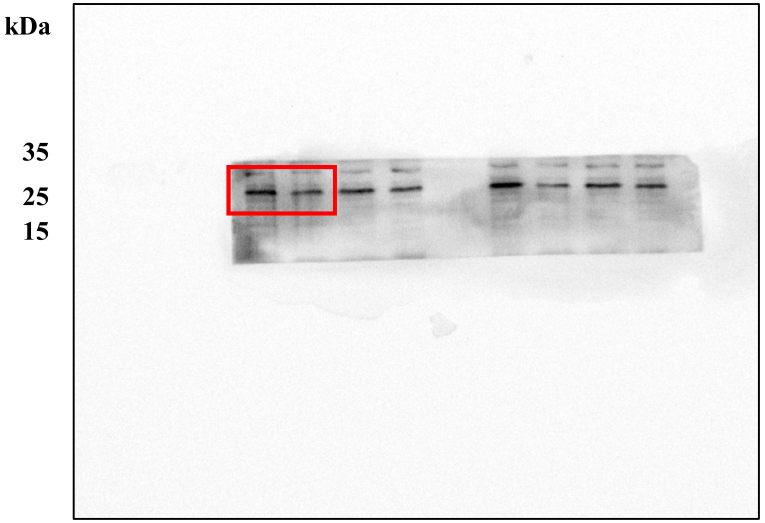

Anti-BCL-2

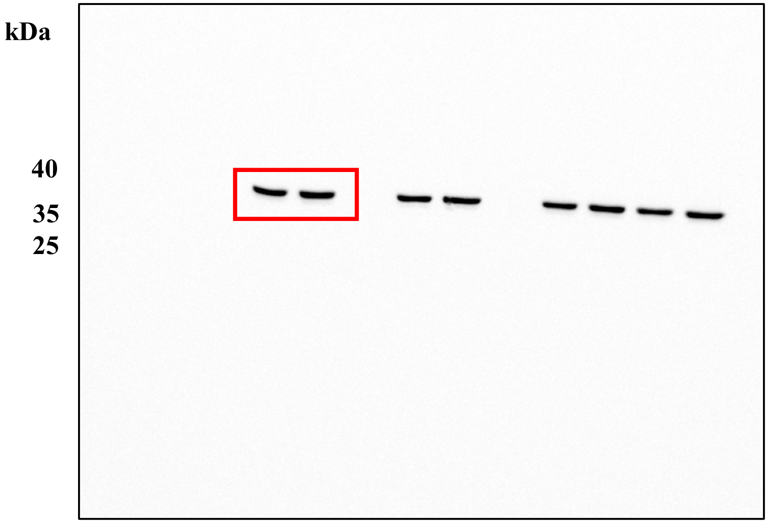

Anti-GAPDH

J. Full unedited blots for Fig.5g HCT116(si#2)

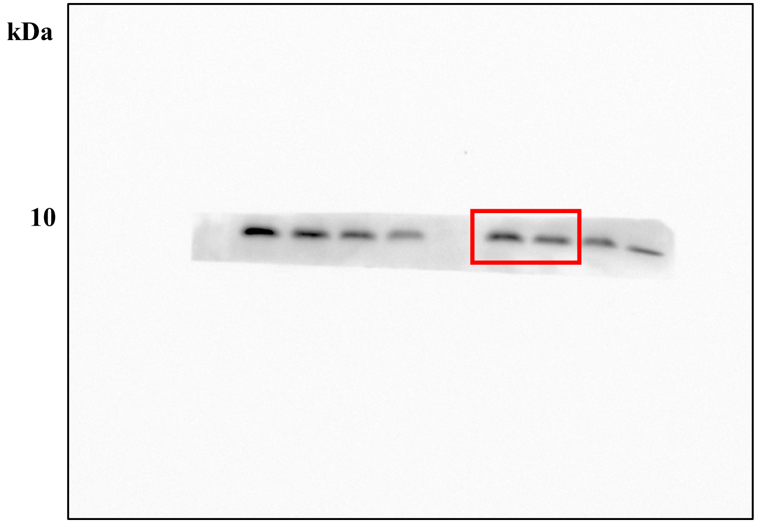

Anti-SRP9

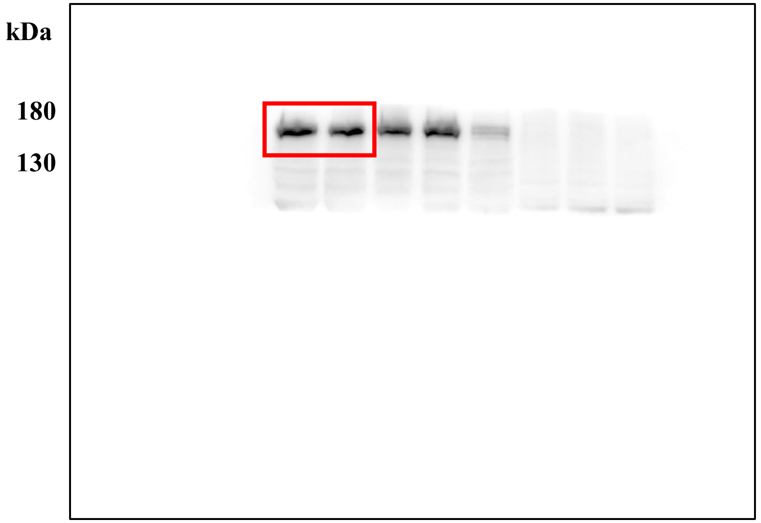

Anti-T-ASK1

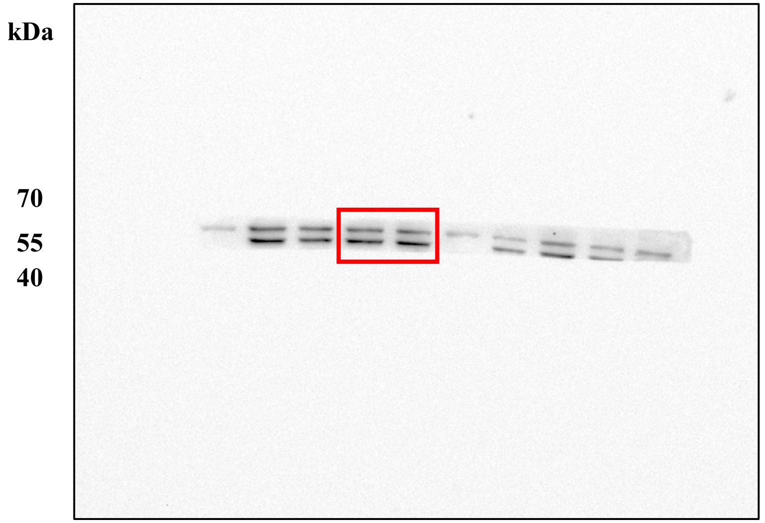

Anti-T- JNK

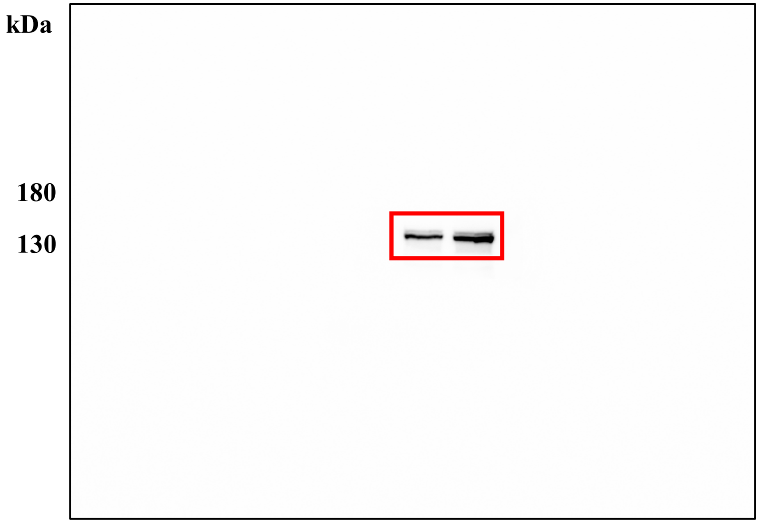

Anti-p-ASK1(Thr845)

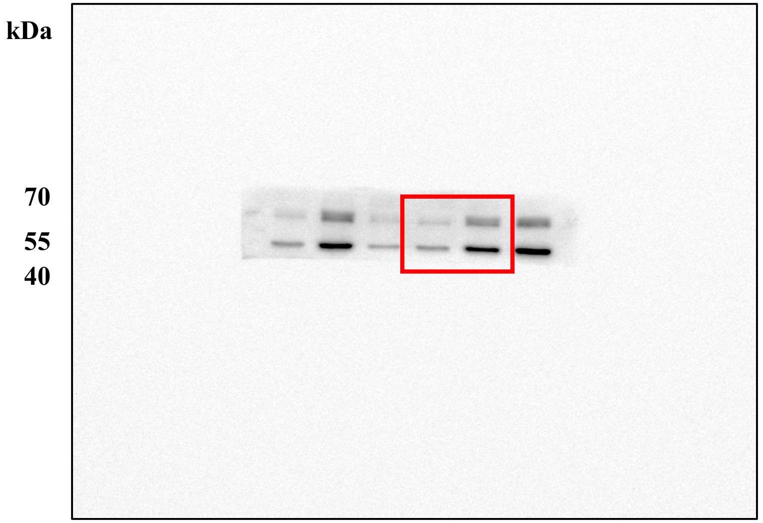

Anti-p- JNK(Thr183 / Tyr185)

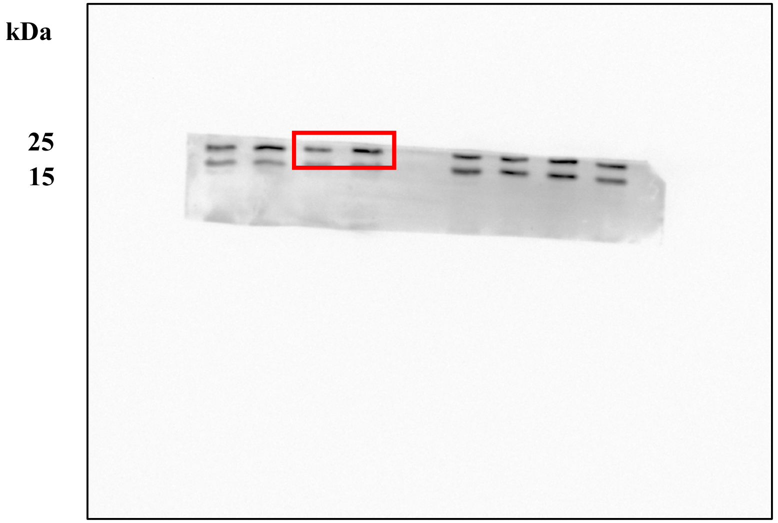

Anti-BAX

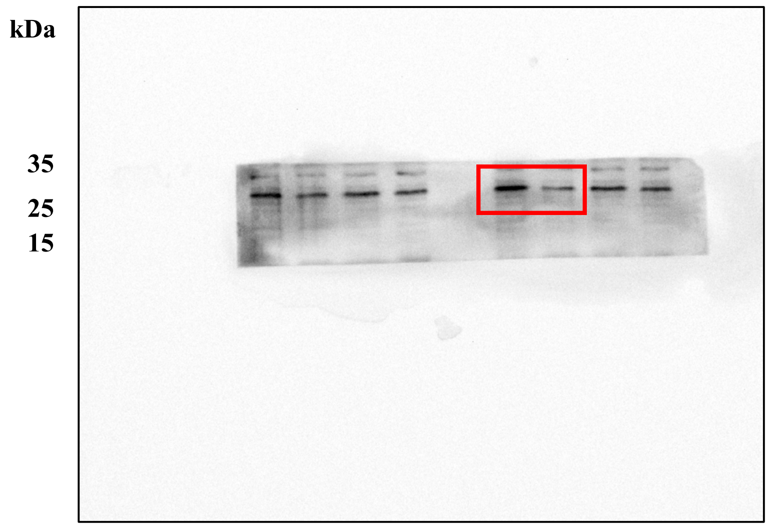

Anti-BCL-2

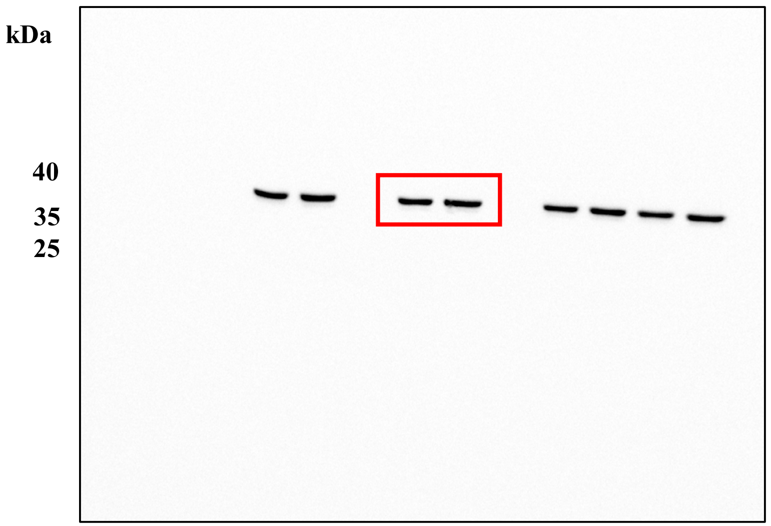

Anti-GAPDH

Sup J. Full unedited blots for Fig.5g (si#2)

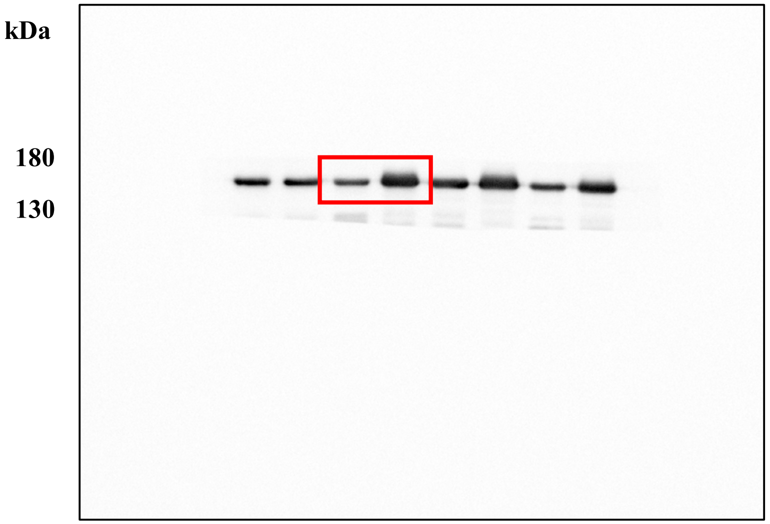

HCT116 Anti-p-ASK1(Thr845)

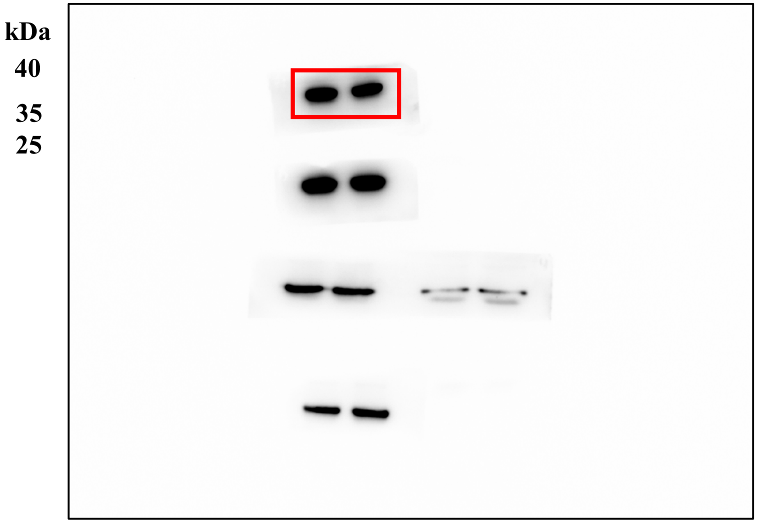

HT29 Anti-GAPDH

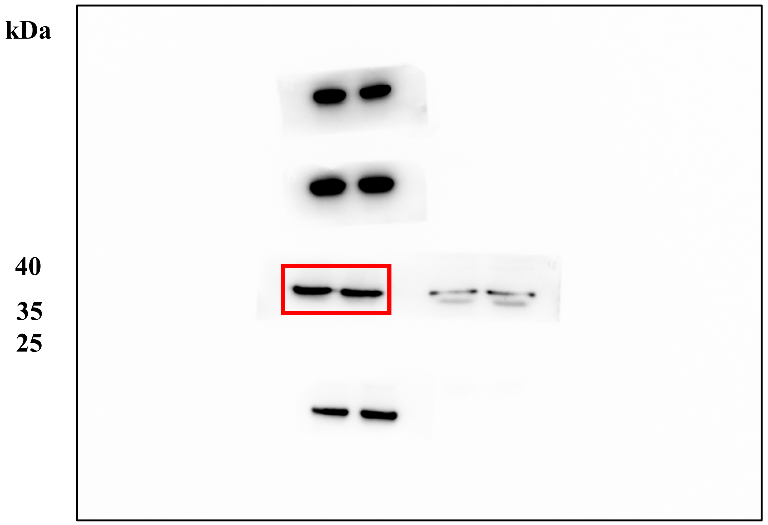

HCT116 Anti-GAPDH

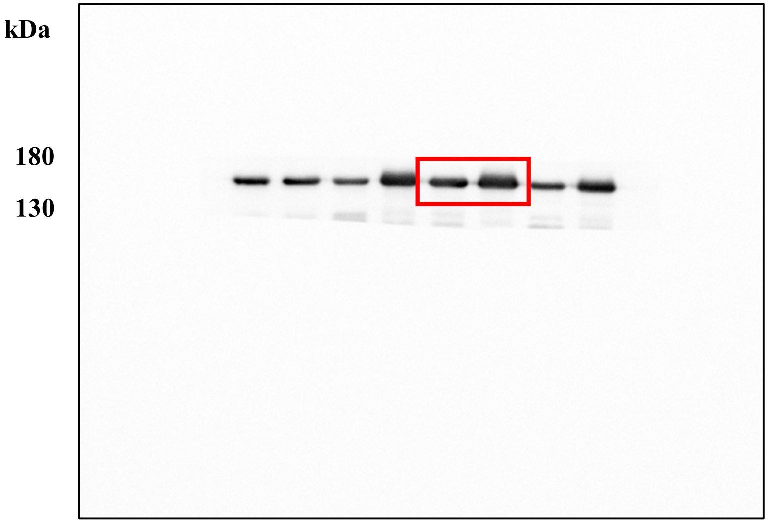

HCT116 Anti-p-ASK1(Thr845)

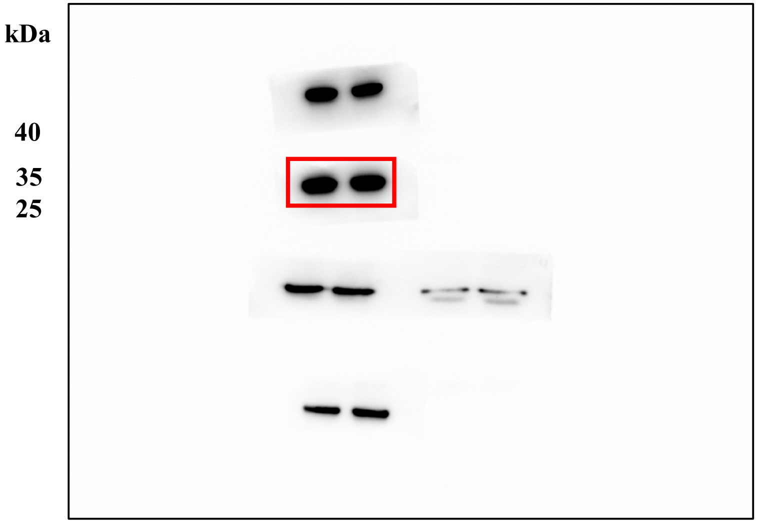

HT29 Anti-GAPDH

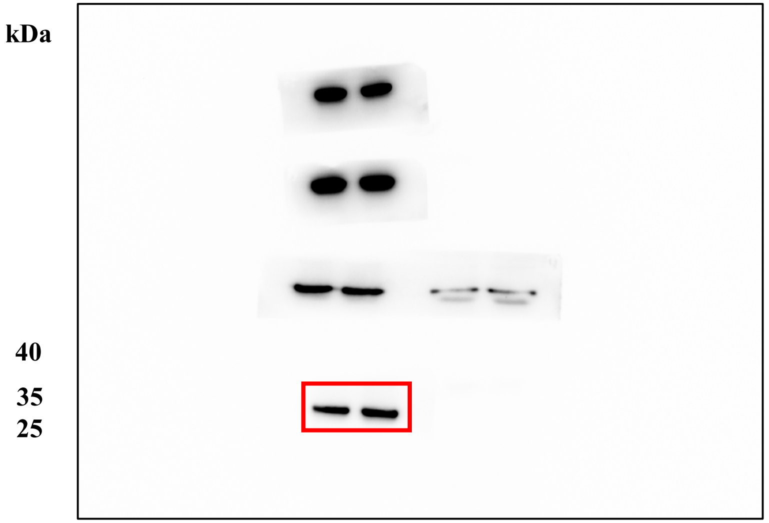

HCT116 Anti-GAPDH

K. Full unedited blots for Fig.5h HT29

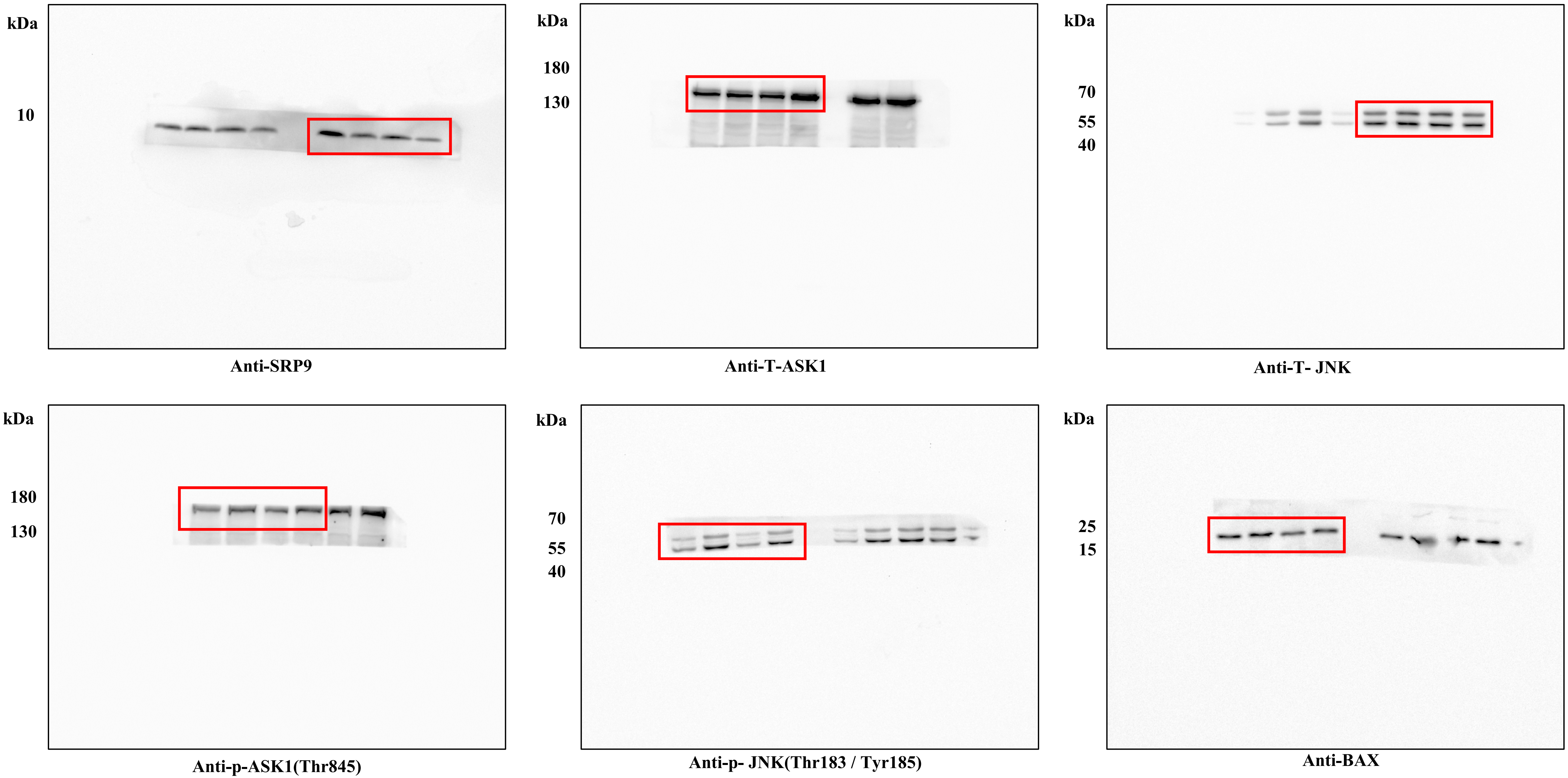

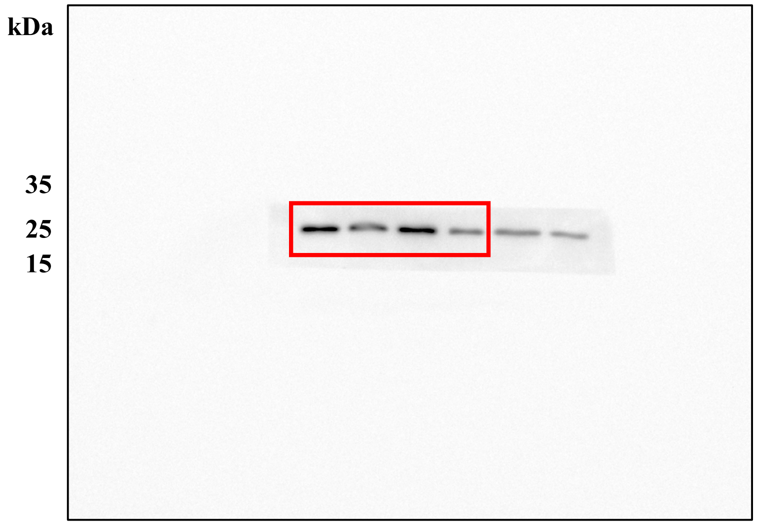

Anti-BCL-2

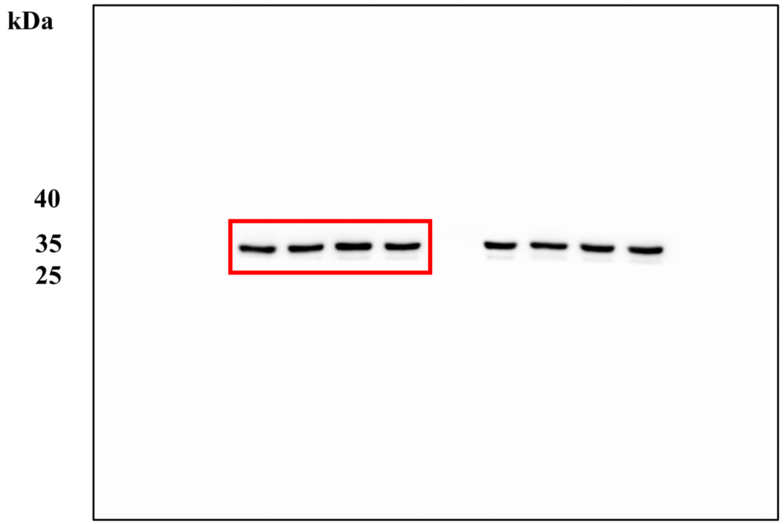

Anti-GAPDH

K. Full unedited blots for Fig.5h HCT116

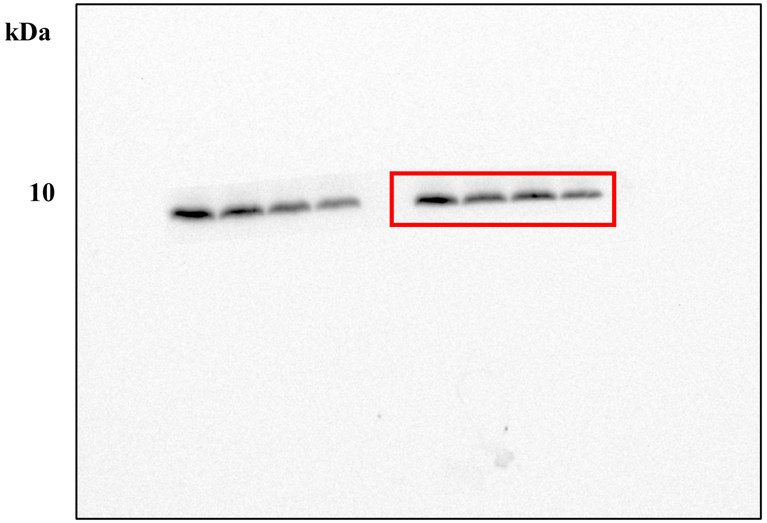

Anti-SRP9

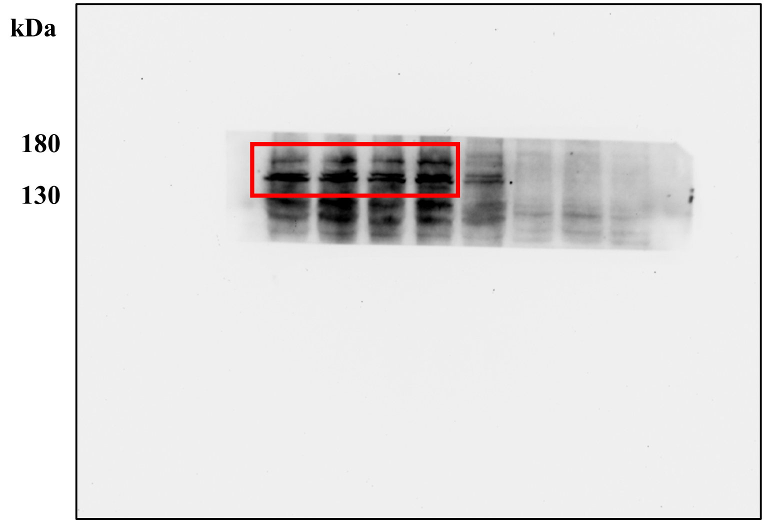

Anti-T-ASK1

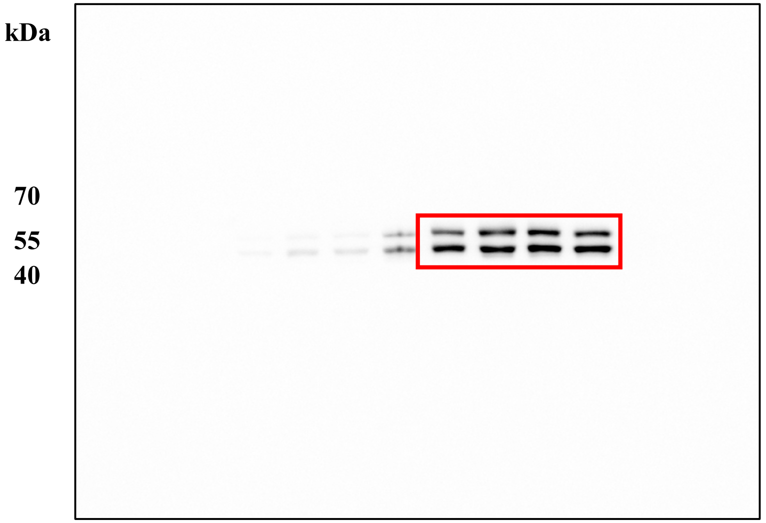

Anti-T- JNK

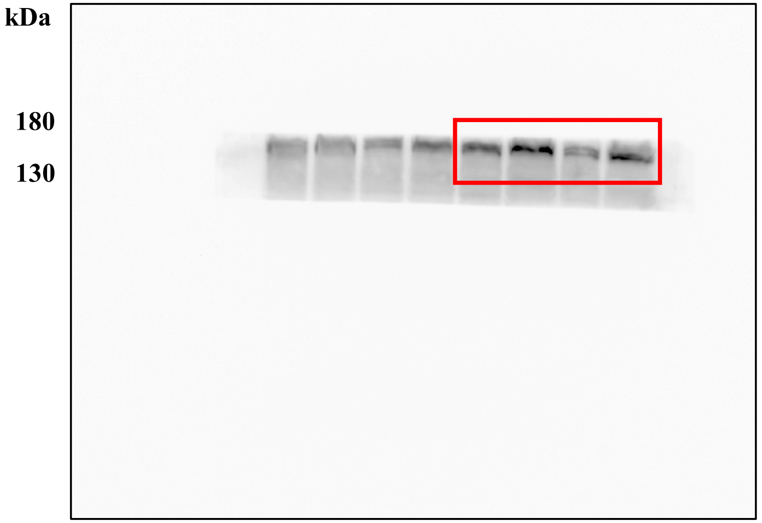

Anti-p-ASK1(Thr845)

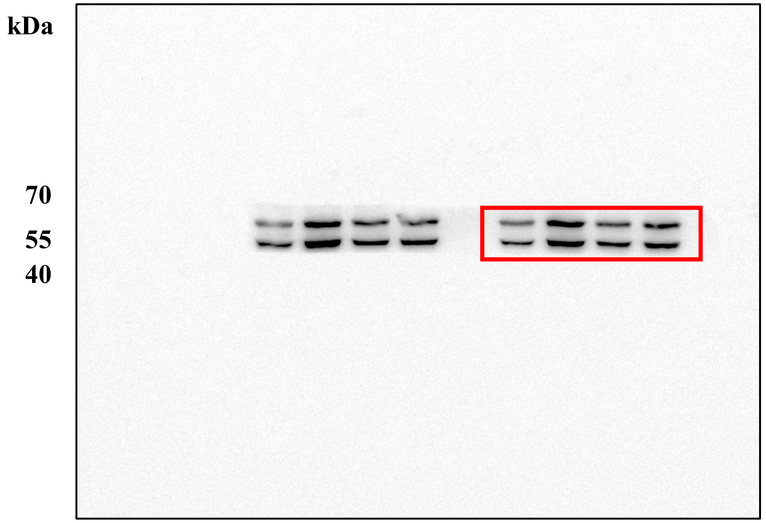

Anti-p- JNK(Thr183 / Tyr185)

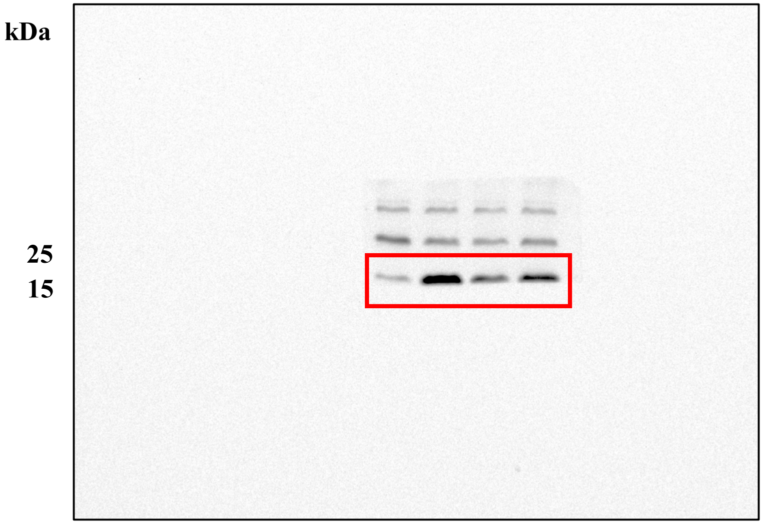

Anti-BAX

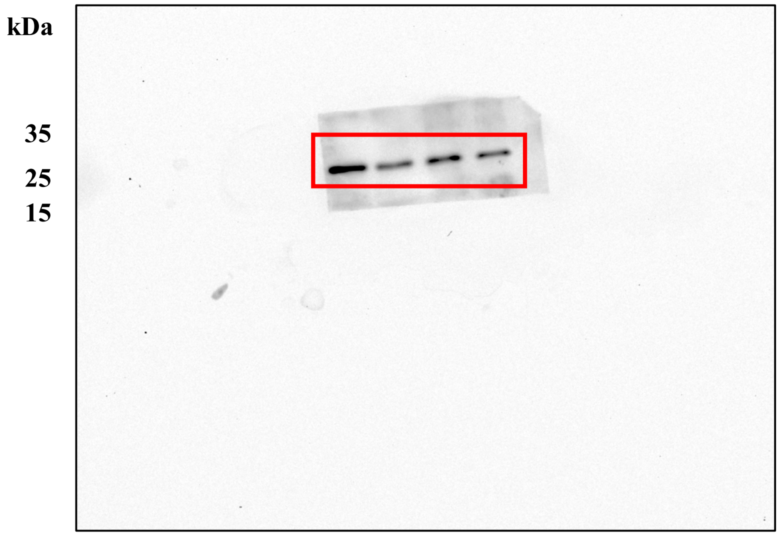

Anti-BCL-2

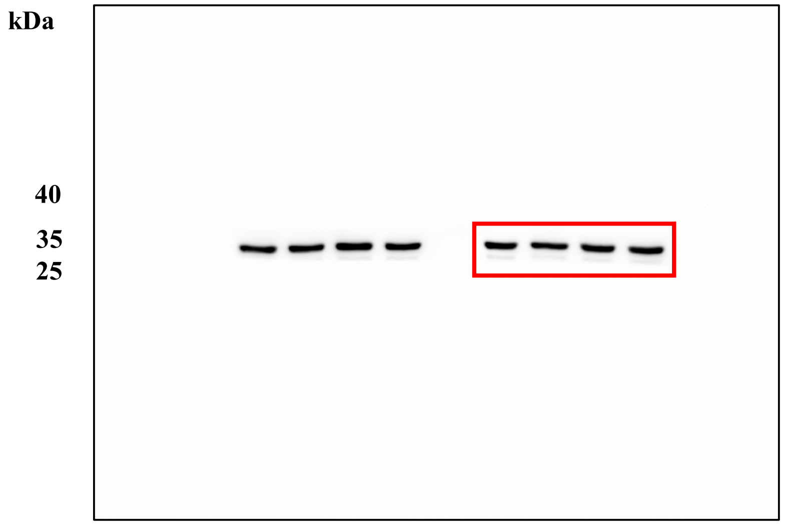

Anti-GAPDH

Sup K. Full unedited blots for Fig.5h

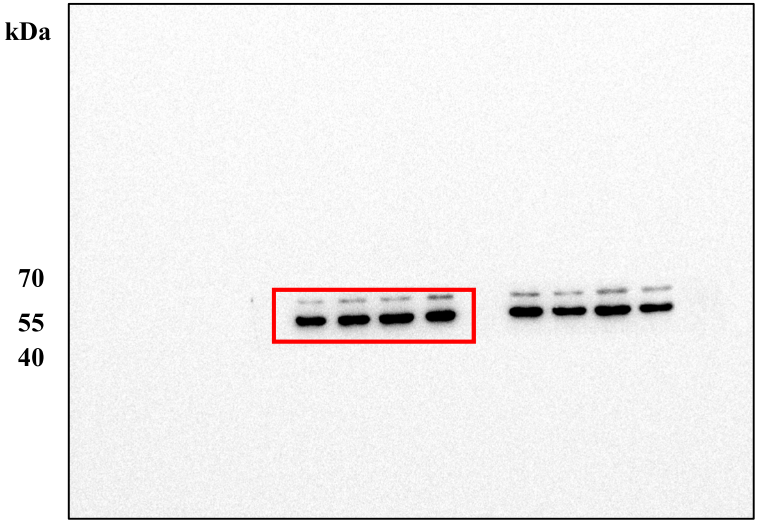

HT29 Anti-T-JNK

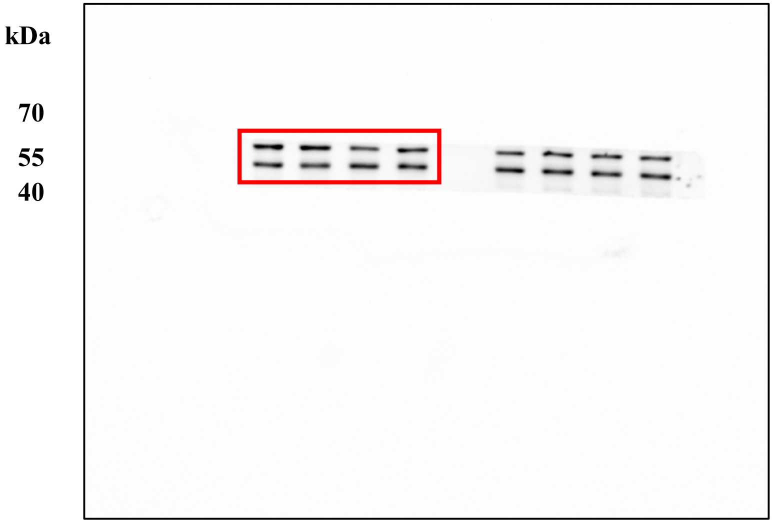

HCT116 Anti-T-JNK

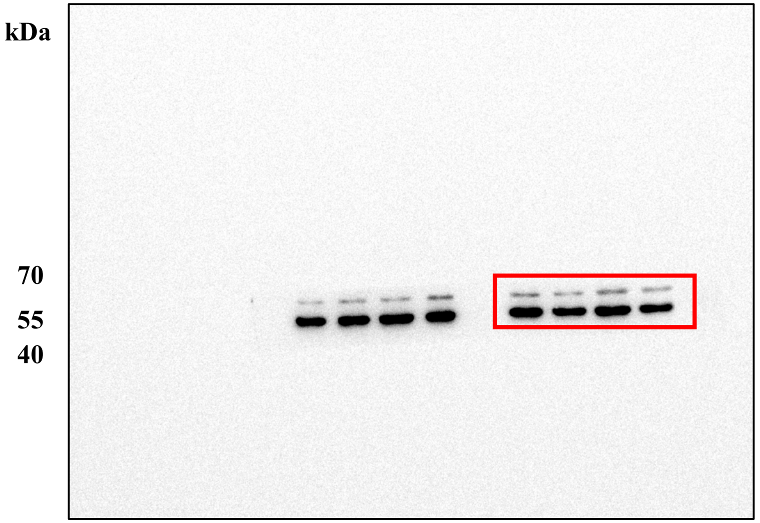

HT29 Anti-T-JNK

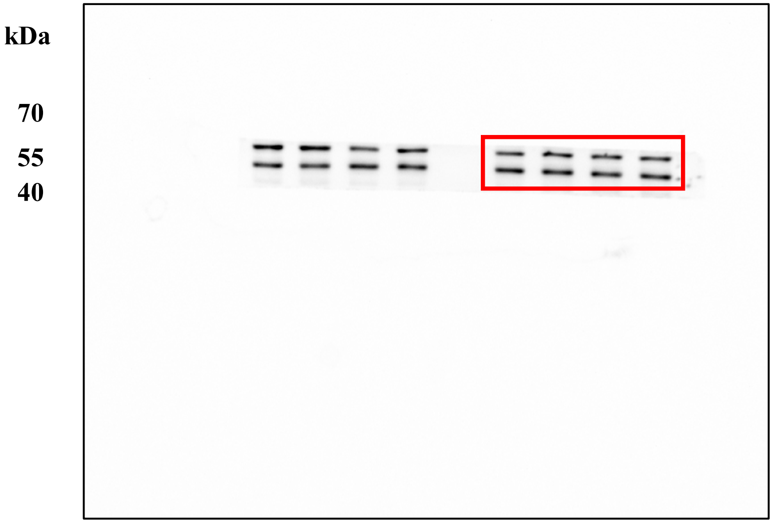

HCT116 Anti-T-JNK

Sup K. Full unedited blots for Fig.5h

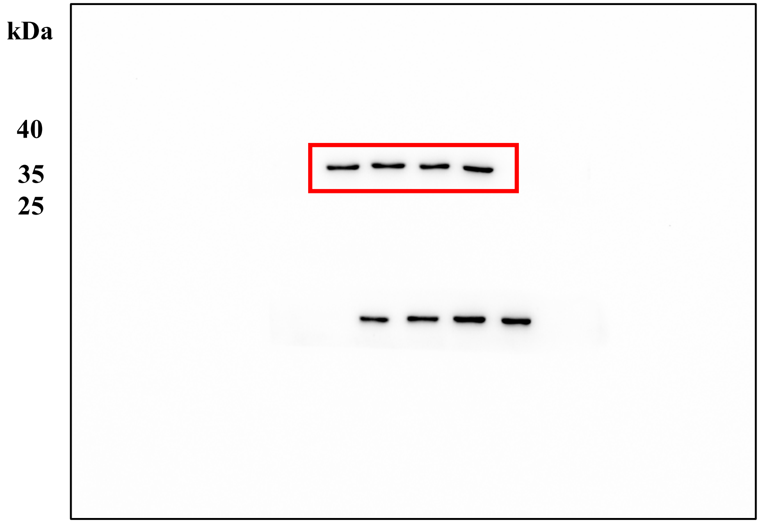

HT29 Anti-GAPDH

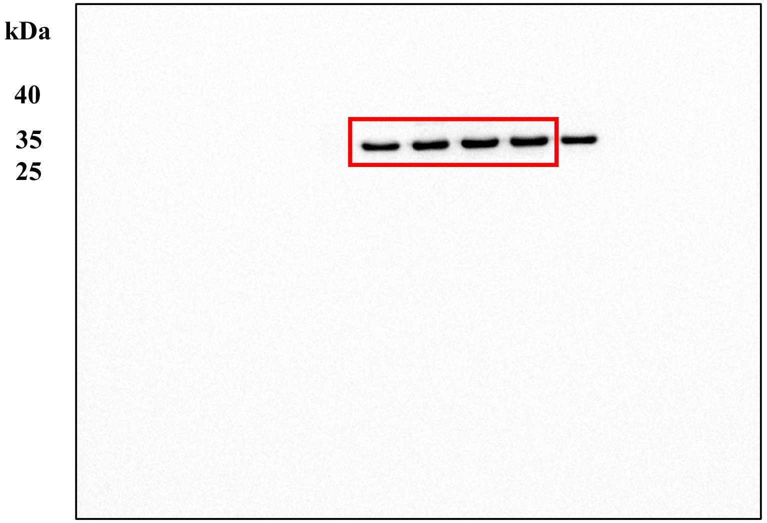

HCT116 Anti-GAPDH

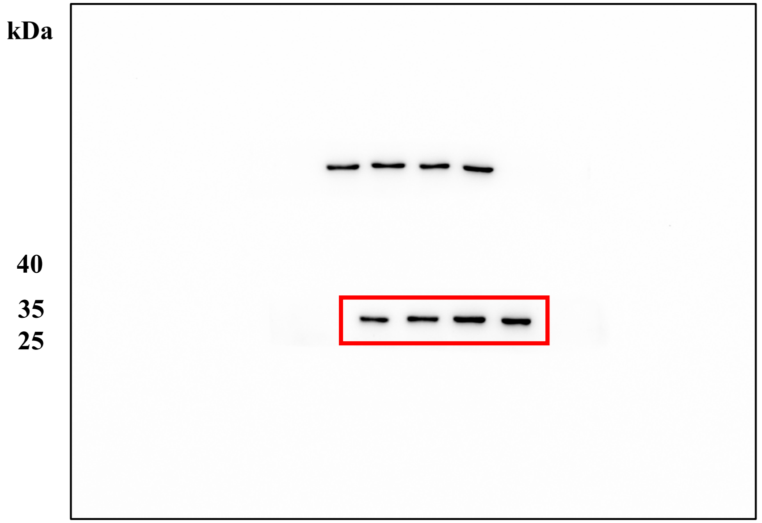

HT29 Anti-GAPDH

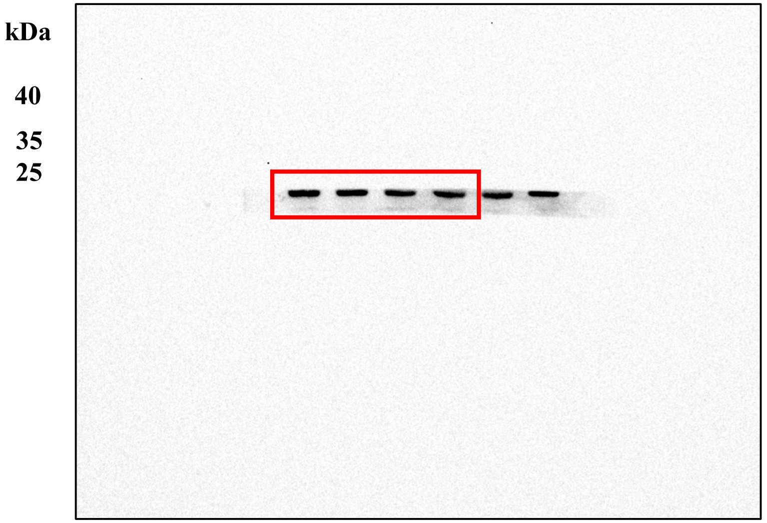

HCT116 Anti-GAPDH

L. Full unedited blots for Fig.6d

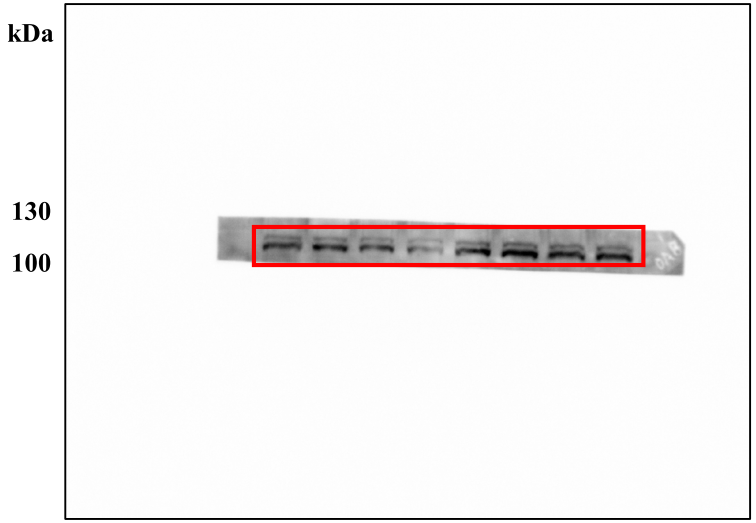

Anti-DAB2IP

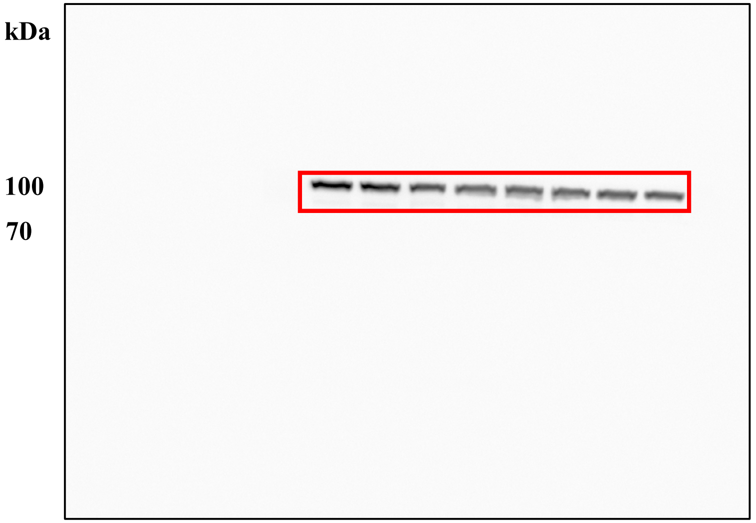

Anti-HSP90AA1

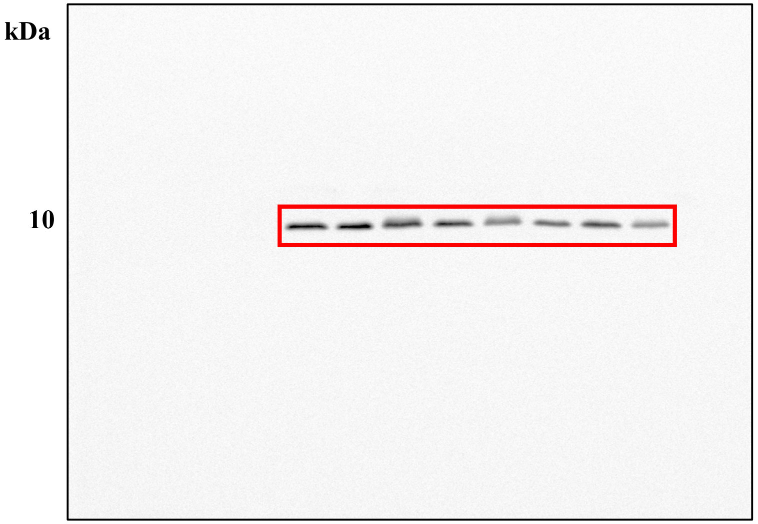

Anti-SRP9

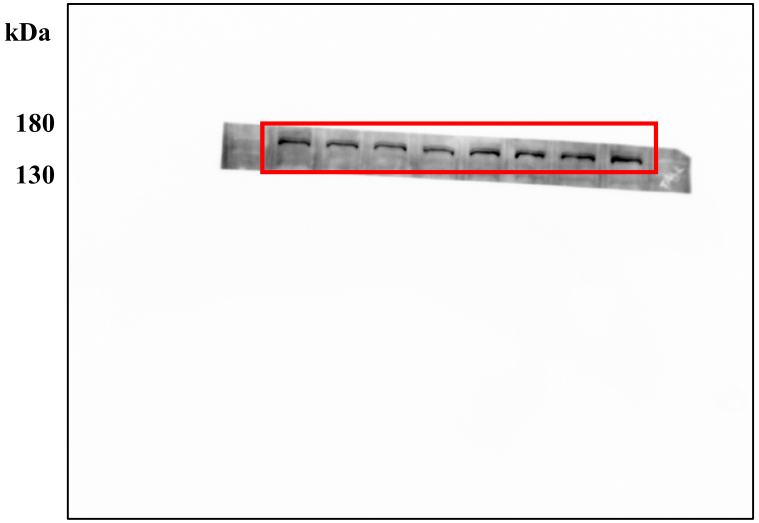

Anti-p-ASK1(Thr845)

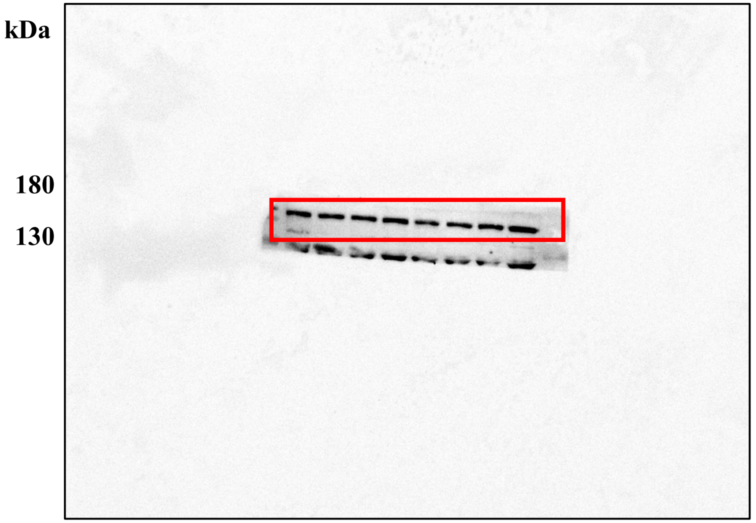

Anti-T-ASK1

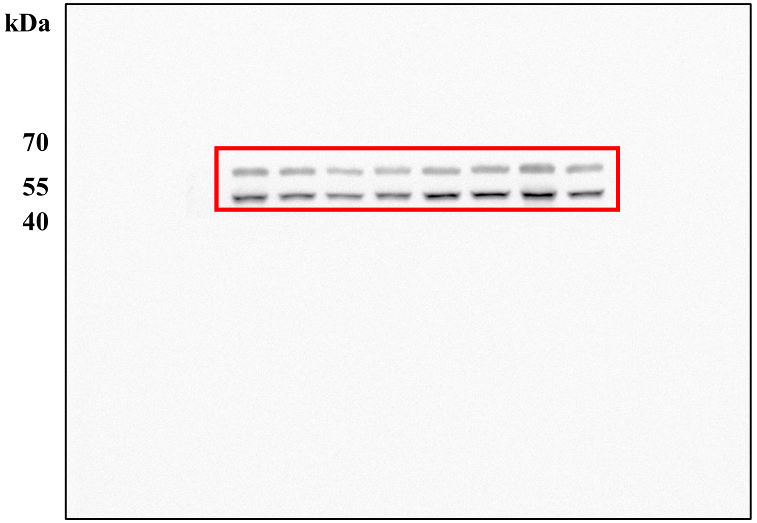

Anti-p- JNK(Thr183 / Tyr185)

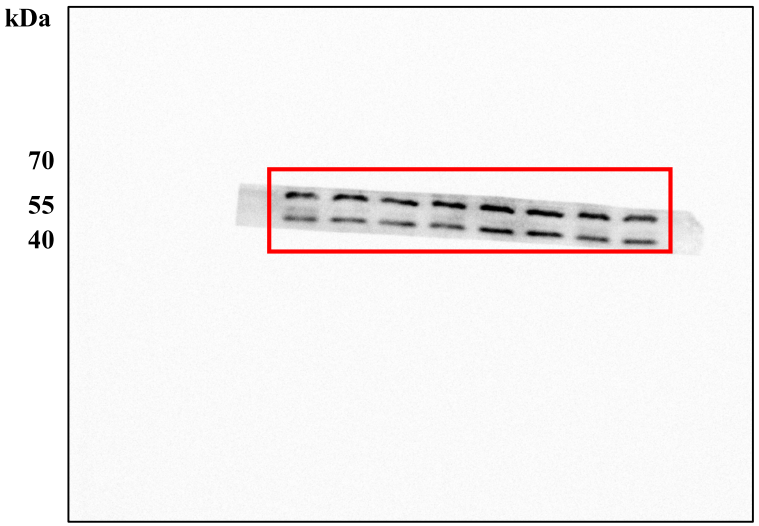

Anti-T- JNK

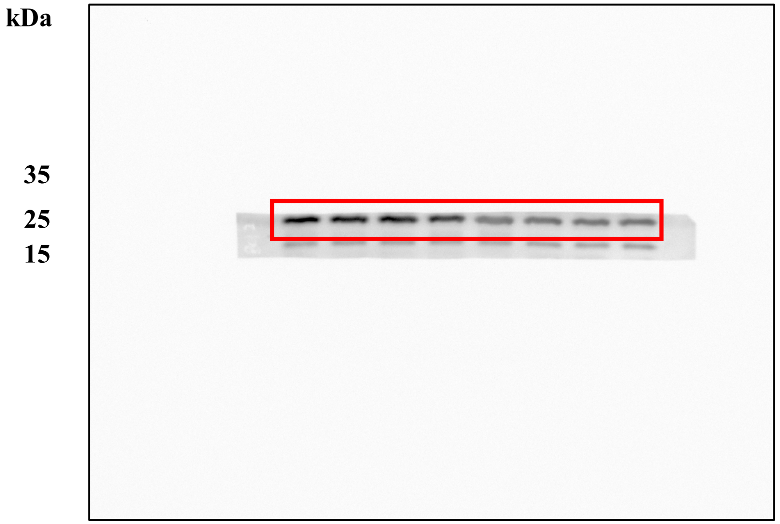

Anti-BCL-2

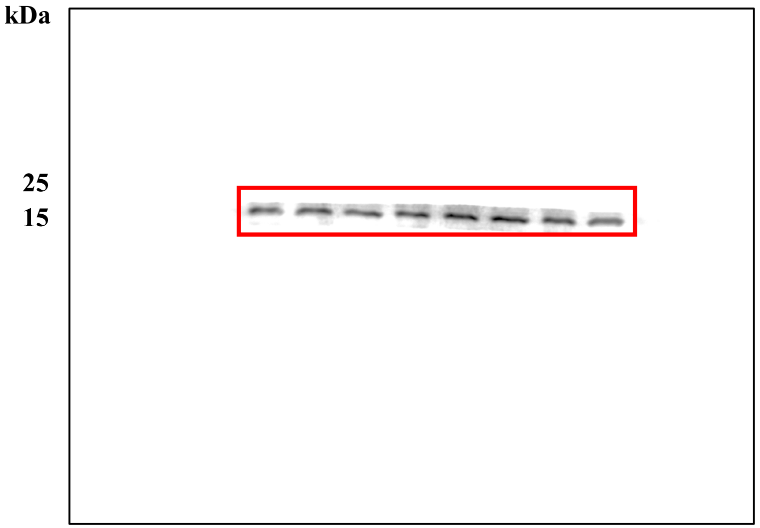

Anti-BAX

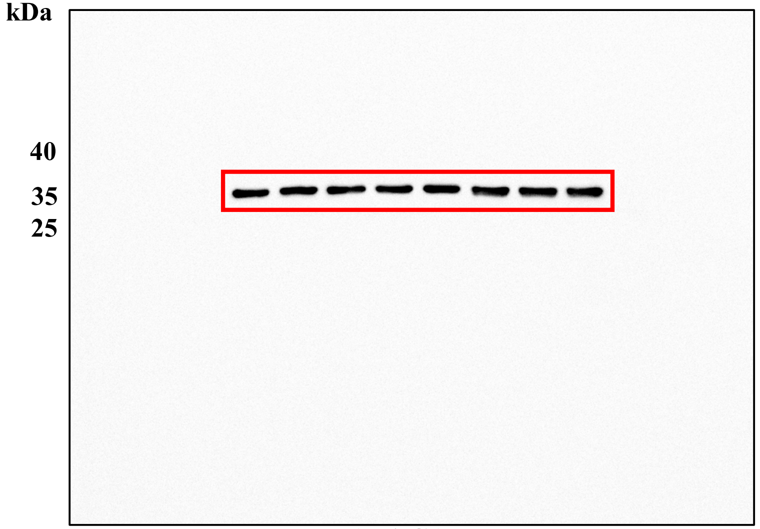

Anti-GAPDH

M. Full unedited blots for Fig.S2

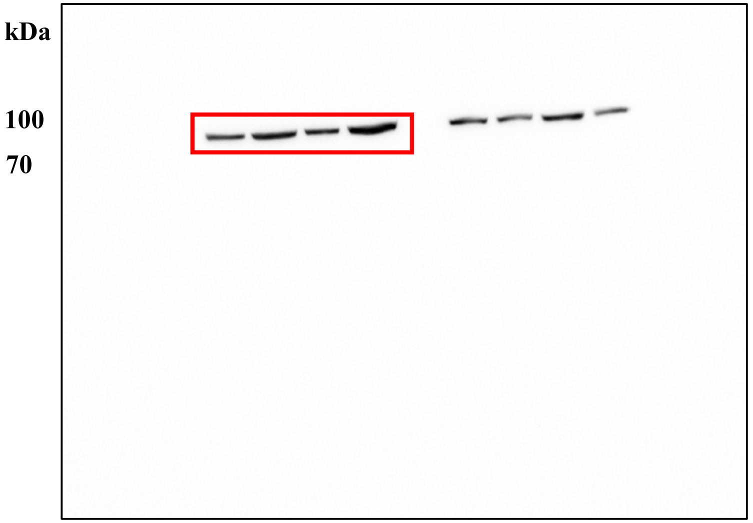

Anti-HSP90AA1

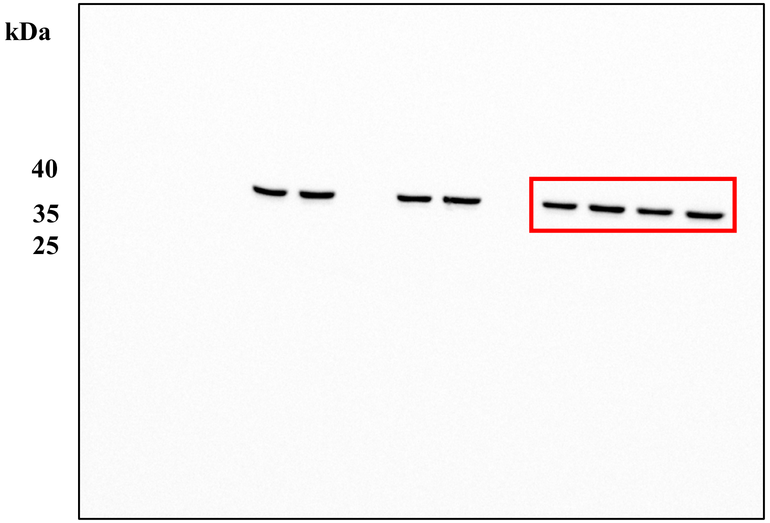

Anti-GAPDH

Sup M. Full unedited blots for Fig.S2

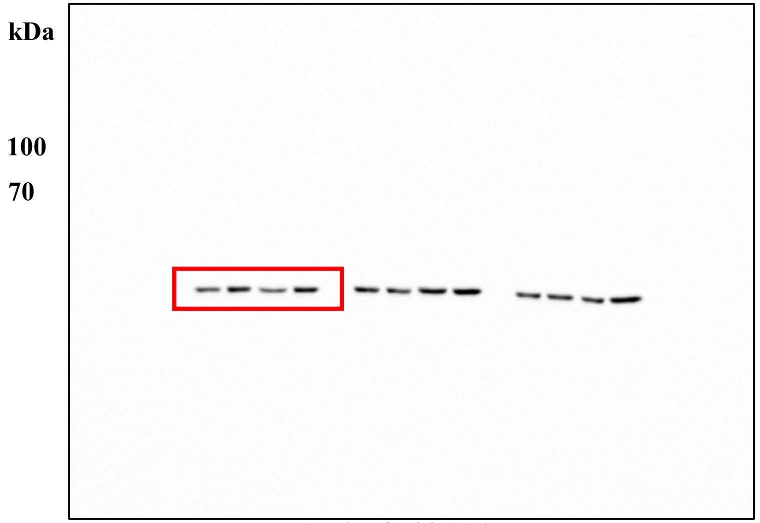

Anti-HSP90AA1

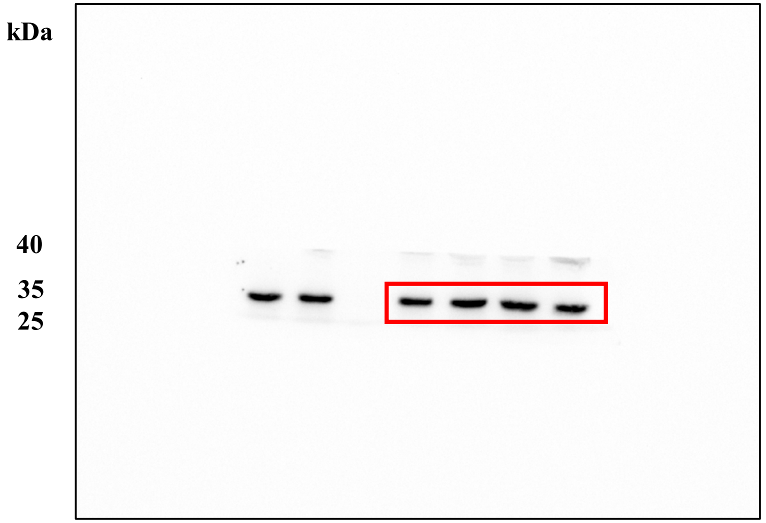

Anti-GAPDH

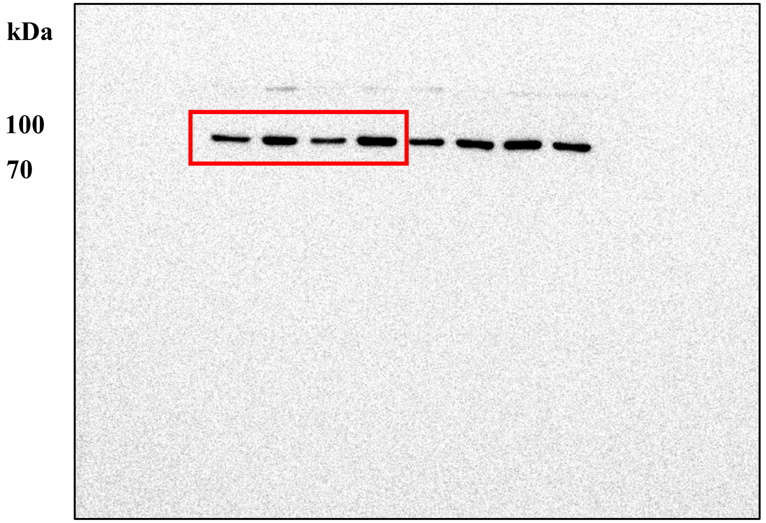

Anti-HSP90AA1

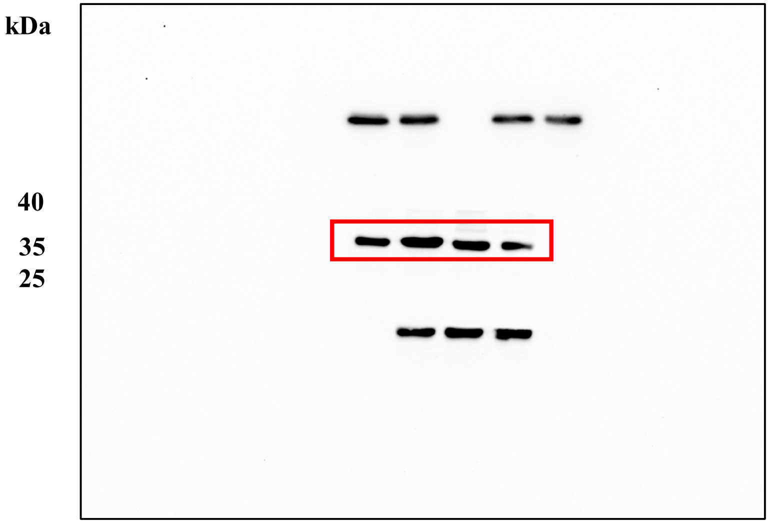

Anti-GAPDH

N. Full unedited blots for Fig.S5

HT29

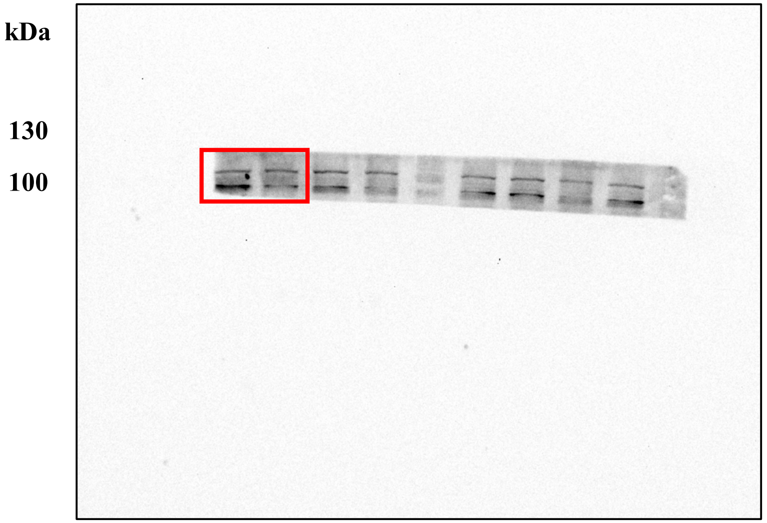

Anti-DAB2IP

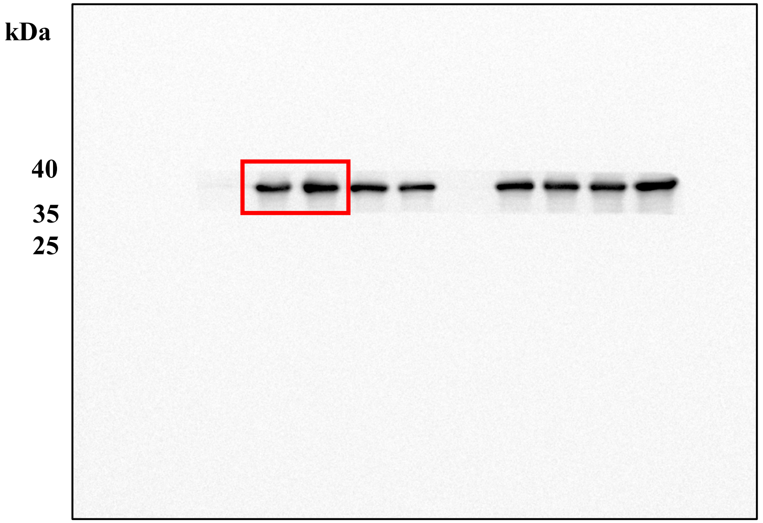

Anti-GAPDH

HCT116

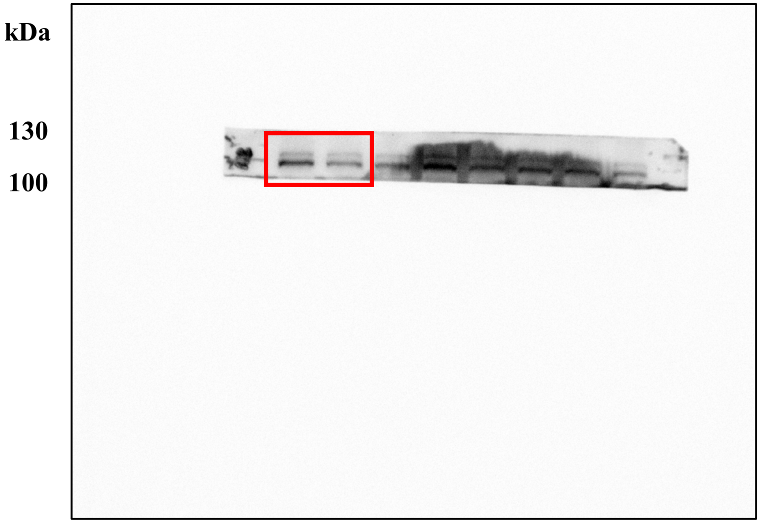

Anti-DAB2IP

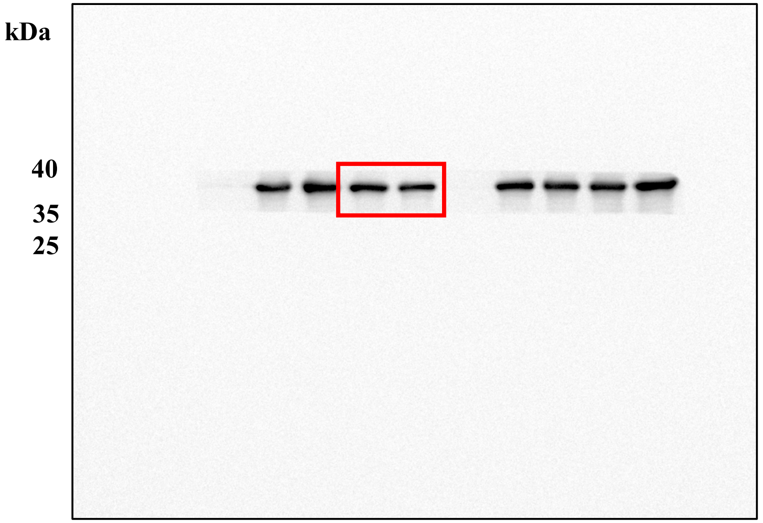

Anti-GAPDH

HT29

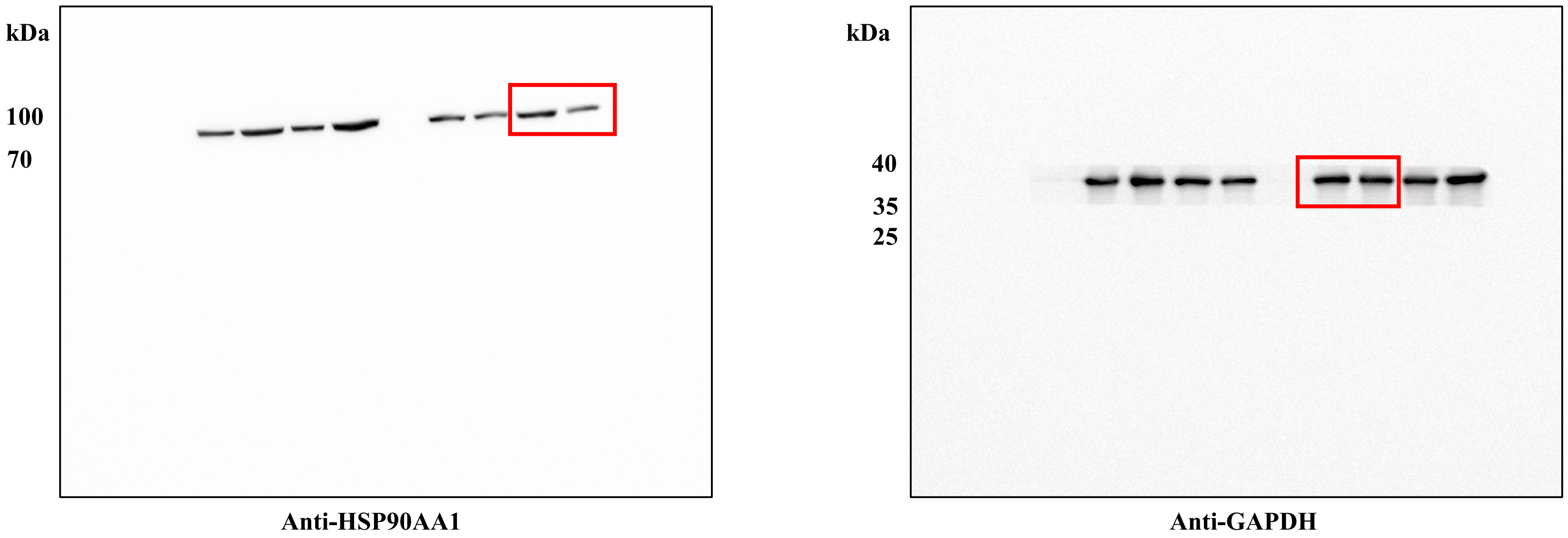
kDa
100
70
kDa
40
35
25
Anti-HSP90AA1
Anti-GAPDH

HCT116

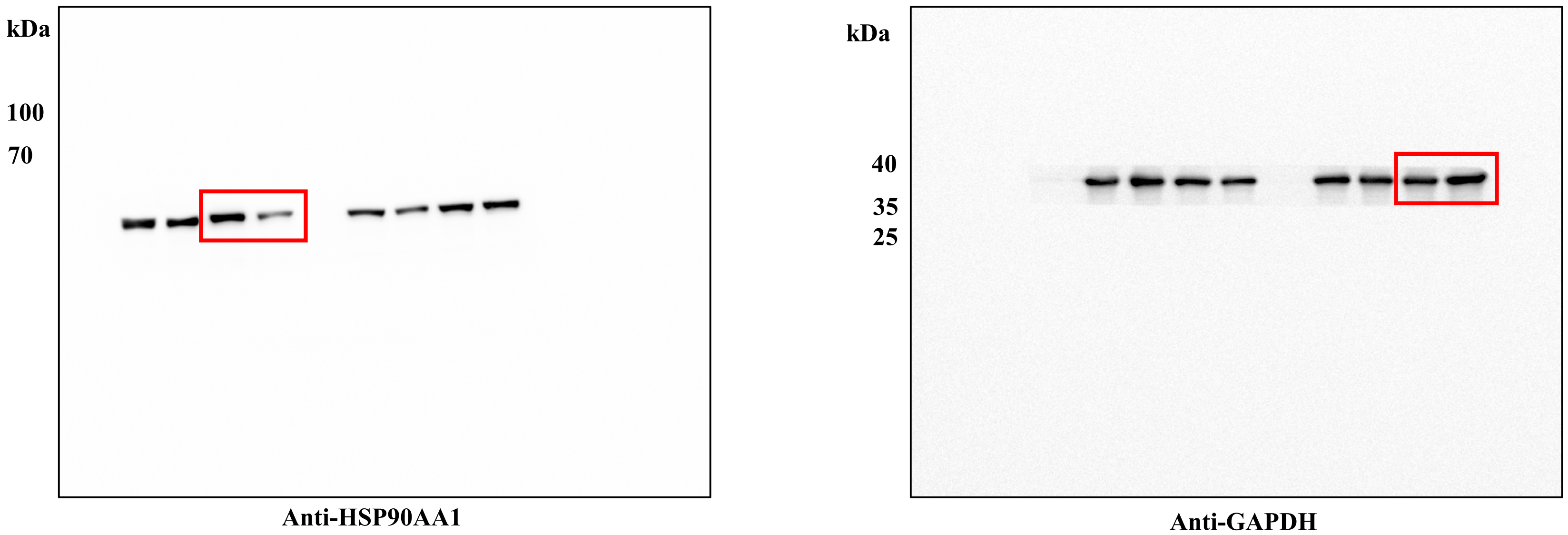
kDa
100
70
kDa
40
35
25
Anti-HSP90AA1
Anti-GAPDH

HT29

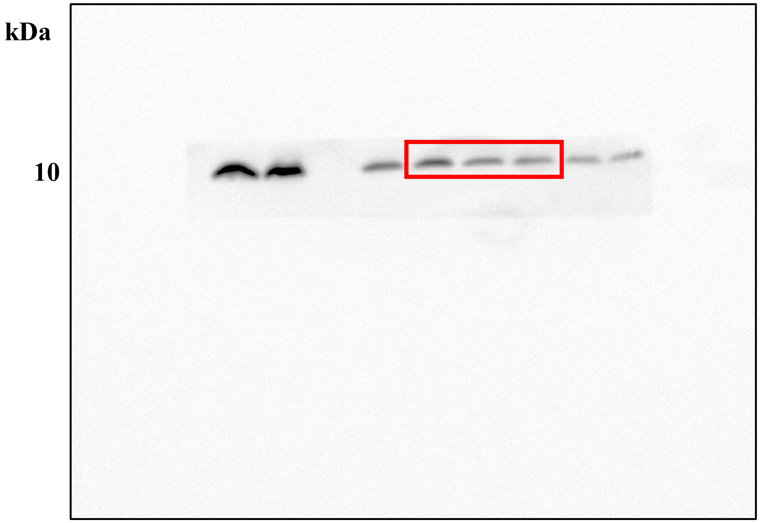

Anti-SRP9

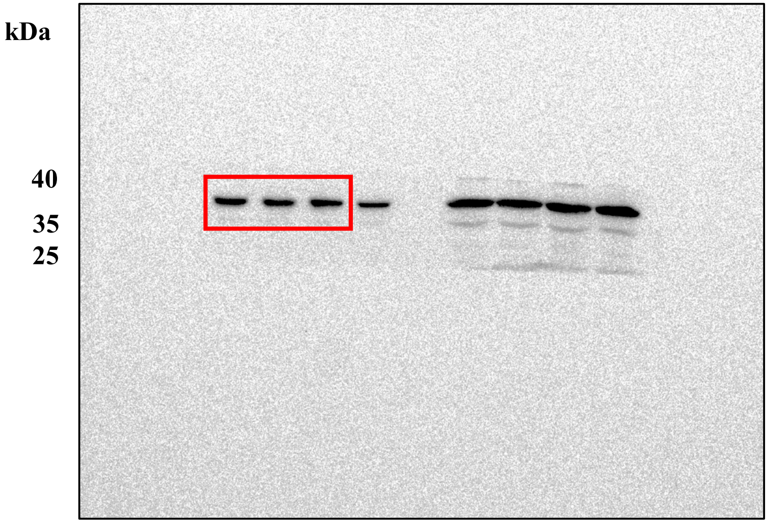

Anti-GAPDH

HCT116

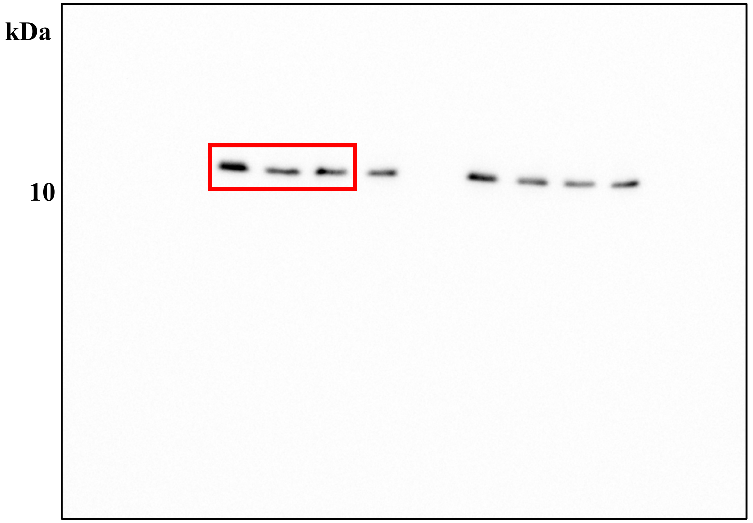

Anti-SRP9

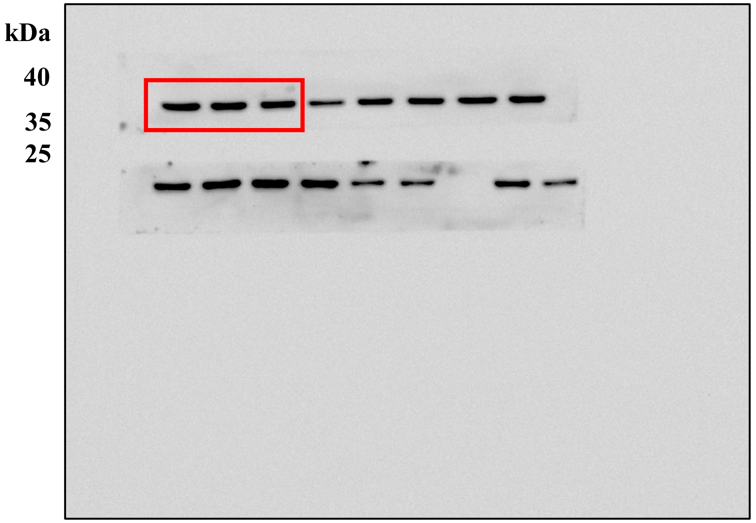

Anti-GAPDH

Sup N. Full unedited blots for Fig.S5

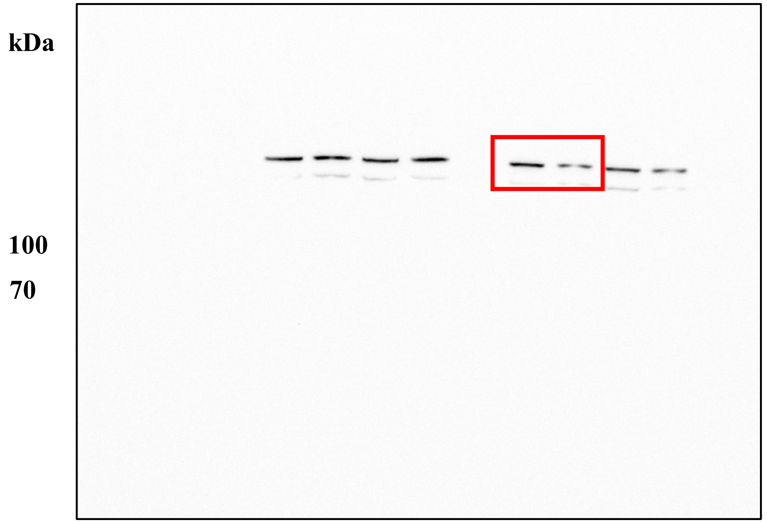

HT29 Anti-HSP90AA1

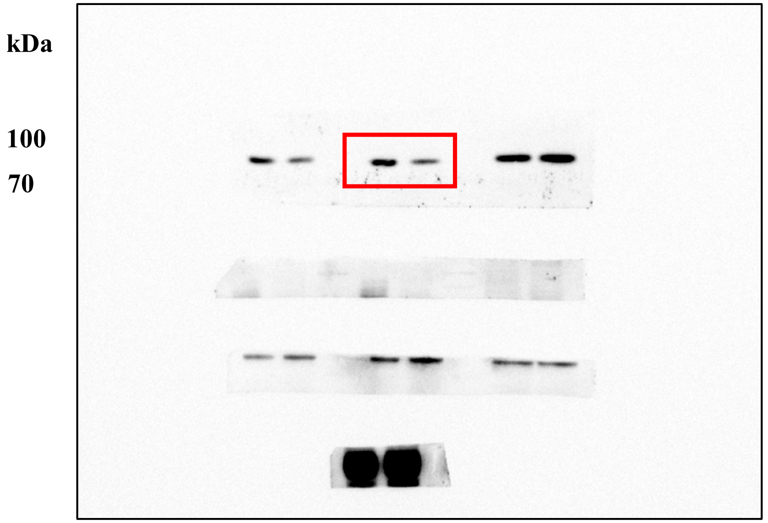

HCT116 Anti-HSP90AA1

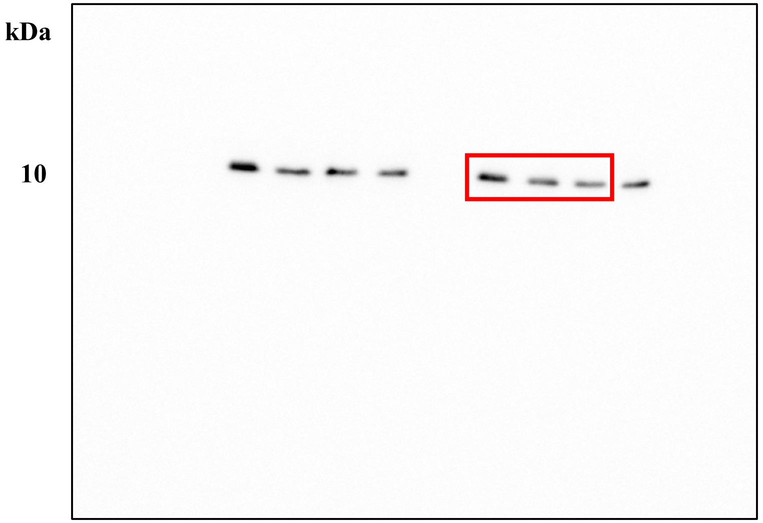

HCT116 Anti-SRP9

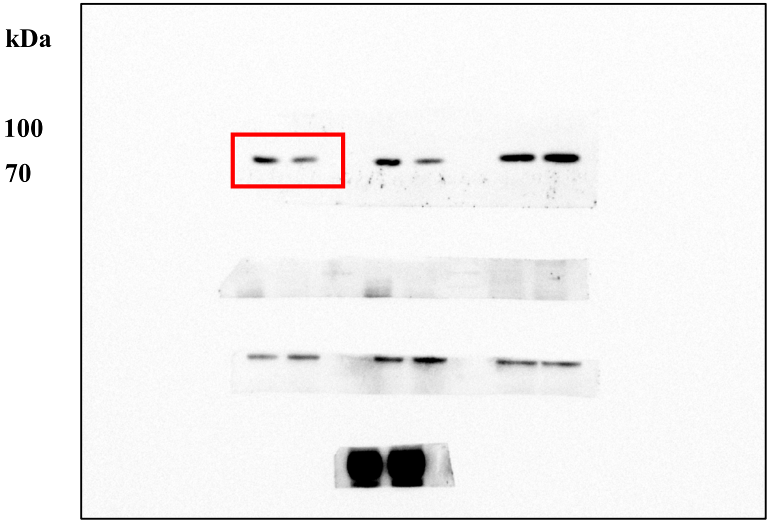

HT29 Anti-HSP90AA1

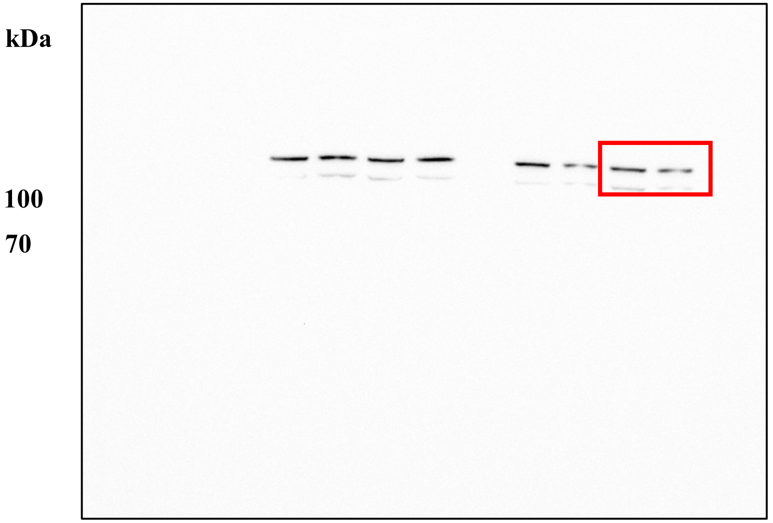

HCT116 Anti-HSP90AA1

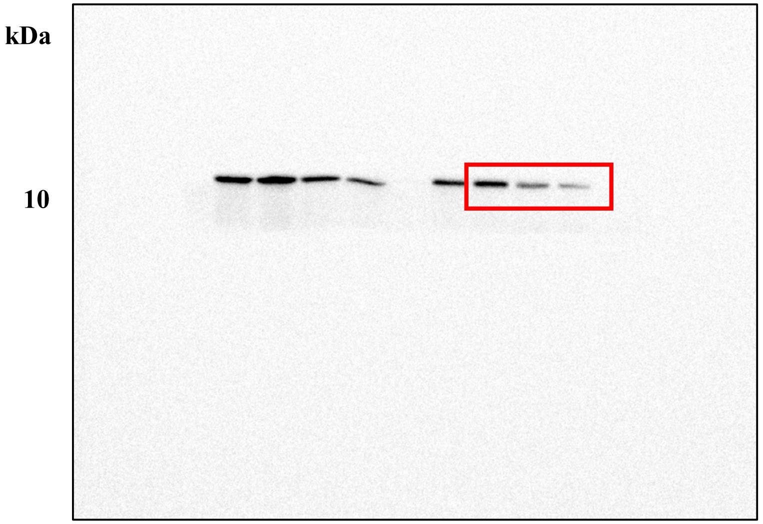

HCT116 Anti-SRP9
