# Supplementary material for: DAB2IP down-regulates HSP90AA1 to inhibit the malignant biological behaviors of colorectal cancer
Source: BMC Cancer. 2022 May 19;22:561. doi: 10.1186/s12885-022-09596-z (PMC9118737; doi:10.1186/s12885-022-09596-z)

A. Full unedited blots for Fig.1k DAB2IP

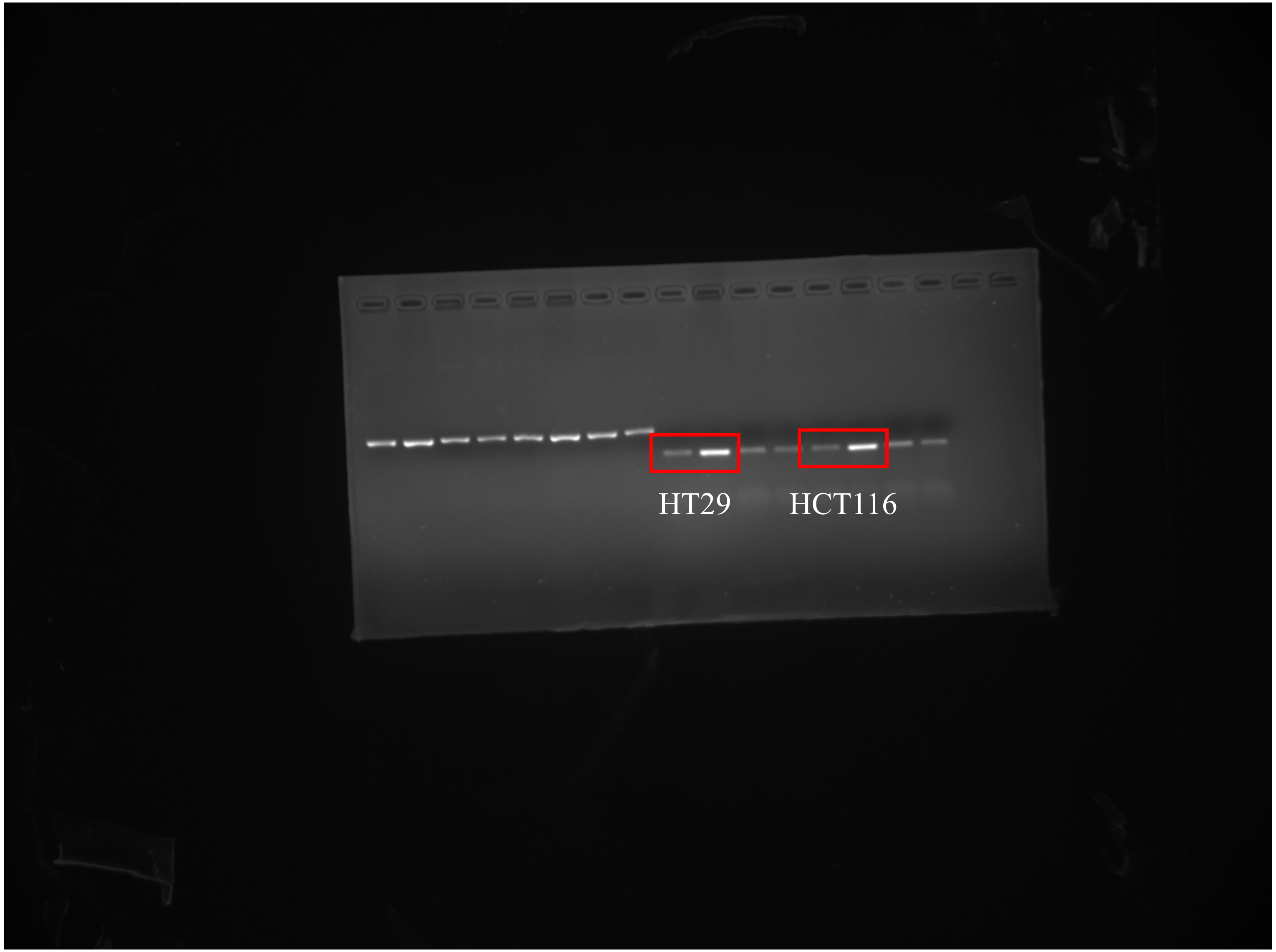

B. Full unedited blots for Fig.1k HSP90AA1

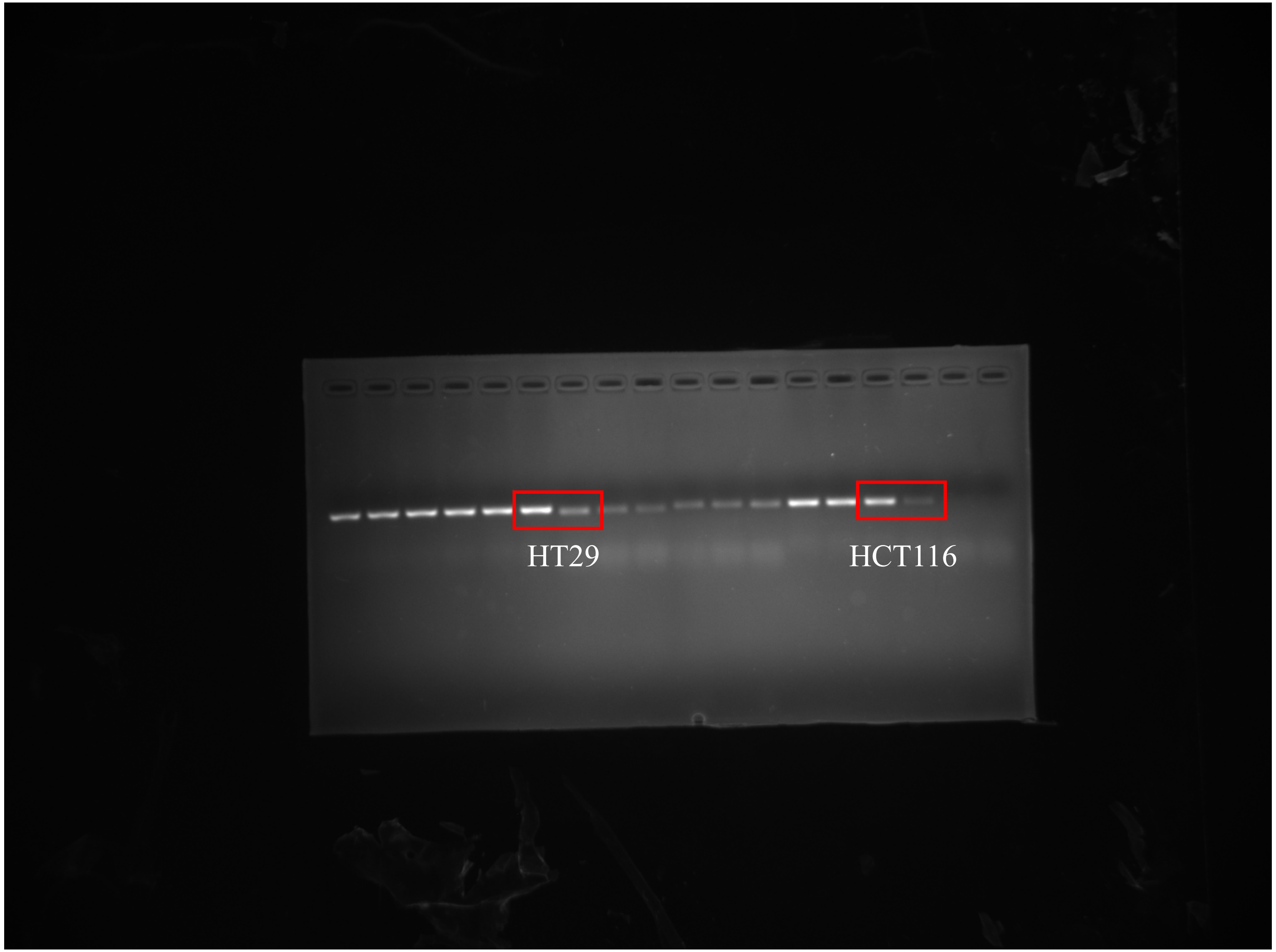

C. Full unedited blots for Fig.1k GAPDH

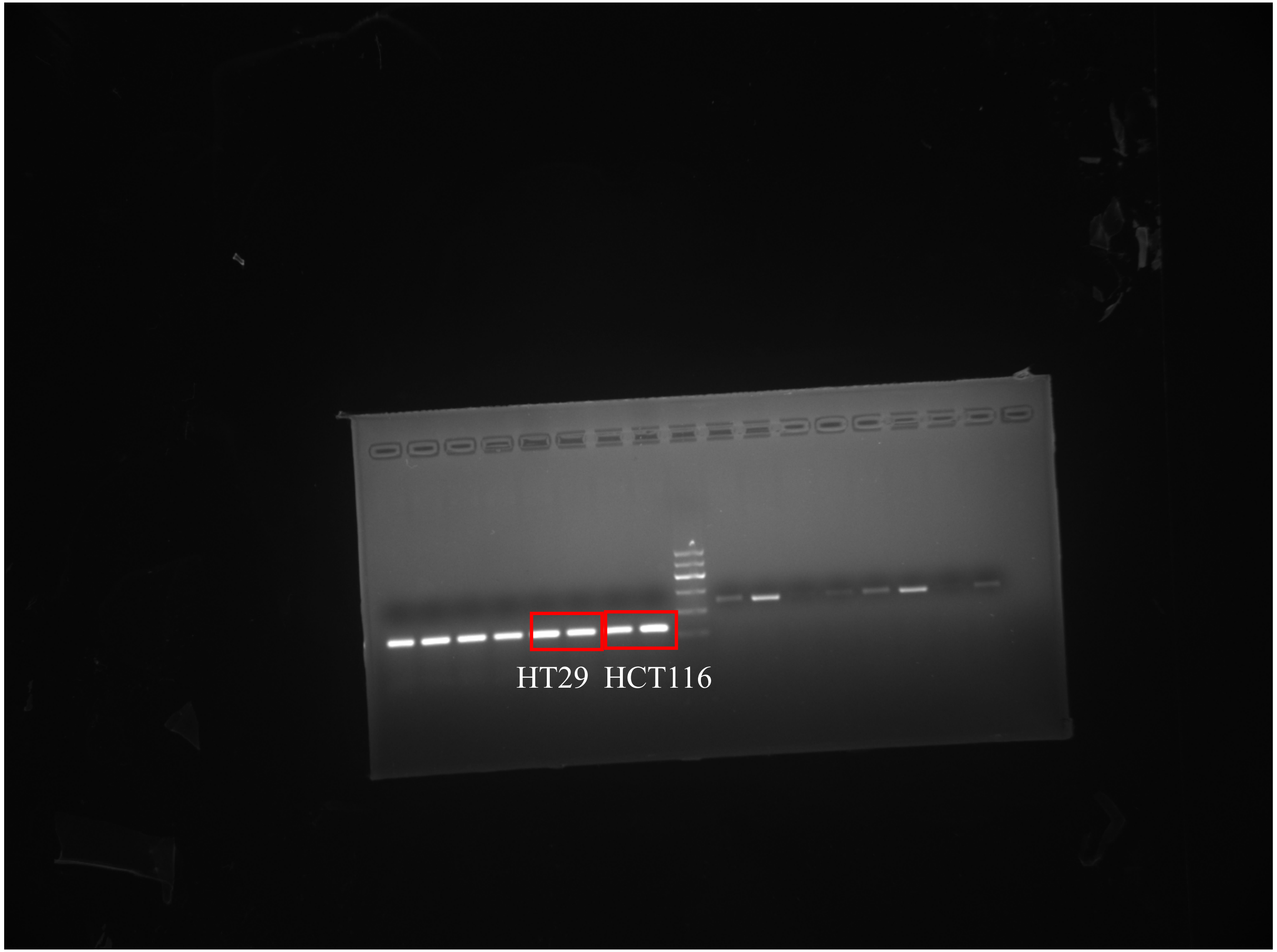

D. Full unedited blots for Fig.1m DAB2IP

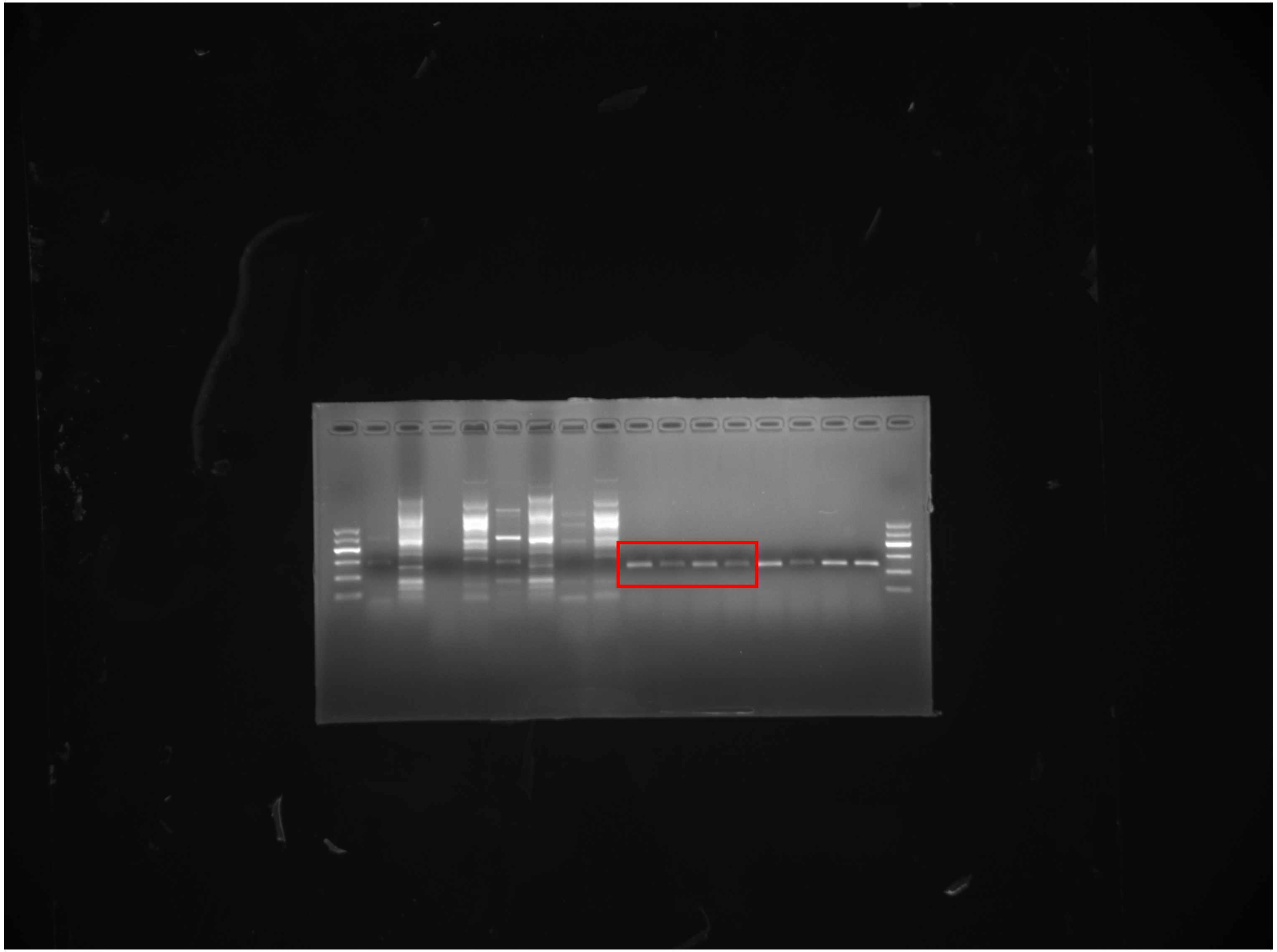

E. Full unedited blots for Fig.1m HSP90AA1

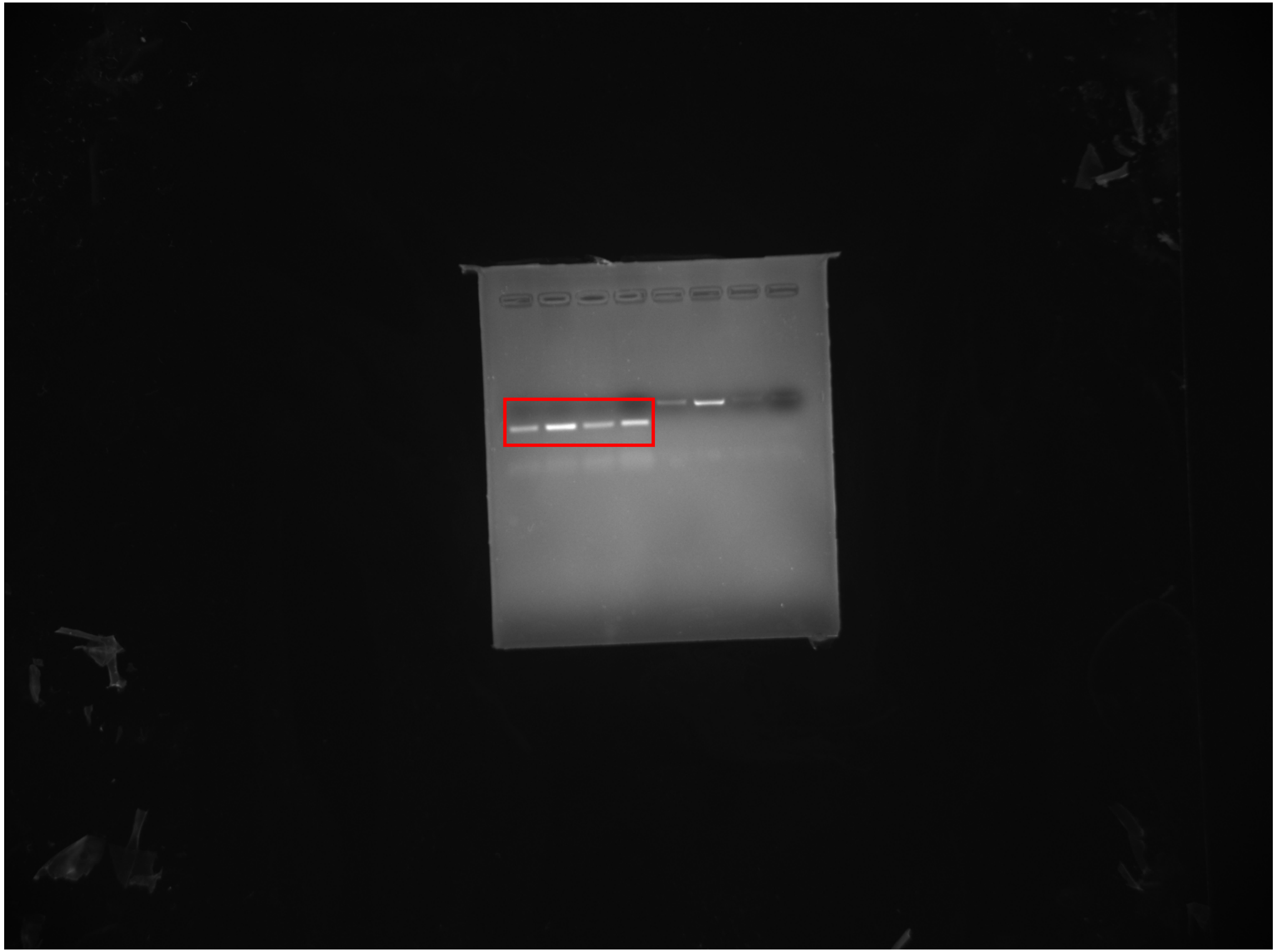

F. Full unedited blots for Fig.1m GAPDH

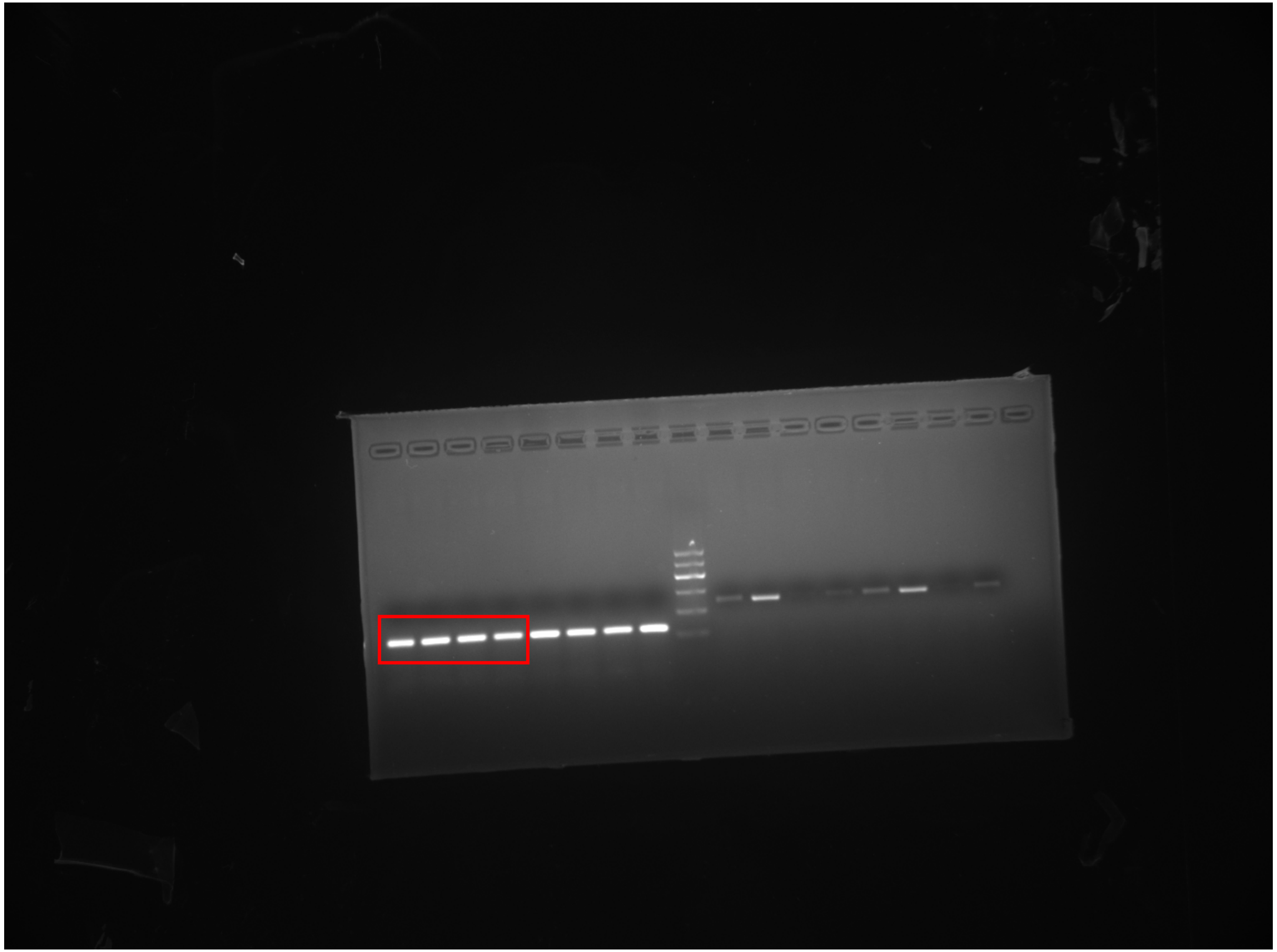

G. Full unedited blots for Fig.4a HSP90AA1

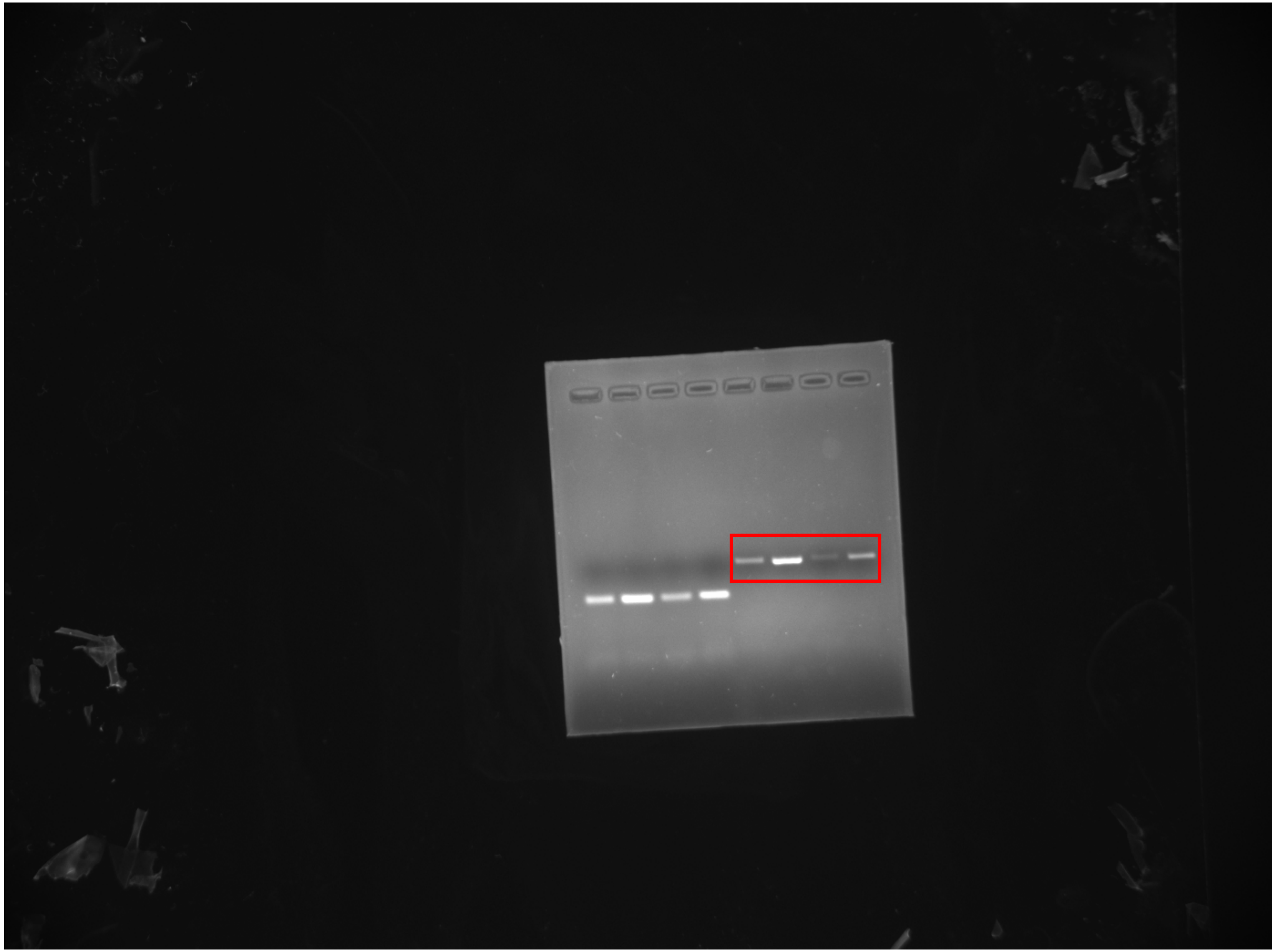

H. Full unedited blots for Fig.4a SRP9

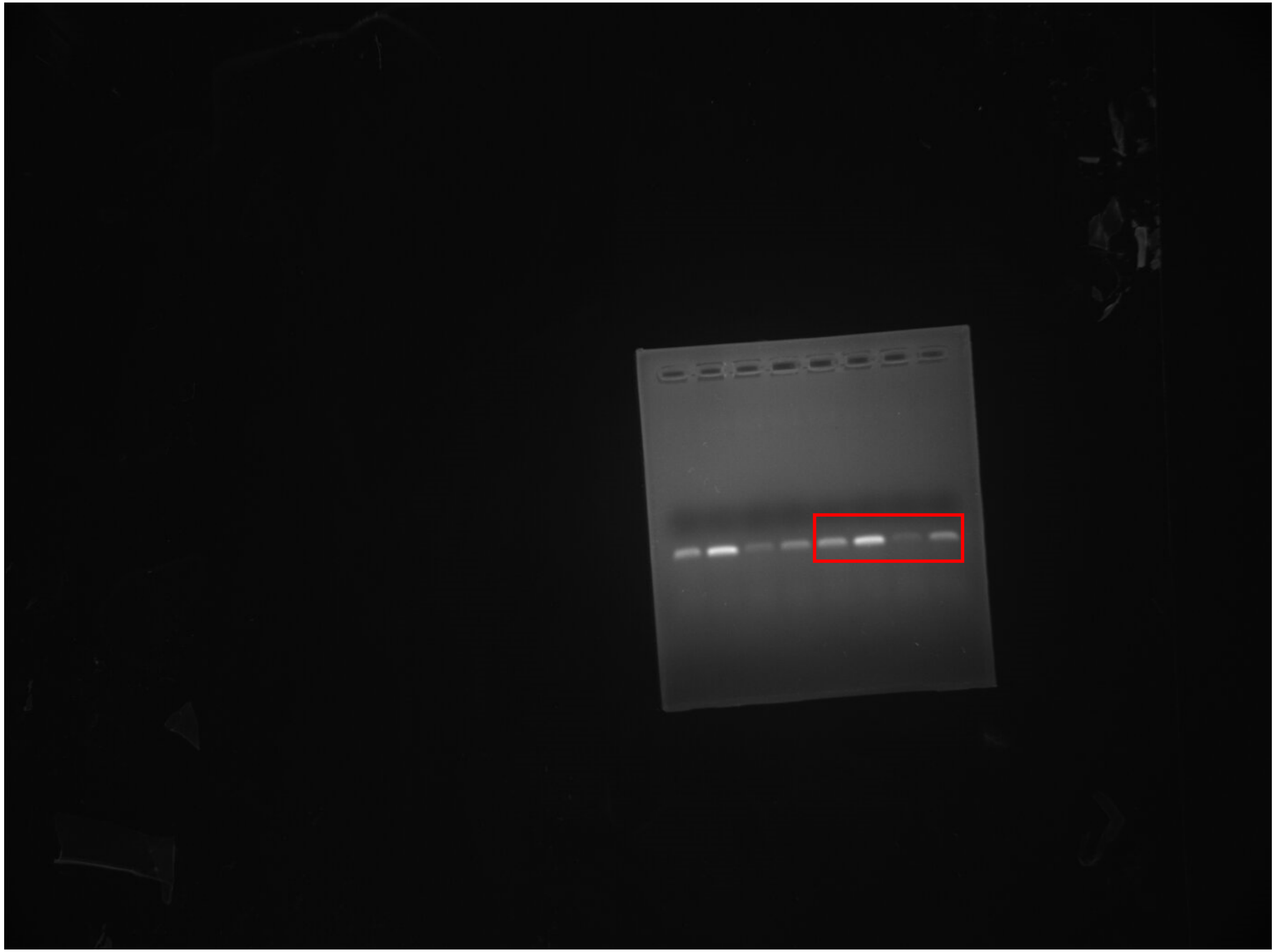

I. Full unedited blots for Fig.4a GAPDH

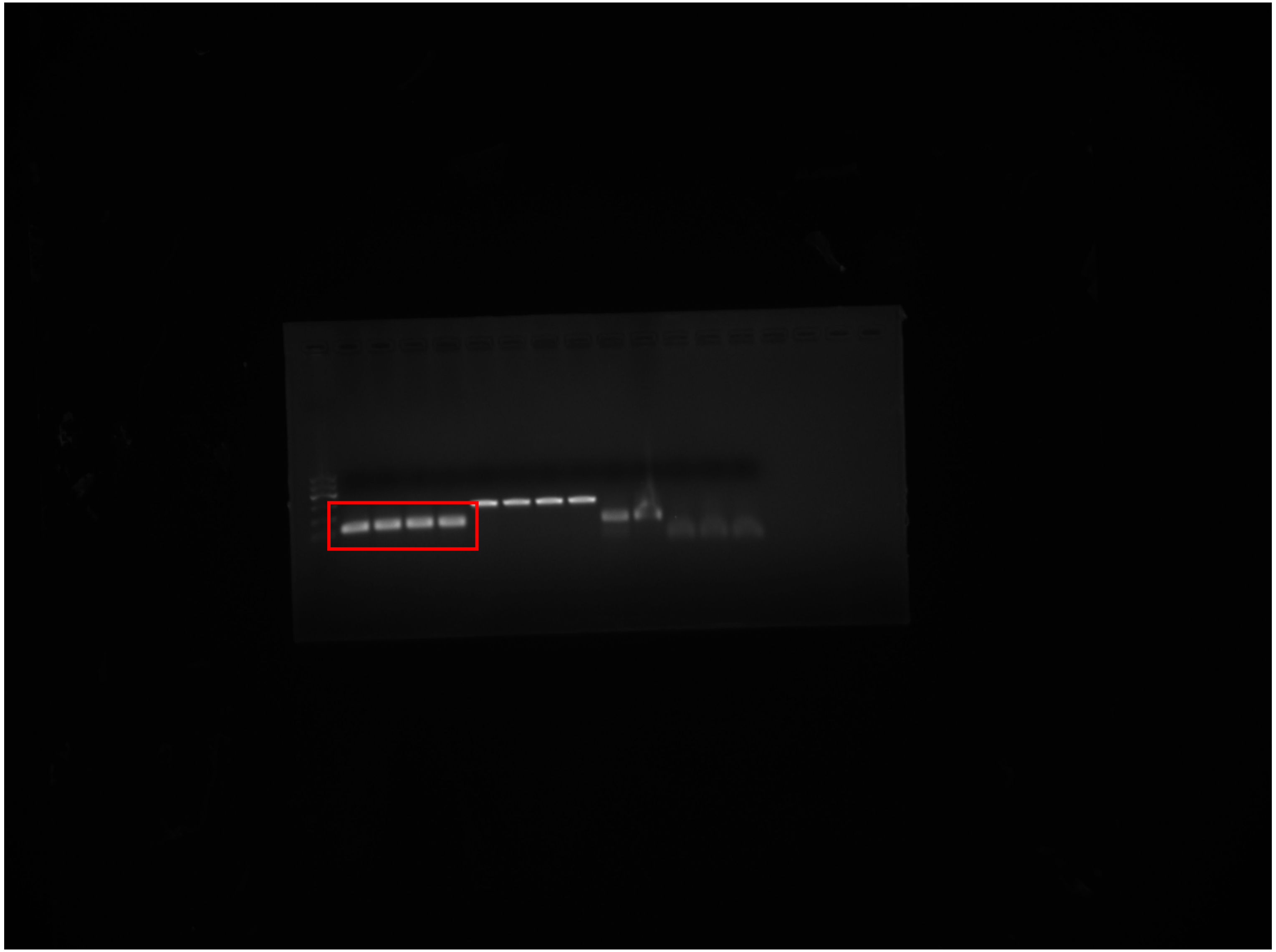

J. Full unedited blots for Fig.4a HCT116 HSP90AA1

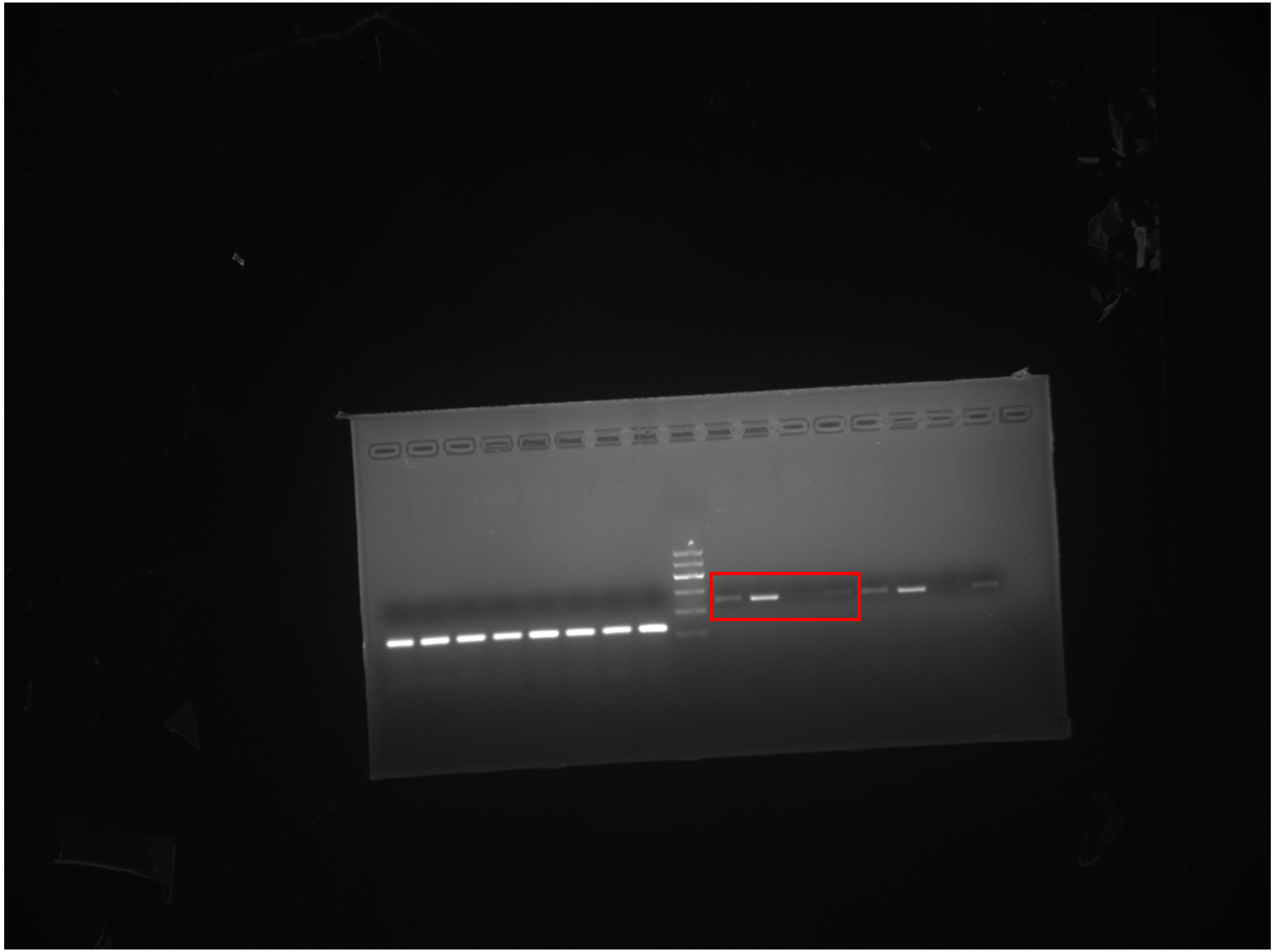

K. Full unedited blots for Fig.4a HCT116 SRP9

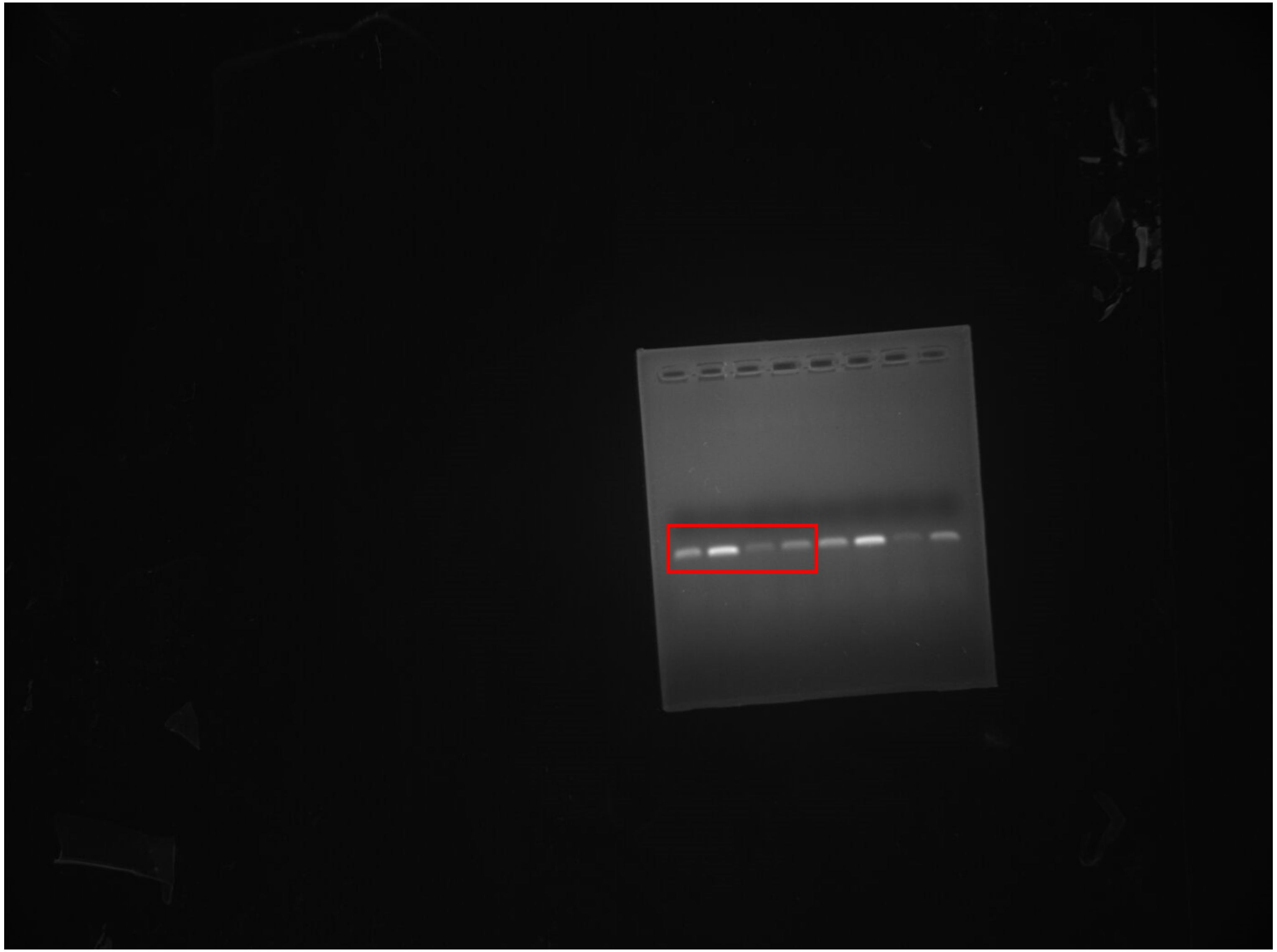

L. Full unedited blots for Fig.4a HCT116 GAPDH

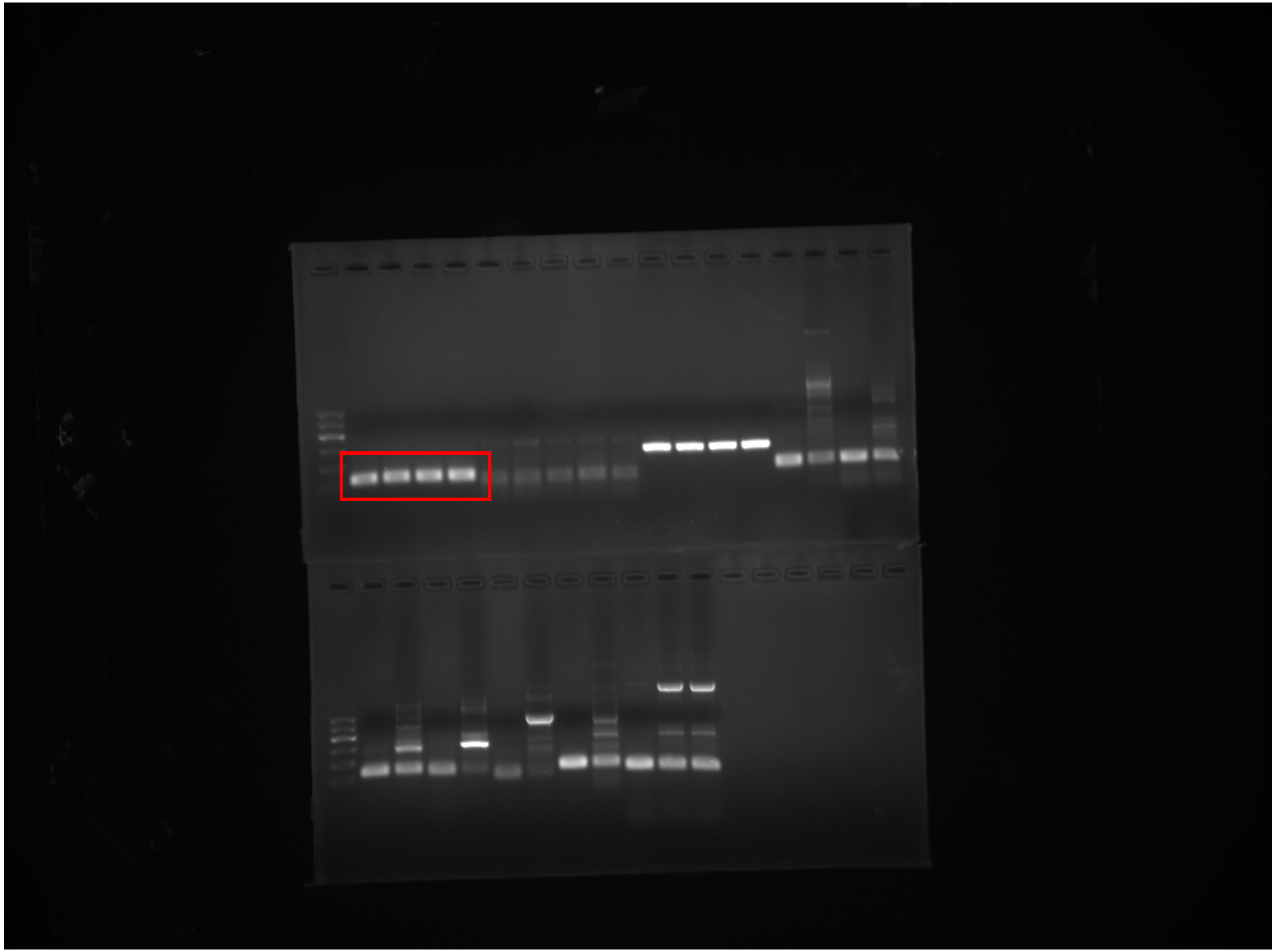

N. Full unedited blots for Fig.S2 HSP90AA1

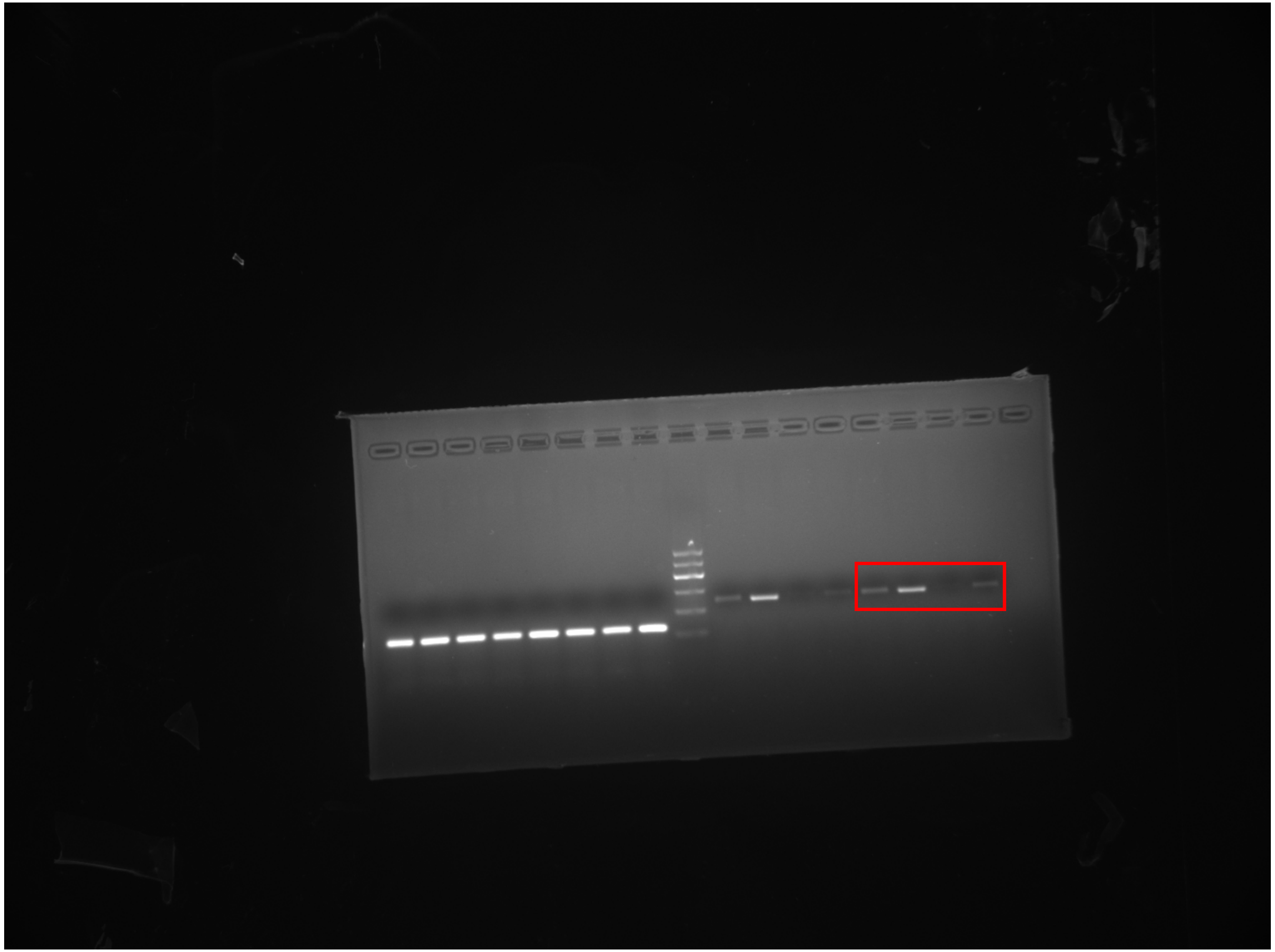

M. Full unedited blots for Fig.S2 GAPDH

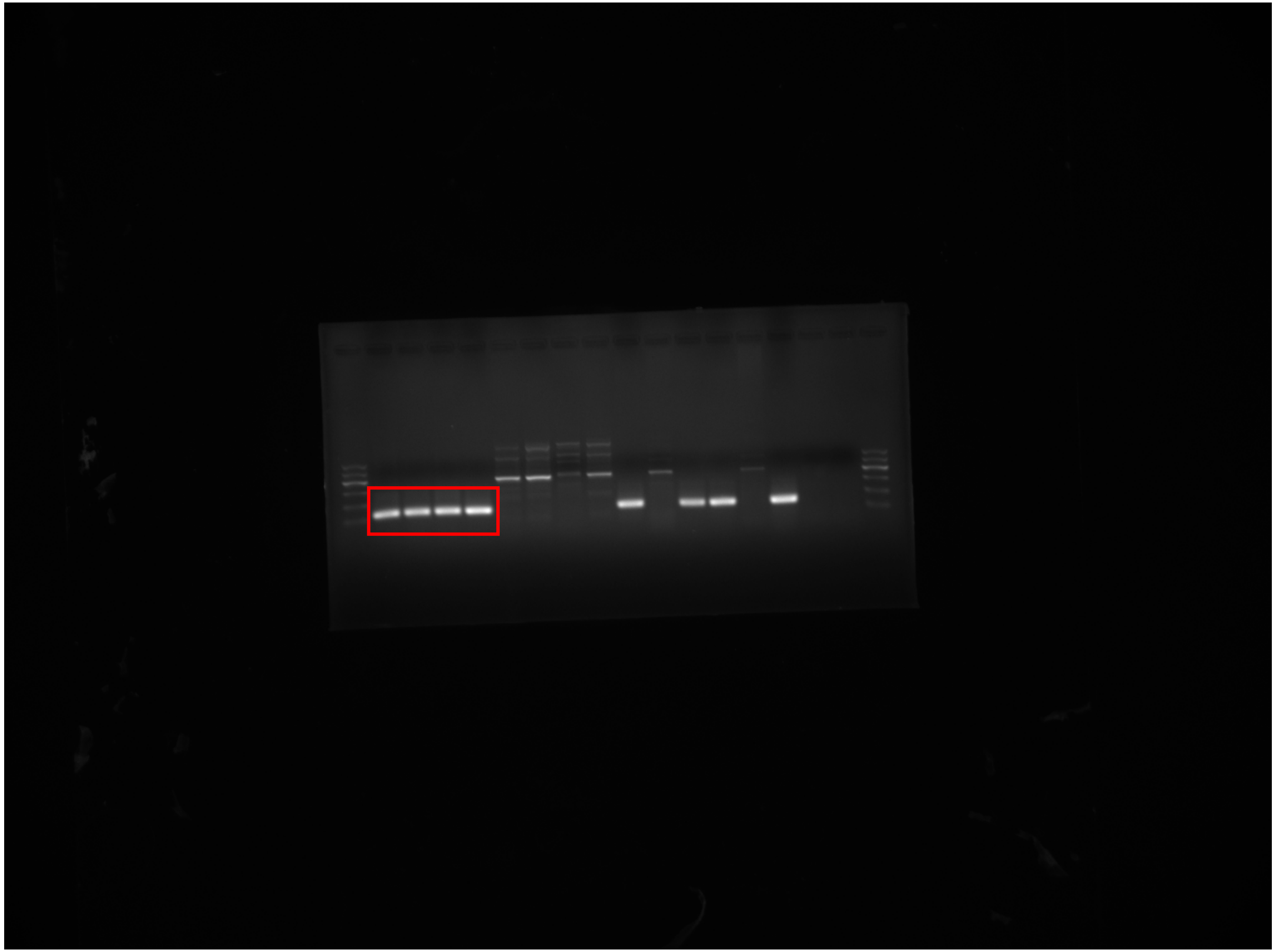

O. Full unedited blots for Fig.S5c

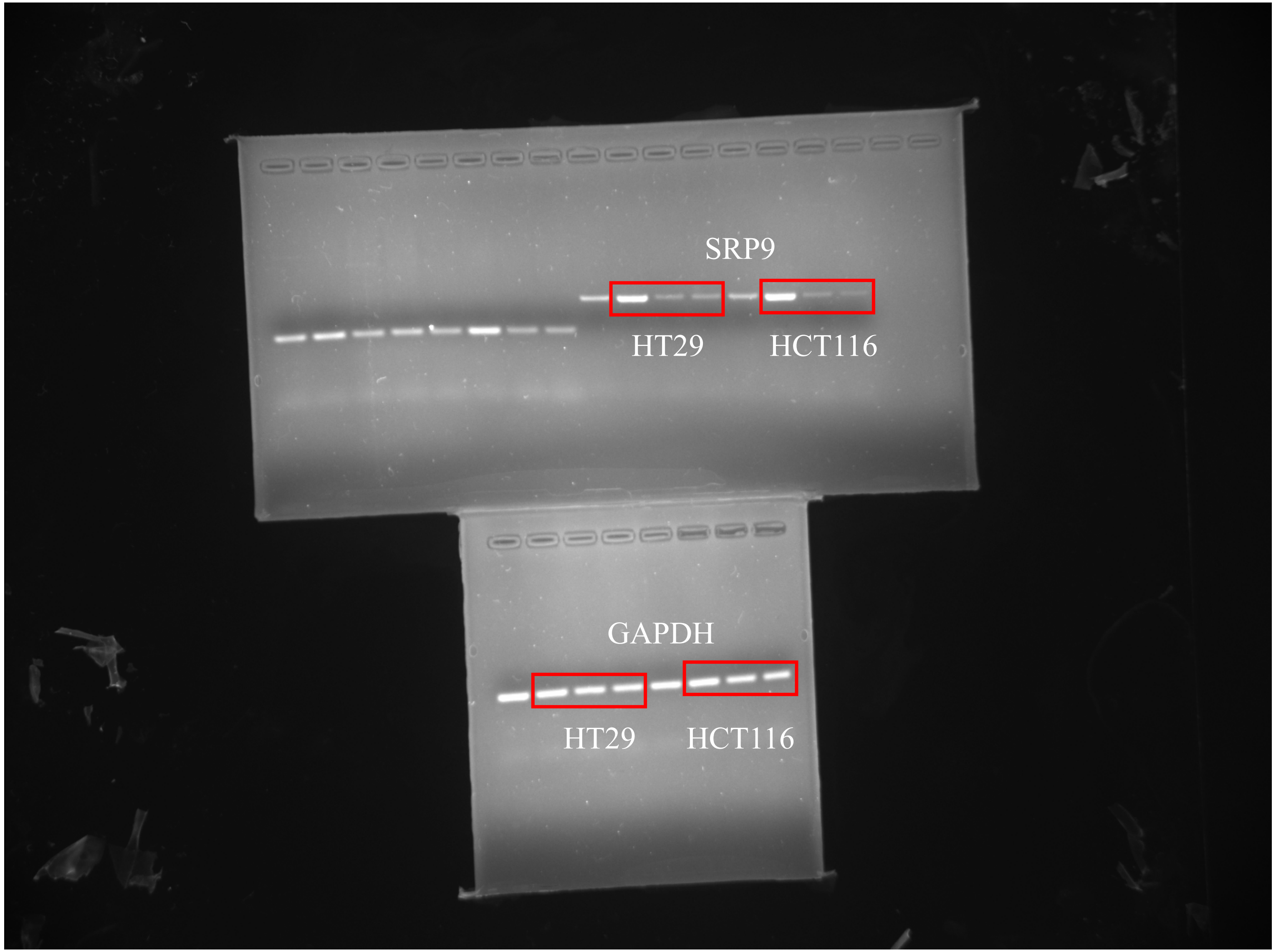

Supplement: Supplementary file 3 — Additional file 3. [file 12885_2022_9596_MOESM3_ESM.pdf]
